# Catalytic Enantioselective [6π] Photocyclization Reactions by Chromophore Activation with a Chiral Lewis Acid

**DOI:** 10.1021/jacs.5c17390

**Published:** 2025-12-05

**Authors:** Dominik Grünwald, Chithra Mohan Jayakumari, Noah Jeremias, Julian Zuber, Christopher J. Stein, Thorsten Bach

**Affiliations:** † Department Chemie and Catalysis Research Center (CRC), School of Natural Sciences, 9184 Technische Universität München, D-85747 Garching, Germany; ‡ Atomistic Modelling Center, Munich Data Science Institute, Technische Universität München, D-85748 Garching, Germany

## Abstract

The [6π] photocyclization of 3-[(1,1′-biphenyl)-2-yl]-2-methylcyclopent-2-enones was found to proceed enantioselectively, when a chiral AlBr_3_-activated oxazaborolidine was employed as photocatalyst. A low Lewis acid catalyst loading (2.5–5.0 mol %) was sufficient to induce a high enantioselectivity in the photochemical reaction. A total number of 21 tetracyclic products were obtained as single diastereoisomers in 78–99% *ee* upon variation of the terminal phenyl ring in the substrate (39–83% yield). Evidence was collected that the chiral Lewis acid coordinates to the enone and induces a bathochromic shift, which allows for selective excitation of the substrate-catalyst complex at λ = 368 nm or λ = 405 nm. The substrate binds to the Lewis acid by coordination of its carbonyl oxygen atom to the Lewis acidic boron atom of the oxazaborolidine and by a noncovalent hydrogen bond of one of its methylene C–H atoms to the oxygen atom of the heterocycle. The latter interaction restricts rotation around the coordinative B–O bond and favors a distinct conformation. Quantum-chemical calculations suggest that the preferred binding mode of the substrate induces a helical conformation in which the *Re* face relative to the C2 carbon atom of the substrate is accessible for an attack in the photocyclization. The photocyclization step likely occurs on the triplet hypersurface and leads to an intermediate cyclohexadiene which undergoes a 1,5-hydrogen shift to restore the aromaticity in the two benzene rings of the product. The photocyclization at −80 °C was significantly slowed down by deuterium substitution at the terminal phenyl group of the substrate.

## Graphical abstract

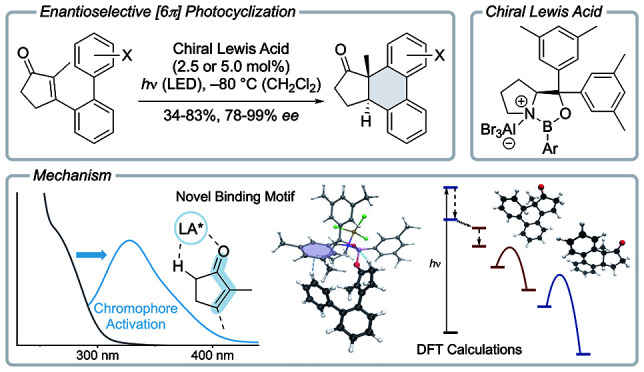

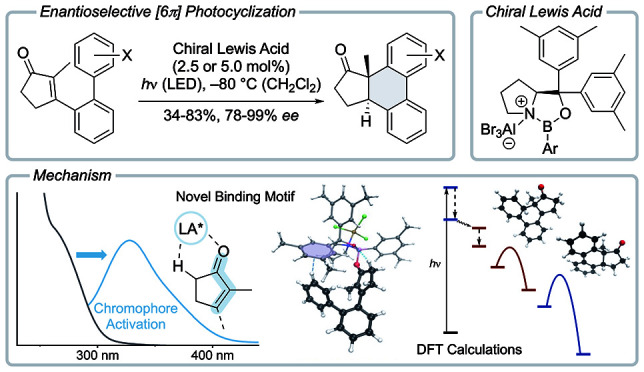

## Introduction

Compounds with an array of six conjugated π electrons can undergo a cyclization that represents a classical example of an electrocyclic reaction. In their landmark paper on the conservation of orbital symmetry,^1^ Woodward and Hoffmann discussed the photochemical [6π] cyclization as an illustrative case for a cyclization occurring in a concerted, conrotatory fashion. They explicitly mentioned the cyclization and cycloreversion reactions in the Vitamin D series,^2^ a photochemical cyclohexadiene→hexatriene reaction,^3^ the photocyclization of *cis*-stilbene,^4^ and Chapman’s work on the photocyclization of *N*-alkenyl anilines.^5^ Although many additional [6π] photocyclizations have been discovered and exploited in organic synthesis since the 1960s,^6^ the mechanistic analysis of the reaction has remained largely unchanged. Upon excitation of a substrate represented by general structure **I** (Scheme 1), the double bonds linked by Y rotate in the same direction (conrotatory) forming an intermediate *rac*-**II**. In an ensuing suprafacial migration, which is classified as a 1,4- or 1,5-hydrogen shift depending on whether Y represents one or two atoms, the aromaticity within the arene is restored. Although compound *rac*-**III** should be the primary product of the reaction, many [6π] photocyclizations lead partially or even predominantly to the *cis*-isomer *rac*-**III′**, because the proton transfer to the formal anionic center in *rac*-**II** occurs intermolecularly or because there is an epimerization upon workup.

**Scheme 1:**
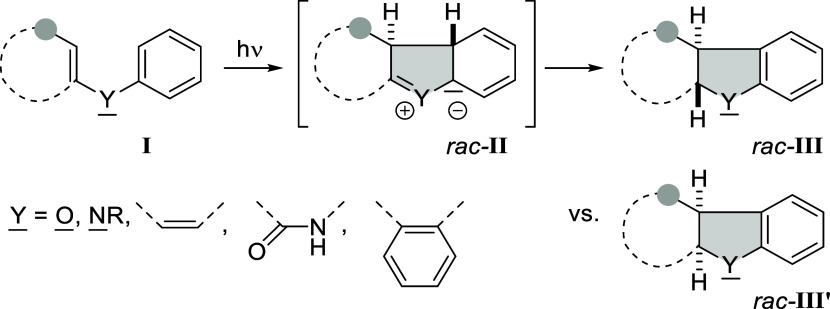
General Mechanistic Scheme for a [6π] Photocyclization Reaction Leading to Products *rac*-**III** and *rac*-**III′**

While the traditional view on the [6π] photocyclization suggested the reaction to be a concerted process from an excited singlet state, it has become evident that many [6π] photocyclizations occur from the first excited triplet state T_1_.^7^ This holds in particular, if a carbonyl group is part of the π-system (grey colored circle = CO) and the substrate is an α,β-unsaturated carbonyl compound, which undergoes rapid intersystem crossing (ISC).^8^ In the course of a [6π] photocyclization, two new stereogenic centers are formed and the C–C bond forming step is responsible for the absolute product configuration. Even though the [6π] system **I** seems planar as drawn in Scheme 1, it adopts a helical orientation which facilitates bond formation. Since the helix is intrinsically chiral,^9^ attempts have been made to prearrange substrates of a [6π] photocyclization in the solid state and to perform an enantioselective reaction.^10^ In this context, Toda and co-workers recorded a high enantiomeric excess (*ee*) in the [6π] photocyclization of 2-arylthio-3-methylcyclohexen-1-ones,^11^ *N*-phenyl enaminones,^12^ and *N*-acrylanilides^13^ when performed in an inclusion crystal with chiral host molecules. Early attempts to achieve an enantioselective^14^ [6π] photocyclization in solution revolved around the use of chiral templates^15^ or catalyst-mediated triplet state energy transfer to the substrate.^16^ While conceptually interesting, the enantioselectivity remained moderate (up to 60% *ee*). In more recent years, three key discoveries demonstrated impressively the feasibility of catalytic approaches toward an enantioselective [6π] photocyclization reaction (Figure 1). Smith, Paton and co-workers reported on the formation of tricyclic products **1** from the respective alkenoic acid anilides in which the alkene ring was part of an oxygen heterocycle (2,3-dihydrofuran or 3,4-dihydro-2*H*-pyran).^17^ Enantioface differentiation was achieved by a scandium Lewis acid with *N*,*N′*-dioxide **2** ^18^ serving as the chiral ligand. Coordination in a chelating fashion leads to a lowering of the triplet state energy^19^ which facilitates triplet energy transfer from the iridium cocatalyst. The six-membered dihydropyrans (*n* = 2) performed much better (72–90% *ee*) than a five-membered dihydrofuran (*n* = 1) which gave only 14% *ee*. Calculations supported the mode of action and identified the ring-closing step as enantiodetermining. Yoon, Baik, and co-workers employed chiral iridium complex **4** with a hydrogen bonding pyrazole in the enantioselective [6π] photocyclization to products **3**.^20^ Here, the pyrazole recruits the substrate via its imidazole nitrogen atom, and the chiral-at-metal complex is responsible for C–C bond formation at the *Si* face of the enone. DFT calculations suggested the 1,4-hydrogen shift to be the rate-determining step for formation of the major product. Enantioselectivities were generally high for all reported examples but the diastereoselectivities were variable. The most recent study on the topic was performed by the groups of Gilmour and Neugebauer who used aluminum-salen complex **6** to catalyze the [6π] photocyclization.^21^ The substrates were secondary and tertiary alkenoic acid anilides with the olefin being part of a carbocyclic ring. Upon ring closure, they delivered tricyclic products **5**, of which the six-membered (*n* = 2) alicyclic products gave the best enantioselectivities. The varying degrees of diastereoselectivity were explained by external protonation of the intermediate zwitterion competing with the 1,5-hydrogen shift. Coordination to the Lewis acid did not result in a bathochromic shift and, in agreement with computational data, an energy transfer pathway was proposed to operate. In all three examples, the formation of regioisomers is inevitable when the aryl substituent X is meta-positioned in the substrate, and the respective products were obtained as mixtures of regioisomers.

**Figure 1:**
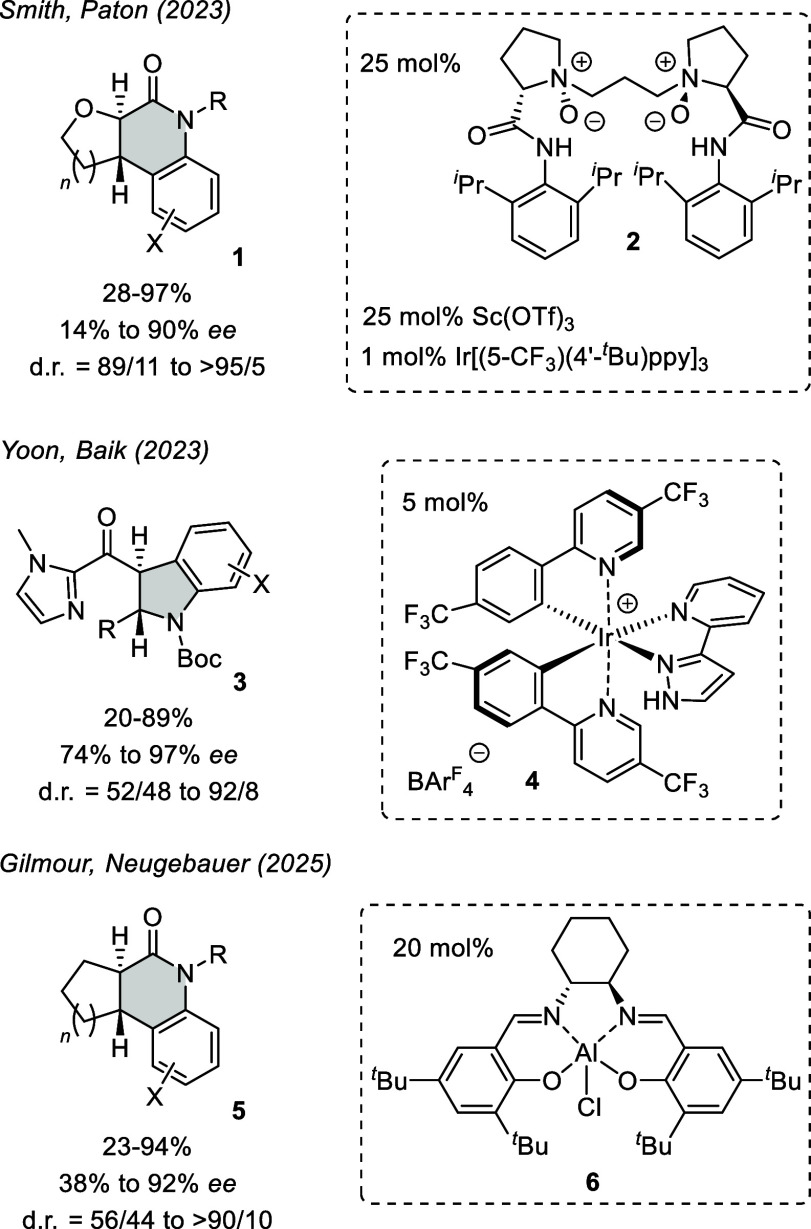
Products **1**, **3**, **5** of enantioselective [6π] photocyclization reactions obtained by applying a chiral ligand (**2**) or a chiral metal complex (**4**, **6**) in catalytic amounts. Abbreviations: d.r. = diastereomeric ratio; ppy = 2-phenylpyridine; BAr^F^ = tetrakis­[3,5-bis­(trifluoromethyl)­phenyl]­borate.

Our renewed^15b,16^ interest in [6π] photocyclization reactions was kindled by the recent discovery that chiral AlBr_3_-activated oxazaborolidines coordinate to photochemically reactive ketones not only via the expected Lewis acid–base interaction of boron center and carbonyl oxygen atom but also by a hydrogen bond from an adjacent sp^3^-C–H bond to the oxygen atom of the oxazaborolidine.^22^ 2-Acetonaphthones **7** underwent a highly enantioselective intramolecular *ortho* photocycloaddition, the enantioselectivity of which relied on this binding motif as shown for Lewis acid **8**·AlBr_3_ (Figure 2). We hypothesized that a similar hydrogen bond might form in cyclic enones such as compound **9a** when coordinated to an AlBr_3_-activated oxazaborolidine,^23,24^ e.g., **10**·AlBr_3_. It had been previously found that enones can bind in two different modes to Lewis acids, such as **10**·AlBr_3_, one being convex relative to the bicyclic oxazaborolidine core, the other concave.^25^ Since the binding mode depends on the nature of the substituents, we were optimistic that one binding mode would prevail for a given Lewis acid. If this was the case, we envisioned that (a) a notable bathochromic shift would be observed, which would allow for selective excitation of the Lewis acid-substrate complex, and that (b) the ensuing cyclization would occur enantioselectively within the constraints of the chiral Lewis acid.

**Figure 2:**
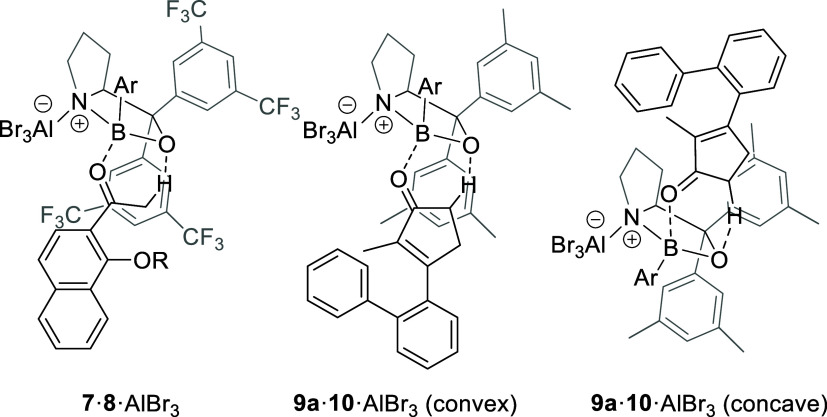
Previously studied complex of 2-acetonaphthone **7** to chiral Lewis acid **8**·AlBr_3_ (Ar = 6-fluoro-2-trifluoromethylphenyl) as a key intermediate in an enantioselective *ortho* photocycloaddition reaction and putative complexes of enone **9a** to chiral Lewis acid **10**·AlBr_3_.

The choice of 2-methylcyclopent-2-enones **9** as substrates for the [6π] photocyclization was based on the fact that the compounds display only one set of C–H bonds in α-position for a coordination and that they have been previously shown to undergo the desired racemic reaction^26^ upon direct excitation. We now report in detail on the search for a suitable Lewis acid for the enantioselective [6π] photocyclization of compounds **9**, on the structure elucidation of the products, on mechanistic studies, and on quantum-chemical calculations on the reaction course.

## Results and Discussion

### Optimization of Reaction Conditions, Product Scope, and Structure Elucidation

The chromophore of compound **9a** is a cyclic α,β-unsaturated ketone, an enone, which features a forbidden *n*π* transition as its longest wavelength absorption at λ = 300–350 nm.^27^ Not surprisingly, we found the photocyclization to be complete after 1 h of irradiation with fluorescent lamps displaying an emission maximum at λ = 350 nm (for details on the emission properties of the light sources, see the Supporting Information). The expected product *rac*-**11a** was isolated in 95% yield (Scheme 2).

**Scheme 2:**
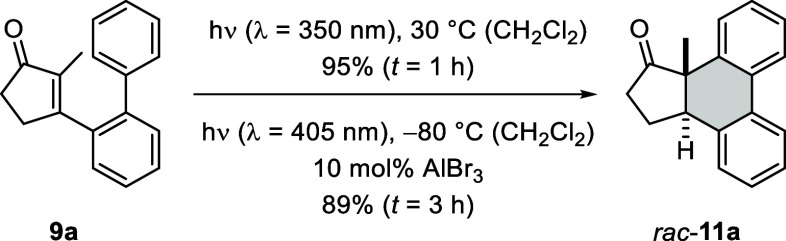
[6π] Photocyclization of 3-[(1,1′-Biphenyl)-2-yl]-2-methylcyclopent-2-enone (**9a**) to Racemic Product *rac*-**11a** with and without Lewis Acid Catalysis

Catalytic activity can be achieved in photochemical reactions if a Lewis or Brønsted acid shifts the absorption of a given chromophore to longer wavelengths. In this scenario, a wavelength needs to be identified at which the substrate itself is unreactive, while the Lewis acid-substrate complex can be selectively excited. The chromophore is activated by the acid toward excitation.^28^ For substrate **9a**, UV–vis data (Figure 3) indicated that its absorption completely ceases at wavelengths λ > 350 nm. In fact, irradiation by a light emitting diode (LED) with an emission maximum at λ = 405 nm did not lead to a notable conversion. In stark contrast, addition of 10 mol % AlBr_3_ facilitated a complete conversion of substrate **9a** within 3 h resulting in a yield of 89% for product *rac*-**11a**. The results match the absorption properties of the compound in the presence of the Lewis acid (Figure 3, blue-shaded area) which indicates that the absorption of the Lewis acid complex **9a**·AlBr_3_ is shifted into the visible region and has a notable overlap with the emission spectrum of the LED (Figure 3, orange-shaded area). Remarkably, we could in the current study record a spectrum of a putative oxazaborolidine-AlBr_3_ complex with the substrate (Figure 3, purple-shaded area). The data indicated that there was a chance for direct excitation of the complex with a visible light LED. However, even if catalysis was achieved, it would not necessarily guarantee a high enantioselectivity.

**Figure 3:**
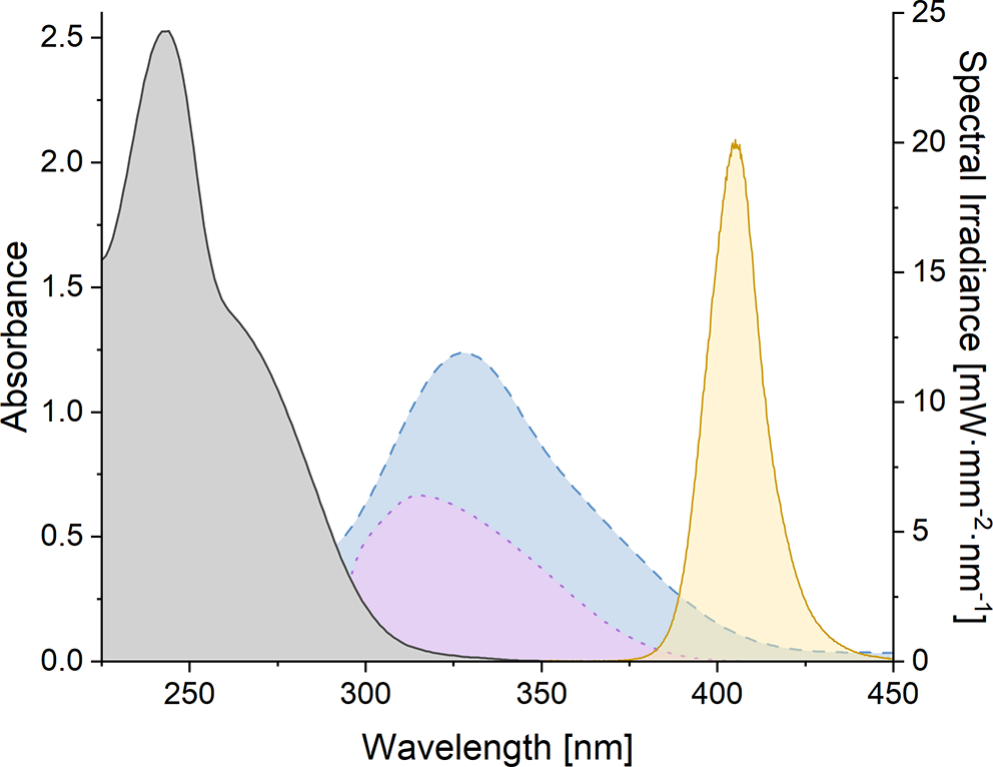
UV–vis spectrum of compound **9a** (gray shaded) in dichloromethane solution (*c* = 1.0 mM). The blue-shaded area covers the UV–vis spectrum of the same compound in the presence of AlBr_3_ (10 mM) and the purple-shaded area in the presence of a mixture of an oxazaborolidine (10 mM) and AlBr_3_ (5 mM). The orange-shaded area displays the emission spectrum of the 405 nm LED used in some of the optimization studies (*vide infra*).

Oxazaborolidine formation is typically performed by condensation of an aryl boronic acid and an amino alcohol followed by Lewis acid activation.^29^ Catalyst screening experiments commenced with an evaluation of different (*S*)-proline-derived amino alcohols, from which bis­(3,5-dimethylphenyl)-(*S*)-pyrrolidin-2-ylmethanol emerged as the preferred choice (see the Supporting Information for details). A set of oxazaborolidines **10** was subsequently generated by varying the aryl boronic acid ArB­(OH)_2_ and was tested upon activation with AlBr_3_ in the envisaged [6π] photocyclization (Table 1). The first set of experiments was performed at λ = 398 nm to guarantee a short reaction time and to avoid catalyst decomposition. The oxazaborolidine was used in excess relative to AlBr_3_ to minimize potential racemic reactions catalyzed by the achiral Lewis acid. Since previous work with cyclic enone substrates had required relatively large catalyst loadings (25–50 mol %),^30^ the experiments were initially performed with 30–50 mol % of the oxazaborolidine (OAB, entries 1–4). The best enantioselectivity (90% *ee*) was observed with catalyst **10b**·AlBr_3_ being used in 15 mol % (entry 3).

**Table 1:**
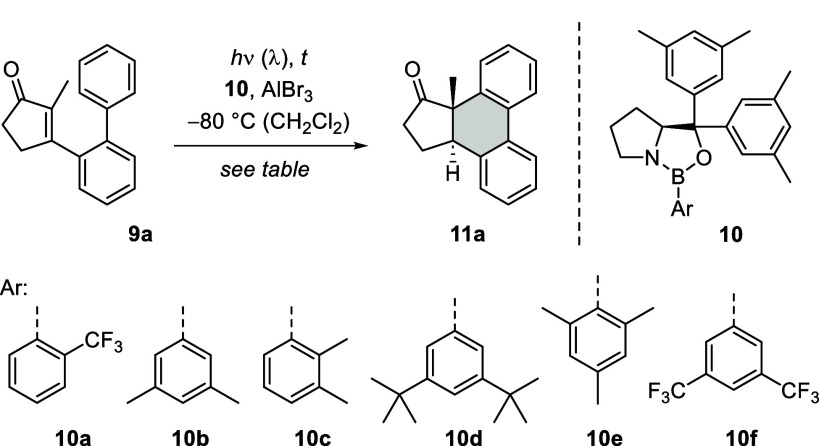

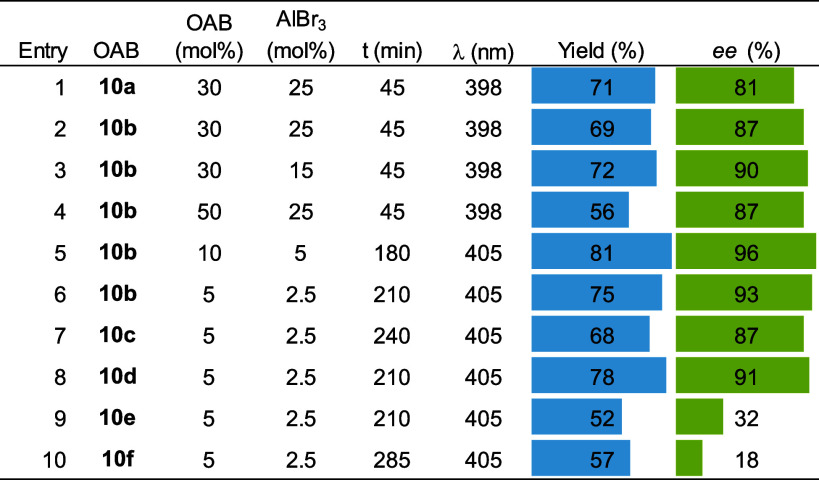
Optimization of the Enantioselective [6π] Photocyclization **9a** → **11a**

A significant improvement was subsequently achieved by running the reaction at longer wavelength. Despite the slightly extended reaction times, catalyst stability did not seem to be an issue, and the reactions performed with catalyst **10b**·AlBr_3_ at loadings of either 5 or 2.5 mol % were both high yielding and highly enantioselective (entries 5, 6). Never had an oxazaborolidine-catalyzed photochemical reaction in our hands given this high an enantioselectivity (96% *ee*) with this low a catalyst loading. Although the performance of oxazaborolidine **10b** was close to perfection, some other aryl boronic acids were employed that led to representative oxazaborolidines **10c**-**10f** (entries 7–10, see the Supporting Information for further variations). Only the 3,5-di-*tert*-butylphenyl-substituted compound came close to the results achieved before (entry 8). The important role that the aryl group Ar plays became evident with oxazaborolidines **10e** and **10f** which performed far worse than compound **10b**. Since the emphasis was on enantioselectivity and low catalyst loading, the conditions of entries 5 and 6 were selected to screen other 2-methylcyclopent-2-enones **9** in the enantioselective [6π] photocyclization (Scheme 3). It turned out that the substitution pattern at the peripheral arene ring influences the absorption properties of the compounds notably. Electron-withdrawing substituents shift the absorption to shorter wavelengths, which required an adaptation of the light source. Hence, an LED displaying an emission maximum at λ = 368 nm was consistently used in this case. Since the bathochromic absorption shift induced by the Lewis acid is roughly identical for every substrate, the observed Δλ becomes smaller at shorter wavelengths. In other words, the perfect situation shown in Figure 3, in which there is no overlap of the emission wavelength and the enone absorption, was not always met, and racemic background reactions compete with the catalytic process. We opted to keep the catalyst loading low even though the latter situation in some cases compromised the enantioselectivity of the [6π] photocyclization.

**Scheme 3:**
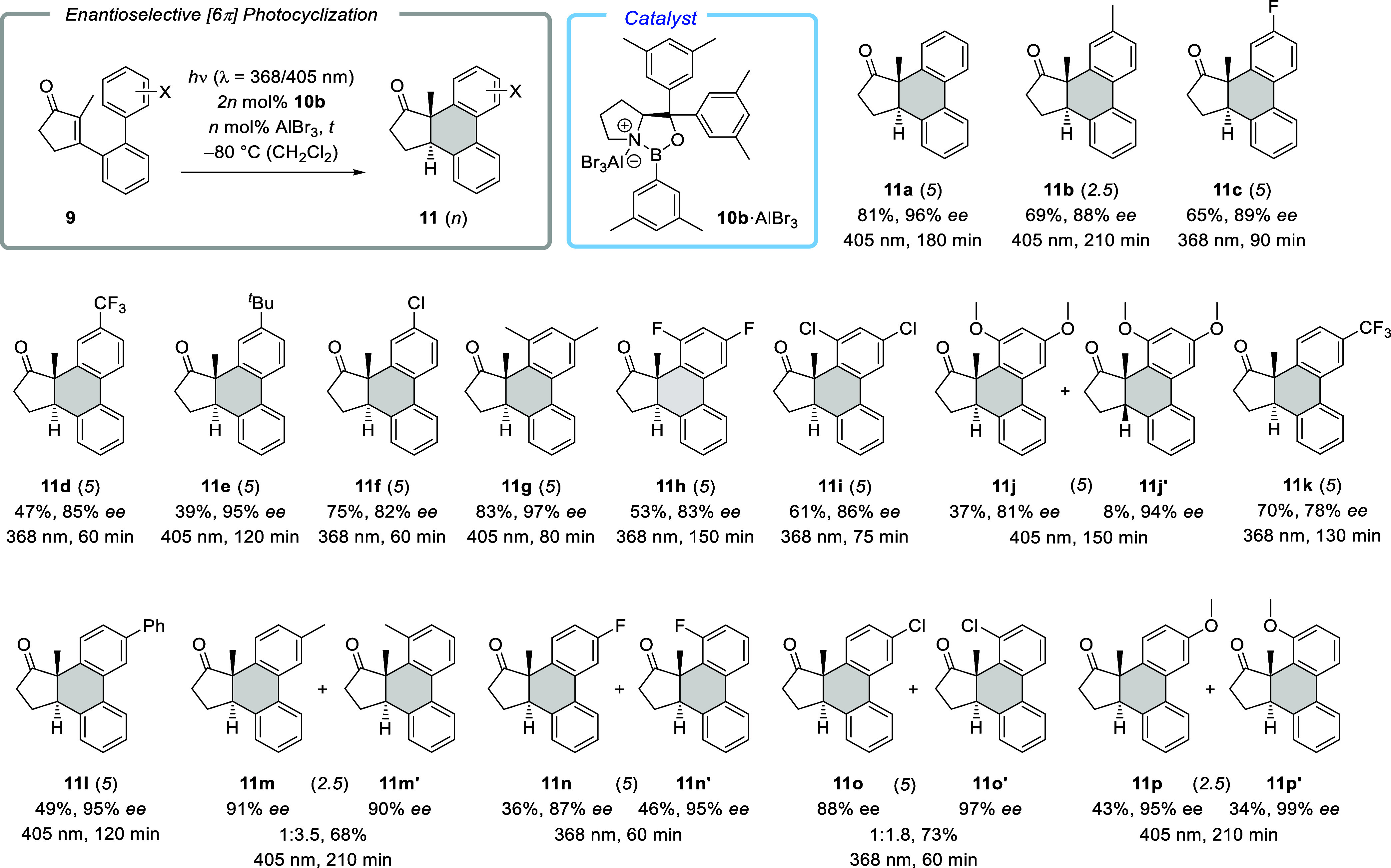
Enantioselective [6π] Photocyclization of Substituted 3-[(1,1′-Biphenyl)-2-yl]-2-methylcyclopent-2-enones **9**

In general, the overall yield for the isolated [6π] photocyclization products **11** varied between 39% and 83%. Substrates bearing a single substituent in *para*-position or two substituents in a 3,5-relationship delivered a single product. The methyl-substituted product **11b** was obtained in 88% *ee* at a catalyst loading of only 2.5 mol %. The *tert*-butyl-substituted product **11e** was isolated in 95% *ee* under standard conditions and the 3,5-dimethyl-substituted product **11g** in 97% *ee*. Fluorine (**9c**), trifluoromethyl (**9d**), or chlorine (**9f**) *para*-substitution in the substrate led to the mentioned hypsochromic shift, and the respective enones were irradiated at λ = 368 nm. Product yields were moderate to good (47–75%), but the enantioselectivity decreased slightly (82–89% *ee*) as compared to the alkyl-substituted products.

The same trend was observed for halo-3,5-disubstitution, i.e., for difluoro compound **11h** (53%, 83% *ee*) and for dichloro compound **11i** (61%, 86% *ee*). The 3,5-dimethoxy-substituted substrate **9j** could be irradiated at standard conditions but gave surprisingly not only the *trans*-substituted product **11j** but also its *cis*-epimer **11j’**. The latter product was formed with high enantioselectivity (94% *ee*). In all other cases, the simple diastereoselectivity of the transformation was high, and a single diastereoisomer was obtained (d.r. >95/5). If the terminal phenyl group was *meta*-substituted, product mixtures resulted from a cyclization in *para*- or in *ortho*-position relative to the existing substituent. In cases where the two regioisomers were separable, the yield for each of them was determined for the isolated regioisomer, as was the *ee*. Monofluorinated products **11n** and **11n′** were isolated in 36 and 46%, respectively. Despite the short irradiation wavelength required, the enantioselectivities were remarkably high (87% *ee* and 95% *ee*). Also, the methoxy-substituted products **11p** and **11p′** were formed in excellent *ee* (95 and 99%) at a catalyst loading of only 2.5 mol %. If the products were not separable, as for **11m**/**11m′** and for **11o**/**11o′**, the yield was recorded for the mixture of regioisomers and their ratio was determined by NMR. The *ee* was in these cases typically measured after reduction to the alcohol (*vide infra*), and it was found to be high for all compounds (88–97% *ee*). As an exception, no regioisomers were observed for the *meta*-trifluoromethyl- and the *meta*-phenyl-substituted substrates **9k** and **9l**, which produced single products **11k** (70%, 78% *ee*) and **11l** (49%, 95% *ee*).

Reduction of ketones **11** occurred diastereoselectively and the resulting polar alcohols were in some cases easier to separate on chiral HPLC than the ketones. For the parent compound **11a**, the diastereoselectivity was particularly pronounced, and its reduction with sodium borohydride led to a single diastereomeric product **12a** (Scheme 4). The relative configuration was corroborated by NOESY spectra that suggested a *cis*-relationship of the adjacent hydroxy and methyl groups within the five-membered ring. Since initial attempts to obtain crystalline material for single-crystal X-ray diffraction (SC-XRD) were unsuccessful, we prepared the Mosher esters **13a** and **13b**.^31^ Proof of the absolute (*R*)-configuration at the stereogenic alcohol carbon atom provided indirect proof for the absolute configuration at the other stereogenic centers (see the Supporting Information for details). After some experimentation, it was eventually found that *para*-bromobenzoate **14** obtained by acylation of alcohol **12a** was a crystalline compound. Anomalous dispersion effects in diffraction measurements on the crystal delivered unambiguous proof of the absolute configuration, and the configuration assignment of **11a** was assumed to be likewise applicable to all other products **11**.

**Scheme 4:**
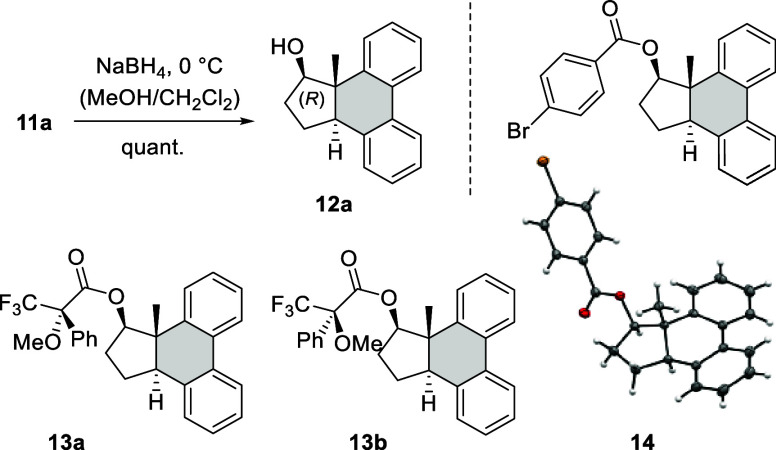
Reduction of Ketone **11a** and Consecutive Products **13** and **14** Employed for an Assignment of the Absolute Configuration

The absolute configuration found in product **11a** suggests that the [6π] photocyclization occurs at the C2-carbon atom of the enone from the *Re* face (Scheme 5). Conversion of intermediate **15a** to the final product involves a 1,5-hydrogen shift, which seems to be stereospecific for all products. Only in one instance (**11j′**), a *cis*-configured product was isolated in small quantities. In all other cases, the products are consistently *trans*-configured. If the opposite helical conformation **9a″** was preferred upon coordination to the catalyst, the opposite enantiomeric intermediate *ent*-**15a** and product *ent*-**11a** would be expected.

**Scheme 5:**
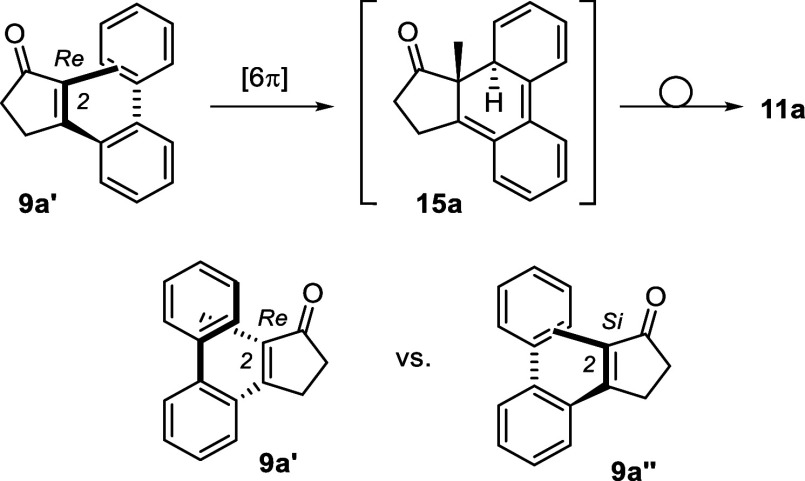
Absolute Configuration in Product **11a** Resulting from an Attack at Carbon Atom C2 from the *Re* Face in Conformation **9a′**

Key questions related to the mechanistic course of the reaction seemed to be best addressed by quantum-chemical calculations. In particular, the binding mode of substrate **9a** to the Lewis **10b**·AlBr_3_ had to be elucidated and was expected to provide an explanation for the observed enantioface differentiation.

### Quantum-Chemical Calculations on the Substrate-Catalyst Complexes and on the [6π] Photocyclization

We first addressed the course of the reaction in the absence of the Lewis acid. To rationalize the photochemical pathway, we calculated the free energy profile for the reaction with density functional theory (DFT) employing the ωB97X-D3 functional^32^ and def2-TZVP basis set.^33^ All calculations were carried out in the ORCA quantum-chemistry package version 5.0.4.^34^ Since initial TD-DFT calculations within the Tamm-Dancoff approximation revealed that the barrier for the photocyclization is roughly 10 kJ mol^–1^ higher for the S_1_ state than for the T_1_ state, we focused on a reaction mechanism involving the triplet manifold. This is further supported by a rather large spin–orbit coupling element hinting that the triplet manifold can be readily accessed. After an initial excitation to the close-lying bright S_2_ or S_3_ states, internal conversion leads to a relaxation to the S_1_ state from which the triplet manifold is populated by intersystem crossing (ISC), facilitated by a spin–orbit coupling element of 7.44 cm^–1^ at the minimum energy crossing point (Figure 4). Already before the actual bond formation in the photocyclization step, the C–C distance of the bond to be formed is reduced by 40 pm in the T_1_ minimum structure compared to its S_1_ counterpart (see the Supporting Information). The actual C–C bond formation then proceeds on the lowest triplet excited-state T_1_ with an electronic energy barrier of 29.8 kJ mol^–1^, which slightly increases to a free energy barrier of 35.3 kJ mol^–1^ when the effect of an implicit solvent model and free energy corrections were added. We calculated these corrections with the CPCM implicit solvation model^35^ with parameters for dichloromethane, and estimated the vibrational contribution to the free energy from harmonic vibrational frequencies calculated with the same ωB97X-D3/def2-TZVP DFT method (see the Supporting Information for further details) at –80 °C. After relaxation to the S_0_ ground state through another ISC, a 1,5-hydrogen shift leads to the final product **11a** with a high free energy barrier of 63.2 kJ mol^–1^. Naturally, in the absence of chiral information, product formation will be racemic. However, it can already clearly be seen from the calculation that the arbitrarily chosen substrate conformation **9a′** of substrate **9a** leads to the enantiomer **11a** as depicted in Scheme 5.

**Figure 4:**
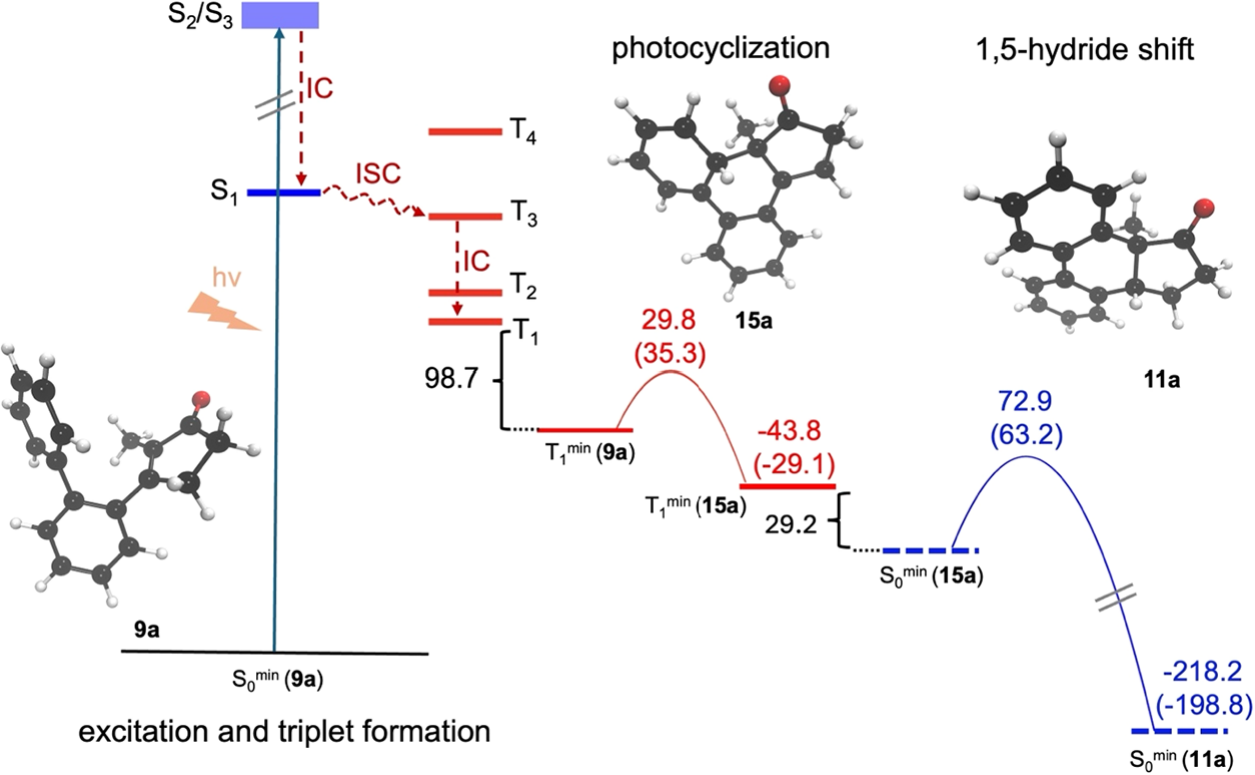
Energy profile (in kJ mol^–1^) of the uncatalyzed racemic reaction of substrate **9a** to product **11a**. Singlet state energies are shown in blue, whereas triplet state energies are shown in red. Barrier heights and product energies are given relative to the reactant structure of the respective elementary step in the same spin manifold. Values in brackets correspond to free energies, whereas the other values are pure electronic energies (see text and Supporting Information for more details on the methodology).

To rationalize the observed enantioselectivity in the catalytic reaction, we first identified the most stable S_0_ ground-state assemblies of substrate **9a** with the Lewis acid **10b**·AlBr_3_. Employing both an extensive search based on chemical intuition and on an automated CREST conformer search^36^ with the GFN2-xTB semiempirical method,^37^ we identified several low-energy conformers eventually leading to either enantiomer **11a** or *ent*-**11a** of the product. We then calculated their electronic energies with the same DFT method as above. For the two lowest-energy conformers **C1** and **C2**, we determined their respective free energies from a highly accurate composite scheme similar to our previous work.^22^ There, we recalculated the electronic energy with the accurate DLPNO–CCSD­(T) method^38^ and the cc-pVTZ and cc-pVTZ/C (auxiliary) basis sets,^39^ and added the same solvent and free energy corrections as described above. The concave binding mode was on average found to be more stable by ∼35 kJ mol^–1^ as compared to the convex binding mode (cf. Figure 2). This preference cannot be explained by a single stabilizing interaction absent in the convex binding mode, but it is rather the result of reduced strain or many slightly enhanced weak interactions when accommodating the B–O and O–H bonding interactions between substrate and catalyst. We also found that the hydrogen bond with the oxygen atom of the oxazaborolidine ring was more stable when formed with a hydrogen atom from the CH_2_ group neighboring the carbonyl group in substrate **9a**, rather than with a hydrogen atom of the methyl group. In conformer **C1**, the *Si* face of carbon atom C2 in conformation **9a″** is exposed to C–C bond formation, eventually leading to enantiomer *ent*-**11a**. Conformer **C2** induces a helical conformation **9a′** (cf. Scheme 5) of the substrate displaying the *Re* face for an attack and will lead to the observed major enantiomer **11a**. As highlighted in Figure 5, assembly **C2** is characterized by a CH-π-interaction between substrate and catalyst in addition to the hydrogen bond present in both conformers, leading to a stabilization of 8.10 kJ mol^–1^ in terms of its DLPNO–CCSD­(T) electronic energy compared to **C1**, which reduces to 3.81 kJ mol^–1^ once the free energy corrections are added in the full composite scheme. Conformer **C2** leads to the major enantiomer found in the experimental work. Tentatively assuming that the ground-state assembly determines the observed enantioselectivity, we can estimate the *ee* according to $\mathrm{ee}=\frac{1-\exp\left(-\Delta \Delta G/\mathrm{RT}\right)}{1+\exp\left(-\Delta \Delta G/\mathrm{RT}\right)}$ , with ΔΔ*G* being the free energy difference between both assemblies, *R* the ideal gas constant, and *T* the temperature. With this, we obtain an estimate *ee* value of 83% in good agreement with the even higher *ee* of 96% observed experimentally.

**Figure 5:**
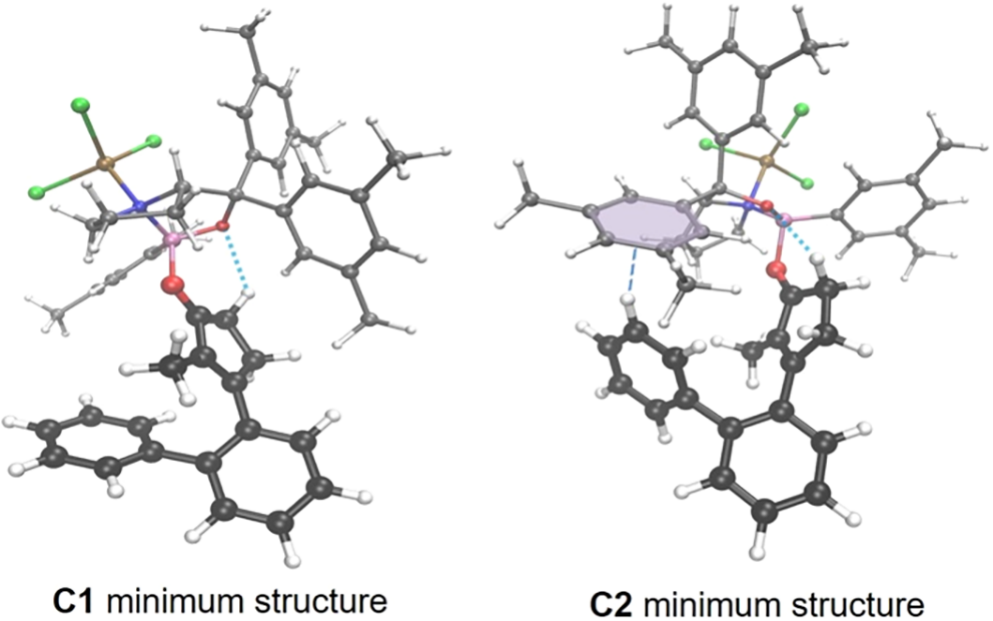
Ball-and-stick representation of the two lowest-energy conformers of the ground-state assembly of substrate **9a** with the Lewis acid **10b**·AlBr_3_ leading to the two enantiomers of product **11a**. Note the additional stabilizing CH-π-interaction in assembly **C2**.

Tracking both conformers over the same photochemical pathway as shown for the uncatalyzed reaction (cf. Figure 4), we find that the T_1_ transition-state barriers for **C1** and **C2** differ by 2.94 kJ mol^–1^ in favor of the **C2** conformer when calculated with DFT including solvation and free energy corrections (see the Supporting Information for details). This means that once the triplet manifold has been reached, the fraction of photocyclization reactions over other relaxation mechanisms is higher for the **C2** conformer than for the **C1** conformer. Since photocyclization in the **C2** conformer leads to the predominantly observed enantiomer **11a**, this further enhances the *ee*, bringing our theoretical estimate in closer agreement with the experimentally determined result. Support for the fact that a triplet pathway is followed even in the presence of Lewis acid **10b**·AlBr_3_ was gathered by performing qualitative quenching studies with piperylene. Unlike for a singlet reaction, the reaction was considerably slower in the presence of a triplet quencher.

### Studies on the Nature of the 1,5-Hydrogen Migration

Typically, the migration step required to complete the [6π] photocyclization (cf. Scheme 1) is considered to be a concerted hydrogen shift, in the present case a 1,5-migration. In an attempt to substantiate the intramolecular nature of this step, we subjected deuterated substrate **9a**-*d*_5_ to the conditions of the enantioselective [6π] photocyclization. The reaction proceeded more slowly than the reaction of substrate **9a** and delivered only 6% of product after an irradiation time of 90 min (Scheme 6A). 67% of substrate were reisolated. NMR analysis showed the deuterium incorporation to be incomplete, and the ratio of fully deuterated product **11a**-*d*_5_ to product **11a**-*d*_4_ was 26/74.

**Scheme 6:**
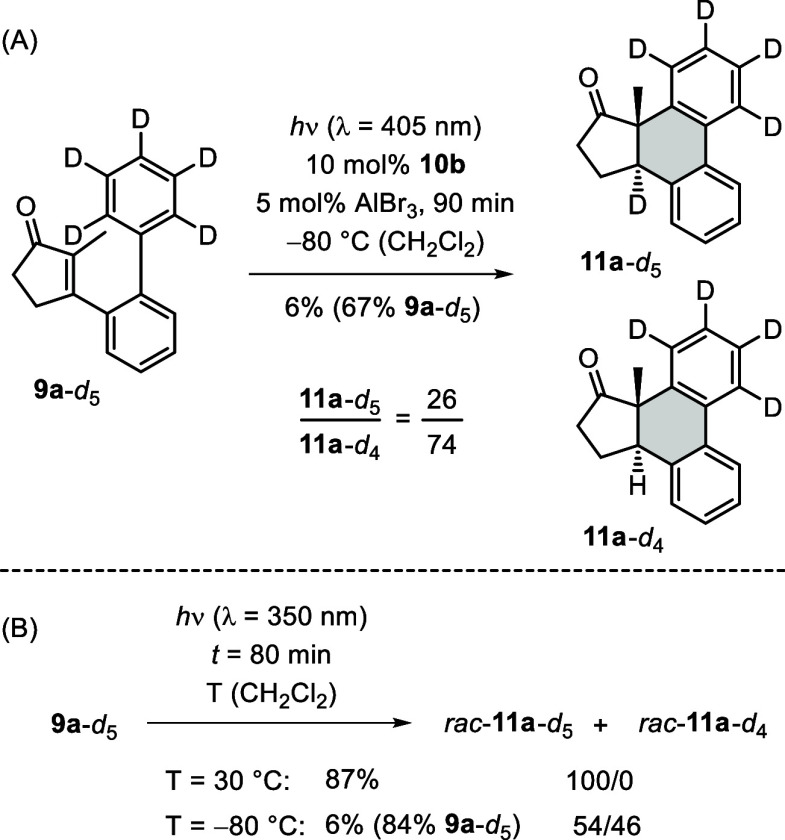
Catalytic [6π] Photocyclization Performed with Deuterated Substrate **9a**-*d*_5_ (A) and Temperature Influence on the [6π] Photocyclization in the Absence of a Catalyst (B)

The result indicated that deuteration inhibits a step in the [6π] photocyclization, and we performed for comparison the uncatalyzed reaction of the same substrate (Scheme 6B). In line with previous work,^26^ we observed at 30 °C a clean and complete conversion that led exclusively to the expected deuterated product *rac*-**11a**-*d*_5_. When the same reaction was conducted at −80 °C, it was found to be as sluggish as the catalytic reaction providing only 6% of product as a mixture of *rac*-**11a**-*d*_5_ and *rac*-**11a**-*d*_4_ in a ratio of 54/46. Lewis acid coordination can be ruled out as a possible reason for the incomplete conversion of substrate **9a**-*d*_5_, since the AlBr_3_-promoted reaction of **9a**-*d*_5_ at room temperature proceeded smoothly (see the Supporting Information for a complete set of results). The incorporation of a proton suggests that the reaction is stalled at the stage of the respective intermediate **15a**-*d*_5_ (cf. Scheme 5). In fact, the high free energy barrier (63.2 kJ mol^–1^) calculated for the 1,5-hydrogen shift renders a thermal reaction at low temperature (−80 °C) extremely slow. However, due to the rigidity of the transition state with a large imaginary frequency of 1377.59 cm^–1^ (Figure 6), we calculate a crossover temperature^40^ for hydrogen tunneling of 42.3 °C, much higher than the experimental conditions of −80 °C. In agreement with related observations on 1,5-hydrogen migration reactions,^41^ we tentatively propose that quantum mechanical tunneling^42^ is the dominating pathway for the 1,5-hydrogen shift reaction under catalytic reaction conditions. Figure 6 includes an overlay of the two structures before and after the hydrogen shift, along with the calculated root-mean-square deviation (excluding the transferred hydrogen) of only 0.463 Å (46.3 pm), illustrating the rigidity of the transition state and confirming that this reaction step is best described by motion of the hydrogen atom only, explaining the large tunneling probability due to its low mass.

**Figure 6:**
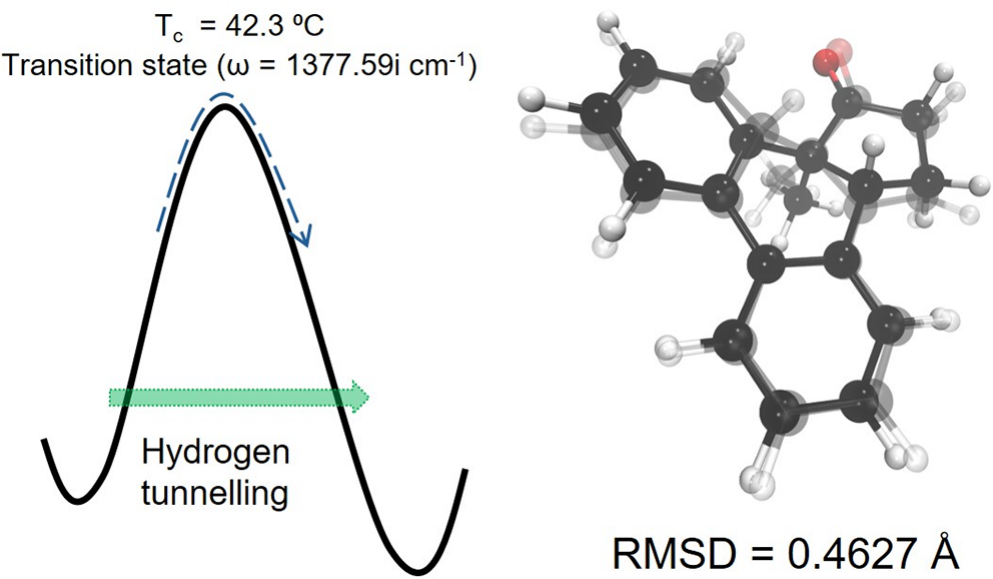
Illustration for the hydrogen tunneling through the wide barrier as measured by the large imaginary frequency and the resulting high crossover temperature (left). The right-hand side shows an overlay of the two structures before and after the 1,5-hydrogen shift, along with the calculated root-mean-square deviation excluding the shifted hydrogen atom.

For the deuterated species **15a**-*d*_5_, we calculated a crossover temperature of −27.5 °C from an imaginary frequency of 1072.61 cm^–1^, which is considerably lower than that of the undeuterated species. This crossover temperature indicates that the deuterated species is also in the tunneling regime at the experimental conditions. However, the significantly lowered transmission probability leaves enough time for competing deactivation processes, rendering the 1,5-migration inefficient.

## Conclusion

In summary, we have identified α-methyl-substituted α,β-unsaturated enones as a new substrate class to be involved in enantioselective, oxazaborolidine-catalyzed photochemical reactions. In the present study, cyclopent-2-enones **9** with a 1,1’-biphenyl substituent attached via carbon atom C2 to the enone β-carbon atom were employed as substrates. They delivered in a [6π] photocyclization 2,3,3a,11b-tetrahydro-1*H*-cyclopenta­[*l*]­phenanthren-1-ones **11** with high control of absolute configuration. The low catalyst loading of 2.5 to 5.0 mol % is testimony to the efficient activation of the substrate by the Lewis acid enabling irradiation at a wavelength regime at which the noncoordinated substrate is transparent. The binding mode of the substrate to the catalyst results in a differentiation of the enantiotopic faces relative to the reactive enone double bond, and it is conceivable that other photochemical transformations of related substrates can be controlled in a similar fashion. The use of Lewis acid-activated oxazaborolidines for another class of photochemical reactions beyond [2 + 2] photocycloadditions,^22,24a,30^ photoredox processes,^24b,24c^ and oxadi-π-methane rearrangements^25^ underpins their enormous versatility.

## Supplementary Material



## Data Availability

The data that supports the findings of this study are available in the Supporting Information of this article. Primary research data are openly available in the Chemotion Repository at DOI: 10.14272/collection/DGR_2025-10-09.

## Notes

Experimental details, materials, and methods; analytical data and NMR spectra for all new compounds; details and supplementary results of the quantum chemical calculations (PDF)
