# Supplementary material for: Catalytic Enantioselective [6π] Photocyclization Reactions by Chromophore Activation with a Chiral Lewis Acid
Source: J Am Chem Soc. 2025 Dec 5;147(50):46525–34. doi: 10.1021/jacs.5c17390 (PMC12715800; doi:10.1021/jacs.5c17390)
Supplement: Supplementary file 1 [file ja5c17390_si_001.pdf]

Supporting Information for:

Catalytic Enantioselective [6π] Photocyclization Reactions by Chromophore Activation with a Chiral Lewis Acid

Dominik Grünwald,[a] Chithra M. Jayakumari,[a] Noah Jeremias,[a] Julian Zuber,[a]

Christopher J. Stein,[a,b*] and Thorsten Bach[a*]

[a] *Department Chemie and Catalysis Research Center (CRC),*

School of Natural Sciences

Technische Universität München,

D-85747 Garching, Germany

[b] *Atomistic Modelling Center,*

Munich Data Science Institute,

Technische Universität München,

D-85748 Garching, Germany

thorsten.bach@ch.tum.de

Primary research data for the experiments described herein is openly available in the Chemotion Repository, accessible under DOI: 10.14272/collection/DGR_2025-10-09

Table of Contents

General Information S-2

Analytical Methods S-3

Emission Spectra of Light Sources S-4

Experimental Details S-9

General Procedures S-9

Synthesis of Chiral Lewis Acids S-11

Synthesis of Bis-Aryl Cyclopentenones..S-15

Photochemical Reactions S-29

Synthesis of Esters S-55

Determination of Absolute Configuration by Mosher Ester Analysis S-59

Determination of Absolute Configuration by SC-XRD S-61

Screening of Chiral Lewis Acids and Reaction Conditions S-62

Deuterium Labeling Experiments S-63

Chiral HPLC Traces S-65

NMR Spectra of New Compounds..S-89

Details of DFT Calculations...S-140

Free Energy Calculations - Methods S-140

Free Energy Calculations - Results S-141

Calculation of Crossover Temperature...S-143

Cartesian Coordinates of all Equilibrium Structures...S-144

References S-169

SC-XRD Structure Report for Compound 14..S-169

General Information

All air- and moisture-sensitive reactions were carried out in flame-dried glassware under an argon atmosphere using standard *Schlenk* techniques.

Unless otherwise noted, commercial chemicals were used without further purification. Dichloromethane (CH_2Cl_2) and tetrahydrofuran (THF) were dried using a *MBraun* MBSPS 800 solvent purification system. The following columns were used:

THF:	2 × MB-KOL-M type 2 (3 Å molecular sieves)
CH_2Cl_2:	2 × MB-KOL-A type 2 (aluminum oxide)

For the preparation of 1,3,2-oxazaborolidines, commercially available dry toluene was used (*Acros Organics,* 99.8% extra dry, over molecular sieves). Dibromomethane (CH_2Br_2) was degassed by four freeze-pump-thaw cycles and then dried by storing over 4 Å molecular sieves for at least 48 h. Stock solutions of aluminum bromide ($AlBr_3$) were prepared from anhydrous $AlBr_3$ and dry, degassed CH_2Br_2 at a concentration of 0.1 mM and stored at 0 °C under argon. To ensure reproducibility, stock solutions were prepared freshly every other week.

For photochemical reactions, dry CH_2Cl_2 was degassed using four freeze-pump-thaw cycles and stored over 4 Å molecular sieves.

Technical grade solvents including hexanes (Hex), ethyl acetate (EtOAc), dichloromethane (CH_2Cl_2) and methanol (MeOH) were used for column chromatography after simple distillation. Flash column chromatography was performed on silica 60 *(Merck,* 230-400 mesh) using the indicated eluent mixtures (v/v). Automated column chromatography was performed on a *Büchi* Pure C-815 or *Biotage* Selekt chromatography system using *Biotage* Sfär Duo column cartridges.

Racemic photoreactions were carried out in flame dried *Duran* tubes and irradiated inside a cylindrical array of 16 fluorescent UV lamps (8 W each). Enantioselective photoreactions were performed in flame dried *Duran Schlenk*-tubes in a negative geometry setup. For irradiation, light from the LED is transmitted through a partially ground glass rod with the ground portion submerged into the solution. A detailed description of the setup including pictures can be found in previous publications by our group.[1] 10 W LEDs with absorption maxima of 405, 398 and 368 nm were used, driven at a constant current of 700 mA. For reactions at -80 °C, the reaction flask was submerged in a *Dewar* flask filled with ethanol and cooled using a *Huber* TC100E cryocooler.

Analytical Methods

Thin Layer Chromatography (TLC) was performed on silica coated glass plates (*Merck* TLC silica gel 60 F_{254}) using the specified eluent mixtures (v/v). Spots were visualized under UV light (λ = 254 nm) and/or by staining in a potassium permanganate ($KMnO_4$) solution followed by heat treatment.

Melting points (M.p.) were determined using a *Kofler* heating bar designed by *Ludwig Kofler (Reichert)* without correction.

Nuclear Magnetic Resonance (NMR) spectra were recorded on *Bruker* AVHD-300, AVHD-400, NEO-400, AVHD-500 or AV-500 cryo spectrometers at room temperature. ^{1}H chemical shifts were referenced to the residual proton signal of chloroform-d_1 (δ = 7.26 ppm), dimethylsulfoxide-d_6 (δ = 2.50 ppm) or methanol-d_4 (δ = 3.31 ppm). ^{13}C spectra were referenced to the signal of chloroform-d_1 (δ = 77.16 ppm), dimethylsulfoxide-d_6 (δ = 128.06 ppm) or methanol-d_4 (δ = 49.00 ppm). ^{19}F spectra were not referenced. Assignment and multiplicity of ^{13}C signals was derived from 2D NMR experiments (COSY, HMBC, HSQC). Apparent multiplets of ^{1}H signals that result from equal or similar coupling constants to magnetically non-equivalent protons are marked as virtual (*virt.*). The following abbreviations for single multiplicities are used: *br* – broad, s – singlet, d – doublet, t – triplet, q – quartet, m – multiplet.

Infrared Spectra (IR) were recorded on an *Agilent* Cary 630 FTIR spectrometer by ATR technique. The following abbreviations are used to describe signal intensity: vs – very strong, s – strong, m – medium, w – weak.

High Resolution Mass Spectra (HRMS) were recorded on a *Thermo Scientific* LTQ-FT Ultra for ESI and a *Thermo Scientific* DFS-HRMS for EI.

Chiral High Performance Liquid Chromatography (HPLC) was performed on a *Thermo Scientific* Ultimate 3000 HPLC using the specified chiral stationary phase, eluent gradient, flow rate, temperature and detection wavelength.

UV/VIS spectra were recorded on a *PerkinElmer* 365+ using *Hellma* quartz cells with 1 mm path length.

Emission Spectra of Light Sources

350 nm fluorescent:

Lehrstuhl OC 1 - TUM

200 nm | 250 nm | 300 nm | 350 nm | 400 nm | 450 nm | 500 nm | 550 nm | 600 nm | 650 nm

Datasheet FLT021 LZC-UVA

Basic Information

Type	Fluorescent light tube
Description	Luzchem LZC-UVA
Manufacturer / Supplier	Hitachi / Luzchem
Order number / Date of purch.	LZC-UVA / 09/2015
Internal lot / serial number	2015-09 / FLT021

Specification Manufacturer

Type / size	T5 tube, G5 socket
Mechanical specification	16 mm diameter, 288 mm length
Electrical specification	8 W
Wavelength (range, typ.)	300 - 400 nm, 350m nm, UV-A
Spectral width (FWHM)	~ 40 nm
Datasheet	

Characterization

Description of measurement	Measured with Ocean-optics USB4000 spectrometer using a calibrated setup (cosine corrector/fibre). The cosine corrector was placed at 20 mm distance from a single fluorescent tube at half height.	
Measured dominant wavelength / Int.	350 nm	115 µW/mm²nm
Measured spectral width (FWHM)	40 nm	
Integral Reference intensity / range	5017 µW/cm²	300-425 nm

Spectrum

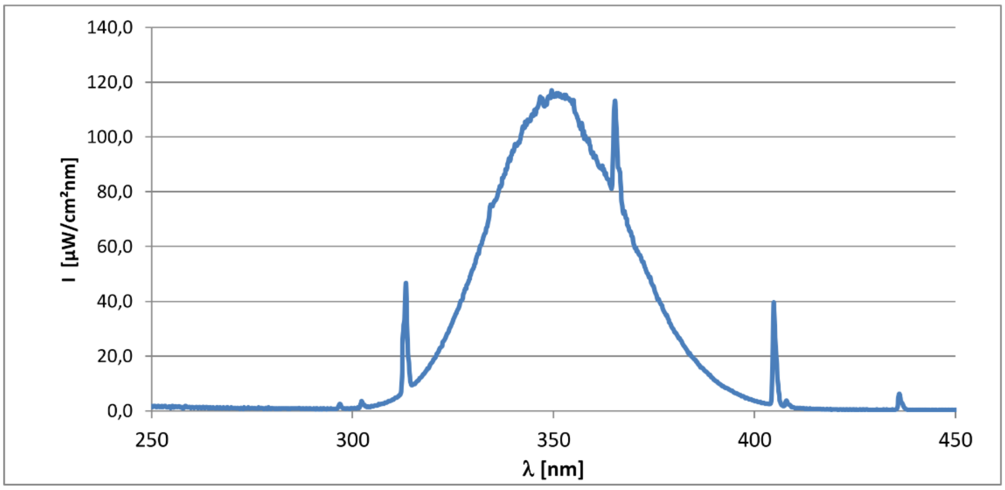

366 nm fluorescent:

Lehrstuhl OC 1 - TUM

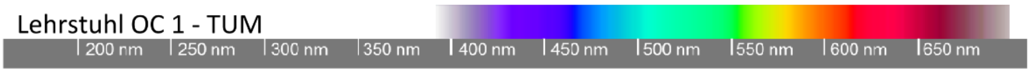

Datasheet FLT031_365 Philipps-BLB-365

Basic Information

Type	Fluorescent light tube	
Description	Philipps TL 8W BLB	
Manufacturer / Supplier	Philipps / multi-lite	
Order number / Date of purch.	n/a / 02/2024	
Internal lot / serial number	2024-02 / FLT030	

Specification Manufacturer

Type / size	T5 tube, G5 socket	
Mechanical specification	16 mm diameter, 288 mm length	
Electrical specification	8 W	
Wavelength (range, typ.)	350 - 400 nm	
Spectral width (FWHM)	~ 16 nm	
Datasheet Measurement	13.02.2024	

Characterization

Description of measurement	Measured with Ocean-optics USB4000 spectrometer using a calibrated setup (cosine corrector/fibre). The cosine corrector was placed at 20 mm distance from a single fluorescent tube at half height.	
Measured dominant wavelength / Int.	365 nm	183 µW/mm²nm
Measured spectral width (FWHM)	17 nm	
Integral Reference intensity / range	3594 µW/cm²	300-450 nm

Spectrum

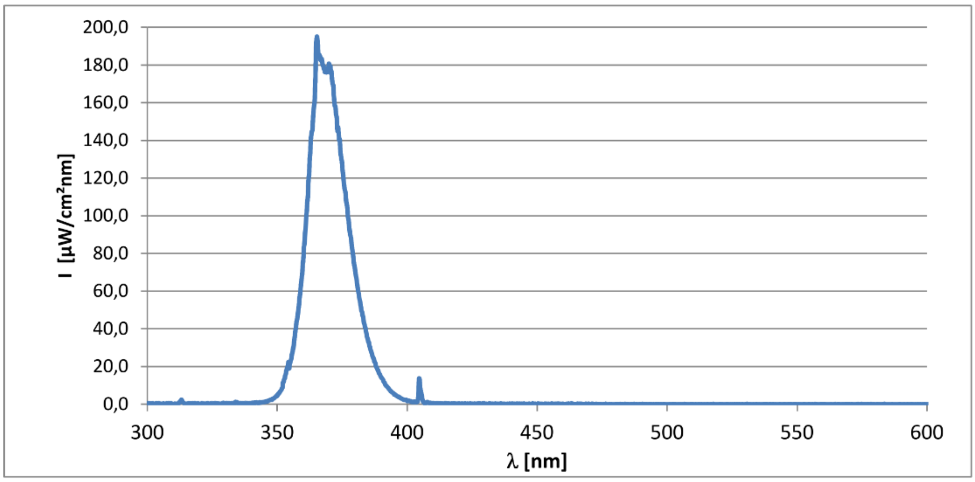

368 nm LED:

Lehrstuhl OC 1 - TUM

200 nm | 250 nm | 300 nm | 350 nm | 400 nm | 450 nm | 500 nm | 550 nm | 600 nm | 650 nm

#VALUE! 366 / 10W

Basic Information

Type	Ultra-High-Power UV-A-LED
Description	High Power LEDs - Single Colour 365nm LZ4 EMITTER
Manufacturer / Supplier	LED Engin / Mouser
Order number / Date of purch.	LZ4-44UV00-0000 / 06/2016
Internal lot / serial number	2016-01 / LED040

Specification Manufacturer

Type / size	quattro emitter / not spec.
Mechanical specification	
Electrical specification	700 mA @ 18 V
Wavelength (range, typ.)	366 nm
Spectral width (FWHM)	n/a
Datasheet	LZ4-04UV-series

Characterization

Description of measurement	Measured with Ocean-optics USB4000 spectrometer using a calibrated setup (cosine corrector/fibre). The distance between the emitting surface and the surface of the cosine corrector was 20 mm. The LED was operated at 700 mA on a passive heat-sink at approx. 20 °C	
Measured dominant wavelength / Int.	370 nm	14090 µW/mm²nm
Measured spectral width (FWHM)	13 nm	
Integral Reference intensity / range	211111 µW/cm²	350-425 nm

Spectrum

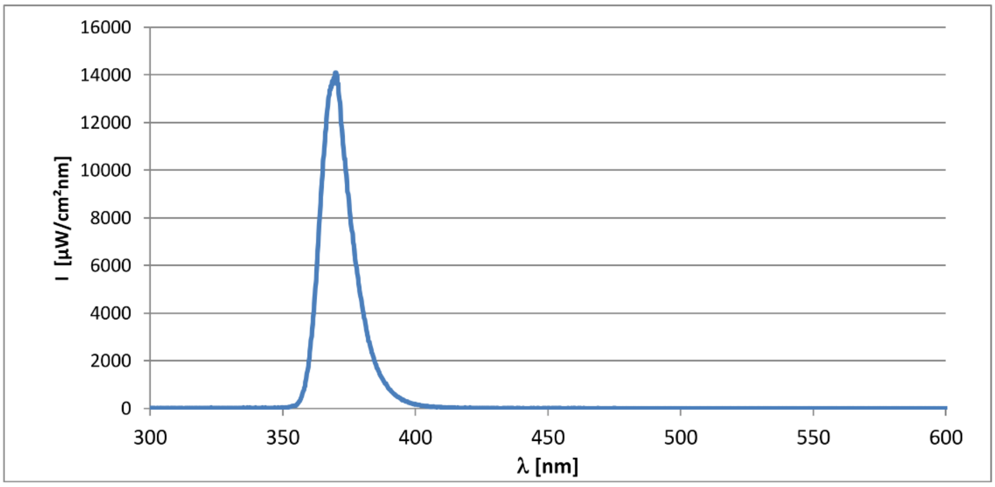

398 nm LED:

Lehrstuhl OC 1 - TUM

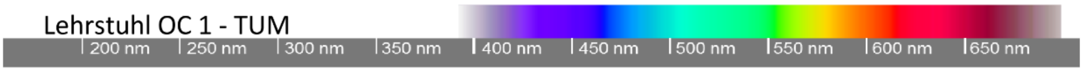

Datasheet LED026 400 / 10 W

Basic Information

	Ultra-High-Power Violet (400)
Type	High-Power-LED
Description	
Manufacturer / Supplier	LED-Engine Mouser
Order number / Date of purch.	LZ4-40UA00-00U6 / 01/2016
Internal lot / serial number	2016-01 / LED026

Specification Manufacturer

Type / size	quattro emitter / not spec.
Mechanical specification	
Electrical specification	700 mA @15 V
Wavelength (range, typ.)	
Spectral width (FWHM)	
Datasheet	LZ4-00UA-series.pdf

Characterization

Description of measurement	Measured with Ocean-optics USB4000 spectrometer using a calibrated setup (cosine corrector/fibre). The distance between the emitting surface and the surface of the cosine corrector was 20 mm. The LED was operated at 500 mA on a passive heat-sink at approx. 20 °C
Measured wavelength	398 nm
Measured spectral width	15 nm
Integral Reference intensity	317017 µW/cm² (360-480 nm @ 20 mm distance, 4 mmcosine corr.)

Spectrum

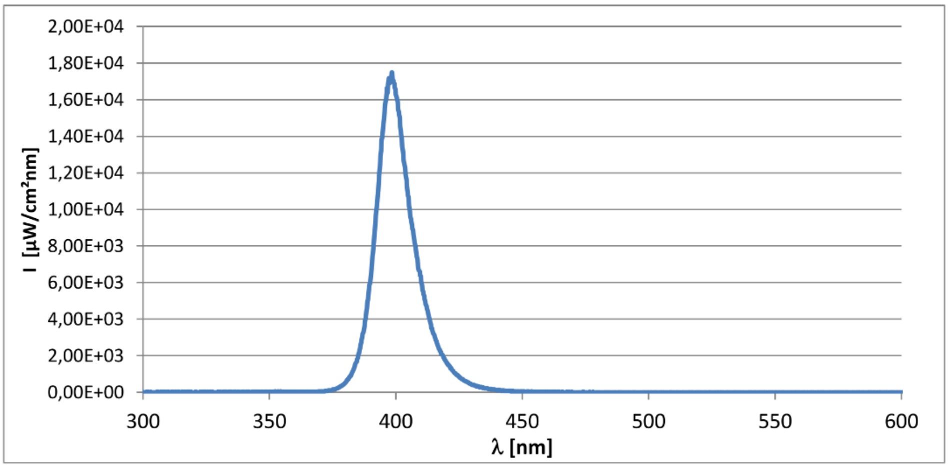

405 nm LED:

Lehrstuhl OC 1 - TUM

200 nm 250 nm 300 nm 350 nm 400 nm 450 nm 500 nm 550 nm 600 nm 650 nm

Datasheet LED074 — 405 nm / 10 W

Basic Information

Type	High-Power-LED	
Description	Single Colour Ultraviolet 10 Watt 400nm - 405nm	
Manufacturer / Supplier	ams OSRAM / Mouser	
Order number / Date of purch.	LZ4-40UA00-00U7 / 03/2016	
Internal lot / serial number	2016-03 / LED074	

Specification Manufacturer

Type / size	single emitter / ca. 7 x 7 mm	
Mechanical specification		
Electrical specification	700 mA, UF 13.76 - 18.56 V	
Wavelength (range, typ.)	400 - 405 nm	
Spectral width (FWHM)	n/a	
Datasheet	n/a	

Characterization

Description of measurement	Measured with Ocean-optics USB4000 spectrometer using a calibrated setup (cosine corrector/fibre). The distance between the emitting surface and the surface of the cosine corrector was 20 mm. The LED was operated at 700 mA on an active heat sink at approx. 20 °C	
Measured dominant wavelength / Int.	405 nm	21326 µW/mm²nm
Measured spectral width (FWHM)	17 nm	
Integral Reference intensity / range	378857 µW/cm²	350-500 nm

Spectrum

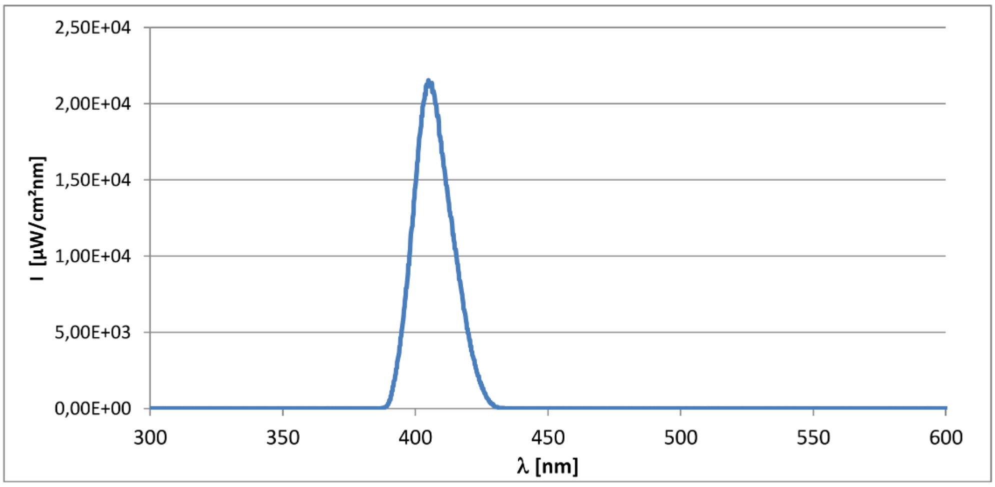

Experimental Details

General Procedures

GP A: Suzuki-Miyaura Coupling

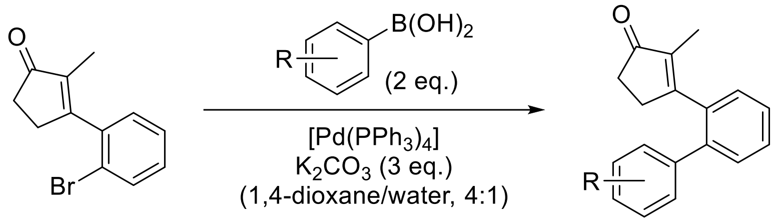

In analogy to a modified literature procedure[2], the respective iodide or bromide (1.00 eq.), the respective phenylboronic acid (2.00 eq.) and K_2CO_3 (3.00 eq.) were dissolved in a mixture of 1,4-dioxane and water (4:1). After degassing by bubbling argon through the solution in an ultrasonic bath for 15 minutes, $Pd(PPh_3)_4$ (3.00 or 5.00 mol%) was added and the mixture was heated to the respective temperature and stirred for the specified time. Once TLC indicated completion, the reaction mixture was allowed to cool to room temperature, diluted with EtOAc and filtered through a pad of silica topped with Celite. The crude product was concentrated *in vacuo* and purified by column chromatography using the specified eluent.

GP B: Enantioselective Photocyclization

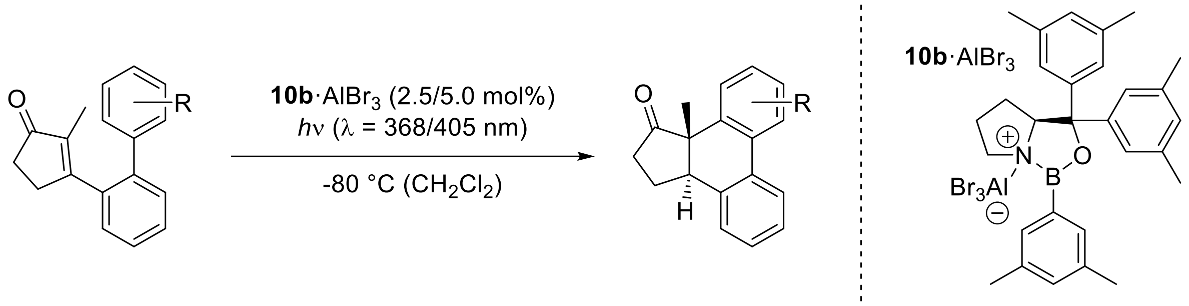

In a flame-dried *Schlenk* tube equipped with a ground glass rod[1], the starting material (50.0 µmol, 1.00 eq.) was dissolved in CH_2Cl_2 (final volume 5 mL, 10 mM) under an argon atmosphere. After cooling to -80 °C, 0.25 or 0.50 mL (1.25 µmol, 2.50 mol% or 2.50 µmol, 5.00 mol%, respectively) of the catalyst solution (*vide infra*) was added, and the reaction mixture was irradiated using an LED of the respective wavelength mounted to the ground glass rod. After TLC indicated consumption of the starting material, the Lewis acid was quenched by addition of NEt_3 (0.1 mL), the reaction mixture was allowed to warm to room temperature and concentrated *in vacuo*. The crude product was purified by automated column chromatography using the specified eluent.

GP C: Reduction of Ketone Cyclization Products

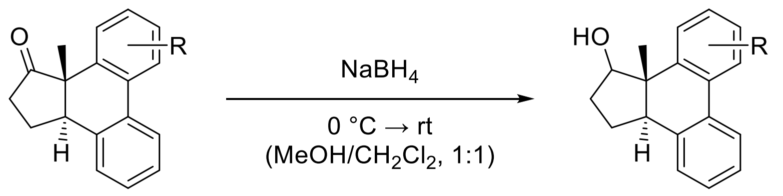

An excess of $NaBH_4$ (4-10 eq.) was added to a stirred solution of the respective ketone in a mixture of MeOH and CH_2Cl_2 (1:1) at 0 °C, after which the reaction mixture was allowed to warm to room temperature. Once TLC indicated consumption of the starting material, the reaction was quenched by addition of a saturated aqueous solution of $NaHCO_3$ (*bicarb*) at 0 °C and stirred until effervescence ceased. The phases were separated, and the aqueous layer was extracted three times with CH_2Cl_2. The combined organic phases were washed with brine, dried over Na_2SO_4 and concentrated *in vacuo*, followed by purification of the crude product via column chromatography using the specified eluent.

GP D: Synthesis of Prolinols

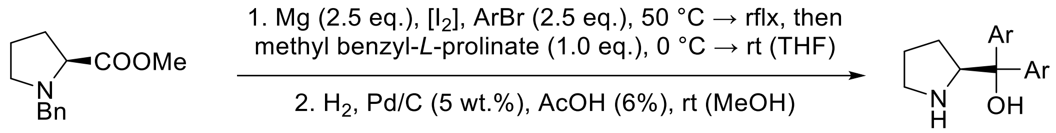

According to a modified literature procedure[3], a flask was charged with magnesium turnings (2.50 eq.), a small crystal of iodine, and THF (2.50 M). The suspension was heated to 50 °C and a few drops of the respective aryl bromide were added, upon which the fading of the brown color indicated the initiation of the *Grignard* reaction. Thus, the remaining aryl bromide (2.50 eq. in total) was added dropwise via syringe pump (0.1 mL/min), and the mixture was heated to reflux for 1 h. After cooling to 0 °C and addition of more THF as needed to prevent solidification, methyl benzyl-*L*-prolinate (**S1**) (1.00 eq.) was added dropwise. The mixture was allowed to warm up to room temperature and stirred until TLC indicated consumption of the ester. After cooling to 0 °C, excess *Grignard* reagent was quenched by addition of a saturated aqueous solution of NH_4Cl and the phases were separated. The aqueous layer was extracted three times with CH_2Cl_2 and the combined organic phases were washed with brine, dried over Na_2SO_4 and concentrated *in vacuo*. The crude product was purified by column chromatography before hydrogenolysis of the benzyl group.

The respective benzylated prolinol was dissolved in a mixture of acetic acid (6%) in methanol (125 mM). Before addition of 5 mol% palladium on carbon (varying loadings), the reaction flask was flushed with argon to prevent spontaneous combustion. A hydrogen atmosphere was then introduced by evacuating and backfilling the flask five times with hydrogen gas via

balloon, and the reaction was stirred until TLC indicated complete conversion. The catalyst was then removed by filtration over Celite and the filtrate was concentrated *in vacuo*. The residue was dissolved in equal volumes of 1M NaOH (aq.) and EtOAc, the phases were separated, and the aqueous layer was extracted three times with EtOAc. The combined organic layers were washed with brine, dried over Na_2SO_4 and concentrated *in vacuo*. The product was purified by column chromatography using the specified eluent.

Synthesis of Chiral Lewis Acids

Methyl benzyl-*L*-prolinate (S1)

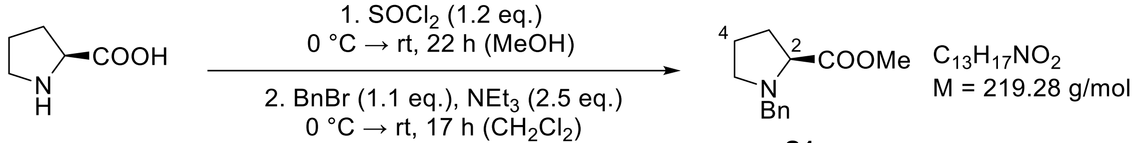

S1

According to a modified literature procedure[3], thionyl chloride (7.57 mL, 12.4 g, 104 mmol, 1.20 eq.) was added via syringe pump (0.3 mL/min) to a solution of *L*-proline (10.0 g, 86.9 mmol, 1.00 eq.) in 174 mL of MeOH at 0 °C. After stirring at room temperature for 22 h, the crude ester was concentrated *in vacuo* and then dissolved in 174 mL of CH_2Cl_2. Addition of NEt_3 (30.3 mL, 22.0 g, 217 mmol, 2.50 eq.) led to the formation of a colorless precipitate. Benzyl bromide (11.3 mL, 16.3 g, 95.6 mmol, 1.10 eq.) was then added dropwise to the suspension under strong stirring at 0 °C. After stirring at room temperature for 17 h, the reaction mixture was filtered, washed with small amounts of CH_2Cl_2, and concentrated *in vacuo*. Flash column chromatography (silica, Hex/EtOAc = 5:1) afforded **S1** (15.6 g, 70.9 mmol, 82%) as a yellow oil.

TLC: R_f = 0.23 (Hex/EtOAc = 6:1) [UV, $KMnO_4$].

^{1}H NMR (400 MHz, $CDCl_3$, 298 K): δ [ppm] = 7.36 – 7.18 (m, 5 H, H-Ph), 3.89 (d, ^2J = 12.8 Hz, 1 H, C*H*HPh), 3.65 (s, 3 H, OCH_3), 3.59 (d, ^2J = 12.8 Hz, 1 H, CH*H*Ph), 3.26 (dd, ^3J = 8.9 Hz, ^3J = 6.1 Hz, 1 H, H-2), 3.11 – 3.01 (m, 1 H, *H*H-5), 2.46 – 2.36 (m, 1 H, H*H*-5), 2.20 – 2.07 (m, 1 H, *H*H-3), 2.03 – 1.85 (m, 2 H, H*H*-3, *H*H-4), 1.83 – 1.72 (m, 1 H, H*H*-4).
^{13}C NMR (101 MHz, $CDCl_3$, 298 K): δ [ppm] = 174.6 (s, CO), 138.3 (s, C-Ph), 129.4 (d, 2 C, C-Ph), 128.3 (d, 2 C, C-Ph), 127.3 (d, C-Ph), 65.4 (d, C-2), 58.8 (t, CH_2Ph), 53.4 (t, C-5), 51.9 (q, OCH_3), 29.5 (t, C-3), 23.1 (t, C-4).

The recorded analytial data match the reported values.[3]

(*S*)-Diphenyl(pyrrolidin-2-yl)methanol (S2)

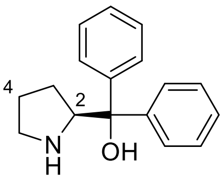

S2

$C_{17}H_{19}NO$

M = 253.35 g/mol

According to GP D, **S1** (500 mg, 2.28 mmol, 1.00 eq.) was reacted with magnesium (139 mg, 5.70 mmol, 2.50 eq.) and bromobenzene (0.60 mL, 0.90 g, 5.70 mmol, 2.50 eq.) in 10 mL of THF over 14 h. Following workup, purification by flash column chromatography (silica, Hex/EtOAc = 10:1) yielded the intermediate (535 mg, 1.56 mmol, 68%) as a colorless solid.

The material was subjected to hydrogenolysis in 12.5 mL MeOH and 0.75 mL AcOH with 5 wt.% Pd/C (100 mg) over 18 h. After workup, the crude product was purified by flash column chromatography (silica, CH_2Cl_2/MeOH = 10:1) to afford **S2** (365 mg, 1.41 mmol, 90%) as a colorless solid.

TLC: R_f = 0.23 (CH_2Cl_2/MeOH = 10:1) [UV, $KMnO_4$].

^{1}H NMR (400 MHz, MeOD, 298 K): δ [ppm] = 7.60 – 7.51 (m, 2 H, H-*o*-Ph), 7.50 – 7.42 (m, 2 H, H-*o*-Ph), 7.33 – 7.22 (m, 4 H, H-*m*-Ph), 7.20 – 7.11 (m, 2 H, H-*p*-Ph), 4.32 – 4.25 (m, 1 H, H-2), 3.03 – 2.92 (m, 1 H, *H*H-5), 2.91 – 2.79 (m, 1 H, H*H*-5), 1.79 – 1.67 (m, 3 H, *H*H-3, H-4), 1.67 – 1.54 (m, 1 H, H*H*-3).

^{13}C NMR (101 MHz, MeOD, 298 K): δ [ppm] = 148.4 (s, C-*i*-Ph), 147.6 (s, C-*i*-Ph), 129.2 (d, 2 C, C-*m*-Ph), 128.9 (d, 2 C, C-*m*-Ph), 127.6 (d, C-*p*-Ph), 127.5 (d, C-*p*-Ph), 127.4 (d, 2 C, C-*o*-Ph), 126.9 (d, 2 C, C-*o*-Ph), 79.7 (s, COH), 65.9 (d, C-2), 48.2 (t, C-5), 28.0 (t, C-3), 27.1 (t, C-4).

The recorded analytial data match the reported values.[3]

(*S*)-Bis(2,3-dimethylphenyl)(pyrrolidin-2-yl)methanol (S3)

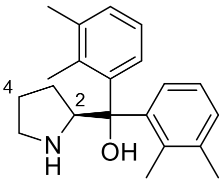

S3

$C_{21}H_{27}NO$

M = 309.45 g/mol

According to GP D, **S1** (500 mg, 2.28 mmol, 1.00 eq.) was reacted with magnesium (139 mg, 5.70 mmol, 2.50 eq.) and 1-bromo-2,3-dimethylbenzene (0.78 mL, 1.05 g, 5.70 mmol, 2.50 eq.) in 10 mL of THF over 15 h. Following workup, purification by automated column chromatography (silica, Hex/EtOAc = 100:0→80:20) yielded the intermediate (573 mg, 1.43 mmol, 63%) as a viscous yellow oil.

The material was subjected to hydrogenolysis in 11.5 mL MeOH and 0.7 mL AcOH with 5 wt.% Pd/C (116 mg) over 19 h. After workup, the crude product was purified by flash column chromatography (silica, CH_2Cl_2/MeOH/NH_3 = 20:1:0.1) to afford **S3** (201 mg, 648 μmol, 45%) as a glassy solid.

TLC: R_f = 0.51 (CH_2Cl_2/MeOH/NH_3 = 10:1:0.1) [UV, $KMnO_4$].

^{1}H NMR (500 MHz, MeOD, 298 K): δ [ppm] = 7.93 – 7.50 (m, 2 H, H-*o*-Ar), 7.12 – 6.99 (m, 4 H, H-*m*-Ar, H-*p*-Ar), 4.38 – 4.23 (m, 1 H, H-2), 3.04 – 2.83 (m, 2 H, H-5), 2.14 (d, ^4J = 3.0 Hz, 6 H, CH_3-*m*-Ar), 1.88 (s, 6 H, CH_3-*o*-Ar), 1.81 – 1.58 (m, 4 H, H-3, H-4).
^{13}C NMR (126 MHz, MeOD, 298 K): δ [ppm] = 145.0 (s, C-*i*-Ar), 144.5 (s, C-*i*-Ar), 139.6 (s, C-*m*-Ar), 138.8 (s, C-*m*-Ar), 129.9 (d, C-*p*-Ar), 129.6 (d, C-*p*-Ar), 127.5 (d, C-*o*-Ar), 127.1 (d, C-*o*-Ar), 125.5 (d, C-*m*-Ar), 125.2 (d, C-*m*-Ar), 80.9 (s, COH), 64.1 (d, C-2), 48.1 (t, C-5), 29.6 (t, C-3), 27.2 (t, C-4), 21.3 (q, CH_3-*m*-Ar), 21.3 (q, CH_3-*m*-Ar), 17.2 (q, CH_3-*o*-Ar), 17.2 (q, CH_3-*o*-Ar). Signals of (CH_3)*C*-*o*-Ar were not observable in the ^{13}C NMR spectrum.

The recorded analytial data match the reported values.[3]

(*S*)-Bis(3,5-dimethylphenyl)(pyrrolidin-2-yl)methanol (S4)

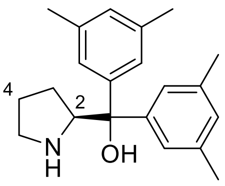

S4
$C_{21}H_{27}NO$
M = 309.45 g/mol

According to GP D, **S1** (1.00 g, 4.56 mmol, 1.00 eq.) was reacted with magnesium (277 mg, 11.4 mmol, 2.50 eq.) and 1-bromo-3,5-dimethylbenzene (1.55 mL, 2.11 g, 11.4 mmol, 2.50 eq.) in 10 mL of THF over 1.5 h. Following workup, purification by automated column chromatography (silica, Hex/EtOAc = 100:0→85:15) yielded the intermediate (1.33 g, 3.33 mmol, 73%) as a colorless solid.

The material was subjected to hydrogenolysis in 25 mL MeOH and 1.6 mL AcOH with 5 wt.% Pd/C (500 mg) over 21 h. After workup, the crude product was purified by automated column chromatography (silica, CH_2Cl_2/MeOH/NH_3 = 100:0:0→10:1:0.1) to afford **S4** (749 mg, 2.42 mmol, 73%) as a colorless solid.

TLC: R_f = 0.56 (CH_2Cl_2/MeOH/NH_3 = 10:1:0.1) [UV, $KMnO_4$].

^{1}H NMR (500 MHz, MeOD, 298 K): δ [ppm] = 7.17 – 7.15 (m, 2 H, H-*o*-Ar), 7.07 – 7.03 (m, 2 H, H-*o*-Ar), 6.83 – 6.81 (m, 1 H, H-*p*-Ar), 6.81 – 6.79 (m, 1 H, H-*p*-Ar), 4.26 – 4.22 (m, 1 H, H-2), 3.01 – 2.93 (m, 1 H, *H*H-5), 2.87 – 2.80 (m, 1 H, H*H*-5), 2.27 (s, 6 H, CH_3-Ar), 2.25 (s, 6 H, CH_3-Ar), 1.77 – 1.57 (m, 4 H, H-3, H-4).
^{13}C NMR (126 MHz, MeOD, 298 K): δ [ppm] = 148.1 (s, C-*i*-Ar), 147.5 (s, C-*i*-Ar), 138.7 (s, 2 C, C-*m*-Ar), 138.4 (s, 2 C, C-*m*-Ar), 129.1 (d, C-*p*-Ar), 128.9 (d, C-*p*-Ar), 125.1 (d, 2 C, C-*o*-Ar), 124.6 (d, 2 C, C-*o*-Ar), 79.6 (s, COH), 65.9 (d, C-2), 48.2 (t, C-5), 28.0 (t, C-3), 27.1 (t, C-4), 21.6 (q, 2 C, CH_3-Ar), 21.6 (q, 2 C, CH_3-Ar).

The recorded analytial data match the reported values.[3]

Representative Procedure: Preparation and Activation of 1,3,2-Oxazaborolidines

Due to their instability, 1,3,2-oxazaborolidines were freshly prepared ahead of each photoreaction and employed without further purification or characterization.

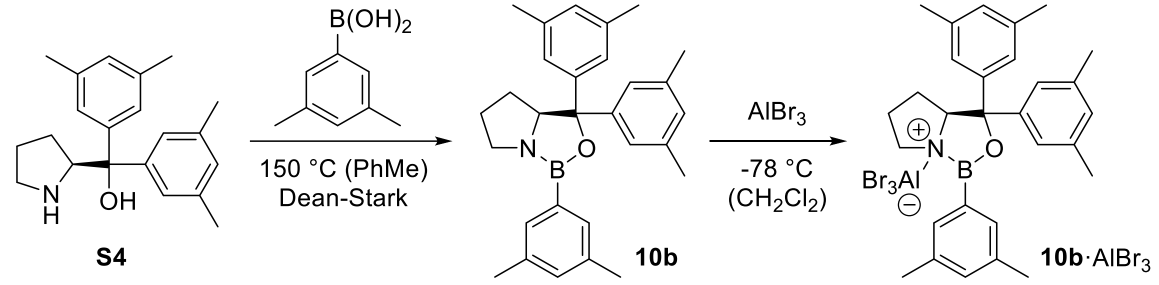

In analogy to a modified literature procedure[3], **S4** (12.4 mg, 40.0 µmol, 2.00 eq.) and (3,5-dimethylphenyl)boronic acid (6.00 mg, 40.0 µmol, 2.00 eq.) in 20 mL toluene were heated to reflux in a *Schlenk* flask fitted with a *Dean-Stark* trap (10 mL capacity) filled with toluene. After 3 h, the trap was drained and some distillate was collected (approx. 5 mL), which was subsequently drained again. The trap was then refilled with dry toluene and the procedure was repeated after another 3 h, before leaving the reaction to reflux over night. Toluene was then distilled off under a flow of argon and further removed *in vacuo*. The resulting 1,3,2-oxazaborolidine **10b** was dissolved in CH_2Cl_2 (3.8 mL) and cooled to -78 °C, after which a solution of $AlBr_3$ (0.2 mL, 0.1 M in CH_2Br_2 ≙ 20.0 µmol, 1.00 eq.) was added and stirring was continued for 10 minutes to yield a 5 mM solution of active catalyst **10b**·$AlBr_3$.

Synthesis of Bis-Aryl Cyclopentenones

3-Iodo-2-methylcyclopent-2-en-1-one (S5)

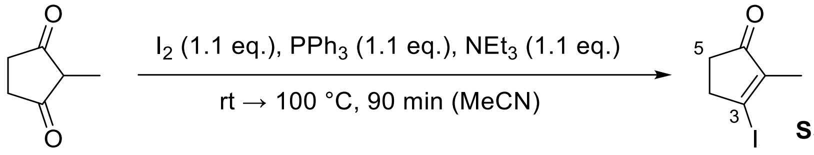

S5

C_6H_7IO
M = 222.03 g/mol

In analogy to a modified literature procedure[4], iodine (14.0 g, 55.0 mmol, 1.10 eq.) was added to a stirred solution of triphenylphosphine (14.4 g, 55.0 mmol, 1.10 eq.) in 250 mL of MeCN (0.20 M) at room temperature. After 30 minutes, triethylamine (7.62 mL, 9.98 g, 55.0 mmol, 1.10 eq.) was added and stirring continued for five minutes before addition of 2-methylcyclopentane-1,3-dione (5.61 g, 50.0 mmol, 1.00 eq.). After heating the reaction mixture to reflux for 90 minutes, the solvent was removed under reduced pressure, and the black residue was triturated with Et_2O (3 × 100 mL). The combined ether phases were filtered through a pad of silica and concentrated *in vacuo*. Flash column chromatography (silica, Hex/EtOAc = 2:1) afforded **S5** (9.98 g, 44.9 mmol, 90%) as yellow crystals.

TLC: R_f = 0.56 (Hex/EtOAc = 2:1) [UV, $KMnO_4$].

M.p.: 69-71 °C.

^{1}H NMR (500 MHz, $CDCl_3$, 298 K): δ [ppm] = 3.03 – 2.96 (m, 2 H, H-4), 2.55 – 2.50 (m, 2 H, H-5), 1.81 (t, ^5J = 2.2 Hz, 3 H, CH_3).
^{13}C NMR (101 MHz, $CDCl_3$, 298 K): δ [ppm] = 202.7 (s, C-1), 148.0 (s, C-2), 133.9 (s, C-3), 39.2 (t, C-4), 36.6 (t, C-5), 13.0 (q, CH_3).

The recorded analytial data match the reported values.[4]

3-(2-Bromophenyl)-2-methylcyclopent-2-en-1-one (S6)

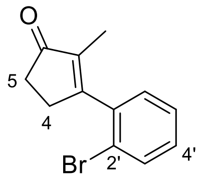

S6
$C_{12}H_{11}BrO$
M = 251.12 g/mol

According to GP A, **S5** (1.48 g, 6.64 mmol, 1.00 eq.) was reacted with (2-bromophenyl)boronic acid (1.33 g, 6.64 mmol, 1.00 eq.) in the presence of K_2CO_3 (2.75 g, 19.9 mmol, 3.00 eq.) and $Pd(PPh_3)_4$ (0.23 g, 0.20 mmol, 3.00 mol%) in 30 mL 1,4-dioxane/water (4:1) under reflux for 22 h. Flash column chromatography (silica, Hex/EtOAc = 4:1) afforded **S6** (1.37 g, 5.44 mmol, 82%) as a red-brown solid.

TLC: R_f = 0.45 (Hex/EtOAc = 4:1) [UV].

M.p.: 52 °C.

IR (ATR): $\tilde{\nu}$ [cm^{-1}] = 3056 (m, Ar-CH), 2945 (m, sp^3-CH), 2913 (m, sp^3-CH), 2842 (w, sp^3-CH), 1692 (vs, C=O), 1640 (s, C=C), 1338 (s, sp^3-CH), 1021 (s), 755 (vs, Ar-CH).

^{1}H NMR (500 MHz, $CDCl_3$, 298 K): δ [ppm] = 7.65 (dd, ^3J = 8.1 Hz, ^4J = 1.2 Hz, 1 H, H-6'), 7.38 (*virt.* td, $^3J \approx {}^3J$ = 7.5 Hz, ^4J = 1.2 Hz, 1 H, H-4'), 7.26 – 7.22 (m, 1 H, H-5'), 7.14 (dd, ^3J = 7.6 Hz, ^4J = 1.7 Hz, 1 H, H-3'), 2.88 – 2.81 (m, 2 H, H-4), 2.59 – 2.54 (m, 2 H, H-5), 1.63 (t, ^5J = 2.1 Hz, 3 H, CH_3).

^{13}C NMR (101 MHz, $CDCl_3$, 298 K): δ [ppm] = 209.7 (s, C-1), 168.9 (s, C-3), 139.5 (s, C-2), 138.6 (s, C-1'), 133.2 (d, C-3'), 129.9 (d, C-4'), 128.5 (d, C-6'), 127.6 (d, C-5'), 121.0 (s, C-2'), 34.5 (t, C-5), 31.0 (t, C-4), 9.1 (q, CH_3).

HRMS (ESI) m/z: calculated for $[M+H]^+$: 251.0066, found: 251.0061.

3-([1,1'-Biphenyl]-2-yl)-2-methylcyclopent-2-en-1-one (9a)

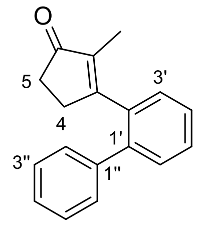

9a

$C_{18}H_{16}O$

M = 248.33 g/mol

According to GP A, **S6** (502 mg, 2.00 mmol, 1.00 eq.) was reacted with phenylboronic acid (488 mg, 4.00 mmol, 2.00 eq.) in the presence of K_2CO_3 (829 mg, 6.00 mmol, 3.00 eq.) and $Pd(PPh_3)_4$ (69.3 mg, 60.0 µmol, 3.00 mol%) in 20 mL 1,4-dioxane/water (4:1) at 70 °C for 48 h. Flash column chromatography (silica, Hex/EtOAc = 5:1) afforded **9a** (439 mg, 1.77 mmol, 88%) as a colorless solid.

TLC: R_f = 0.45 (Hex/EtOAc = 5:1) [UV].

M.p.: 94 °C.

^{1}H NMR (400 MHz, $CDCl_3$, 298 K): δ [ppm] = 7.49 – 7.39 (m, 3 H, H-4', H-5', H-6'), 7.37 – 7.29 (m, 3 H, H-3'', H-4'', H-5''), 7.26 – 7.22 (m, 3 H, H-2'', H-3', H-6''), 2.47 – 2.39 (m, 2 H, H-4), 2.36 – 2.30 (m, 2 H, H-5), 1.58 (t, ^5J = 2.0 Hz, 3 H, CH_3).

^{13}C NMR (126 MHz, $CDCl_3$, 298 K): δ [ppm] = 210.2 (s, C-1), 170.9 (s, C-3), 140.9 (s, C-1''), 140.4 (s, C-1'), 138.8 (s, C-2), 136.0 (s, C-2'), 130.5 (d, C-6'), 129.0 (d, C-5'), 128.6 (d, 2 C, C-3'', C-5''), 128.5 (d, 2 C, C-2'', C-6''), 128.1 (d, C-3'), 127.6 (d, C-4''), 127.4 (d, C-4'), 34.6 (t, C-5), 31.0 (t, C-4), 9.2 (q, CH_3).

The recorded analytial data match the reported values.[2]

3-([1,1'-Biphenyl]-2-yl-2',3',4',5',6'-d_5)-2-methylcyclopent-2-en-1-one (9a-d_5)

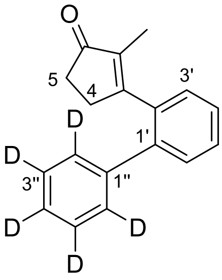

9a-d_5
$C_{18}H_{11}D_5O$
M = 253.36 g/mol

According to GP A, **S6** (174 mg, 0.69 mmol, 1.00 eq.) was reacted with (2,3,4,5,6-pentadeuteriophenyl)boronic acid (176 mg, 1.39 mmol, 2.00 eq.) in the presence of K_2CO_3 (287 mg, 2.08 mmol, 3.00 eq.) and $Pd(PPh_3)_4$ (40.0 mg, 34.6 µmol, 5.00 mol%) in 7 mL 1,4-dioxane/water (4:1) at 70 °C for 42 h. Flash column chromatography (silica, Hex/EtOAc = 5:1) afforded **9a**-d_5 (139 mg, 0.55 mmol, 79%) as a colorless solid.

TLC: R_f = 0.45 (Hex/EtOAc = 5:1) [UV].

M.p.: 100-105 °C.

^{1}H NMR (500 MHz, $CDCl_3$, 298 K): δ [ppm] = 7.50 – 7.37 (m, 3 H, H-4', H-5', H-6'), 7.26 – 7.23 (m, 1 H, H-3'), 2.45 – 2.41 (m, 2 H, H-4), 2.35 – 2.31 (m, 2 H, H-5), 1.58 (t, ^5J = 2.1 Hz, 3 H, CH_3).

^{13}C NMR (101 MHz, $CDCl_3$, 298 K): δ [ppm] = 210.1 (s, C-1), 170.9 (s, C-3), 140.8 (s, C-1''), 140.4 (s, C-1'), 138.8 (s, C-2), 136.0 (s, C-2'), 130.5 (d, C-6'), 129.0 (d, C-5'), 128.1 (d, C-3'), 127.4 (d, C-4'), 34.6 (t, C-5), 31.0 (t, C-4), 9.1 (q, CH_3).

Signals of deuterated carbon atoms were not observed in ^{13}C-NMR spectroscopy.

HRMS (EI, 70 eV): calculated for $C_{18}H_{11}D_5O$ $[M]^+$: 253.1510, found: 253.1508,
calculated for $C_{17}{}^{13}CH_{11}D_5O$ $[M]^+$: 254.1543, found: 254.1545.

2-Methyl-3-(4'-methyl-[1,1'-biphenyl]-2-yl)cyclopent-2-en-1-one (9b)

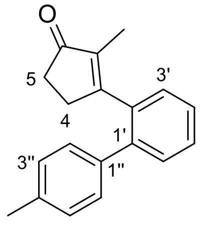

9b
$C_{19}H_{18}O$
M = 262.35 g/mol

According to GP A, **S6** (200 mg, 796 µmol, 1.00 eq.) was reacted with *p*-tolylboronic acid (217 mg, 1.59 mmol, 2.00 eq.) in the presence of K_2CO_3 (330 mg, 2.39 mmol, 3.00 eq.) and $Pd(PPh_3)_4$ (46.0 mg, 39.8 µmol, 5.00 mol%) in 8 mL 1,4-dioxane/water (4:1) at 70 °C for 16 h. Automated column chromatography (silica, Hex/EtOAc = 96:4→80:20) afforded **9b** (163 mg, 623 µmol, 78%) as a colorless solid.

TLC: R_f = 0.55 (Hex/EtOAc = 2:1) [UV].

M.p.: 90-95 °C.

IR (ATR): $\tilde{\nu}$ [cm^{-1}] = 3060 (w, Ar-CH), 3021 (w, Ar-CH), 2946 (m, sp^3-CH), 2907 (m, sp^3-CH), 1690 (vs, C=O), 1629 (s, C=C), 1336 (s), 826 (s, Ar-CH), 759 (vs, Ar-CH).

^{1}H NMR (400 MHz, $CDCl_3$, 298 K): δ [ppm] = 7.47 – 7.36 (m, 3 H, H-4', H-5', H-6'), 7.23 (d, ^{3}J = 7.2 Hz, 1 H, H-3'), 7.16 – 7.10 (m, 4 H, H-2'', H-3'', H-5'', H-6''), 2.49 – 2.42 (m, 2 H, H-4), 2.36 (s, 3 H, Ar-CH_3), 2.38 – 2.32 (m, 2 H, H-5), 1.57 (t, ^{5}J = 2.0 Hz, 3 H, CH_3).
^{13}C NMR (101 MHz, $CDCl_3$, 298 K): δ [ppm] = 210.1 (s, C-1), 171.1 (s, C-3), 140.4 (s, C-1'), 138.7 (s, C-2), 138.1 (s, C-1''), 137.4 (s, C-4''), 136.0 (s, C-2'), 130.5 (d, C-6'), 129.3 (d, 2 C, C-3'', C-5''), 128.9 (d, C-5'), 128.5 (d, 2 C, C-2'', C-6''), 128.1 (d, C-3'), 127.2 (d, C-4'), 34.6 (t, C-5), 31.0 (t, C-4), 21.3 (q, Ar-CH_3), 9.1 (q, CH_3).

HRMS (ESI) m/z: calculated for $[M+H]^+$: 263.1431, found: 263.1425.

3-(4'-Fluoro-[1,1'-biphenyl]-2-yl)-2-methylcyclopent-2-en-1-one (9c)

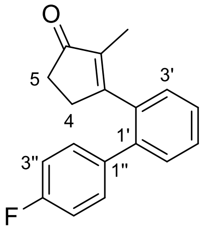

9c
$C_{18}H_{15}FO$
M = 266.32 g/mol

According to GP A, **S6** (251 mg, 1.00 mmol, 1.00 eq.) was reacted with (4-fluorophenyl)boronic acid (280 mg, 2.00 mmol, 2.00 eq.) in the presence of K_2CO_3 (415 mg, 3.00 mmol, 3.00 eq.) and $Pd(PPh_3)_4$ (34.7 mg, 30.0 µmol, 3.00 mol%) in 10 mL 1,4-dioxane/water (4:1) at 80 °C for 24 h. Flash column chromatography (silica, Hex/EtOAc = 4:1) afforded **9c** (245 mg, 920 µmol, 92%) as a colorless solid.

TLC: R_f = 0.35 (Hex/EtOAc = 4:1) [UV].

M.p.: 135 °C.

IR (ATR): $\tilde{\nu}$ [cm^{-1}] = 3062 (w, Ar-CH), 2952 (m, sp^3-CH), 2915 (m, sp^3-CH), 1677 (vs, C=O), 1633 (s, C=C), 1217 (vs, C-F), 837 (vs, Ar-CH), 751 (vs, Ar-CH).

^{1}H NMR (400 MHz, $CDCl_3$, 298 K): δ [ppm] = 7.49 – 7.38 (m, 3 H, H-4', H-5', H-6'), 7.26 – 7.18 (m, 3 H, H-2'', H-3', H-6''), 7.08 – 6.99 (m, 2 H, H-3'', H-5''), 2.48 – 2.39 (m, 2 H, H-4), 2.39 – 2.30 (m, 2 H, H-5), 1.57 (t, ^{5}J = 2.1 Hz, 3 H, CH_3).
^{13}C NMR (101 MHz, $CDCl_3$, 298 K): δ [ppm] = 209.8 (s, C-1), 170.4 (s, C-3), 162.4 (d, $^{1}J_{C\text{-}F}$ = 247.2 Hz, C-4''), 139.3 (s, C-1'), 139.0 (s, C-2), 137.0 (d, $^{4}J_{C\text{-}F}$ = 3.3 Hz, C-1''), 136.0 (s, C-2'), 130.5 (d, C-6')*, 130.2 (d, $^{3}J_{C\text{-}F}$ = 8.1 Hz, 2 C, C-2'', C-6''), 129.0 (d, C-5')*, 128.2 (d, C-3'), 127.6 (d, C-4')*, 115.6 (d, $^{2}J_{C\text{-}F}$ = 21.6 Hz, 2 C, C-3'', C-5''), 34.5 (t, C-5), 31.1 (t, C-4), 9.2 (q, CH_3).
*Assignments are interchangeable.
^{19}F NMR (376 MHz, $CDCl_3$, 298 K): δ [ppm] = -114.66.

HRMS (ESI) m/z: calculated for $[M+H]^+$: 267.1180, found: 267.1174.

2-Methyl-3-(4'-(trifluoromethyl)-[1,1'-biphenyl]-2-yl)cyclopent-2-en-1-one (9d)

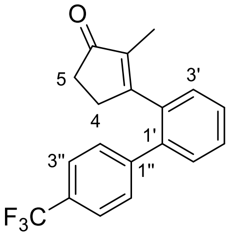

9d

$C_{19}H_{15}F_3O$
M = 316.32 g/mol

According to GP A, **S6** (200 mg, 796 µmol, 1.00 eq.) was reacted with (4-(trifluoromethyl)phenyl)boronic acid (303 mg, 1.59 mmol, 2.00 eq.) in the presence of K_2CO_3 (330 mg, 2.39 mmol, 3.00 eq.) and $Pd(PPh_3)_4$ (46.0 mg, 39.8 µmol, 5.00 mol%) in 8 mL 1,4-dioxane/water (4:1) at 70 °C for 16 h. Flash column chromatography (silica, Hex/EtOAc = 5:1) afforded **9d** (218 mg, 689 µmol, 87%) as a colorless solid.

TLC: R_f = 0.30 (Hex/EtOAc = 5:1) [UV].

M.p.: 142 °C.

IR (ATR): $\tilde{\nu}$ [cm^{-1}] = 2941 (m, sp^3-CH), 2917 (m, sp^3-CH), 1698 (vs, C=O), 1634 (s, C=C), 1616 (s, C=C), 1321 (vs, C-F), 1155 (vs, C-F), 1107 (vs, C-F), 1068 (vs, C-F), 841 (vs, Ar-CH), 759 (vs, Ar-CH).

^{1}H NMR (500 MHz, $CDCl_3$, 298 K): δ [ppm] = 7.63 – 7.58 (m, 2 H, H-3'', H-5''), 7.52 – 7.45 (m, 2 H, H-4', H-5'), 7.45 – 7.42 (m, 1 H, H-6'), 7.39 – 7.34 (m, 2 H, H-2'', H-6''), 7.30 – 7.27 (m, 1 H, H-3'), 2.50 – 2.43 (m, 2 H, H-4), 2.41 – 2.34 (m, 2 H, H-5), 1.55 (t, ^5J = 2.1 Hz, 3 H, CH_3).

^{13}C NMR (126 MHz, $CDCl_3$, 298 K): δ [ppm] = 209.7 (s, C-1), 169.7 (s, C-3), 144.6 (s, C-1''), 139.2 (s, C-2), 138.9 (s, C-1'), 136.0 (s, C-2'), 130.5 (d, C-6'), 129.8 (q, $^2J_{C\text{-}F}$ = 32.7 Hz, C-4''), 129.2 (d, C-5'), 129.0 (d, 2 C, C-2'', C-6''), 128.3 (d, C-3')*, 128.3 (d, C-4')*, 125.5 (q, $^3J_{C\text{-}F}$ = 3.6 Hz, 2 C, C-3'', C-5''), 124.2 (q, $^1J_{C\text{-}F}$ = 272.1 Hz, CF_3), 34.5 (t, C-5), 31.2 (t, C-4), 9.2 (q, CH_3).

*Assignments are interchangeable.

^{19}F NMR (376 MHz, $CDCl_3$, 298 K): δ [ppm] = -62.5.

HRMS (ESI) m/z: calculated for $[M+H]^+$: 317.1148, found: 317.1141.

3-(4'-(*Tert*-butyl)-[1,1'-biphenyl]-2-yl)-2-methylcyclopent-2-en-1-one (9e)

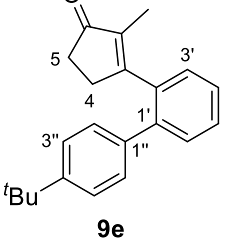

9e

$C_{22}H_{24}O$
M = 304.43 g/mol

According to GP A, **S6** (251 mg, 1.00 mmol, 1.00 eq.) was reacted with (4-(*tert*-butyl)phenyl)boronic acid (356 mg, 2.00 mmol, 2.00 eq.) in the presence of K_2CO_3 (415 mg, 3.00 mmol, 3.00 eq.) and $Pd(PPh_3)_4$ (34.7 mg, 30.0 µmol, 3.00 mol%) in 10 mL 1,4-dioxane/water (4:1) at 80 °C for 24 h. Flash column chromatography (silica, Hex/EtOAc = 4:1) afforded **9e** (249 mg, 817 µmol, 82%) as a colorless solid.

TLC: R_f = 0.50 (Hex/EtOAc = 4:1) [UV].

M.p.: 98-102 °C.

IR (ATR): $\tilde{\nu}$ [cm^{-1}] = 3060 (w, Ar-CH), 3027 (w, Ar-CH), 2960 (s, sp^3-CH), 2911 (m, sp^3-CH), 2864 (m, sp^3-CH), 1690 (vs, C=O), 1629 (s, C=C), 1336 (s), 839 (s, Ar-CH), 827 (s, Ar-CH), 766 (vs, Ar-CH), 755 (vs, Ar-CH).

^{1}H NMR (400 MHz, $CDCl_3$, 298 K): δ [ppm] = 7.47 – 7.37 (m, 3 H, H-4', H-5', H-6'), 7.37 – 7.33 (m, 2 H, H-3'', H-5''), 7.25 – 7.21 (m, 1 H, H-3'), 7.19 – 7.15 (m, 2 H, H-2'', H-6''), 2.47 – 2.41 (m, 2 H, H-4), 2.37 – 2.31 (m, 2 H, H-5), 1.57 (t, ^5J = 2.0 Hz, 3 H, CH_3), 1.33 (s, 9 H, $C(CH_3)_3$).

^{13}C NMR (101 MHz, $CDCl_3$, 298 K): δ [ppm] = 210.2 (s, C-1), 171.1 (s, C-3), 150.6 (s, C-4''), 140.4 (s, C-1'), 138.7 (s, C-2), 137.9 (s, C-1''), 136.0 (s, C-2'), 130.5 (d, C-6'), 128.9 (d, C-5'), 128.3 (d, 2 C, C-2'', C-6''), 128.1 (d, C-3'), 127.2 (d, C-4'), 125.4 (d, 2 C, C-3'', C-5''), 34.7 (s, $\mathit{C}(CH_3)_3$), 34.6 (t, C-5), 31.5 (q, 3 C, $C(\mathit{C}H_3)_3$), 31.0 (t, C-4), 9.1 (q, CH_3).

HRMS (ESI) m/z: calculated for $[M+H]^+$: 305.1900, found: 305.1894.

3-(4'-Chloro-[1,1'-biphenyl]-2-yl)-2-methylcyclopent-2-en-1-one (9f)

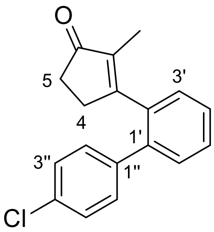

9f

$C_{18}H_{15}ClO$
M = 282.77 g/mol

According to GP A, **S6** (251 mg, 1.00 mmol, 1.00 eq.) was reacted with (4-chlorophenyl)boronic acid (313 mg, 2.00 mmol, 2.00 eq.) in the presence of K_2CO_3 (415 mg, 3.00 mmol, 3.00 eq.) and $Pd(PPh_3)_4$ (34.7 mg, 30.0 µmol, 3.00 mol%) in 10 mL 1,4-dioxane/water (4:1) at 80 °C for 15 h. Flash column chromatography (silica, Hex/EtOAc = 4:1) afforded **9f** (220 mg, 777 µmol, 78%) as a pale orange solid.

TLC: R_f = 0.28 (Hex/EtOAc = 4:1) [UV].

M.p.: 110-112 °C.

IR (ATR): $\tilde{\nu}$ [cm^{-1}] = 3056 (w, Ar-CH), 2914 (m, sp^3-CH), 1698 (vs, C=O), 1636 (s, C=C), 1087 (s), 837 (vs, Ar-CH), 759 (vs, Ar-CH).

^{1}H NMR (400 MHz, $CDCl_3$, 298 K): δ [ppm] = 7.47 – 7.39 (m, 3 H, H-4', H-5', H-6'), 7.34 – 7.29 (m, 2 H, H-2'', H-6''), 7.27 – 7.22 (m, 1 H, H-3'), 7.20 – 7.15 (m, 2 H, H-3'', H-5''), 2.49 – 2.43 (m, 2 H, H-4), 2.39 – 2.34 (m, 2 H, H-5), 1.57 (t, ^5J = 2.0 Hz, 3 H, CH_3).

^{13}C NMR (101 MHz, $CDCl_3$, 298 K): δ [ppm] = 209.7 (s, C-1), 170.1 (s, C-3), 139.4 (s, C-1'), 139.1 (s, C-1''), 139.1 (s, C-2), 136.0 (s, C-2'), 133.8 (s, C-4''), 130.4 (d, C-4'), 129.9 (d, 2 C, C-3'', C-5''), 129.1 (d, C-5'), 128.8 (d, 2 C, C-2'', C-6''), 128.2 (d, C-3'), 127.8 (d, C-6'), 34.5 (t, C-5), 31.1 (t, C-4), 9.2 (q, CH_3).

HRMS (ESI) m/z: calculated for $[M+H]^+$: 283.0884, found: 283.0877.

3-(3',5'-Dimethyl-[1,1'-biphenyl]-2-yl)-2-methylcyclopent-2-en-1-one (9g)

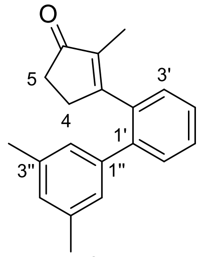

9g
$C_{20}H_{20}O$
M = 276.38 g/mol

According to GP A, **S6** (200 mg, 796 µmol, 1.00 eq.) was reacted with (3,5-dimethylphenyl)boronic acid (239 mg, 1.59 mmol, 2.00 eq.) in the presence of K_2CO_3 (330 mg, 2.39 mmol, 3.00 eq.) and $Pd(PPh_3)_4$ (46.0 mg, 39.8 µmol, 5.00 mol%) in 8 mL 1,4-dioxane/water (4:1) at 80 °C for 16 h. Flash column chromatography (silica, Hex/EtOAc = 5:1) afforded **9g** (201 mg, 727 µmol, 91%) as a colorless solid.

TLC: R_f = 0.28 (Hex/EtOAc = 5:1) [UV].

M.p.: 91 °C.

IR (ATR): $\tilde{\nu}$ [cm^{-1}] = 2915 (m, sp^3-CH), 2855 (w, sp^3-CH), 1690 (vs, C=O), 1634 (s, C=C), 854 (s, Ar-CH), 772 (vs, Ar-CH).

^{1}H NMR (400 MHz, $CDCl_3$, 298 K): δ [ppm] = 7.46 – 7.35 (m, 3 H, H-4', H-5', H-6'), 7.24 – 7.20 (m, 1 H, H-3'), 6.96 – 6.93 (m, 1 H, H-4''), 6.87 – 6.84 (m, 2 H, H-2'', H-6''), 2.47 – 2.39 (m, 2 H, H-4), 2.36 – 2.30 (m, 2 H, H-5), 2.29 (s, 6 H, Ar-CH_3), 1.61 (t, ^5J = 2.0 Hz, 3 H, CH_3).
^{13}C NMR (101 MHz, $CDCl_3$, 298 K): δ [ppm] = 210.0 (s, C-1), 171.2 (s, C-3), 140.9 (s, C-1''), 140.7 (s, C-1'), 138.5 (s, C-2), 137.9 (s, 2 C, C-3'', C-5''), 136.0 (s, C-2'), 130.4 (d, C-6'), 129.2 (d, C-4''), 128.8 (d, C-5'), 128.0 (d, C-3'), 127.2 (d, C-4'), 126.5 (d, 2 C, C-2'', C-6''), 34.6 (t, C-5), 31.1 (t, C-4), 21.4 (q, 2 C, Ar-CH_3), 9.2 (q, CH_3).

HRMS (ESI) m/z: calculated for $[M+H]^+$: 277.1587, found: 277.1581.

3-(3',5'-Difluoro-[1,1'-biphenyl]-2-yl)-2-methylcyclopent-2-en-1-one (9h)

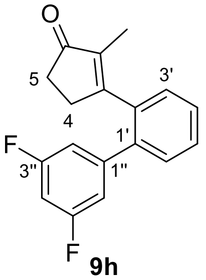

9h

$C_{18}H_{14}F_2O$
M = 284.31 g/mol

According to GP A, **S6** (200 mg, 796 µmol, 1.00 eq.) was reacted with (3,5-difluorophenyl)boronic acid (251 mg, 1.59 mmol, 2.00 eq.) in the presence of K_2CO_3 (330 mg, 2.39 mmol, 3.00 eq.) and $Pd(PPh_3)_4$ (46.0 mg, 39.8 µmol, 5.00 mol%) in 8 mL 1,4-dioxane/water (4:1) at 80 °C for 16 h. Flash column chromatography (silica, Hex/EtOAc = 3:1) afforded **9h** (193 mg, 645 µmol, 81%) as an off-white solid.

TLC: R_f = 0.38 (Hex/EtOAc = 3:1) [UV].

M.p.: 94 °C.

IR (ATR): $\tilde{\nu}$ [cm^{-1}] = 3097 (w, Ar-CH), 2915 (m, sp^3-CH), 1688 (s, C=O), 1620 (vs, C=C), 1592 (vs), 1338 (s, C-F), 1113 (vs, C-F), 988 (vs, C-F), 762 (s, Ar-CH).

^{1}H NMR (400 MHz, $CDCl_3$, 298 K): δ [ppm] = 7.50 – 7.44 (m, 2 H, H-4', H-5'), 7.44 – 7.39 (m, 1 H, H-6'), 7.30 – 7.22 (m, 1 H, H-3'), 6.82 – 6.73 (m, 3 H, H-2'', H-4'', H-6''), 2.54 – 2.48 (m, 2 H, H-4), 2.44 – 2.38 (m, 2 H, H-5), 1.58 (t, ^5J = 2.1 Hz, 3 H, CH_3).

^{13}C NMR (101 MHz, $CDCl_3$, 298 K): δ [ppm] = 209.5 (s, C-1), 169.3 (s, C-3), 162.8 (dd, $^1J_{C\text{-}F}$ = 249.4 Hz, $^3J_{C\text{-}F}$ = 12.9 Hz, 2 C, C-3'', C-5''), 144.1 (t, $^3J_{C\text{-}F}$ = 9.5 Hz, C-1''), 139.1 (s, C-2), 138.0 (t, $^4J_{C\text{-}F}$ = 2.4 Hz, C-1'), 135.8 (s, C-2'), 130.1 (d, C-6'), 129.1 (d, C-5'), 128.4 (d, C-4'), 128.2 (d, C-3'), 111.5 (m, 2 C, C-2'', C-6''), 103.0 (t, $^2J_{C\text{-}F}$ = 25.2 Hz, C-4''), 34.4 (t, C-5), 31.1 (t, C-4), 9.0 (q, CH_3).

^{19}F NMR (376 MHz, $CDCl_3$, 298 K): δ [ppm] = -109.22.

HRMS (ESI) m/z: calculated for $[M+H]^+$: 285.1086, found: 285.1079.

3-(3',5'-Dichloro-[1,1'-biphenyl]-2-yl)-2-methylcyclopent-2-en-1-one (9i)

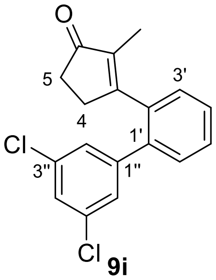

9i

$C_{18}H_{14}Cl_2O$
M = 317.21 g/mol

According to GP A, **S6** (200 mg, 796 µmol, 1.00 eq.) was reacted with (3,5-dichlorophenyl)boronic acid (303 mg, 1.59 mmol, 2.00 eq.) in the presence of K_2CO_3 (330 mg, 2.39 mmol, 3.00 eq.) and $Pd(PPh_3)_4$ (46.0 mg, 39.8 µmol, 5.00 mol%) in 8 mL 1,4-dioxane/water (4:1) at 80 °C for 17 h. Automated column chromatography (silica, Hex/EtOAc = 90:10→60:40) afforded **9i** (175 mg, 553 µmol, 69%) as a colorless solid.

TLC: R_f = 0.43 (Hex/EtOAc = 3:1) [UV].

M.p.: 128 °C.

IR (ATR): $\tilde{\nu}$ [cm^{-1}] = 3052 (m, Ar-CH), 2917 (m, sp^3-CH), 1694 (vs, C=O), 1638 (s, C=C), 1551 (s), 1340 (s), 798 (s, C-Cl), 759 (vs, Ar-CH).

^{1}H NMR (500 MHz, $CDCl_3$, 298 K): δ [ppm] = 7.49 – 7.44 (m, 2 H, H-4', H-5'), 7.42 – 7.38 (m, 1 H, H-6'), 7.32 (t, ^4J = 1.9 Hz, 1 H, H-4''), 7.28 – 7.23 (m, 1 H, H-3'), 7.14 (d, ^4J = 1.9 Hz, 2 H, H-2'', H-6''), 2.55 – 2.49 (m, 2 H, H-4), 2.44 – 2.38 (m, 2 H, H-5), 1.57 (t, ^5J = 2.0 Hz, 3 H, CH_3).

^{13}C NMR (101 MHz, $CDCl_3$, 298 K): δ [ppm] = 209.3 (s, C-1), 169.0 (s, C-3), 143.9 (s, C-1''), 139.4 (s, C-2), 137.6 (s, C-1'), 136.0 (s, C-2'), 135.1 (s, 2 C, C-3'', C-5''), 130.2 (d, C-6'), 129.2 (d, C-5'), 128.6 (d, C-4'), 128.3 (d, C-3'), 127.7 (d, C-4''), 127.1 (d, 2 C, C-2'', C-6''), 34.5 (t, C-5), 31.4 (t, C-4), 9.2 (q, CH_3).

HRMS (ESI) m/z: calculated for $[M+H]^+$: 317.0495, found: 317.0489.

3-(3',5'-Dimethoxy-[1,1'-biphenyl]-2-yl)-2-methylcyclopent-2-en-1-one (9j)

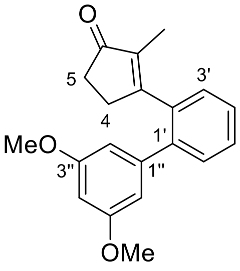

9j
$C_{20}H_{20}O_3$
M = 308.38 g/mol

According to GP A, **S6** (200 mg, 796 µmol, 1.00 eq.) was reacted with (3,5-dimethoxyphenyl)boronic acid (290 mg, 1.59 mmol, 2.00 eq.) in the presence of K_2CO_3 (330 mg, 2.39 mmol, 3.00 eq.) and $Pd(PPh_3)_4$ (46.0 mg, 39.8 µmol, 5.00 mol%) in 8 mL 1,4-dioxane/water (4:1) at 80 °C for 17 h. Automated column chromatography (silica, Hex/EtOAc = 98:2→60:40) afforded **9j** (196 mg, 637 µmol, 80%) as a yellow oil.

TLC: R_f = 0.34 (Hex/EtOAc = 4:1) [UV].

IR (ATR): $\tilde{\nu}$ [cm^{-1}] = 2981 (m, sp^3-CH), 2837 (m, sp^3-CH), 1694 (vs, C=O), 1588 (vs), 1202 (vs, C-O), 1152 (vs, C-O), 762 (s, Ar-CH).

^{1}H NMR (400 MHz, $CDCl_3$, 298 K): δ [ppm] = 7.49 – 7.38 (m, 3 H, H-4', H-5', H-6'), 7.25 – 7.19 (m, 1 H, H-3'), 6.43 – 6.38 (m, 3 H, H-2'', H-4'', H-6''), 3.74 (s, 6 H, OCH_3), 2.49 – 2.42 (m, 2 H, H-4), 2.38 – 2.32 (m, 2 H, H-5), 1.65 (t, ^5J = 2.0 Hz, 3 H, CH_3).

^{13}C NMR (101 MHz, $CDCl_3$, 298 K): δ [ppm] = 209.9 (s, C-1), 171.1 (s, C-3), 160.8 (s, 2 C, C-3'', C-5''), 142.9 (s, C-1''), 140.3 (s, C-1'), 138.7 (s, C-2), 136.0 (s, C-2'), 130.3 (d, C-6'), 128.9 (d, C-5'), 128.1 (d, C-3'), 127.6 (d, C-4'), 107.0 (d, 2 C, C-2'', C-6''), 99.6 (d, C-4''), 55.5 (q, OCH_3), 34.7 (t, C-5), 31.1 (t, C-4), 9.2 (q, CH_3).

HRMS (ESI) m/z: calculated for $[M+H]^+$: 309.1485, found: 309.1478.

2-Methyl-3-(3'-(trifluoromethyl)-[1,1'-biphenyl]-2-yl)cyclopent-2-en-1-one (9k)

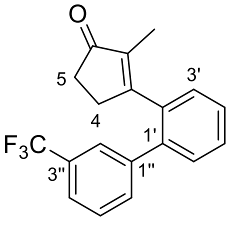

9k
$C_{19}H_{15}F_3O$
M = 316.32 g/mol

According to GP A, **S6** (200 mg, 796 µmol, 1.00 eq.) was reacted with (3-(trifluoromethyl)phenyl)boronic acid (303 mg, 1.59 mmol, 2.00 eq.) in the presence of K_2CO_3 (330 mg, 2.39 mmol, 3.00 eq.) and $Pd(PPh_3)_4$ (46.0 mg, 39.8 µmol, 5.00 mol%) in 8 mL 1,4-dioxane/water (4:1) at 80 °C for 16 h. Flash column chromatography (silica, Hex/EtOAc = 3:1) afforded **9k** (235 mg, 744 µmol, 93%) as a colorless solid.

TLC: R_f = 0.41 (Hex/EtOAc = 2:1) [UV].

M.p.: 64 °C.

IR (ATR): $\tilde{\nu}$ [cm^{-1}] = 3069 (m, Ar-CH), 2919 (m, sp^3-CH), 1683 (vs, C=O), 1633 (s, C=C), 1334 (vs), 1155 (vs, C-F), 1114 (vs, C-F), 1073 (vs, C-F), 759 (vs, Ar-CH), 701 (vs, Ar-CH).

^{1}H NMR (400 MHz, $CDCl_3$, 298 K): δ [ppm] = 7.60 – 7.56 (m, 1 H, H-4''), 7.56 – 7.52 (m, 1 H, H-2''), 7.52 – 7.40 (m, 5 H, H-4', H-5', H-5'', H-6', H-6''), 7.31 – 7.26 (m, 1 H, H-3'), 2.52 – 2.46 (m, 2 H, H-4), 2.38 – 2.32 (m, 2 H, H-5), 1.51 (t, ^5J = 2.1 Hz, 3 H, CH_3).

^{13}C NMR (126 MHz, $CDCl_3$, 298 K): δ [ppm] = 209.5 (s, C-1), 169.6 (s, C-3), 141.7 (s, C-2'), 139.3 (s, C-2), 138.8 (s, C-1'), 136.1 (s, C-1''), 131.8 (d, C-6''), 131.0 (q, $^2J_{C-F}$ = 32.3 Hz, C-3''), 130.4 (d, C-5'')*, 129.2 (d, C-5'), 129.0 (d, C-6')*, 128.2 (d, 2 C, C-3', C-4')*, 125.5 (q, $^3J_{C-F}$ = 3.8 Hz, C-2''), 124.3 (q, $^3J_{C-F}$ = 3.7 Hz, C-4''), 124.0 (q, $^1J_{C-F}$ = 272.3 Hz, CF_3), 34.4 (t, C-5), 31.3 (t, C-4), 9.1 (q, CH_3).

*Assignments are interchangeable.

^{19}F NMR (376 MHz, $CDCl_3$, 298 K): δ [ppm] = -62.8.

HRMS (ESI) m/z: calculated for $[M+H]^+$: 317.1148, found: 317.1141.

3-([1,1':3',1''-Terphenyl]-2-yl)-2-methylcyclopent-2-en-1-one (9l)

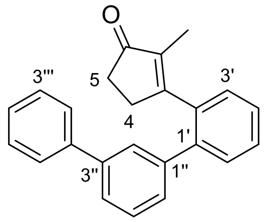

9l
$C_{24}H_{20}O$
M = 324.42 g/mol

According to GP A, **S6** (200 mg, 796 µmol, 1.00 eq.) was reacted with (3-phenylphenyl)boronic acid (315 mg, 1.59 mmol, 2.00 eq.) in the presence of K_2CO_3 (330 mg, 2.39 mmol, 3.00 eq.) and $Pd(PPh_3)_4$ (46.0 mg, 39.8 µmol, 5.00 mol%) in 8 mL 1,4-dioxane/water (4:1) at 80 °C for 6 h. Flash column chromatography (silica, Hex/EtOAc = 5:1) afforded **9l** (150 mg, 461 µmol, 58%) as a colorless solid.

TLC: R_f = 0.29 (Hex/EtOAc = 5:1) [UV].

M.p.: 114-116 °C.

IR (ATR): $\tilde{\nu}$ [cm^{-1}] = 3068 (m, Ar-CH), 3055 (m, Ar-CH), 3025 (m, Ar-CH), 2917 (m, sp^3-CH), 1681 (vs, C=O), 1631 (s, C=C), 1338 (s), 747 (vs, Ar-CH), 693 (vs, Ar-CH).

^{1}H NMR (400 MHz, $CDCl_3$, 298 K): δ [ppm] = 7.57 – 7.38 (m, 10 H, H-2'', H-2''', H-3''', H-4', H-4'', H-5', H-5'', H-5''', H-6', H-6'''), 7.38 – 7.32 (m, 1 H, H-4'''), 7.29 – 7.25 (m, 1 H, H-3'), 7.21 (*virt.* dt, ^3J = 7.6 Hz, $^4J \approx {}^4J$ = 1.5 Hz, 1 H, H-6''), 2.53 – 2.46 (m, 2 H, H-4), 2.39 – 2.32 (m, 2 H, H-5), 1.62 (t, ^5J = 2.0 Hz, 3 H, CH_3).
^{13}C NMR (126 MHz, $CDCl_3$, 298 K): δ [ppm] = 210.0 (s, C-1), 170.9 (s, C-3), 141.5 (s, C-3'')*, 141.4 (s, C-1'')*, 140.8 (s, C-1'''), 140.3 (s, C-1'), 138.9 (s, C-2), 136.1 (s, C-2'), 130.5 (d, C-6'), 129.0 (d, 3 C, C-3''', C-4', C-5'''), 129.0 (d, C-5''), 128.1 (d, C-3'), 127.7 (d, C-5')†, 127.6 (d, C-2'')†, 127.5 (d, C-4''')†, 127.5 (d, C-6'')†, 127.2 (d, 2 C, C-2''', C-6'''), 126.4 (d, C-4''), 34.6 (t, C-5), 31.1 (t, C-4), 9.2 (q, CH_3).
*,†Assignments are interchangeable.

HRMS (ESI) m/z: calculated for $[M+H]^+$: 325.1587, found: 325.1578.

2-Methyl-3-(3'-methyl-[1,1'-biphenyl]-2-yl)cyclopent-2-en-1-one (9m)

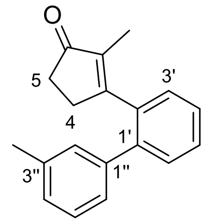

9m
$C_{19}H_{18}O$
M = 262.35 g/mol

According to GP A, **S6** (251 mg, 1.00 mmol, 1.00 eq.) was reacted with (3-methylphenyl)boronic acid (272 mg, 2.00 mmol, 2.00 eq.) in the presence of K_2CO_3 (415 mg, 3.00 mmol, 3.00 eq.) and $Pd(PPh_3)_4$ (34.7 mg, 30.0 µmol, 3.00 mol%) in 10 mL 1,4-dioxane/water (4:1) at 80 °C for 15 h. Flash column chromatography (silica, Hex/EtOAc = 4:1) afforded **9m** (255 mg, 971 µmol, 97%) as a brown solid.

TLC: R_f = 0.45 (Hex/EtOAc = 4:1) [UV].

M.p.: 92 °C.

IR (ATR): $\tilde{\nu}$ [cm^{-1}] = 3055 (m, Ar-CH), 2913 (m, sp^3-CH), 1687 (vs, C=O), 1631 (s, C=C), 1342 (s), 1092 (s), 770 (vs, Ar-CH), 704 (s, Ar-CH).

^{1}H NMR (400 MHz, $CDCl_3$, 298 K): δ [ppm] = 7.46 – 7.36 (m, 3 H, H-4', H-5', H-6'), 7.27 – 7.20 (m, 1 H, H-3'), 7.21 (*virt.* t, $^3J \approx {}^3J$ = 7.5 Hz, 1 H, H-5''), 7.12 (d, ^3J = 7.5 Hz, 1 H, H-4''), 7.08 (s, 1 H, H-2''), 7.01 (d, ^3J = 7.6 Hz, 1 H, H-6''), 2.46 – 2.40 (m, 2 H, H-4), 2.34 (s, 3 H, Ar-CH_3), 2.34 – 2.30 (m, 2 H, H-5), 1.59 (t, ^5J = 2.0 Hz, 3 H, CH_3).

^{13}**C NMR** (101 MHz, $CDCl_3$, 298 K): δ [ppm] = 210.1 (s, C-1), 171.1 (s, C-3), 140.9 (s, C-1''), 140.6 (s, C-1'), 138.7 (s, C-2), 138.1 (s, C-3''), 136.0 (s, C-2'), 130.5 (d, C-6'), 129.4 (d, C-2''), 128.9 (d, C-5'), 128.4 (d, C-5'')*, 128.3 (d, C-4'')*, 128.1 (d, C-3'), 127.3 (d, C-4'), 125.7 (d, C-6''), 34.6 (t, C-5), 31.0 (t, C-4), 21.6 (q, Ar-CH_3), 9.1 (q, CH_3).
*Assignments are interchangeable.

HRMS (ESI) m/z: calculated for $[M+H]^+$: 263.1431, found: 263.1425.

3-(3'-Fluoro-[1,1'-biphenyl]-2-yl)-2-methylcyclopent-2-en-1-one (9n)

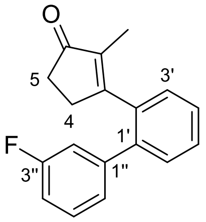

9n
$C_{18}H_{15}FO$
M = 266.32 g/mol

According to GP A, **S6** (200 mg, 796 µmol, 1.00 eq.) was reacted with (3-fluorophenyl)boronic acid (223 mg, 1.59 mmol, 2.00 eq.) in the presence of K_2CO_3 (330 mg, 2.39 mmol, 3.00 eq.) and $Pd(PPh_3)_4$ (46.0 mg, 39.8 µmol, 5.00 mol%) in 8 mL 1,4-dioxane/water (4:1) at 80 °C for 18 h. Flash column chromatography (silica, Hex/EtOAc = 4:1) afforded **9n** (178 mg, 667 µmol, 84%) as a colorless solid.

TLC: R_f = 0.34 (Hex/EtOAc = 4:1) [UV].

M.p.: 90 °C.

IR (ATR): $\tilde{\nu}$ [cm^{-1}] = 3066 (w, Ar-CH), 2919 (m, sp^3-CH), 1681 (vs, C=O), 1634 (s, C=C), 1584 (s), 1340 (s), 1180 (s, C-F), 878 (s, C-F), 759 (vs, Ar-CH), 695 (vs, Ar-CH).

^{1}**H NMR** (400 MHz, $CDCl_3$, 298 K): δ [ppm] = 7.50 – 7.40 (m, 3 H, H-4', H-5', H-6'), 7.33 – 7.22 (m, 2 H, H-3', H-5''), 7.05 – 6.94 (m, 3 H, H-2'', H-4'', H-6''), 2.50 – 2.43 (m, 2 H, H-4), 2.39 – 2.34 (m, 2 H, H-5), 1.57 (t, ^5J = 2.1 Hz, 3 H, CH_3).
^{13}**C NMR** (126 MHz, $CDCl_3$, 298 K): δ [ppm] = 209.9 (s, C-1), 170.1 (s, C-3), 162.7 (d, $^1J_{C\text{-}F}$ = 246.8 Hz, C-3''), 143.2 (d, $^3J_{C\text{-}F}$ = 7.6 Hz, C-1''), 139.1 (d, $^4J_{C\text{-}F}$ = 2.3 Hz, C-1'), 139.0 (s, C-2), 135.9 (s, C-2'), 130.4 (d, C-6'), 130.1 (d, $^3J_{C\text{-}F}$ = 8.5 Hz, C-5''), 129.1 (d, C-5'), 128.2 (d, C-3'), 128.0 (d, C-4'), 124.4 (d, $^4J_{C\text{-}F}$ = 3.0 Hz, C-6''), 115.6 (d, $^2J_{C\text{-}F}$ = 21.7 Hz, C-4''), 114.5 (d, $^2J_{C\text{-}F}$ = 20.9 Hz, C-2''), 34.5 (t, C-5), 31.1 (t, C-4), 9.2 (q, CH_3).
^{19}**F NMR** (376 MHz, $CDCl_3$, 298 K): δ [ppm] = -112.75.

HRMS (ESI) m/z: calculated for $[M+H]^+$: 267.1180, found: 267.1173.

3-(3'-Chloro-[1,1'-biphenyl]-2-yl)-2-methylcyclopent-2-en-1-one (9o)

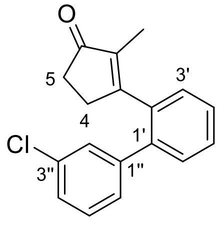

9o
$C_{18}H_{15}ClO$
M = 282.77 g/mol

According to GP A, **S6** (200 mg, 796 µmol, 1.00 eq.) was reacted with (3-chlorophenyl)boronic acid (249 mg, 1.59 mmol, 2.00 eq.) in the presence of K_2CO_3 (330 mg, 2.39 mmol, 3.00 eq.) and $Pd(PPh_3)_4$ (46.0 mg, 39.8 µmol, 5.00 mol%) in 8 mL 1,4-dioxane/water (4:1) at 80 °C for 18 h. Flash column chromatography (silica, Hex/EtOAc = 5:1) afforded **9o** (208 mg, 737 µmol, 93%) as a colorless solid.

TLC: R_f = 0.31 (Hex/EtOAc = 5:1) [UV].

M.p.: 64-66 °C.

IR (ATR): $\tilde{\nu}$ [cm^{-1}] = 3066 (w, Ar-CH), 2919 (m, sp^3-CH), 1679 (vs, C=O), 1631 (s, C=C), 1338 (s), 750 (vs, Ar-CH), 695 (vs, Ar-CH).

^{1}H NMR (400 MHz, $CDCl_3$, 298 K): δ [ppm] = 7.50 – 7.39 (m, 3 H, H-4', H-5', H-6'), 7.32 – 7.22 (m, 4 H, H-2'', H-3', H-4'', H-5''), 7.09 (*virt.* dt, ^3J = 7.3 Hz, $^4J \approx ^4J$ = 1.6 Hz, 1 H, H-6''), 2.50 – 2.43 (m, 2 H, H-4), 2.40 – 2.33 (m, 2 H, H-5), 1.57 (t, ^5J = 2.0 Hz, 3 H, CH_3).
^{13}C NMR (126 MHz, $CDCl_3$, 298 K): δ [ppm] = 209.8 (s, C-1), 170.0 (s, C-3), 142.7 (s, C-1''), 139.0 (s, C-2)*, 138.9 (s, C-1')*, 136.0 (s, C-2'), 134.4 (s, C-3''), 130.4 (d, C-6'), 129.7 (d, C-5'')†, 129.1 (d, C-5')†, 128.7 (d, C-2'')†, 128.2 (d, C-4')†, 128.0 (d, C-3')†, 127.7 (d, C-4'')†, 126.8 (d, C-6''), 34.5 (t, C-5), 31.2 (t, C-4), 9.2 (q, CH_3).
*,†Assignments are interchangeable.

HRMS (ESI) m/z: calculated for $[M+H]^+$: 283.0884, found: 283.0877.

3-(3'-Methoxy-[1,1'-biphenyl]-2-yl)-2-methylcyclopent-2-en-1-one (9p)

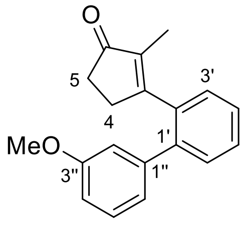

9p
$C_{19}H_{18}O_2$
M = 278.35 g/mol

According to GP A, **S6** (200 mg, 796 µmol, 1.00 eq.) was reacted with (3-methoxyphenyl)boronic acid (242 mg, 1.59 mmol, 2.00 eq.) in the presence of K_2CO_3 (330 mg, 2.39 mmol, 3.00 eq.) and $Pd(PPh_3)_4$ (46.0 mg, 39.8 µmol, 5.00 mol%) in 8 mL 1,4-dioxane/water (4:1) at 80 °C for 60 h. Flash column chromatography (silica, Hex/EtOAc = 5:1) afforded **9p** (113 mg, 405 µmol, 51%) as a colorless solid.

TLC: R_f = 0.34 (Hex/EtOAc = 5:1) [UV].

M.p.: 80 °C.

IR (ATR): $\tilde{\nu}$ [cm^{-1}] = 3058 (w, Ar-CH), 3001 (w, sp^3-CH), 2919 (m, sp^3-CH), 2835 (m, sp^3-CH), 1698 (vs, C=O), 1636 (m, C=C), 1597 (s), 1577 (s), 1210 (s, C-O), 759 (vs, Ar-CH), 697 (vs, Ar-CH).

^{1}H NMR (400 MHz, $CDCl_3$, 298 K): δ [ppm] = 7.47 – 7.39 (m, 3 H, H-4', H-5', H-6'), 7.27 – 7.20 (m, 2 H, H-3', H-5''), 6.86 (ddd, ^3J = 8.3 Hz, ^4J = 2.6 Hz, ^4J = 0.9 Hz, 1 H, H-6''), 6.82 (ddd, ^3J = 7.5 Hz, ^4J = 1.6 Hz, ^4J = 0.9 Hz, 1 H, H-4''), 6.80 (dd, ^4J = 2.6 Hz, ^4J = 1.6 Hz, 1 H, H-2''), 3.77 (s, 3 H, OCH_3), 2.47 – 2.41 (m, 2 H, H-4), 2.37 – 2.31 (m, 2 H, H-5), 1.61 (t, ^5J = 2.0 Hz, 3 H, CH_3).

^{13}C NMR (126 MHz, $CDCl_3$, 298 K): δ [ppm] = 210.1 (s, C-1), 171.1 (s, C-3), 159.5 (s, C-3''), 142.3 (s, C-1''), 140.2 (s, C-1'), 138.7 (s, C-2), 136.0 (s, C-2'), 130.4 (d, C-6'), 129.5 (d, C-5''), 128.9 (d, C-5'), 128.1 (d, C-3'), 127.5 (d, C-4'), 121.1 (d, C-6''), 114.4 (d, C-2''), 112.9 (d, C-4''), 55.3 (q, OCH_3), 34.6 (t, C-5), 31.0 (t, C-4), 9.2 (q, CH_3).

HRMS (ESI) m/z: calculated for $[M+H]^+$: 279.1380, found: 279.1373.

Photochemical Reactions

(3a*R*,11b*S*)-11b-Methyl-2,3,3a,11b-tetrahydro-1*H*-cyclopenta[*l*]phenanthren-1-one (11a)

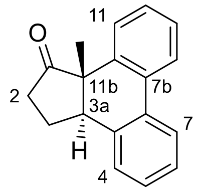

11a

$C_{18}H_{16}O$

M = 248.33 g/mol

According to GP B, **9a** (12.4 mg, 50.0 µmol, 1.00 eq.) was irradiated at λ = 405 nm for 3 h in the presence of **10b**·$AlBr_3$ (2.50 µmol, 5.00 mol%). Automated column chromatography (silica, Hex/EtOAc = 100:0→85:15) afforded **11a** (10.1 mg, 40.7 µmol, 81%, 96% *ee*) as a colorless solid.

TLC: R_f = 0.65 (Hex/EtOAc = 2:1) [UV].

M.p.: 91-93 °C.

^{1}H NMR (500 MHz, $CDCl_3$, 298 K): δ [ppm] = 8.30 – 8.27 (m, 1 H, H-11), 7.80 – 7.77 (m, 1 H, H-8), 7.77 – 7.74 (m, 1 H, H-7), 7.41 – 7.30 (m, 4 H, H-5, H-6, H-9, H-10), 7.28 – 7.23 (m, 1 H, H-4), 3.39 (dd, ^3J = 12.7 Hz, ^3J = 6.0 Hz, 1 H, H-3a), 2.77 (ddd, ^2J = 19.0 Hz, ^3J = 8.5 Hz, ^3J = 0.9 Hz, 1 H, *H*H-2), 2.56 (ddd, ^2J = 19.0 Hz, ^3J = 10.0 Hz, ^3J = 8.8 Hz, 1 H, H*H*-2), 2.45 (dddd, ^2J = 12.2 Hz, ^3J = 8.8 Hz, ^3J = 6.0 Hz, ^3J = 0.9 Hz, 1 H, *H*H-3), 2.13 (dddd, ^3J = 12.7 Hz, ^2J = 12.2 Hz, ^3J = 10.0 Hz, ^3J = 8.5 Hz, 1 H, H*H*-3), 0.89 (s, 3 H, CH_3).

^{13}C NMR (126 MHz, $CDCl_3$, 298 K): δ [ppm] = 217.0 (s, C-1), 141.2 (s, C-11a), 136.8 (s, C-3b), 135.1 (s, C-7a), 134.0 (s, C-7b), 128.1 (d, C-10)*, 128.0 (d, C-5)*, 127.4 (d, C-9), 127.3 (d, C-6), 125.1 (d, C-8), 124.9 (d, C-4), 124.5 (d, C-7), 123.9 (d, C-11), 48.2 (s, C-11b), 46.5 (d, C-3a), 38.1 (t, C-2), 20.1 (t, C-3), 17.3 (q, CH_3).

*Assignments are interchangeable.

The recorded analytial data match the reported values.[2]

HRMS (EI, 70 eV): calculated for $C_{18}H_{16}O$ $[M]^+$: 248.1196, found: 248.1195,
calculated for $C_{17}{}^{13}CH_{16}O$ $[M]^+$: 249.1229, found: 249.1228.

Chiral HPLC: 96% *ee* [Daicel Chiralcel, OD-RH, 150x4.6, H_2O/MeCN = 80:20→0:100 (30 min), 1 mL/min, 215 nm, t_R = 21.86 min (minor), 23.49 min (major)].

(3a*R*,11b*S*)-11b-Methyl-2,3,3a,11b-tetrahydro-1*H*-cyclopenta[*l*]phenanthren-1-one-3a,8,9,10,11-d_5 (11a-d_5) and (3a*R*,11b*S*)-11b-methyl-2,3,3a,11b-tetrahydro-1*H*-cyclopenta[*l*]phenanthren-1-one-8,9,10,11-d_4 (11a-d_4)

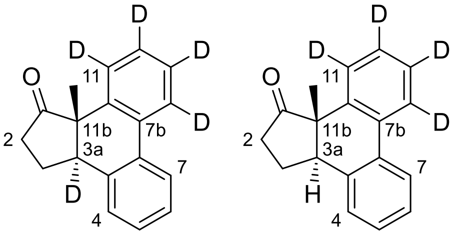

11a-d_5
$C_{18}H_{11}D_5O$
M = 253.36 g/mol

11a-d_4
$C_{18}H_{12}D_4O$
M = 252.35 g/mol

According to GP B, **9a**-d_5 (12.7 mg, 50.0 µmol, 1.00 eq.) was irradiated at λ = 405 nm for 1.5 h in the presence of **10b**·$AlBr_3$ (2.50 µmol, 5.00 mol%). Automated column chromatography (silica, Hex/EtOAc = 100:0→85:15) afforded a mixture of **11a**-d_5 and **11a**-d_4 (26/74, 0.8 mg, 3.16 µmol, 6%) as a colorless solid.

TLC: R_f = 0.65 (Hex/EtOAc = 2:1) [UV].

M.p.: 95-100 °C.

^{1}H NMR (500 MHz, $CDCl_3$, 298 K): δ [ppm] = 7.80 – 7.71 (m, 1 H, H-7), 7.40 – 7.33 (m, 2 H, H-5, H-6), 7.29 – 7.23 (m, 1 H, H-4), 3.39 (dd, ^3J = 12.6 Hz, ^3J = 5.9 Hz, 1 H, H-3a), 2.76 (ddd, ^2J = 19.1 Hz, ^3J = 8.6 Hz, ^3J = 1.1 Hz, 1 H, *H*H-2), 2.56 (dddd, ^2J = 19.1 Hz, ^3J = 10.1 Hz, ^3J = 8.7 Hz, ^3J = 1.5 Hz, 1 H, H*H*-2), 2.50 – 2.41 (m, 1 H, *H*H-3), 2.19 – 2.07 (m, 1 H, H*H*-3), 0.89 (s, 3 H, CH_3).

^{13}C NMR (101 MHz, $CDCl_3$, 298 K): δ [ppm] = 216.9 (s, C-1), 141.1 (s, C-11a), 136.8 (s, C-3b-d_4), 136.7 (s, C-3b), 135.1 (s, C-7a), 135.1 (s, C-7a-d_4), 133.9 (s, C-7b), 128.0 (d, C-5), 127.3 (d, C-6), 125.0 (d, C-4), 124.9 (d, C-4-d_4), 124.5 (d, C-7), 48.3 (s, C-11b-d_4), 48.2 (s, C-11b), 46.6 (d, C-3a-d_4), 46.1 (t, $^1J_{C\text{-}D}$ = 18.8 Hz, C-3a), 38.1 (t, C-2), 20.1 (t, C-3-d_4), 20.0 (t, C-3), 17.4 (q, CH_3).

When carbon atoms in proximity to C-3a gave distinct signals in the ^{13}C NMR spectrum for **11a**-d_5 and **11a**-d_4, signals assigned to **11a**-d_4 are marked accordingly. With the exception of C-3a, signals of deuterated carbon atoms were not observed by ^{13}C NMR spectroscopy.

HRMS (EI, 70 eV): calculated for $C_{18}H_{12}D_4O$ $[M]^+$: 252.1447, found: 252.1445,
calculated for $C_{18}H_{11}D_5O$ $[M]^+$: 253.1510, found: 253.1509.

(3a*R*,11b*S*)-10,11b-Dimethyl-2,3,3a,11b-tetrahydro-1*H*-cyclopenta[*l*]phenanthren-1-one (11b)

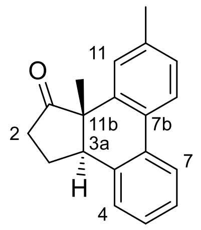

11b
$C_{19}H_{18}O$
M = 262.35 g/mol

According to GP B, **9b** (13.1 mg, 50.0 µmol, 1.00 eq.) was irradiated at λ = 405 nm for 210 min in the presence of **10b**·$AlBr_3$ (1.25 µmol, 2.50 mol%). Automated column chromatography (silica, Hex/EtOAc = 100:0→85:15) afforded **11b** (9.10 mg, 34.7 µmol, 69%) as a colorless oil, which was further reduced to the corresponding alcohol to enable chiral HPLC separation and thus determination of *ee* (*vide infra*).

TLC: R_f = 0.56 (Hex/EtOAc = 4:1) [UV].

IR (ATR): $\tilde{\nu}$ [cm^{-1}] = 2961 (m, sp^3-CH), 2917 (m, sp^3-CH), 1735 (vs, C=O), 1448 (s, sp^3-CH), 1008 (s), 821 (s), 773 (vs, Ar-CH), 751 (vs, Ar-CH), 734 (vs, Ar-CH).

^{1}H NMR (500 MHz, $CDCl_3$, 298 K): δ [ppm] = 8.11 (d, ^4J = 1.9 Hz, 1 H, H-11), 7.73 (dd, ^3J = 7.4 Hz, ^4J = 1.6 Hz, 1 H, H-7), 7.69 (d, ^3J = 7.9 Hz, 1 H, H-8), 7.39 – 7.29 (m, 2 H, H-5, H-6), 7.26 – 7.24 (m, 1 H, H-4), 7.17 (dd, ^3J = 7.9 Hz, ^4J = 1.9 Hz, 1 H, H-9), 3.37 (dd, ^3J = 12.7 Hz, ^3J = 5.9 Hz, 1 H, H-3a), 2.76 (ddd, ^2J = 19.0 Hz, ^3J = 8.4 Hz, ^3J = 0.9 Hz, 1 H, *H*H-2), 2.56 (ddd, ^2J = 19.0 Hz, ^3J = 10.1 Hz, ^3J = 8.8 Hz, 1 H, H*H*-2), 2.48 – 2.42 (m, 1 H, *H*H-3), 2.42 (s, 3 H, Ar-CH_3), 2.12 (*virt.* tdd, $^2J \approx {}^3J$ = 12.4 Hz, ^3J = 10.1 Hz, ^3J = 8.4 Hz, 1 H, H*H*-3), 0.88 (s, 3 H, CH_3).

^{13}C NMR (126 MHz, $CDCl_3$, 298 K): δ [ppm] = 217.1 (s, C-1), 141.1 (s, C-11a), 138.0 (s, C-10), 136.5 (s, C-3b), 135.1 (s, C-7a), 131.2 (s, C-7b), 128.1 (d, C-9), 127.6 (d, C-5), 127.2 (d, C-6), 125.0 (d, C-8), 124.8 (d, C-4), 124.4 (d, C-11), 124.1 (d, C-7), 48.2 (s, C-11b), 46.6 (d, C-3a), 38.2 (t, C-2), 21.6 (q, Ar-CH_3), 20.1 (t, C-3), 17.4 (q, CH_3).

HRMS (EI, 70 eV): calculated for $C_{19}H_{18}O$ $[M]^+$: 262.1352, found: 262.1351,
calculated for $C_{18}{}^{13}CH_{18}O$ $[M]^+$: 263.1386, found: 263.1380.

(1*R*,3a*R*,11b*S*)-10,11b-Dimethyl-2,3,3a,11b-tetrahydro-1*H*-cyclopenta[*l*]phenanthren-1-ol (12b)

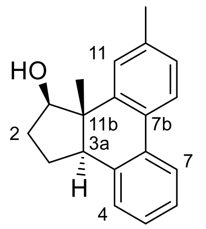

12b

$C_{19}H_{20}O$

M = 264.37 g/mol

According to GP C, **11b** (9.10 mg, 34.7 µmol, 1.00 eq.) was reacted with $NaBH_4$ (5.19 mg, 137 µmol, 4.00 eq.) in 4 mL of MeOH/CH_2Cl_2 (1:1) for 1 h to yield alcohol **12b** (5.00 mg, 18.9 µmol, 55%, 88% *ee*) as a colorless oil after purification by automated column chromatography (silica, Hex/EtOAc = 95:5→60:40).

TLC: R_f = 0.36 (Hex/EtOAc = 4:1) [UV].

IR (ATR): $\tilde{\nu}$ [cm^{-1}] = 3343 (s, broad, O-H), 2962 (s, sp^3-CH), 2917 (s, sp^3-CH), 2850 (s, sp^3-CH), 1448 (s, sp^3-CH), 1407 (s, sp^3-CH), 1066 (s, C-O), 997 (s), 823 (s, Ar-CH), 775 (vs, Ar-CH), 747 (vs, Ar-CH), 736 (vs, Ar-CH).

^{1}H NMR (500 MHz, $CDCl_3$, 298 K): δ [ppm] = 7.71 (dd, ^3J = 7.5 Hz, ^4J = 1.6 Hz, 1 H, H-7), 7.65 (d, ^3J = 7.9 Hz, 1 H, H-8), 7.52 (d, ^4J = 1.9 Hz, 1 H, H-11), 7.33 – 7.29 (m, 1 H, H-6), 7.29 – 7.26 (m, 1 H, H-5), 7.14 (dd, ^3J = 7.9 Hz, ^4J = 1.9 Hz, 1 H, H-9), 7.13 – 7.11 (m, 1 H, H-4), 4.54 (dd, ^3J = 9.1 Hz, ^3J = 7.7 Hz, 1 H, H-1)*, 2.96 (dd, ^3J = 12.1 Hz, ^3J = 7.6 Hz, 1 H, H-3a)*, 2.55 (dddd, ^2J = 13.6 Hz, ^3J = 9.6 Hz, ^3J = 9.1 Hz, ^3J = 6.4 Hz, 1 H, *H*H-2), 2.40 (s, 3 H, Ar-CH_3), 2.05 (dddd, ^2J = 12.6 Hz, ^3J = 9.6 Hz, ^3J = 7.6 Hz, ^3J = 3.7 Hz, 1 H, *H*H-3), 1.98 (*virt.* tdd, $^2J \approx {}^3J$ = 12.4 Hz, ^3J = 11.7 Hz, ^3J = 6.4 Hz, 1 H, H*H*-3), 1.77 (dddd, ^2J = 13.6 Hz, ^3J = 11.7 Hz, ^3J = 7.7 Hz, ^3J = 3.7 Hz, 1 H, H*H*-2), 0.79 (s, 3 H, CH_3).

*Observed NOESY correlation used to derive relative configuration.

^{13}C NMR (101 MHz, $CDCl_3$, 298 K): δ [ppm] = 146.3 (s, C-11a), 138.2 (s, C-3b)*, 138.1 (s, C-10)*, 135.2 (s, C-7a), 131.3 (s, C-7b), 127.7 (d, C-9), 127.4 (d, C-5), 126.6 (d, C-6), 125.2 (d, C-4), 125.0 (d, C-11), 124.6 (d, C-8), 123.8 (d, C-7), 78.2 (d, C-1), 46.6 (s, C-11b), 46.1 (d, C-3a), 33.2 (t, C-2), 21.6 (q, Ar-CH_3), 21.2 (t, C-3), 13.1 (q, CH_3).

*Assignments are interchangeable.

Chiral HPLC: 88% *ee* [Daicel Chiralcel, OD-RH, 150x4.6, H_2O/MeCN = 80:20→0:100 (30 min), 1 mL/min, 215 nm, t_R = 19.70 min (minor), 21.07 min (major)].

(3a*R*,11b*S*)-10-Fluoro-11b-methyl-2,3,3a,11b-tetrahydro-1*H*-cyclopenta[*l*]phenanthren-1-one (11c)

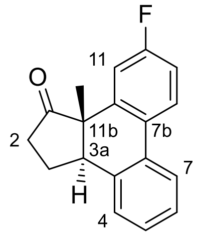

11c
$C_{18}H_{15}FO$
M = 266.32 g/mol

According to GP B, **9c** (13.3 mg, 50.0 µmol, 1.00 eq.) was irradiated at λ = 368 nm for 90 min in the presence of **10b**·$AlBr_3$ (2.50 µmol, 5.00 mol%). Automated column chromatography (silica, Hex/EtOAc = 98:2→80:20) afforded **11c** (8.70 mg, 32.7 µmol, 65%, 89% *ee*) as a colorless solid.

TLC: R_f = 0.87 (Hex/EtOAc = 2:1) [UV].

M.p.: 95 °C.

IR (ATR): $\tilde{\nu}$ [cm^{-1}] = 2960 (m, sp^3-CH), 2922 (s, sp^3-CH), 2850 (m, sp^3-CH), 1735 (vs, C=O), 1448 (s, sp^3-CH), 1002 (vs, C-F), 773 (vs, Ar-CH), 751 (vs, Ar-CH), 731 (vs, Ar-CH).

^{1}H NMR (400 MHz, $CDCl_3$, 298 K): δ [ppm] = 8.03 (dd, $^3J_{H\text{-}F}$ = 10.0 Hz, ^4J = 2.8 Hz, 1 H, H-11), 7.74 (dd, ^3J = 8.7 Hz, $^4J_{H\text{-}F}$ = 5.6 Hz, 1 H, H-8), 7.69 (dd, ^3J = 7.0 Hz, ^4J = 2.0 Hz, 1 H, H-7), 7.40 – 7.31 (m, 2 H, H-5, H-6), 7.28 – 7.22 (m, 1 H, H-4), 7.03 (*virt.* td, $^3J_{H\text{-}F} \approx {}^3J$ = 8.5 Hz, ^4J = 2.8 Hz, 1 H, H-9), 3.35 (dd, ^3J = 12.6 Hz, ^3J = 5.9 Hz, 1 H, H-3a), 2.77 (ddd, ^2J = 18.9 Hz, ^3J = 8.6 Hz, ^3J = 0.9 Hz, 1 H, *H*H-2), 2.57 (ddd, ^2J = 18.9 Hz, ^3J = 9.8 Hz, ^3J = 8.8 Hz, 1 H, H*H*-2), 2.46 (dddd, ^2J = 12.3 Hz, ^3J = 8.8 Hz, ^3J = 5.9 Hz, ^3J = 0.9 Hz, 1 H, *H*H-3), 2.12 (*virt.* tdd, $^2J \approx {}^3J$ = 12.4 Hz, ^3J = 9.8 Hz, ^3J = 8.6 Hz, 1 H, H*H*-3), 0.88 (s, 3 H, CH_3).

^{13}C NMR (126 MHz, $CDCl_3$, 298 K): δ [ppm] = 216.4 (s, C-1), 162.4 (d, $^1J_{C\text{-}F}$ = 247.5 Hz, C-10), 143.3 (d, $^3J_{C\text{-}F}$ = 8.4 Hz, C-11a), 136.1 (s, C-3b)*, 134.4 (s, C-7a)*, 130.2 (d, $^4J_{C\text{-}F}$ = 3.0 Hz, C-7b), 128.0 (d, C-5), 127.4 (d, C-6), 126.7 (d, $^3J_{C\text{-}F}$ = 8.5 Hz, C-8), 125.0 (d, C-4), 124.3 (d, C-7), 114.2 (d, $^2J_{C\text{-}F}$ = 21.7 Hz, C-9), 111.3 (d, $^2J_{C\text{-}F}$ = 23.4 Hz, C-11), 48.1 (d, $^4J_{C\text{-}F}$ = 1.9 Hz, C-11b), 46.4 (d, C-3a), 37.9 (t, C-2), 19.9 (t, C-3), 17.1 (q, CH_3).

*Assignments are interchangeable.

^{19}F NMR (376 MHz, $CDCl_3$, 298 K): δ [ppm] = -112.60 (ddd, $^3J_{H\text{-}F}$ = 10.0 Hz, $^3J_{H\text{-}F}$ = 8.2 Hz, $^4J_{H\text{-}F}$ = 5.6 Hz).

Chiral HPLC: 89% *ee* [Daicel Chiralcel, OJ-RH, 150x4.6, H_2O/MeCN = 80:20→0:100 (30 min), 1 mL/min, 215 nm, t_R = 19.01 min (minor), 19.66 min (major)].

HRMS (EI, 70 eV): calculated for $C_{18}H_{15}OF$ [M]$^+$: 266.1101, found: 266.1101,
calculated for $C_{17}{}^{13}CH_{15}OF$ [M]$^+$: 267.1135, found: 267.1131.

(3a*R*,11b*S*)-11b-Methyl-10-(trifluoromethyl)-2,3,3a,11b-tetrahydro-1*H*-cyclopenta[*l*]-phenanthren-1-one (11d)

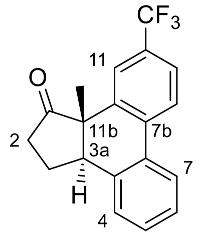

11d
$C_{19}H_{15}F_3O$
M = 316.32 g/mol

According to GP B, **9d** (15.8 mg, 50.0 µmol, 1.00 eq.) was irradiated at λ = 368 nm for 60 min in the presence of **10b**·$AlBr_3$ (2.50 µmol, 5.00 mol%). Automated column chromatography (silica, Hex/EtOAc = 98:2→85:15) afforded **11d** (7.40 mg, 23.4 µmol, 47%, 85% *ee*) as a colorless solid.

TLC: R_f = 0.64 (Hex/EtOAc = 2:1) [UV].

M.p.: 108-110 °C.

IR (ATR): $\tilde{\nu}$ [cm^{-1}] = 2963 (m, sp^3-CH), 2922 (w, sp^3-CH), 1735 (vs, C=O), 1333 (vs, C-F), 1267 (s, C-F), 1103 (vs, C-F), 1077 (vs, C-F), 757 (s, Ar-CH), 736 (s, Ar-CH).

^{1}H NMR (500 MHz, $CDCl_3$, 298 K): δ [ppm] = 8.58 (d, ^4J = 2.0 Hz, 1 H, H-11), 7.87 (d, ^3J = 8.2 Hz, 1 H, H-8), 7.80 – 7.75 (m, 1 H, H-7), 7.60 (dd, ^3J = 8.2 Hz, ^4J = 2.0 Hz, 1 H, H-9), 7.45 – 7.39 (m, 2 H, H-5, H-6), 7.31 – 7.28 (m, 1 H, H-4), 3.38 (ddd, ^3J = 12.5 Hz, ^3J = 5.9 Hz, ^4J = 1.1 Hz, 1 H, H-3a), 2.80 (ddd, ^2J = 19.2 Hz, ^3J = 8.5 Hz, ^3J = 0.9 Hz, 1 H, *H*H-2), 2.59 (ddd, ^2J = 19.2 Hz, ^3J = 10.0 Hz, ^3J = 8.8 Hz, 1 H, H*H*-2), 2.48 (dddd, ^2J = 12.2 Hz, ^3J = 8.8 Hz, ^3J = 5.9 Hz, ^3J = 0.9 Hz, 1 H, *H*H-3), 2.14 (dddd, ^3J = 12.5 Hz, ^2J = 12.2 Hz, ^3J = 10.0 Hz, ^3J = 8.5 Hz, 1 H, H*H*-3), 0.90 (s, 3 H, CH_3).

^{13}C NMR (126 MHz, $CDCl_3$, 298 K): δ [ppm] = 216.0 (s, C-1), 141.7 (s, C-11a), 137.4 (s, C-7b), 137.1 (s, C-3b), 133.9 (s, C-7a), 129.7 (q, $^2J_{C\text{-}F}$ = 32.3 Hz, C-10), 129.2 (d, C-5), 127.6 (d, C-6), 125.3 (d, C-8), 125.2 (d, C-4), 125.0 (d, C-7), 124.3 (q, $^1J_{C\text{-}F}$ = 272.0 Hz, CF_3), 124.3 (q, $^3J_{C\text{-}F}$ = 3.6 Hz, C-9), 121.1 (q, $^3J_{C\text{-}F}$ = 3.6 Hz, C-11), 47.9 (s, C-11b), 46.2 (d, C-3a), 37.9 (t, C-2), 20.0 (t, C-3), 17.1 (q, CH_3).

^{19}F NMR (376 MHz, $CDCl_3$, 298 K): δ [ppm] = -62.4.

Chiral HPLC: 85% *ee* [Daicel Chiralcel, OJ-RH, 150x4.6, H_2O/MeCN = 80:20→0:100 (30 min), 1 mL/min, 215 nm, t_R = 19.94 min (minor), 20.37 min (major)].

HRMS (EI, 70 eV): calculated for $C_{19}H_{15}OF_3$ $[M]^+$: 316.1070, found: 316.1068,
calculated for $C_{18}{}^{13}CH_{15}OF_3$ $[M]^+$: 317.1103, found: 317.1099.

(3a*R*,11b*S*)-10-(*Tert*-butyl)-11b-methyl-2,3,3a,11b-tetrahydro-1*H*-cyclopenta[*l*]phenanthren-1-one (11e)

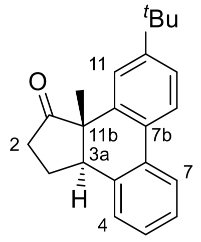

11e
$C_{22}H_{24}O$
M = 304.43 g/mol

According to GP B, **9e** (15.2 mg, 50.0 µmol, 1.00 eq.) was irradiated at λ = 405 nm for 120 min in the presence of **10b**·$AlBr_3$ (2.50 µmol, 5.00 mol%). Automated column chromatography (silica, Hex/EtOAc = 100:0→85:15) afforded **11e** (6.00 mg, 19.7 µmol, 39%, 95% *ee*) as a colorless solid.

TLC: R_f = 0.93 (Hex/EtOAc = 2:1) [UV].

M.p.: 194 °C.

IR (ATR): $\tilde{\nu}$ [cm^{-1}] = 2961 (s, sp^3-CH), 2905 (m, sp^3-CH), 2868 (m, sp^3-CH), 2814 (w, sp^3-CH), 1739 (vs, C=O), 1478 (s, sp^3-CH), 1452 (s, sp^3-CH), 1360 (s, sp^3-CH), 1258 (s, sp^3-CH), 1012 (s), 757 (vs, Ar-CH), 740 (vs, Ar-CH).

^{1}H NMR (400 MHz, $CDCl_3$, 298 K): δ [ppm] = 8.36 (d, ^4J = 2.1 Hz, 1 H, H-11), 7.73 (dd, ^3J = 7.2 Hz, ^4J = 1.8 Hz, 1 H, H-7), 7.72 (d, ^3J = 8.2 Hz, 1 H, H-8), 7.38 (dd, ^3J = 8.2 Hz, ^4J = 2.1 Hz, 1 H, H-9), 7.39 – 7.30 (m, 2 H, H-5, H-6), 7.26 – 7.23 (m, 1 H, H-4), 3.40 (dd, ^3J = 12.7 Hz, ^3J = 5.9 Hz, 1 H, H-3a), 2.76 (ddd, ^2J = 19.0 Hz, ^3J = 8.5 Hz, ^3J = 0.9 Hz, 1 H, *H*H-2), 2.56 (ddd, ^2J = 19.0 Hz, ^3J = 10.0 Hz, ^3J = 8.7 Hz, 1 H, H*H*-2), 2.45 (dddd, ^2J = 12.1 Hz, ^3J = 8.7 Hz, ^3J = 5.9 Hz, ^3J = 0.9 Hz, 1 H, *H*H-3), 2.12 (dddd, ^3J = 12.7 Hz, ^2J = 12.1 Hz, ^3J = 10.0 Hz, ^3J = 8.5 Hz, 1 H, H*H*-3), 1.39 (s, 9 H, $C(CH_3)_3$), 0.90 (s, 3 H, CH_3).

^{13}C NMR (101 MHz, $CDCl_3$, 298 K): δ [ppm] = 217.0 (s, C-1), 151.3 (s, C-10), 141.0 (s, C-11a), 136.7 (s, C-3b), 135.2 (s, C-7a), 131.2 (s, C-7b), 127.6 (d, C-5), 127.2 (d, C-6), 124.8 (d, C-4), 124.7 (d, C-8), 124.2 (d, C-7)*, 124.2 (d, C-9)*, 120.9 (d, C-11), 48.4 (s, C-11b), 46.7 (d, C-3a), 38.2 (t, C-2), 35.1 (s, *C*(CH_3)$_3$), 31.5 (q, 3 C, C(*C*H_3)$_3$), 20.1 (t, C-3), 17.5 (q, CH_3).
*Assignments are interchangeable.

Chiral HPLC: 95% *ee* [Daicel Chiralcel, OJ-RH, 150x4.6, H_2O/MeCN = 80:20→0:100 (30 min), 1 mL/min, 215 nm, t_R = 21.13 min (minor), 22.04 min (major)].

HRMS (EI, 70 eV): calculated for $C_{22}H_{24}O$ $[M]^+$: 304.1822, found: 304.1820.

(3a*R*,11b*S*)-10-Chloro-11b-methyl-2,3,3a,11b-tetrahydro-1*H*-cyclopenta[*l*]phenanthren-1-one (11f)

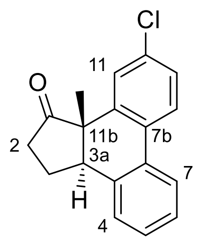

11f
$C_{18}H_{15}ClO$
M = 282.76 g/mol

According to GP B, **9f** (14.1 mg, 50.0 µmol, 1.00 eq.) was irradiated at λ = 368 nm for 60 min in the presence of **10b**·$AlBr_3$ (2.50 µmol, 5.00 mol%). Automated column chromatography (silica, Hex/EtOAc = 98:2→85:15) afforded **11f** (10.6 mg, 37.5 µmol, 75%, 82% *ee*) as an off-white solid.

TLC: R_f = 0.62 (Hex/EtOAc = 2:1) [UV].

M.p.: 62 °C.

IR (ATR): $\tilde{\nu}$ [cm^{-1}] = 3068 (w, Ar-CH), 2965 (m, sp^3-CH), 2924 (m, sp^3-CH), 1735 (vs, C=O), 1476 (s, sp^3-CH), 1444 (s, sp^3-CH), 822 (s, C-Cl), 772 (vs, C-Cl), 753 (vs, Ar-CH), 731 (vs, Ar-CH).

^{1}H NMR (500 MHz, $CDCl_3$, 298 K): δ [ppm] = 8.30 (d, ^4J = 2.3 Hz, 1 H, H-11), 7.73 – 7.68 (m, 1 H, H-7), 7.69 (d, ^3J = 8.4 Hz, 1 H, H-8), 7.41 – 7.34 (m, 2 H, H-5, H-6), 7.31 (dd, ^3J = 8.4 Hz, ^4J = 2.3 Hz, 1 H, H-9), 7.29 – 7.23 (m, 1 H, H-4), 3.34 (ddd, ^3J = 12.7 Hz, ^3J = 5.9 Hz, ^4J = 1.2 Hz, 1 H, H-3a), 2.77 (ddd, ^2J = 19.2 Hz, ^3J = 8.5 Hz, ^3J = 0.9 Hz, 1 H, *H*H-2), 2.56 (ddd, ^2J = 19.2 Hz, ^3J = 10.0 Hz, ^3J = 8.8 Hz, 1 H, H*H*-2), 2.45 (dddd, ^2J = 12.1 Hz, ^3J = 8.8 Hz, ^3J = 5.9 Hz, ^3J = 0.9 Hz, 1 H, *H*H-3), 2.12 (dddd, ^3J = 12.7 Hz, ^2J = 12.1 Hz, ^3J = 10.0 Hz, ^3J = 8.5 Hz, 1 H, H*H*-3), 0.87 (s, 3 H, CH_3).

^{13}C NMR (126 MHz, $CDCl_3$, 298 K): δ [ppm] = 216.2 (s, C-1), 142.7 (s, C-11a), 136.5 (s, C-3b), 134.2 (s, C-7a), 133.8 (s, C-10), 132.5 (s, C-7b), 128.4 (d, C-5), 127.5 (d, C-6), 127.4 (d, C-9), 126.4 (d, C-8), 125.1 (d, C-4), 124.4 (d, C-7), 124.3 (d, C-11), 48.1 (s, C-11b), 46.3 (d, C-3a), 37.9 (t, C-2), 20.0 (t, C-3), 17.1 (q, CH_3).

Chiral HPLC: 82% *ee* [Daicel Chiralcel, OJ-RH, 150x4.6, H_2O/MeCN = 80:20→0:100 (30 min), 1 mL/min, 215 nm, t_R = 20.87 min (minor), 21.88 min (major)].

HRMS (EI, 70 eV): calculated for $C_{18}H_{15}O^{35}Cl$ [M]$^+$: 282.0806, found: 282.0806,
calculated for $C_{18}H_{15}O^{37}Cl$ [M]$^+$: 284.0776, found: 284.0784.

(3a*R*,11b*S*)-9,11,11b-Trimethyl-2,3,3a,11b-tetrahydro-1*H*-cyclopenta[*l*]phenanthren-1-one (11g)

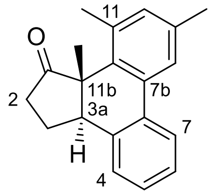

11g
$C_{20}H_{20}O$
M = 276.38 g/mol

According to GP B, **9g** (13.8 mg, 50.0 µmol, 1.00 eq.) was irradiated at λ = 405 nm for 80 min in the presence of **10b**·$AlBr_3$ (2.50 µmol, 5.00 mol%). Automated column chromatography (silica, Hex/EtOAc = 100:0→85:15) afforded **11g** (11.5 mg, 41.6 µmol, 83%, 97% *ee*) as a colorless oil.

TLC: R_f = 0.70 (Hex/EtOAc = 2:1) [UV].

IR (ATR): $\tilde{\nu}$ [cm^{-1}] = 2963 (s, sp^3-CH), 2922 (s, sp^3-CH), 1741 (vs, C=O), 1444 (s, sp^3-CH), 854 (s, Ar-CH), 775 (vs, Ar-CH), 749 (vs, Ar-CH), 731 (vs, Ar-CH).

^{1}H NMR (500 MHz, $CDCl_3$, 298 K): δ [ppm] = 7.62 (dd, ^3J = 7.2 Hz, ^4J = 1.9 Hz, 1 H, H-7), 7.48 – 7.44 (m, 1 H, H-8), 7.39 – 7.30 (m, 2 H, H-5, H-6), 7.25 – 7.22 (m, 1 H, H-4), 7.05 – 7.01 (m, 1 H, H-10), 3.30 (dd, ^3J = 12.8 Hz, ^3J = 6.1 Hz, 1 H, H-3a), 2.82 (ddd, ^2J = 18.2 Hz, ^3J = 9.7 Hz, ^3J = 1.6 Hz, 1 H, *H*H-2), 2.56 (s, 3 H, (CH_3)-11), 2.51 (*virt.* dt, ^2J = 18.2 Hz, ^3J = ^3J = 9.1 Hz, 1 H, H*H*-2), 2.40 (dddd, ^2J = 12.3 Hz, ^3J = 9.1 Hz, ^3J = 6.1 Hz, ^3J = 1.6 Hz, 1 H, *H*H-3), 2.37 (s, 3 H, (CH_3)-9), 2.18 (dddd, ^3J = 12.8 Hz, ^2J = 12.3 Hz, ^3J = 9.7 Hz, ^3J = 9.1 Hz, 1 H, H*H*-3), 1.05 (s, 3 H, CH_3).

^{13}C NMR (101 MHz, $CDCl_3$, 298 K): δ [ppm] = 213.8 (s, C-1), 136.6 (s, 2 C, C-9, C-11), 136.3 (s, C-11a)*, 136.2 (s, C-7a)*, 135.9 (s, C-3b), 135.4 (s, C-7b), 132.9 (d, C-10), 127.5 (d, C-5), 127.3 (d, C-6), 125.3 (d, C-7), 124.5 (d, C-4), 123.7 (d, C-8), 52.1 (s, C-11b), 47.2 (d, C-3a), 37.3 (t, C-2), 23.7 (q, C-11-*C*H_3), 21.1 (q, C-9-*C*H_3), 19.1 (t, C-3), 14.7 (q, CH_3).

*Assignments are interchangeable.

Chiral HPLC: 97% *ee* [Daicel Chiralcel, OJ-RH, 150x4.6, H_2O/MeCN = 80:20→0:100 (30 min), 1 mL/min, 215 nm, t_R = 20.73 min (minor), 22.84 min (major)].

HRMS (EI, 70 eV): calculated for $C_{20}H_{20}O$ [M]$^+$: 276.1509, found: 276.1505,
calculated for $C_{19}{}^{13}CH_{20}O$ [M]$^+$: 277.1542, found: 277.1539.

(3a*R*,11b*S*)-9,11-Difluoro-11b-methyl-2,3,3a,11b-tetrahydro-1*H*-cyclopenta[*l*]phen-anthren-1-one (11h)

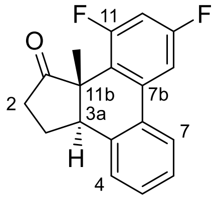

11h
$C_{18}H_{14}F_2O$
M = 284.31 g/mol

According to GP B, **9h** (14.2 mg, 50.0 µmol, 1.00 eq.) was irradiated at λ = 368 nm for 150 min in the presence of **10b**·$AlBr_3$ (2.50 µmol, 5.00 mol%). Automated column chromatography (silica, Hex/EtOAc = 98:2→80:20) afforded **11h** (7.80 mg, 26.3 µmol, 53%, 83% *ee*) as a yellow oil.

TLC: R_f = 0.63 (Hex/EtOAc = 2:1) [UV].

IR (ATR): $\tilde{\nu}$ [cm^{-1}] = 2958 (s, sp^3-CH), 2924 (vs, sp^3-CH), 2855 (s, sp^3-CH), 1750 (s, C=O), 1463 (s, sp^3-CH), 1262 (s, C-F), 1019 (s, C-F), 800 (s, Ar-CH).

^{1}H NMR (500 MHz, $CDCl_3$, 298 K): δ [ppm] = 7.62 (dd, ^3J = 7.3 Hz, ^4J = 1.8 Hz, 1 H, H-7), 7.43 – 7.36 (m, 2 H, H-5, H-6), 7.32 (ddd, $^3J_{H-F}$ = 9.5 Hz, ^4J = 2.5 Hz, $^5J_{H-F}$ = 1.1 Hz, 1 H, H-8), 7.29 – 7.26 (m, 1 H, H-4), 6.81 (ddd, $^3J_{H-F}$ = 10.6 Hz, $^3J_{H-F}$ = 8.6 Hz, ^4J = 2.5 Hz, 1 H, H-10), 3.35 (dd, ^3J = 12.7 Hz, ^3J = 6.0 Hz, 1 H, H-3a), 2.82 (ddd, ^2J = 18.6 Hz, ^3J = 9.2 Hz, ^3J = 1.3 Hz, 1 H, *H*H-2), 2.57 (ddd, ^2J = 18.6 Hz, ^3J = 9.4 Hz, ^3J = 9.0 Hz, 1 H, H*H*-2), 2.45 (dddd, ^2J = 12.3 Hz, ^3J = 9.0 Hz, ^3J = 6.0 Hz, ^3J = 1.3 Hz, 1 H, *H*H-3), 2.15 (*virt.* tt, $^2J \approx {}^3J$ = 12.5 Hz, $^3J \approx {}^3J$ = 9.3 Hz, 1 H, H*H*-3), 0.98 (s, 3 H, CH_3).

^{13}C NMR (126 MHz, $CDCl_3$, 298 K): δ [ppm] = 211.0 (s, C-1), 162.2 (dd, $^1J_{C-F}$ = 246.6 Hz, $^3J_{C-F}$ = 12.6 Hz, C-11)*, 160.4 (dd, $^1J_{C-F}$ = 254.5 Hz, $^3J_{C-F}$ = 12.4 Hz, C-9)*, 138.3 (t, $^3J_{C-F}$ = 9.0 Hz, C-7b), 136.2 (s, C-3b), 133.7 (t, $^4J_{C-F}$ = 2.8 Hz, C-7a), 129.2 (d, C-5), 127.6 (d, C-6), 125.2 (d, C-7), 125.1 (d, C-4), 123.1 (dd, $^2J_{C-F}$ = 18.7 Hz, $^4J_{C-F}$ = 3.7 Hz, C-11a), 107.8 (dd, $^2J_{C-F}$ = 22.5 Hz, $^4J_{C-F}$ = 3.2 Hz, C-8), 104.2 (dd, $^2J_{C-F}$ = 27.7 Hz, $^2J_{C-F}$ = 24.9 Hz, C-10), 50.4 (d, $^3J_{C-F}$ = 6.1 Hz, C-11b), 46.5 (d, C-3a), 37.4 (t, C-2), 19.5 (t, C-3), 15.2 (q, CH_3).

*Assignments are interchangeable.

^{19}F NMR (376 MHz, $CDCl_3$, 298 K): δ [ppm] = -101.87 (dd, J = 10.6 Hz, J = 8.0 Hz), -112.21 (q, J = 8.7 Hz).

Chiral HPLC: 83% *ee* [Daicel Chiralcel, OJ-RH, 150x4.6, H_2O/MeCN = 80:20→0:100 (30 min), 1 mL/min, 215 nm, t_R = 18.70 min (minor), 19.16 min (major)].

HRMS (EI, 70 eV): calculated for $C_{18}H_{14}OF_2$ $[M]^+$: 284.1007, found: 284.1012,
calculated for $C_{17}{}^{13}CH_{14}OF_2$ $[M]^+$: 285.1041, found: 285.1049.

(3a*R*,11b*S*)-9,11-Dichloro-11b-methyl-2,3,3a,11b-tetrahydro-1*H*-cyclopenta[*l*]phenanthren-1-one (11i)

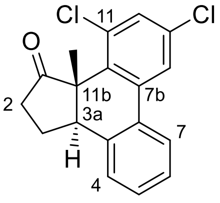

11i

$C_{18}H_{14}Cl_2O$

M = 317.21 g/mol

According to GP B, **9i** (15.9 mg, 50.0 µmol, 1.00 eq.) was irradiated at λ = 368 nm for 75 min in the presence of **10b**·$AlBr_3$ (2.50 µmol, 5.00 mol%). Automated column chromatography (silica, Hex/EtOAc = 98:2→80:20) afforded **11i** (10.0 mg, 30.6 µmol, 61%, 86% *ee*) as a yellow oil.

TLC: R_f = 0.60 (Hex/EtOAc = 2:1) [UV].

IR (ATR): $\tilde{\nu}$ [cm^{-1}] = 2958 (s, sp^3-CH), 2924 (vs, sp^3-CH), 2855 (s, sp^3-CH), 1748 (s, C=O), 1722 (s), 1463 (s, sp^3-CH), 1265 (s), 1098 (s), 805 (s, C-Cl), 771 (s, Ar-CH), 749 (s, Ar-CH).

^{1}H NMR (500 MHz, $CDCl_3$, 298 K): δ [ppm] = 7.68 (d, ^4J = 2.1 Hz, 1 H, H-10), 7.56 (dd, ^3J = 7.1 Hz, ^4J = 1.8 Hz, 1 H, H-7), 7.42 – 7.36 (m, 3 H, H-5, H-6, H-8), 7.27 – 7.23 (m, 1 H, H-4), 3.17 (dd, ^3J = 12.7 Hz, ^3J = 6.4 Hz, 1 H, H-3a), 2.91 (ddd, ^2J = 18.0 Hz, ^3J = 10.7 Hz, ^3J = 2.1 Hz, 1 H, *H*H-2), 2.49 (ddd, ^2J = 18.0 Hz, ^3J = 9.2 Hz, ^3J = 7.8 Hz, 1 H, H*H*-2), 2.38 (dddd, ^2J = 12.3 Hz, ^3J = 9.2 Hz, ^3J = 6.4 Hz, ^3J = 2.1 Hz, 1 H, *H*H-3), 2.25 (*virt.* tdd, $^2J \approx {}^3J$ = 12.5 Hz, ^3J = 10.7 Hz, ^3J = 7.8 Hz, 1 H, H*H*-3), 1.20 (s, 3 H, CH_3).

^{13}C NMR (101 MHz, $CDCl_3$, 298 K): δ [ppm] = 209.4 (s, C-1), 138.7 (s, C-7b), 136.1 (s, C-11a), 135.4 (s, C-3b), 134.4 (s, C-7a), 133.9 (s, C-9)*, 133.8 (s, C-11)*, 130.2 (d, C-8), 129.0 (d, C-5), 127.7 (d, C-6), 125.6 (d, C-7), 125.0 (d, C-4), 123.4 (d, C-10), 51.9 (s, C-11b), 45.7 (d, C-3a), 35.7 (t, C-2), 18.2 (t, C-3), 12.7 (q, CH_3).

*Assignments are interchangeable.

Chiral HPLC: 86% *ee* [Daicel Chiralcel, OD-RH, 150x4.6, H_2O/MeCN = 80:20→0:100 (30 min), 1 mL/min, 215 nm, t_R = 25.04 min (minor), 25.44 min (major)].

HRMS (EI, 70 eV): calculated for $C_{18}H_{14}OCl_2$ $[M]^+$: 316.0416, found: 316.0415,
calculated for $C_{17}{}^{13}CH_{14}OCl_2$ $[M]^+$: 317.0450, found: 317.0453.

(3a*R*,11b*S*)-9,11-Dimethoxy-11b-methyl-2,3,3a,11b-tetrahydro-1*H*-cyclopenta[*l*]phen-anthren-1-one (11j)

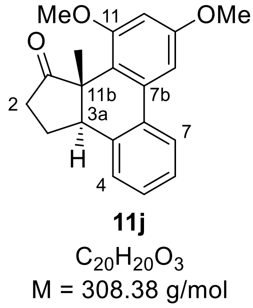

According to GP B, **9j** (15.4 mg, 50.0 µmol, 1.00 eq.) was irradiated at λ = 405 nm for 150 min in the presence of **10b**·$AlBr_3$ (2.50 µmol, 5.00 mol%). Automated column chromatography (silica, Hex/EtOAc = 90:10→60:40) afforded **11j** (5.70 mg, 18.5 µmol, 37%, 81% *ee*) as a yellow oil along with **11j'** (*vide infra*).

TLC: R_f = 0.52 (Hex/EtOAc = 4:1) [UV].

IR (ATR): $\tilde{\nu}$ [cm^{-1}] = 2961 (m, sp^3-CH), 2926 (s, sp^3-CH), 2838 (m, sp^3-CH), 1743 (vs, C=O), 1597 (vs), 1202 (vs, C-O), 1148 (vs, C-O), 1025 (vs), 729 (vs, Ar-CH).

^{1}H NMR (500 MHz, $CDCl_3$, 298 K): δ [ppm] = 7.66 – 7.60 (m, 1 H, H-7), 7.37 – 7.32 (m, 2 H, H-5, H-6), 7.25 – 7.22 (m, 1 H, H-4), 6.97 (d, ^4J = 2.4 Hz, 1 H, H-8), 6.55 (d, ^4J = 2.4 Hz, 1 H, H-10), 3.89 (s, 3 H, H_3CO-11), 3.88 (s, 3 H, H_3CO-9), 3.21 (ddd, ^3J = 12.7 Hz, ^3J = 6.1 Hz, ^4J = 1.1 Hz, 1 H, H-3a), 2.82 (ddd, ^2J = 17.9 Hz, ^3J = 9.8 Hz, ^3J = 1.6 Hz, 1 H, *H*H-2), 2.47 (ddd, ^2J = 17.9 Hz, ^3J = 9.1 Hz, ^3J = 8.7 Hz, 1 H, H*H*-2), 2.37 (dddd, ^2J = 12.2 Hz, ^3J = 9.1 Hz, ^3J = 6.1 Hz, ^3J = 1.6 Hz, 1 H, *H*H-3), 2.16 (*virt.* tdd, $^2J \approx {}^3J$ = 12.5 Hz, ^3J = 9.8 Hz, ^3J = 8.7 Hz, 1 H, H*H*-3), 1.02 (s, 3 H, CH_3).

^{13}C NMR (126 MHz, $CDCl_3$, 298 K): δ [ppm] = 211.7 (s, C-1), 159.9 (s, C-9), 158.9 (s, C-11), 136.8 (s, C-7b), 136.3 (s, C-3b), 135.4 (s, C-7a), 128.0 (d, C-5), 127.2 (d, C-6), 125.0 (d, C-7), 124.8 (d, C-4), 121.9 (s, C-11a), 102.0 (d, C-8), 99.6 (d, C-10), 56.1 (q, C-11-O*C*H_3), 55.6 (q, C-9-O*C*H_3), 50.9 (s, C-11b), 46.7 (d, C-3a), 37.1 (t, C-2), 19.0 (t, C-3), 14.2 (q, CH_3).

Chiral HPLC: 81% *ee* [Daicel Chiralcel, OJ-RH, 150x4.6, H_2O/MeCN = 80:20→0:100 (30 min), 1 mL/min, 215 nm, t_R = 17.16 min (minor), 18.41 min (major)].

HRMS (EI, 70 eV): calculated for $C_{20}H_{20}O_3$ $[M]^+$: 308.1407, found: 308.1409,
calculated for $C_{19}{}^{13}CH_{20}O_3$ $[M]^+$: 309.1441, found: 309.1446.

Relevant correlations observed in NOESY to confirm relative configuration:

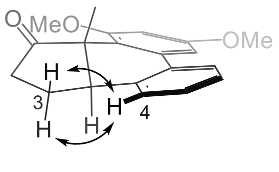

No clear NOE correlation between H-3a and CH_3 is observed, while correlation between both H-3 with H-4 indicates that the five-membered ring is in-plane, and thus *trans*-fused, with the dihydropenanthrene moiety.

(3a*S*,11b*S*)-9,11-Dimethoxy-11b-methyl-2,3,3a,11b-tetrahydro-1*H*-cyclopenta[*l*]phen-anthren-1-one (11j')

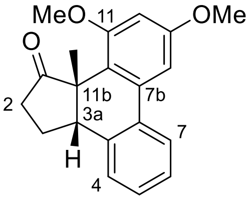

11j'
$C_{20}H_{20}O_3$
M = 308.38 g/mol

11j' (1.20 mg, 3.89 µmol, 8%, 94% *ee*) was isolated as a yellow oil alongside **11j** (*vide supra*).

TLC: R_f = 0.41 (Hex/EtOAc = 4:1) [UV].

IR (ATR): $\tilde{\nu}$ [cm^{-1}] = 2961 (s, sp^3-CH), 2926 (s, sp^3-CH), 2838 (m, sp^3-CH), 1741 (vs, C=O), 1200 (vs, C-O), 1148 (vs, C-O), 1023 (vs), 773 (s, Ar-CH), 751 (s, Ar-CH).

^{1}H NMR (500 MHz, $CDCl_3$, 298 K): δ [ppm] = 7.81 (dd, ^3J = 7.8 Hz, ^4J = 1.3 Hz, 1 H, H-7), 7.37 (ddd, ^3J = 7.8 Hz, ^3J = 7.4 Hz, ^4J = 1.5 Hz, 1 H, H-6), 7.30 (*virt.* td, ^3J = ^3J = 7.4 Hz, ^4J = 1.3 Hz, 1 H, H-5), 7.25 (dd, ^3J = 7.4 Hz, ^4J = 1.5 Hz, 1 H, H-4), 7.04 (d, ^4J = 2.4 Hz, 1 H, H-8), 6.43 (d, ^4J = 2.4 Hz, 1 H, H-10), 3.89 (s, 3 H, H_3CO-9), 3.75 (s, 3 H, H_3CO-11), 2.76 (dd, ^3J = 11.6 Hz, ^3J = 7.5 Hz, 1 H, H-3a), 2.37 (ddd, ^2J = 18.0 Hz, ^3J = 12.2 Hz, ^3J = 2.9 Hz, 1 H, *H*H-2), 2.20 (ddd, ^2J = 18.0 Hz, ^3J = 9.4 Hz, ^3J = 7.7 Hz, 1 H, H*H*-2), 2.05 (dddd, ^2J = 13.1 Hz, ^3J = 9.4 Hz, ^3J = 7.5 Hz, ^3J = 2.9 Hz, 1 H, *H*H-3), 1.63 (dddd, ^2J = 13.1 Hz, ^3J = 12.2 Hz, ^3J = 11.6 Hz, ^3J = 7.7 Hz, 1 H, H*H*-3), 1.09 (s, 3 H, CH_3).

^{13}C NMR (126 MHz, $CDCl_3$, 298 K): δ [ppm] = 217.3 (s, C-1), 160.1 (s, C-9), 158.7 (s, C-11), 137.5 (s, C-3b), 135.2 (s, C-7b), 131.8 (s, C-7a), 128.8 (d, C-4), 128.3 (d, C-5), 127.7 (d, C-6), 124.7 (d, C-7), 116.5 (s, C-11a), 101.2 (d, C-10), 98.4 (d, C-8), 55.5 (q, 2 C, C-9-O*C*H_3, C-11-O*C*H_3), 51.5 (s, C-11b), 48.0 (d, C-3a), 34.7 (t, C-2), 26.0 (t, C-3), 18.2 (q, CH_3).

Chiral HPLC: 94% *ee* [Daicel Chiralcel, OD-RH, 150x4.6, H_2O/MeCN = 80:20→0:100 (30 min), 1 mL/min, 215 nm, t_R = 18.66 min (minor), 19.74 min (major)].

HRMS (ESI) m/z: calculated for [M+H]$^+$: 309.1485, found: 309.1482.

Relevant correlations observed in NOESY to confirm relative configuration:

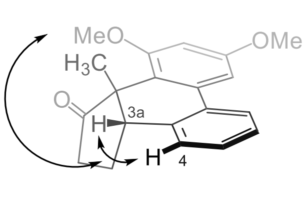

H-3a exhibits NOE correlation with CH_3 as well as H-4, while none is observed between H-4 and HH-3. This indicates a *cis*-fused ring system with the five-membered ring out-of-plane of the dihydrophenanthrene moiety, as is further supported by an upfield shift of the CH_2 signals relative to **11j**.

(3a*R*,11b*S*)-11b-Methyl-9-(trifluoromethyl)-2,3,3a,11b-tetrahydro-1*H*-cyclopenta[*l*]phenanthren-1-one (11k)

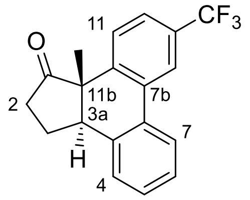

11k
$C_{19}H_{15}F_3O$
M = 316.32 g/mol

According to GP B, **9k** (15.8 mg, 50.0 µmol, 1.00 eq.) was irradiated at λ = 368 nm for 130 min in the presence of **10b**·$AlBr_3$ (2.50 µmol, 5.00 mol%). Automated column chromatography (silica, Hex/EtOAc = 100:0→85:15) afforded **11k** (11.1 mg, 35.1 µmol, 70%, 78% *ee*) as a colorless solid.

TLC: R_f = 0.60 (Hex/EtOAc = 2:1) [UV].

M.p.: 118-121 °C.

IR (ATR): $\tilde{\nu}$ [cm^{-1}] = 2969 (m, sp^3-CH), 2926 (w, sp^3-CH), 1731 (vs, C=O), 1418 (s, sp^3-CH), 1332 (vs, C-F), 1141 (vs, C-F), 846 (s, Ar-CH), 782 (s, Ar-CH), 755 (vs, Ar-CH).

^{1}H NMR (500 MHz, $CDCl_3$, 298 K): δ [ppm] = 8.42 (d, ^3J = 8.2 Hz, 1 H, H-11), 8.01 (d, ^4J = 1.9 Hz, 1 H, H-8), 7.82 – 7.74 (m, 1 H, H-7), 7.57 (dd, ^3J = 8.2 Hz, ^4J = 1.9 Hz, 1 H, H-10), 7.44 – 7.39 (m, 2 H, H-5, H-6), 7.31 – 7.27 (m, 1 H, H-4), 3.37 (ddd, ^3J = 12.6 Hz, ^3J = 5.9 Hz, ^4J = 1.2 Hz, 1 H, H-3a), 2.79 (ddd, ^2J = 19.2 Hz, ^3J = 8.5 Hz, ^3J = 0.8 Hz, 1 H, *H*H-2), 2.58 (ddd, ^2J = 19.2 Hz, ^3J = 10.0 Hz, ^3J = 8.8 Hz, 1 H, H*H*-2), 2.48 (dddd, ^2J = 12.2 Hz, ^3J = 8.8 Hz, ^3J = 5.9 Hz, ^3J = 0.8 Hz, 1 H, *H*H-3), 2.15 (dddd, ^3J = 12.6 Hz, ^2J = 12.2 Hz, ^3J = 10.0 Hz, ^3J = 8.5 Hz, 1 H, H*H*-3), 0.89 (s, 3 H, CH_3).

^{13}C NMR (126 MHz, $CDCl_3$, 298 K): δ [ppm] = 216.1 (s, C-1), 144.6 (s, C-11a), 136.7 (s, C-3b), 134.8 (s, C-7b), 133.8 (s, C-7a), 129.7 (q, $^2J_{C\text{-}F}$ = 32.2 Hz, C-9), 128.9 (d, C-5), 127.6 (d, C-6), 125.1 (d, C-4), 124.7 (d, C-7), 124.6 (q, $^3J_{C\text{-}F}$ = 3.9 Hz, C-10), 124.5 (d, C-11), 124.4 (q, $^1J_{C\text{-}F}$ = 272.2 Hz, CF_3), 121.8 (q, $^3J_{C\text{-}F}$ = 3.9 Hz, C-8), 48.2 (s, C-11b), 46.1 (d, C-3a), 37.9 (t, C-2), 20.0 (t, C-3), 17.0 (q, CH_3).

Chiral HPLC: 78% *ee* [Daicel Chiralcel, OD-RH, 150x4.6, H_2O/MeCN = 80:20→0:100 (30 min), 1 mL/min, 40 °C, 215 nm, t_R = 21.44 min (minor), 21.83 min (major)].

HRMS (EI, 70 eV): calculated for $C_{19}H_{15}OF_3$ $[M]^+$: 316.1070, found: 316.1070.

(3a*R*,11b*S*)-11b-Methyl-9-phenyl-2,3,3a,11b-tetrahydro-1*H*-cyclopenta[*l*]phenanthren-1-one (11l)

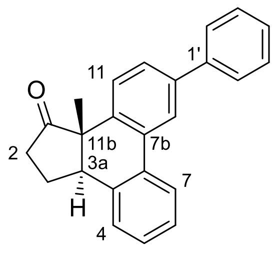

11l

$C_{24}H_{20}O$

M = 324.42 g/mol

According to GP B, **9l** (16.2 mg, 50.0 µmol, 1.00 eq.) was irradiated at λ = 405 nm for 120 min in the presence of **10b**·$AlBr_3$ (2.50 µmol, 5.00 mol%). Automated column chromatography (silica, Hex/EtOAc = 100:0→85:15) afforded **11l** (8.00 mg, 24.7 µmol, 49%, 95% *ee*) as a yellow oil.

TLC: R_f = 0.64 (Hex/EtOAc = 2:1) [UV].

IR (ATR): $\tilde{\nu}$ [cm^{-1}] = 2967 (w, sp^3-CH), 2920 (m, sp^3-CH), 2853 (w, sp^3-CH), 1741 (vs, C=O), 1482 (s, sp^3-CH), 1444 (s, sp^3-CH), 1008 (vs), 757 (vs, Ar-CH), 695 (vs, Ar-CH).

^{1}H NMR (400 MHz, $CDCl_3$, 298 K): δ [ppm] = 8.35 (d, ^3J = 8.0 Hz, 1 H, H-11), 8.00 (d, ^4J = 1.9 Hz, 1 H, H-8), 7.86 – 7.81 (m, 1 H, H-7), 7.68 – 7.61 (m, 2 H, H-2', H-6'), 7.55 (dd, ^3J = 8.0 Hz, ^4J = 1.9 Hz, 1 H, H-10), 7.50 – 7.44 (m, 2 H, H-3', H-5'), 7.43 – 7.33 (m, 3 H, H-4', H-5, H-6), 7.31 – 7.27 (m, 1 H, H-4), 3.43 (dd, ^3J = 12.7 Hz, ^3J = 5.9 Hz, 1 H, H-3a), 2.79 (dd, ^2J = 18.8 Hz, ^3J = 8.5 Hz, 1 H, *H*H-2), 2.58 (ddd, ^2J = 18.8 Hz, ^3J = 9.8 Hz, ^3J = 8.7 Hz, 1 H, H*H*-2), 2.48 (ddd, ^2J = 12.1 Hz, ^3J = 8.7 Hz, ^3J = 5.9 Hz, 1 H, *H*H-3), 2.15 (*virt.* tdd, $^2J \approx {}^3J$ = 12.4 Hz, ^3J = 9.8 Hz, ^3J = 8.5 Hz, 1 H, H*H*-3), 0.93 (s, 3 H, CH_3).

^{13}C NMR (101 MHz, $CDCl_3$, 298 K): δ [ppm] = 216.8 (s, C-1), 141.4 (s, C-1'), 140.5 (s, C-9)*, 140.3 (s, C-11a)*, 136.9 (s, C-3b), 135.1 (s, C-7a), 134.5 (s, C-7b), 129.0 (d, 2 C, C-3', C-5'), 128.2 (d, C-5), 127.5 (d, C-4')†, 127.4 (d, C-6)†, 127.3 (d, 2 C, C-2', C-6'), 126.9 (d, C-10), 125.0 (d, C-4), 124.6 (d, C-7), 124.4 (d, C-11), 124.0 (d, C-8), 48.2 (s, C-11b), 46.6 (d, C-3a), 38.1 (t, C-2), 20.1 (t, C-3), 17.4 (q, CH_3).

*,† Assignments are interchangeable.

Chiral HPLC: 95% *ee* [Daicel Chiralcel, OJ-RH, 150x4.6, H_2O/MeCN = 80:20→0:100 (30 min), 1 mL/min, 215 nm, t_R = 23.86 min (minor), 27.38 min (major)].

HRMS (EI, 70 eV): calculated for $C_{24}H_{20}O$ [M]$^+$: 324.1509, found: 324.1507,
calculated for $C_{23}{}^{13}CH_{20}O$ [M]$^+$: 325.1542, found: 325.1536.

(3a*R*,11b*S*)-9,11b-Dimethyl-2,3,3a,11b-tetrahydro-1*H*-cyclopenta[*l*]phenanthren-1-one (11m) and (3a*R*,11b*S*)-11,11b-dimethyl-2,3,3a,11b-tetrahydro-1*H*-cyclopenta[*l*]phenanthren-1-one (11m')

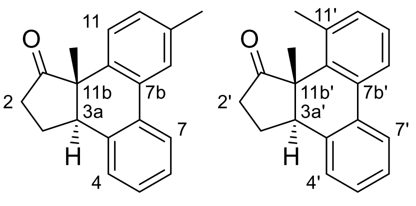

11m **11m'**

$C_{19}H_{18}O$

M = 262.35 g/mol

According to GP B, **9m** (13.1 mg, 50.0 µmol, 1.00 eq.) was irradiated at λ = 405 nm for 210 min in the presence of **10b**·$AlBr_3$ (1.25 µmol, 2.50 mol%). Automated column chromatography (silica, Hex/EtOAc = 100:0→85:15) afforded a mixture of regioisomers **11m** and **11m'** (9.00 mg, 34.3 µmol, 68%, r.r. = 1:3.5, **11m**: 91% *ee*) as a colorless oil. The material was reduced to the respective alcohols to determine *ee* of **11m'** by chiral HPLC (*vide infra*).

TLC: R_f = 0.91 (Hex/EtOAc = 2:1) [UV].

^{1}H NMR (500 MHz, $CDCl_3$, 298 K): δ [ppm] = 8.16 (d, ^{3}J = 7.9 Hz, 0.3 H, H-11), 7.75 (dd, ^{3}J = 7.4 Hz, ^{4}J = 1.6 Hz, 0.3 H, H-7), 7.64 (dd, ^{3}J = 7.6 Hz, ^{4}J = 1.5 Hz, 0.7 H, H-8'), 7.62 – 7.60 (m, 1 H, H-7', H-8), 7.39 – 7.32 (m, 2 H, H-5, H-5', H-6, H-6'), 7.25 (*virt.* t, ^{3}J = ^{3}J = 7.6 Hz, 0.7 H, H-9'), 7.27 – 7.22 (m, 1 H, H-4, H-4'), 7.19 (dd, ^{3}J = 7.6 Hz, ^{4}J = 1.5 Hz, 0.7 H, H-10'), 7.15 (dd, ^{3}J = 7.9 Hz, ^{4}J = 1.9 Hz, 0.3 H, H-10), 3.36 (dd, ^{3}J = 12.7 Hz, ^{3}J = 6.0 Hz, 0.3 H, H-3a), 3.33 (ddd, ^{3}J = 12.8 Hz, ^{3}J = 6.2 Hz, ^{4}J = 1.1 Hz, 0.7 H, H-3a'), 2.83 (ddd, ^{2}J = 18.1 Hz, ^{3}J = 9.8 Hz, ^{3}J = 1.6 Hz, 0.7 H, *H*H-2'), 2.75 (ddd, ^{2}J = 19.0 Hz, ^{3}J = 8.5 Hz, ^{3}J = 0.9 Hz, 0.3 H, *H*H-2), 2.59 (s, 2.1 H, Ar'-CH_3), 2.55 (ddd, ^{2}J = 19.0 Hz, ^{3}J = 9.9 Hz, ^{3}J = 8.8 Hz, 0.3 H, H*H*-2), 2.52 (*virt.* dt, ^{2}J = 18.1 Hz, ^{3}J ≈ ^{3}J = 9.1 Hz, 0.7 H, H*H*-2'), 2.45 (dddd, ^{2}J = 12.0 Hz, ^{3}J = 8.8 Hz, ^{3}J = 6.0 Hz, ^{3}J = 0.9 Hz, 0.3 H, *H*H-3), 2.41 (s, 0.9 H, Ar-CH_3), 2.40 (dddd, ^{2}J = 12.2 Hz, ^{3}J = 9.2 Hz, ^{3}J = 6.2 Hz, ^{3}J = 1.6 Hz, 0.7 H, *H*H-3'), 2.19 (*virt.* tdd, ^{2}J ≈ ^{3}J = 12.5 Hz, ^{3}J = 9.8 Hz, ^{3}J = 9.0 Hz, 0.7 H, H*H*-3'), 2.12 (*virt.* tdd, ^{2}J ≈ ^{3}J = 12.4 Hz, ^{3}J = 9.9 Hz, ^{3}J = 8.5 Hz, 0.3 H, H*H*-3), 1.07 (s, 2.1 H, CH_3'), 0.87 (s, 0.9 H, CH_3).

^{13}C NMR (126 MHz, $CDCl_3$, 298 K): δ [ppm] = 217.1 (s, C-1), 213.8 (s, C-1'), 138.9 (s, C-11a'), 138.4 (s, C-11a), 136.9 (d, 2 C, C-3b, C-9), 136.8 (s, C-11'), 136.2 (s, C-3b'), 135.8 (s, C-7a'), 135.4 (s, C-7b'), 135.1 (s, C-7a), 133.8 (s, C-7b), 132.1 (d, C-10'), 128.7 (d, C-10), 127.9 (d, C-5), 127.6 (d, C-5'), 127.4 (d, C-6'), 127.2 (d, C-6), 127.2 (d, C-9'), 125.7 (d, C-8), 125.3 (d, C-7'), 124.9 (d, C-4), 124.6 (d, C-4'), 124.4 (d, C-7), 123.7 (d, C-11), 122.9 (d, C-8'), 52.3 (s, C-11b'), 48.1 (s, C-11b), 46.9 (d, C-3a'), 46.7 (d, C-3a), 38.1 (t, C-2), 37.2 (t, C-2'), 23.8 (q, C-11'-CH_3), 21.6 (q, C-9-CH_3), 20.1 (t, C-3), 19.0 (t, C-3'), 17.4 (q, CH_3), 14.6 (q, CH_3').

Chiral HPLC: 91% *ee* (**11m**) [Daicel Chiralcel, OD-RH, 150x4.6, H_2O/MeCN = 80:20→0:100 (30 min), 1 mL/min, 215 nm, t_R = 21.86 min (**11m** minor), 23.11 min (**11m** major), 25.68 min (**11m'**)].

HRMS (EI, 70 eV): calculated for $C_{19}H_{18}O$ $[M]^+$: 262.1352, found: 262.1350,
calculated for $C_{18}{}^{13}CH_{18}O$ $[M]^+$: 263.1386, found: 263.1378.

Reduction of 11m/11m'

According to GP C, **11m/11m'** (9.00 mg, 34.3 µmol, 1.00 eq., r.r. = 1:3.5) was reacted with $NaBH_4$ (7.00 mg, 185 µmol, 5.39 eq.) in 4 mL of MeOH/CH_2Cl_2 (1:1) for 1 h to yield alcohols **(*S*)-12m'** (2.40 mg, 9.08 µmol, 26%, 90% *ee*), **(*R*)-12m'** (1.80 mg, 6.81 µmol, 20%, 90% *ee*) and **12m** (2.30 mg, 8.70 µmol, 25%) as off-white solids after purification by automated column chromatography (silica, Hex/EtOAc = 92:8→70:30).

(1S,3aR,11bS)-11,11b-Dimethyl-2,3,3a,11b-tetrahydro-1H-cyclopenta[l]phenanthren-1-ol ((S)-12m')

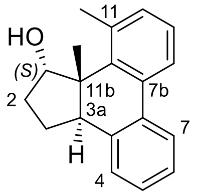

(*S*)-12m'
$C_{19}H_{20}O$
M = 264.37 g/mol

TLC: R_f = 0.52 (Hex/EtOAc = 4:1) [UV].

M.p.: 58-62 °C.

^{1}H NMR (500 MHz, $CDCl_3$, 298 K): δ [ppm] = 7.69 (dd, ^3J = 7.7 Hz, ^4J = 1.4 Hz, 1 H, H-8), 7.64 – 7.60 (m, 1 H, H-7), 7.34 – 7.27 (m, 2 H, H-5, H-6), 7.24 (*virt.* t, $^3J \approx {}^3J$ = 7.6 Hz, 1 H, H-9), 7.22 – 7.18 (m, 1 H, H-4), 7.14 (ddd, ^3J = 7.5 Hz, ^4J = 1.4 Hz, ^4J = 0.7 Hz, 1 H, H-10), 4.73 (d, ^3J = 5.3 Hz, 1 H, H-1), 3.62 (dd, ^3J = 11.8 Hz, ^3J = 7.9 Hz, 1 H, H-3a), 2.60 (s, 3 H, Ar-CH_3), 2.38 (dddd, ^2J = 14.7 Hz, ^3J = 11.3 Hz, ^3J = 5.3 Hz, ^3J = 3.2 Hz, 1 H, *H*H-2), 2.25 (dddd, ^2J = 12.3 Hz, ^3J = 9.5 Hz, ^3J = 7.9 Hz, ^3J = 3.2 Hz, 1 H, *H*H-3), 1.99 (ddd, ^2J = 14.7 Hz, ^3J = 9.5 Hz, ^3J = 5.9 Hz, 1 H, H*H*-2), 1.90 (*virt.* qd, $^2J \approx {}^3J \approx {}^3J$ = 11.8 Hz, ^3J = 5.9 Hz, 1 H, H*H*-3), 0.75 (s, 3 H, CH_3).

^{13}C NMR (126 MHz, $CDCl_3$, 298 K): δ [ppm] = 140.9 (s, C-11a), 138.4 (s, C-3b), 136.7 (s, C-7b), 135.4 (s, C-7a), 134.1 (s, C-11), 131.8 (d, C-10), 127.7 (d, C-5), 127.2 (d, C-9), 126.6 (d, C-6), 125.5 (d, C-4), 124.9 (d, C-7), 123.8 (d, C-8), 77.3 (d, C-1), 52.7 (s, C-11b), 44.8 (d, C-3a), 32.4 (t, C-2), 21.6 (q, Ar-CH_3), 21.1 (t, C-3), 15.4 (q, CH_3).

Chiral HPLC: 90% *ee* [Daicel Chiralcel, OD-RH, 150x4.6, H_2O/MeCN = 80:20→0:100 (30 min), 1 mL/min, 215 nm, t_R = 24.60 min (minor), 26.82 min (major)].

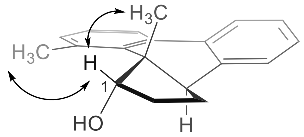

Relevant correlations observed in NOESY to confirm relative configuration.

(1*R*,3a*R*,11b*S*)-11,11b-Dimethyl-2,3,3a,11b-tetrahydro-1*H*-cyclopenta[l]phenanthren-1-ol ((*R*)-12m')

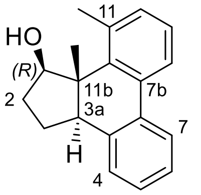

(*R*)-12m'
$C_{19}H_{20}O$
M = 264.37 g/mol

TLC: R_f = 0.47 (Hex/EtOAc = 4:1) [UV].

M.p.: 50-54 °C.

^{1}H NMR (500 MHz, $CDCl_3$, 298 K): δ [ppm] = 7.64 (dd, ^3J = 7.1 Hz, ^4J = 1.8 Hz, 1 H, H-7), 7.61 (dd, ^3J = 7.6 Hz, ^4J = 1.4 Hz, 1 H, H-8), 7.35 – 7.27 (m, 2 H, H-5, H-6), 7.21 (*virt.* t, ^3J = ^3J = 7.6 Hz, 1 H, H-9), 7.17 – 7.10 (m, 2 H, H-4, H-10), 4.76 (dd, ^3J = 9.7 Hz, ^3J = 4.9 Hz, 1 H, H-1), 3.12 (dd, ^3J = 12.6 Hz, ^3J = 6.8 Hz, 1 H, H-3a), 2.66 (s, 3 H, Ar-CH_3), 2.63 (*virt.* dq, ^2J = 14.1 Hz, ^3J ≈ ^3J ≈ ^3J = 9.3 Hz, 1 H, *H*H-2), 2.07 (dddd, ^2J = 11.7 Hz, ^3J = 9.2 Hz, ^3J = 6.8 Hz, ^3J = 2.3 Hz, 1 H, *H*H-3), 2.04 – 1.95 (m, 1 H, H*H*-3), 1.81 (dddd, ^2J = 14.1 Hz, ^3J = 10.5 Hz, ^3J = 4.9 Hz, ^3J = 2.3 Hz, 1 H, H*H*-2), 0.78 (s, 3 H, CH_3).

The sample quantity was insufficient for ^{13}C NMR analysis.

Chiral HPLC: 90% *ee* [Daicel Chiralcel, OD-RH, 150x4.6, H_2O/MeCN = 80:20→0:100 (30 min), 1 mL/min, 215 nm, t_R = 21.30 min (minor), 22.80 min (major)].

Relevant correlations observed in NOESY to confirm relative configuration:

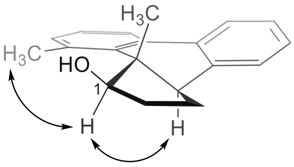

(1*R*,3a*R*,11b*S*)-9,11b-Dimethyl-2,3,3a,11b-tetrahydro-1*H*-cyclopenta[l]phenanthren-1-ol (12m)

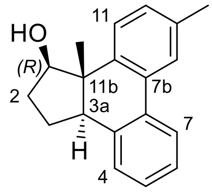

12m

$C_{19}H_{20}O$

M = 264.37 g/mol

TLC: R_f = 0.44 (Hex/EtOAc = 4:1) [UV].

M.p.: 75-77 °C.

^{1}H NMR (500 MHz, $CDCl_3$, 298 K): δ [ppm] = 7.73 (dd, ^3J = 7.2 Hz, ^4J = 1.7 Hz, 1 H, H-7), 7.58 (d, ^3J = 7.9 Hz, 1 H, H-11), 7.57 (s, 1 H, H-8), 7.34 – 7.27 (m, 2 H, H-5, H-6), 7.15 – 7.10 (m, 2 H, H-4, H-10), 4.52 (dd, ^3J = 9.2 Hz, ^3J = 7.7 Hz, 1 H, H-1), 2.95 (dd, ^3J = 12.2 Hz, ^3J = 7.6 Hz, 1 H, H-3a), 2.54 (*virt.* dtd, ^2J = 13.6 Hz, $^3J \approx {}^3J$ = 9.4 Hz, ^3J = 6.4 Hz, 1 H, *H*H-2), 2.41 (s, 3 H, Ar-CH_3), 2.05 (dddd, ^2J = 12.5 Hz, ^3J = 9.6 Hz, ^3J = 7.6 Hz, ^3J = 3.6 Hz, 1 H, *H*H-3), 1.97 (*virt.* qd, $^2J \approx {}^3J \approx {}^3J$ = 12.2 Hz, ^3J = 6.4 Hz, 1 H, H*H*-3), 1.76 (dddd, ^2J = 13.6 Hz, ^3J = 11.5 Hz, ^3J = 7.7 Hz, ^3J = 3.6 Hz, 1 H, H*H*-2), 0.77 (s, 3 H, CH_3).

^{13}C NMR (126 MHz, $CDCl_3$, 298 K): δ [ppm] = 143.6 (s, C-11a), 138.5 (s, C-3b), 136.4 (s, C-9), 135.1 (s, C-7a), 133.9 (s, C-7b), 128.8 (d, C-10), 127.7 (d, C-5), 126.6 (d, C-6), 125.4 (d, C-8)*, 125.2 (d, C-4)*, 124.2 (d, C-11), 124.0 (d, C-7), 78.3 (d, C-1), 46.3 (d, C-3a), 46.1 (s, C-11b), 33.1 (t, C-2), 21.6 (q, Ar-CH_3), 21.2 (t, C-3), 13.2 (q, CH_3).

*Assignments are interchangeable.

Relevant correlations observed in NOESY to confirm relative configuration:

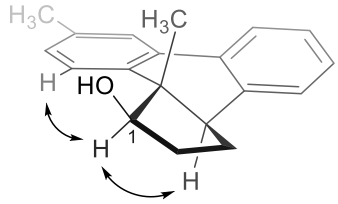

(3a*R*,11b*S*)-9-Fluoro-11b-methyl-2,3,3a,11b-tetrahydro-1*H*-cyclopenta[*l*]phenanthren-1-one (11n)

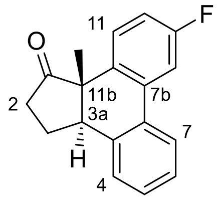

11n
$C_{18}H_{15}FO$
M = 266.32 g/mol

According to GP B, **9n** (13.3 mg, 50.0 µmol, 1.00 eq.) was irradiated at λ = 368 nm for 60 min in the presence of **10b**·$AlBr_3$ (2.50 µmol, 5.00 mol%). Automated column chromatography (silica, Hex/EtOAc = 98:2→80:20) afforded **11n** (4.80 mg, 18.0 µmol, 36%, 87% *ee*) as a colorless oil, along with regioisomer **11n'** (*vide infra*).

TLC: R_f = 0.49 (Hex/EtOAc = 9:1) [UV].

IR (ATR): $\tilde{\nu}$ [cm^{-1}] = 2963 (m, sp^3-CH), 2920 (m, sp^3-CH), 1735 (vs, C=O), 1492 (s, sp^3-CH), 1448 (s, sp^3-CH), 1180 (s, C-F), 824 (s, Ar-CH), 751 (vs, Ar-CH).

^{1}H NMR (500 MHz, $CDCl_3$, 298 K): δ [ppm] = 8.26 (dd, ^3J = 8.6 Hz, $^4J_{H-F}$ = 6.0 Hz, 1 H, H-11), 7.72 – 7.65 (m, 1 H, H-7), 7.46 (dd, $^3J_{H-F}$ = 10.3 Hz, ^4J = 2.7 Hz, 1 H, H-8), 7.42 – 7.35 (m, 2 H, H-5, H-6), 7.31 – 7.23 (m, 1 H, H-4), 7.01 (*virt.* td, $^3J \approx {}^3J_{H-F}$ = 8.5 Hz, ^4J = 2.7 Hz, 1 H, H-10), 3.36 (ddd, ^3J = 12.7 Hz, ^3J = 5.9 Hz, ^4J = 1.1 Hz, 1 H, H-3a), 2.77 (ddd, ^2J = 19.1 Hz, ^3J = 8.6 Hz, ^3J = 1.0 Hz, 1 H, *H*H-2), 2.56 (ddd, ^2J = 19.1 Hz, ^3J = 9.9 Hz, ^3J = 8.8 Hz, 1 H, H*H*-2), 2.46 (dddd, ^2J = 12.2 Hz, ^3J = 8.8 Hz, ^3J = 5.9 Hz, ^3J = 1.0 Hz, 1 H, *H*H-3), 2.12 (dddd, ^3J = 12.7 Hz, ^2J = 12.2 Hz, ^3J = 9.9 Hz, ^3J = 8.6 Hz, 1 H, H*H*-3), 0.86 (s, 3 H, CH_3).
^{13}C NMR (126 MHz, $CDCl_3$, 298 K): δ [ppm] = 216.7 (s, C-1), 162.3 (d, $^1J_{C-F}$ = 244.0 Hz, C-9), 137.0 (d, $^4J_{C-F}$ = 3.0 Hz, C-11a), 136.9 (s, C-3b), 136.2 (d, $^3J_{C-F}$ = 8.0 Hz, C-7b), 134.2 (d, $^4J_{C-F}$ = 2.4 Hz, C-7a), 128.7 (d, C-5), 127.4 (d, C-6), 125.7 (d, $^3J_{C-F}$ = 8.0 Hz, C-11), 125.1 (d, C-4), 124.6 (d, C-7), 114.4 (d, $^2J_{C-F}$ = 21.0 Hz, C-10), 111.8 (d, $^2J_{C-F}$ = 22.6 Hz, C-8), 47.9 (s, C-11b), 46.6 (d, C-3a), 37.9 (t, C-2), 20.0 (t, C-3), 17.4 (q, CH_3).
^{19}F NMR (376 MHz, $CDCl_3$, 298 K): δ [ppm] = -115.16 (ddd, $^3J_{H-F}$ = 10.3 Hz, $^3J_{H-F}$ = 8.3 Hz, $^4J_{H-F}$ = 6.0 Hz).

Chiral HPLC: 87% *ee* [Daicel Chiralcel, OJ-RH, 150x4.6, H_2O/MeCN = 80:20→0:100 (30 min), 1 mL/min, 215 nm, t_R = 19.14 min (minor), 19.94 min (major)].

HRMS (EI, 70 eV): calculated for $C_{18}H_{15}OF$ $[M]^+$: 266.1101, found: 266.1100.

(3a*R*,11b*S*)-11-Fluoro-11b-methyl-2,3,3a,11b-tetrahydro-1*H*-cyclopenta[*l*]phenanthren-1-one (11n')

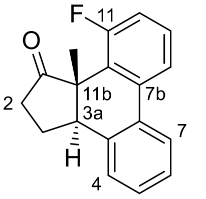

11n'
$C_{18}H_{15}FO$
M = 266.32 g/mol

11n' (6.10 mg, 22.9 µmol, 46%, 95% *ee*) was isolated as a colorless oil alongside **11n** (*vide supra*).

TLC: R_f = 0.37 (Hex/EtOAc = 9:1) [UV].

IR (ATR): $\tilde{\nu}$ [cm^{-1}] = 2967 (m, sp^3-CH), 2926 (m, sp^3-CH), 1748 (vs, C=O), 1457 (s, sp^3-CH), 1237 (s, C-F), 895 (s, Ar-CH), 757 (vs, Ar-CH).

^{1}H NMR (500 MHz, $CDCl_3$, 298 K): δ [ppm] = 7.71 – 7.65 (m, 1 H, H-7), 7.60 (dd, ^3J = 7.8 Hz, ^4J = 1.2 Hz, 1 H, H-8), 7.40 – 7.35 (m, 2 H, H-5, H-6), 7.32 (*virt.* td, $^3J \approx {}^3J$ = 8.0 Hz, $^4J_{H\text{-}F}$ = 5.2 Hz, 1 H, H-9), 7.29 – 7.23 (m, 1 H, H-4), 7.07 (ddd, $^3J_{H\text{-}F}$ = 10.6 Hz, ^3J = 8.2 Hz, ^4J = 1.2 Hz, 1 H, H-10), 3.38 (ddd, ^3J = 12.7 Hz, ^3J = 6.0 Hz, ^4J = 1.2 Hz, 1 H, H-3a), 2.82 (ddd, ^2J = 18.6 Hz, ^3J = 9.1 Hz, ^3J = 1.3 Hz, 1 H, *H*H-2), 2.58 (ddd, ^2J = 18.6 Hz, ^3J = 9.5 Hz, ^3J = 9.0 Hz, 1 H, H*H*-2), 2.45 (dddd, ^2J = 12.2 Hz, ^3J = 9.0 Hz, ^3J = 6.0 Hz, ^3J = 1.3 Hz, 1 H, *H*H-3), 2.16 (*virt.* ddt, ^3J = 12.7 Hz, ^2J = 12.2 Hz, $^3J \approx {}^3J$ = 9.3 Hz, 1 H, H*H*-3), 1.01 (s, 3 H, CH_3).
^{13}C NMR (126 MHz, $CDCl_3$, 298 K): δ [ppm] = 211.4 (s, C-1), 160.0 (d, $^1J_{C\text{-}F}$ = 251.7 Hz, C-11), 137.2 (d, $^3J_{C\text{-}F}$ = 6.8 Hz, C-7b), 136.1 (s, C-3b), 134.5 (d, $^4J_{C\text{-}F}$ = 2.6 Hz, C-7a), 128.8 (d, $^3J_{C\text{-}F}$ = 8.6 Hz, C-9), 128.5 (d, C-5), 127.5 (d, C-6), 126.9 (d, $^2J_{C\text{-}F}$ = 18.2 Hz, C-11a), 125.2 (d, C-7), 125.0 (d, C-4), 120.7 (d, $^4J_{C\text{-}F}$ = 2.9 Hz, C-8), 116.4 (d, $^2J_{C\text{-}F}$ = 23.6 Hz, C-10), 50.7 (d, $^3J_{C\text{-}F}$ = 5.7 Hz, C-11b), 46.5 (d, C-3a), 37.6 (t, C-2), 19.6 (t, C-3), 15.3 (q, CH_3).
^{19}F NMR (471 MHz, $CDCl_3$, 298 K): δ [ppm] = -106.19 (dd, $^3J_{H\text{-}F}$ = 10.6 Hz, $^4J_{H\text{-}F}$ = 5.2 Hz).

Chiral HPLC: 95% *ee* [Daicel Chiralcel, OJ-RH, 150x4.6, H_2O/MeCN = 80:20→0:100 (30 min), 1 mL/min, 215 nm, t_R = 17.66 min (minor), 18.24 min (major)].

HRMS (EI, 70 eV): calculated for $C_{18}H_{15}OF$ [M]$^+$: 266.1101, found: 266.1097,
calculated for $C_{17}{}^{13}CH_{15}OF$ [M]$^+$: 267.1135, found: 267.1131.

(3a*R*,11b*S*)-9-Chloro-11b-methyl-2,3,3a,11b-tetrahydro-1*H*-cyclopenta[*l*]phenanthren-1-one (11o) and (3a*R*,11b*S*)-11-chloro-11b-methyl-2,3,3a,11b-tetrahydro-1*H*-cyclopenta[*l*]phenanthren-1-one (11o')

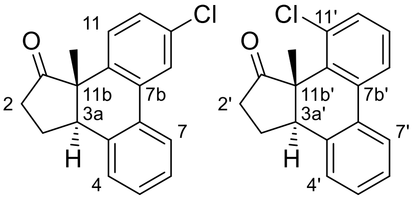

11o **11o'**

$C_{18}H_{15}ClO$
M = 282.77 g/mol

According to GP B, **9o** (14.1 mg, 50.0 µmol, 1.00 eq.) was irradiated at λ = 368 nm for 60 min in the presence of **10b**·$AlBr_3$ (2.50 µmol, 5.00 mol%). Automated column chromatography (silica, Hex/EtOAc = 98:2→80:20) afforded a mixture of regioisomers **11o** and **11o'** (10.3 mg, 36.4 µmol, 73%, r.r. = 1:1.8) as a colorless oil, which was further reduced to the respective alcohols to enable chiral HPLC separation (*vide infra*).

TLC: R_f = 0.61 (Hex/EtOAc = 2:1) [UV].

IR (ATR): $\tilde{\nu}$ [cm^{-1}] = 2967 (m, sp^3-CH), 2928 (m, sp^3-CH), 1744 (vs, C=O), 1438 (s, sp^3-CH), 757 (vs, Ar-CH), 732 (vs, C-Cl).

^{1}H NMR (500 MHz, $CDCl_3$, 298 K): δ [ppm] = 8.23 (d, ^3J = 8.3 Hz, 0.4 H, H-11), 7.74 (d, ^4J = 2.2 Hz, 0.4 H, H-8), 7.72 – 7.69 (m, 1 H, H-7, H-8'), 7.62 – 7.55 (m, 0.6 H, H-7'), 7.41 – 7.34 (m, 2.6 H, H-5, H-5', H-6, H-6', H-10'), 7.30 – 7.23 (m, 2 H, H-4, H-4', H-9', H-10), 3.34 (ddd, ^3J = 12.6 Hz, ^3J = 5.9 Hz, ^4J = 1.1 Hz, 0.4 H, H-3a), 3.19 (ddd, ^3J = 12.7 Hz, ^3J = 6.4 Hz, ^4J = 1.2 Hz, 0.6 H, H-3a'), 2.92 (ddd, ^2J = 17.8 Hz, ^3J = 10.7 Hz, ^3J = 2.1 Hz, 0.6 H, *H*H-2'), 2.77 (ddd, ^2J = 19.1 Hz, ^3J = 8.5 Hz, ^3J = 0.9 Hz, 0.4 H, *H*H-2), 2.56 (ddd, ^2J = 19.1 Hz, ^3J = 9.9 Hz, ^3J = 8.8 Hz, 0.4 H, H*H*-2), 2.49 (ddd, ^2J = 17.8 Hz, ^3J = 9.2 Hz, ^3J = 7.7 Hz, 0.6 H, H*H*-2'), 2.46 (dddd, ^2J = 12.2 Hz, ^3J = 8.8 Hz, ^3J = 5.9 Hz, ^3J = 0.9 Hz, 0.4 H, *H*H-3), 2.38 (dddd, ^2J = 12.4 Hz, ^3J = 9.2 Hz, ^3J = 6.4 Hz, ^3J = 2.1 Hz, 0.6 H, *H*H-3'), 2.26 (*virt.* tdd, $^2J \approx {}^3J$ = 12.5 Hz, ^3J = 10.7 Hz, ^3J = 7.8 Hz, 0.6 H, H*H*-3'), 2.12 (dddd, ^3J = 12.6 Hz, ^2J = 12.2 Hz, ^3J = 9.9 Hz, ^3J = 8.5 Hz, 0.4 H, H*H*-3), 1.23 (s, 1.8 H, H_3C'), 0.86 (s, 1.2 H, H_3C).

^{13}C NMR (126 MHz, $CDCl_3$, 298 K): δ [ppm] = 216.5 (s, C-1), 210.0 (s, C-1'), 139.5 (s, C-11a), 137.4 (s, C-11a')*, 137.3 (s, C-7b')*, 136.8 (s, C-3b), 135.9 (s, C-9), 135.3 (s, C-3b'), 134.9 (s, C-7a'), 133.9 (s, C-7b)†, 133.5 (s, C-11'), 133.3 (s, C-7a)†, 130.8 (d, C-10'), 128.7 (d, C-5), 128.4 (d, C-9'), 128.3 (d, C-5'), 127.8 (d, C-10), 127.5 (d, C-6'), 127.5 (d, C-6), 125.4 (d, 2 C, C-11, C-7'), 125.1 (d, 2 C, C-4, C-8), 124.9 (d, C-4'), 124.6 (d, C-7), 123.2 (d, C-8'), 52.2 (s, C-11b'), 47.9 (s, C-11b), 46.3 (d, C-3a), 45.7 (d, C-3a'), 37.9 (t, C-2), 35.7 (t, C-2'), 20.0 (t, C-3), 18.2 (t, C-3'), 17.2 (q, CH_3), 12.7 (q, CH_3'). *,†Assignments are interchangeable.

HRMS (EI, 70 eV): calculated for $C_{18}H_{15}OCl$ [M]$^+$: 282.0806, found: 282.0805,
calculated for $C_{18}H_{15}O^{37}Cl$ [M]$^+$: 284.0776, found: 284.0784.

Reduction of 11o/11o'

According to GP C, **11o/11o'** (10.3 mg, 36.4 µmol, 1.00 eq., r.r. = 1:1.8) was reacted with $NaBH_4$ (7.00 mg, 159 µmol, 5.08 eq.) in 2 mL of MeOH/CH_2Cl_2 (1:1) for 1 h to yield alcohols **12o** (1.60 mg, 5.62 µmol, 15%, 88% *ee*) and **12o'** (2.90 mg, 10.2 µmol, 28%, d.r. = 2.5:1, 97% *ee*) as off-white solids after purification by automated column chromatography (silica, Hex/EtOAc = 95:5→70:30).

(1R,3aR,11bS)-9-Chloro-11b-methyl-2,3,3a,11b-tetrahydro-1H-cyclopenta[l]phenanthren-1-ol (12o)

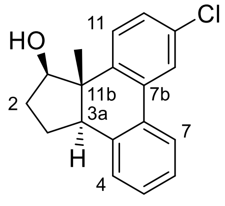

12o
$C_{18}H_{17}ClO$
M = 284.78 g/mol

TLC: R_f = 0.23 (Hex/EtOAc = 4:1) [UV].

M.p.: 65-70 °C.

IR (ATR): $\tilde{\nu}$ [cm^{-1}] = 3369 (m, broad, O-H), 2961 (m, sp^3-CH), 2920 (s, sp^3-CH), 1444 (s, sp^3-CH), 1018 (vs, C-O), 747 (vs, Ar-CH).

^{1}H NMR (500 MHz, $CDCl_3$, 298 K): δ [ppm] = 7.70 (d, ^4J = 2.2 Hz, 1 H, H-8), 7.70 – 7.67 (m, 1 H, H-7), 7.65 (d, ^3J = 8.2 Hz, 1 H, H-11), 7.37 – 7.29 (m, 2 H, H-5, H-6), 7.26 (dd, ^3J = 8.2 Hz, ^4J = 2.2 Hz, 1 H, H-10), 7.15 – 7.12 (m, 1 H, H-4), 4.50 (dd, ^3J = 9.2 Hz, ^3J = 7.7 Hz, 1 H, H-1), 2.94 (dd, ^3J = 12.1 Hz, ^3J = 7.6 Hz, 1 H, H-3a), 2.55 (*virt.* dtd, ^2J = 13.6 Hz, $^3J \approx {}^3J$ = 9.3 Hz, ^3J = 6.3 Hz, 1 H, *H*H-2), 2.06 (dddd, ^2J = 12.9 Hz, ^3J = 9.4 Hz, ^3J = 7.6 Hz, ^3J = 3.6 Hz, 1 H, *H*H-3), 1.98 (*virt.* qd, $^2J \approx {}^3J \approx {}^3J$ = 12.2 Hz, ^3J = 6.3 Hz, 1 H, H*H*-3), 1.76 (dddd, ^2J = 13.6 Hz, ^3J = 11.6 Hz, ^3J = 7.7 Hz, ^3J = 3.6 Hz, 1 H, H*H*-2), 0.77 (s, 3 H, CH_3).

^{13}C NMR (126 MHz, $CDCl_3$, 298 K): δ [ppm] = 144.7 (s, C-11a), 138.5 (s, C-3b), 135.8 (s, C-7b), 134.0 (s, C-7a), 132.7 (s, C-9), 128.4 (d, C-5), 127.9 (d, C-10), 126.8 (d, C-6), 125.9 (d, C-11), 125.4 (d, C-4), 124.7 (d, C-8), 124.2 (d, C-7), 78.0 (d, C-1), 46.3 (s, C-11b), 45.8 (d, C-3a), 33.0 (t, C-2), 21.2 (t, C-3), 13.0 (q, CH_3).

Chiral HPLC: 88% *ee* [Daicel Chiralcel, OJ-RH, 150x4.6, H_2O/MeCN = 80:20→0:100 (30 min), 1 mL/min, 215 nm, t_R = 18.65 min (minor), 19.32 min (major)].

Relevant correlations observed in NOESY to confirm relative configuration:

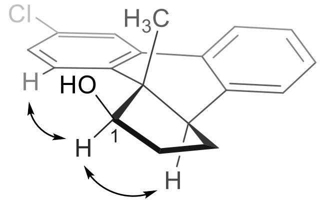

(1S,3aR,11bS)-11-Chloro-11b-methyl-2,3,3a,11b-tetrahydro-1H-cyclopenta[l]phenanthren-1-ol (12o')

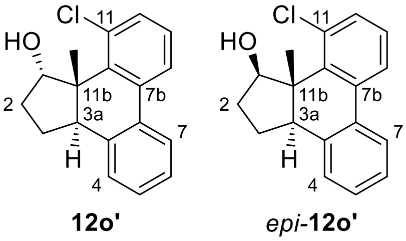

$C_{18}H_{17}ClO$
M = 284.78 g/mol

TLC: R_f = 0.47 (Hex/EtOAc = 4:1) [UV].

M.p.: 42-44 °C.

IR (ATR): $\tilde{\nu}$ [cm^{-1}] = 3448 (m, broad, O-H), 2935 (s, sp^3-CH), 2874 (m, sp^3-CH), 1439 (s, sp^3-CH), 1023 (s, C-O), 755 (vs, Ar-CH).

^{1}H NMR (500 MHz, $CDCl_3$, 298 K): δ [ppm] = 7.75 (dd, ^3J = 7.7 Hz, ^4J = 1.4 Hz, 0.7 H, H-8), 7.69 (dd, ^3J = 7.7 Hz, ^4J = 1.4 Hz, 0.3 H, *epi*-H-8), 7.65 – 7.59 (m, 1 H, H-7, *epi*-H-7), 7.36 – 7.29 (m, 3 H, H-5, *epi*-H-5, H-6, *epi*-H-6, H-10, *epi*-H-10), 7.26 (t, ^3J = 7.7 Hz, 0.7 H, H-9), 7.26 – 7.20 (m, 1 H, H-4, *epi*-H-9), 7.20 – 7.15 (m, 0.3 H, *epi*-H-4), 5.04 (d, ^3J = 6.0 Hz, 0.7 H, H-1), 4.93 (dd, ^3J = 10.0 Hz, ^3J = 4.3 Hz, 0.3 H, *epi*-H-1), 3.64 (dd, ^3J = 12.0 Hz, ^3J = 7.8 Hz, 0.7 H, H-3a), 3.15 (dd, ^3J = 11.7 Hz, ^3J = 6.8 Hz, 0.3 H, *epi*-H-3a), 2.56 – 2.46 (m, 0.3 H, *epi-H*H-2), 2.40 (dddd, ^2J = 14.6 Hz, ^3J = 11.5 Hz, ^3J = 6.0 Hz, ^3J = 3.1 Hz, 0.7 H, *H*H-2), 2.31 – 2.21 (m, 1 H, *H*H-3, *epi-H*H-3), 2.12 – 2.05 (m, 0.3 H, *epi*-H*H*-3), 2.01 (ddd, ^2J = 14.6 Hz, ^3J = 9.7 Hz, ^3J = 6.3 Hz, 0.7 H, H*H*-2), 2.01 – 1.94 (m, 0.3 H, *epi*-H*H*-2), 1.84 (*virt.* tdd, $^2J \approx {}^3J$ = 12.2 Hz, ^3J = 11.5 Hz, ^3J = 6.3 Hz, 0.7 H, H*H*-3), 0.78 (s, 0.9 H, *epi*-CH_3), 0.76 (s, 2.1 H, CH_3).

^{13}C NMR (126 MHz, $CDCl_3$, 298 K): δ [ppm] = 140.7 (s, C-11a), 138.8 (s, C-3b)*, 138.7 (s, C-7b)*, 134.3 (s, C-7a), 130.9 (s, C-11), 130.1 (d, C-10), 128.5 (d, C-5), 128.2 (d, C-9), 126.7 (d, C-6), 125.9 (d, C-4), 124.9 (d, C-7), 124.4 (d, C-8), 77.1 (d, C-1), 53.0 (s, C-11b), 44.2 (d, C-3a), 31.4 (t, C-2), 21.3 (t, C-3), 15.3 (q, CH_3). *Assignments are interchangeable.

The quantity of *epi*-**12o'** was too small for discernable signals in ^{13}C NMR spectroscopy.

Chiral HPLC: 97% *ee* [Daicel Chiralcel, OD-RH, 150x4.6, H_2O/MeCN = 80:20→0:100 (30 min), 1 mL/min, 215 nm, t_R = 22.30 min (*epi*-minor), 23.18 min (*epi*-major), 25.91 (minor), 27.43 (major)].

Relevant correlations observed in NOESY to confirm relative configuration:

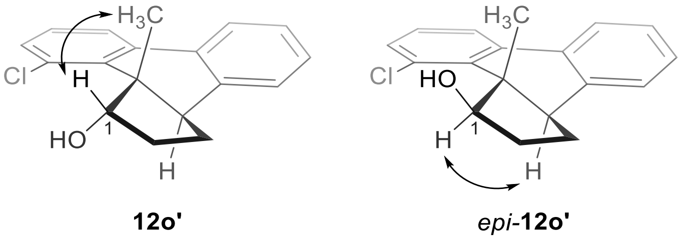

(3a*R*,11b*S*)-9-Methoxy-11b-methyl-2,3,3a,11b-tetrahydro-1*H*-cyclopenta[*l*]phenanthren-1-one (11p)

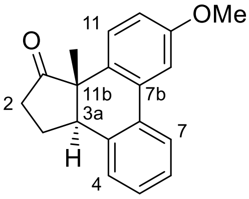

11p
$C_{19}H_{18}O_2$
M = 278.35 g/mol

According to GP B, **9p** (13.9 mg, 50.0 µmol, 1.00 eq.) was irradiated at λ = 405 nm for 210 min in the presence of **10b**·$AlBr_3$ (1.25 µmol, 2.50 mol%). Automated column chromatography (silica, Hex/EtOAc = 96:4→70:30) afforded **11p** (6.00 mg, 21.6 µmol, 43%, 95% *ee*) as a yellow solid, along with regioisomer **11p'** (*vide infra*).

TLC: R_f = 0.69 (Hex/EtOAc = 2:1) [UV].

M.p.: 58 °C.

IR (ATR): $\tilde{\nu}$ [cm^{-1}] = 2956 (m, sp^3-CH), 2926 (s, sp^3-CH), 1735 (vs, C=O), 1448 (s, sp^3-CH), 1217 (vs, C-O), 1008 (vs), 751 (vs, Ar-CH).

^{1}H NMR (500 MHz, $CDCl_3$, 298 K): δ [ppm] = 8.20 (d, ^{3} = 8.5 Hz, 1 H, H-11), 7.76 – 7.69 (m, 1 H, H-7), 7.40 – 7.34 (m, 2 H, H-5, H-6), 7.33 (d, ^4J = 2.7 Hz, 1 H, H-8), 7.29 – 7.23 (m, 1 H, H-4), 6.88 (dd, ^3J = 8.5 Hz, ^4J = 2.7 Hz, 1 H, H-10), 3.87 (s, 3 H, OCH_3), 3.35 (ddd, ^3J = 12.7 Hz, ^3J = 5.9 Hz, ^4J = 1.0 Hz, 1 H, H-3a), 2.75 (ddd, ^2J = 19.1 Hz, ^3J = 8.6 Hz, ^3J = 1.0 Hz, 1 H, *H*H-2), 2.55 (ddd, ^2J = 19.1 Hz, ^3J = 9.9 Hz, ^3J = 8.8 Hz, 1 H, H*H*-2), 2.44 (dddd, ^2J = 12.2 Hz, ^3J = 8.8 Hz, ^3J = 5.9 Hz, ^3J = 1.0 Hz, 1 H, *H*H-3), 2.11 (dddd, ^3J = 12.7 Hz, ^2J = 12.2 Hz, ^3J = 9.9 Hz, ^3J = 8.6 Hz, 1 H, H*H*-3), 0.86 (s, 3 H, CH_3).

^{13}C NMR (101 MHz, $CDCl_3$, 298 K): δ [ppm] = 217.1 (s, C-1), 158.9 (s, C-9), 137.0 (s, C-3b), 135.4 (s, C-7b), 135.0 (s, C-7a), 133.9 (s, C-11a), 128.2 (d, C-5), 127.3 (d, C-6), 125.0 (d, C-11)*, 125.0 (d, C-4)*, 124.5 (d, C-7), 112.8 (d, C-10), 111.1 (d, C-8), 55.5 (q, OCH_3), 48.0 (s, C-11b), 46.9 (d, C-3a), 38.0 (t, C-2), 20.2 (t, C-3), 17.5 (q, CH_3).

*Assignments are interchangeable.

Chiral HPLC: 95% *ee* [Daicel Chiralcel, OJ-RH, 150x4.6, H_2O/MeCN = 80:20→0:100 (30 min), 1 mL/min, 215 nm, t_R = 18.48 min (minor), 20.92 min (major)].

HRMS (EI, 70 eV): calculated for $C_{19}H_{18}O_2$ $[M]^+$: 278.1301, found: 278.1297,
calculated for $C_{18}{}^{13}CH_{18}O_2$ $[M]^+$: 279.1335, found: 279.1331.

(3a*R*,11b*S*)-11-Methoxy-11b-methyl-2,3,3a,11b-tetrahydro-1*H*-yclopenta[*l*]phenanthren-1-one (11p')

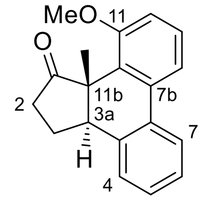

11p'
$C_{19}H_{18}O_2$
M = 278.35 g/mol

11p' (4.80 mg, 17.2 µmol, 34%, 99% *ee*) was isolated as a yellow solid alongside **11p** (*vide supra*).

TLC: R_f = 0.63 (Hex/EtOAc = 2:1) [UV].

M.p.: 130 °C.

IR (ATR): $\tilde{\nu}$ [cm^{-1}] = 2926 (m, sp^3-CH), 2837 (w, sp^3-CH), 1748 (vs, C=O), 1463 (s, sp^3-CH), 1256 (vs, C-O), 1019 (s), 757 (vs, Ar-CH).

^{1}H NMR (500 MHz, $CDCl_3$, 298 K): δ [ppm] = 7.68 – 7.62 (m, 1 H, H-7), 7.44 (dd, ^3J = 7.8 Hz, ^4J = 1.1 Hz, 1 H, H-8), 7.37 – 7.30 (m, 2 H, H-5, H-6), 7.31 (*virt.* t, $^3J \approx {}^3J$ = 8.0 Hz, 1 H, H-9), 7.25 – 7.22 (m, 1 H, H-4), 6.97 (dd, ^3J = 8.2 Hz, ^4J = 1.1 Hz, 1 H, H-10), 3.91 (s, 3 H, OCH_3), 3.25 (ddd, ^3J = 12.8 Hz, ^3J = 6.1 Hz, ^4J = 1.3 Hz, 1 H, H-3a), 2.84 (ddd, ^2J = 17.9 Hz, ^3J = 9.8 Hz, ^3J = 1.6 Hz, 1 H, *H*H-2), 2.49 (*virt.* dt, ^2J = 17.9 Hz, $^3J \approx {}^3J$ = 8.9 Hz, 1 H, H*H*-2), 2.38 (dddd, ^2J = 12.2 Hz, ^3J = 9.1 Hz, ^3J = 6.1 Hz, ^3J = 1.6 Hz, 1 H, *H*H-3), 2.18 (dddd, ^3J = 12.8 Hz, ^2J = 12.2 Hz, ^3J = 9.8 Hz, ^3J = 8.8 Hz, 1 H, H*H*-3), 1.06 (s, 3 H, CH_3).

^{13}C NMR (101 MHz, $CDCl_3$, 298 K): δ [ppm] = 211.3 (s, C-1), 157.9 (s, C-11), 136.4 (s, C-7b), 136.1 (s, C-3b), 135.5 (s, C-7a), 129.0 (s, C-11a), 128.3 (d, C-9), 127.9 (d, C-5), 127.2 (d, C-6), 125.2 (d, C-7), 124.7 (d, C-4), 117.5 (d, C-8), 112.6 (d, C-10), 56.2 (q, OCH_3), 51.3 (s, C-11b), 46.5 (d, C-3a), 37.2 (t, C-2), 19.0 (t, C-3), 14.1 (q, CH_3).

Chiral HPLC: 99% *ee* [Daicel Chiralcel, OD-RH, 150x4.6, H_2O/MeCN = 80:20→0:100 (30 min), 1 mL/min, 215 nm, t_R = 22.02 min (minor), 24.64 min (major)].

HRMS (EI, 70 eV): calculated for $C_{19}H_{18}O_2$ [M]$^+$: 278.1301, found: 278.1296,
calculated for $C_{18}{}^{13}CH_{18}O_2$ [M]$^+$: 279.1335, found: 279.1335.

Synthesis of Esters

Scale-Up Photocyclization

(3a*R*,11b*S*)-11b-Methyl-2,3,3a,11b-tetrahydro-1*H*-cyclopenta[*l*]phenanthren-1-one (11a)

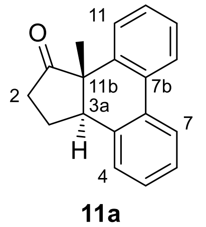

11a

$C_{18}H_{16}O$
M = 248.33 g/mol

According to GP B, **9a** (99.3 mg, 0.40 mmol, 1.00 eq.) was irradiated at λ = 405 nm for 20 h in the presence of **10b**·$AlBr_3$ (20.0 µmol, 5.00 mol%). Automated column chromatography (silica, Hex/EtOAc = 100:0→85:15) afforded **11a** (37.7 mg, 152 µmol, 38%, 91% *ee*) as a colorless solid.

We accredited the poor yield to the prolonged reaction time of 20 h required for full conversion at this scale, where degredation of the catalyst may occur. As a more powerful LED at this wavelength was not available to us, an experiment at a shorter wavelength was conducted to accelerate the reaction. This, however, led to a slight decrease in ee, *presumably due to spectral overlap with the uncoordinated substrate.*

According to GP B, **9a** (124 mg, 0.50 mmol, 1.00 eq.) was irradiated at λ = 368 nm for 16 h in the presence of **10b**·$AlBr_3$ (25.0 µmol, 5.00 mol%). Automated column chromatography (silica, Hex/EtOAc = 100:0→85:15) afforded **11a** (108 mg, 436 µmol, 87%, 85% *ee*) as a colorless solid.

(1*R*,3a*R*,11b*S*)-11b-Methyl-2,3,3a,11b-tetrahydro-1*H*-cyclopenta[*l*]phenanthren-1-ol (12a)

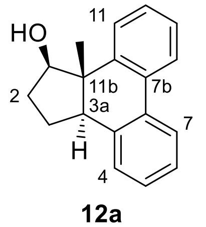

12a

$C_{18}H_{18}O$
M = 250.34 g/mol

According to GP C, **11a** (35.0 mg, 14.1 µmol, 1.00 eq., 91% *ee*) was reacted with $NaBH_4$ (21.3 mg, 564 µmol, 15.5 eq.) in 7 mL of $MeOH/CH_2Cl_2$ (1:1) for 1 h to yield alcohol **12a** as a colorless solid, which was used without further purification.

TLC: R_f = 0.33 (Hex/EtOAc = 4:1) [UV].

M.p.: 84 °C.

IR (ATR): $\tilde{\nu}$ [cm^{-1}] = 3392 (s, broad, O-H), 2973 (m, sp^3-CH), 2876 (m, sp^3-CH), 1483 (s, sp^3-CH), 1439 (s, sp^3-CH), 1060 (s, C-O), 999 (s), 760 (vs, Ar-CH), 734 (vs, Ar-CH).

^{1}H NMR (500 MHz, $CDCl_3$, 298 K): δ [ppm] = 7.77 – 7.72 (m, 2 H, H-7, H-8), 7.72 – 7.68 (m, 1 H, H-11), 7.35 – 7.28 (m, 4 H, H-5, H-6, H-9, H-10), 7.13 (*virt.* dt, ^{3}J = 7.5 Hz, ^{4}J = ^{4}J = 1.5 Hz, 1 H, H-4), 4.55 (dd, ^{3}J = 9.2 Hz, ^{3}J = 7.7 Hz, 1 H, H-1), 2.98 (dd, ^{3}J = 12.1 Hz, ^{3}J = 7.6 Hz, 1 H, H-3a), 2.55 (*virt.* dtd, ^{2}J = 13.7 Hz, $^{3}J \approx {}^{3}J$ = 9.4 Hz, ^{3}J = 6.4 Hz, 1 H, *H*H-2), 2.06 (dddd, ^{2}J = 13.0 Hz, ^{3}J = 9.6 Hz, ^{3}J = 7.6 Hz, ^{3}J = 3.7 Hz, 1 H, *H*H-3), 1.99 (*virt.* qd, $^{2}J \approx {}^{3}J \approx {}^{3}J$ = 12.2 Hz, ^{3}J = 6.4 Hz, 1 H, H*H*-3), 1.77 (dddd, ^{2}J = 13.7 Hz, ^{3}J = 11.6 Hz, ^{3}J = 7.7 Hz, ^{3}J = 3.7 Hz, 1 H, H*H*-2), 0.80 (s, 3 H, CH_3).

^{13}C NMR (101 MHz, $CDCl_3$, 298 K): δ [ppm] = 146.4 (s, C-11a), 138.5 (s, C-3b), 135.1 (s, C-7a), 134.0 (s, C-7b), 128.2 (d, C-5), 127.8 (d, C-10), 127.0 (d, C-9), 126.7 (d, C-6), 125.2 (d, C-4), 124.6 (d, C-8), 124.3 (d, C-11), 124.1 (d, C-7), 78.2 (d, C-1), 46.6 (s, C-11b), 45.9 (d, C-3a), 33.1 (t, C-2), 21.2 (t, C-3), 13.1 (q, CH_3).

(1*R*,3a*R*,11b*S*)-11b-Methyl-2,3,3a,11b-tetrahydro-1*H*-cyclopenta[*l*]phenanthren-1-yl 4-bromobenzoate (14)

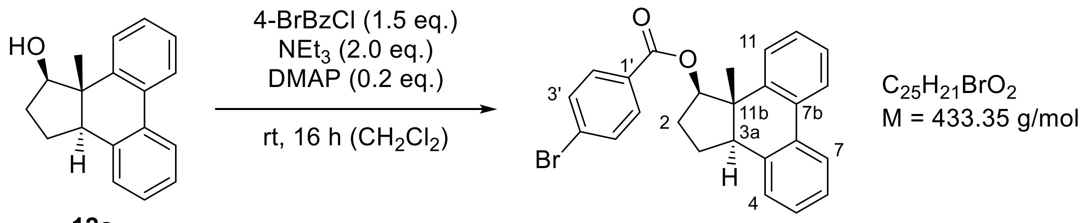

12a **14**

4-Bromobenzoyl chloride (46.1 mg, 210 µmol, 1.50 eq.) was added to a solution of alcohol **12a** (35.1 mg, 140 µmol, 1.00 eq.), triethylamine (39.0 µL, 28.3 mg, 280 µmol, 2.00 eq.) and 4-dimethylaminopyridine (3.42 mg, 28.0 µmol, 0.20 eq.) in 7 mL of CH_2Cl_2 (20 mM) at room temperature. After stirring for 16 h, the reaction was quenched by addition of 1 mL of water, the phases were separated, the organic phase was extracted with CH_2Cl_2 (3 × 2 mL), and the combined organic phases were washed with brine (4 mL), dried over Na_2SO_4 and concentrated *in vacuo*. Purification by automated column chromatography (silica, Hex/EtOAc = 90:10→60:40) afforded **14** (11.1 mg, 25.6 µmol, 18%, 93% *ee*) as a colorless solid.

TLC: R_f = 0.74 (Hex/EtOAc = 9:1) [UV].

M.p.: 158-160 °C.

IR (ATR): $\tilde{\nu}$ [cm^{-1}] = 2973 (m, sp^3-CH), 2926 (m, sp^3-CH), 2855 (m, sp^3-CH), 1715 (vs, C=O), 1271 (vs, C-O), 1118 (vs, C-O), 1103 (vs, C-O), 757 (vs, Ar-CH), 732 (vs, Ar-CH).

^{1}H NMR (500 MHz, $CDCl_3$, 298 K): δ [ppm] = 8.02 – 7.96 (m, 2 H, H-2', H-6'), 7.79 – 7.74 (m, 2 H, H-7, H-8), 7.66 – 7.59 (m, 2 H, H-3', H-5'), 7.39 – 7.29 (m, 3 H, H-5, H-6, H-9), 7.26 – 7.19 (m, 2 H, H-10, H-11), 7.18 (virt. dt, ^3J = 7.4 Hz, ^4J = ^4J = 1.5 Hz, 1 H, H-4), 5.58 (dd, ^3J = 9.3 Hz, ^3J = 7.1 Hz, 1 H, H-1), 3.13 (dd, ^3J = 12.2 Hz, ^3J = 7.4 Hz, 1 H, H-3a), 2.87 (*virt.* dtd, ^2J = 14.0 Hz, $^3J \approx {}^3J$ = 9.4 Hz, ^3J = 6.8 Hz, 1 H, *H*H-2), 2.19 (dddd, ^2J = 12.5 Hz, ^3J = 9.5 Hz, ^3J = 7.4 Hz, ^3J = 3.2 Hz, 1 H, *H*H-3), 2.08 (*virt.* qd, $^2J \approx {}^3J \approx {}^3J$ = 12.1 Hz, ^3J = 6.8 Hz, 1 H, H*H*-3), 1.91 (dddd, ^2J = 14.0 Hz, ^3J = 11.6 Hz, ^3J = 7.1 Hz, ^3J = 3.2 Hz, 1 H, H*H*-2), 1.02 (s, 3 H, CH_3).

^{13}C NMR (126 MHz, $CDCl_3$, 298 K): δ [ppm] = 165.7 (s, COO), 145.3 (s, C-11a), 137.7 (s, C-3b), 135.0 (s, C-7a), 134.0 (s, C-7b), 132.0 (d, 2 C, C-3', C-5'), 131.3 (d, 2 C, C-2', C-6'), 129.5 (s, C-1'), 128.4 (s, C-4'), 128.3 (d, C-10), 128.0 (d, C-5), 127.3 (d, C-9), 126.9 (d, C-6), 125.5 (d, C-4), 124.9 (d, C-7), 124.1 (d, C-8), 123.7 (d, C-11), 80.6 (d, C-1), 46.7 (s, C-11b), 45.7 (d, C-3a), 29.9 (t, C-2), 21.6 (t, C-3), 14.7 (q, CH_3).

HRMS (EI, 70 eV): calculated for $C_{25}H_{21}O_2Br$ $[M]^+$: 432.0719, found: 432.0714,
calculated for $C_{24}{}^{13}CH_{21}O_2Br$ $[M]^+$: 433.0753, found: 433.0738.

Chiral HPLC: 93% *ee* [Daicel Chiralcel, OD-RH, 150x4.6, H_2O/MeCN = 80:20→0:100 (30 min), 1 mL/min, 215 nm, t_R = 29.32 min (minor), 30.24 min (major)].

Representative Procedure: Synthesis of *Mosher* Esters

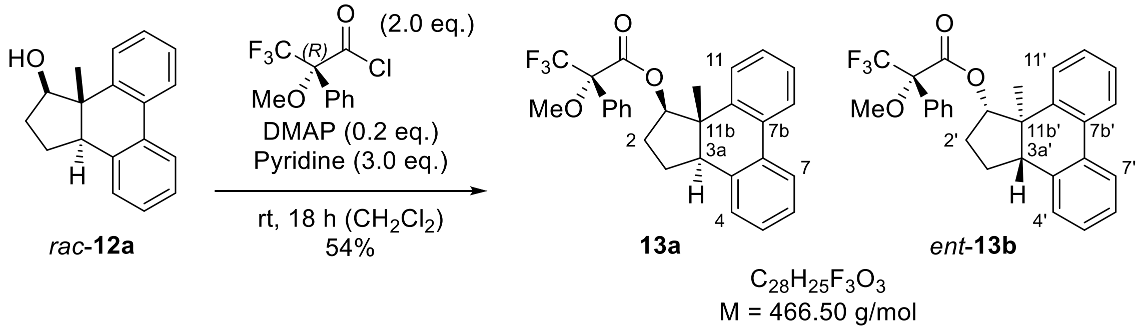

(*R*)-3,3,3-trifluoro-2-methoxy-2-phenylpropanoyl chloride (*Mosher* acid chloride) (4.48 µL, 6.05 mg, 24.0 µmol, 2.00 eq.) was added to a solution of *rac*-**12a** (3.00 mg, 12.0 µmol, 1.00 eq.), pyridine (2.90 µL, 2.84 mg, 36.0 µmol, 3.00 eq.) and 4-dimethylaminopyridine (293 µg, 2.40 µmol, 0.20 eq.) in 0.25 mL of CH_2Cl_2 at room temperature. After stirring for 18 h, the reaction was quenched by addition of 0.5 mL water, the phases were separated, the organic phase was extracted with CH_2Cl_2 (3 × 0.5 mL), and combined organic phases were washed with brine (1 mL), dried over Na_2SO_4 and concentrated *in vacuo.* Purification by automated

column chromatography (silica, Hex/EtOAc = 100:0→60:40) afforded a mixture of diastereomers **13a** and *ent*-**13b** (4.10 mg, 8.79 µmol, 73%, d.r. = 2.4:1) as a pale yellow oil.

TLC: R_f = 0.66 (Hex/EtOAc = 9:1) [UV].

IR (ATR): $\tilde{\nu}$ [cm^{-1}] = 3064 (m, Ar-CH), 2946 (s, sp^3-CH), 2851 (m, sp^3-CH), 1743 (vs, C=O), 1258 (vs, C-O), 1167 (vs, C-F), 1027 (vs, C-O), 732 (vs, Ar-CH).

^{1}H NMR (500 MHz, $CDCl_3$, 298 K): δ [ppm] = 7.76 – 7.72 (m, 0.6 H, H-7', H-8'), 7.72 – 7.69 (m, 1.4 H, H-7, H-8), 7.69 – 7.61 (m, 2 H, H-*o*-Ph, H-*o*-Ph'), 7.49 – 7.44 (m, 3 H, H-*m*-Ph, H-*m*-Ph', H-*p*-Ph, H-*p*-Ph'), 7.36 – 7.29 (m, 2.3 H, H-5, H-5', H-6, H-6', H-9'), 7.27 (*virt.* td, $^3J \approx {}^3J$ = 7.6 Hz, ^4J = 1.3 Hz, 0.7 H, H-9), 7.18 – 7.10 (m, 1.3 H, H-4, H-4', H-10'), 7.04 (*virt.* td, $^3J \approx {}^3J$ = 7.6 Hz, ^4J = 1.3 Hz, 0.7 H, H-10), 6.99 (dd, ^3J = 7.5 Hz, ^4J = 1.3 Hz, 0.3 H, H-11'), 6.77 (dd, ^3J = 7.6 Hz, ^4J = 1.3 Hz, 0.7 H, H-11), 5.52 (dd, ^3J = 9.3 Hz, ^3J = 7.0 Hz, 0.3 H, H-1'), 5.44 (dd, ^3J = 9.2 Hz, ^3J = 7.2 Hz, 0.7 H, H-1), 3.67 (s, 2.1 H, OCH_3), 3.58 (s, 0.9 H, OCH_3'), 3.09 – 3.01 (m, 1 H, H-3a, H-3a'), 2.94 – 2.80 (m, 1 H, *H*H-2, *H*H-2'), 2.21 – 2.11 (m, 1 H, *H*H-3, *H*H-3'), 2.11 – 1.97 (m, 1 H, H*H*-3, H*H*-3'), 1.90 (dddd, ^2J = 14.6 Hz, ^3J = 11.7 Hz, ^3J = 7.2 Hz, ^3J = 3.1 Hz, 0.7 H, H*H*-2), 1.81 (dddd, ^2J = 14.5 Hz, ^3J = 11.7 Hz, ^3J = 7.0 Hz, ^3J = 3.1 Hz, 0.3 H, H*H*-2'), 0.81 (s, 2.1 H, CH_3), 0.80 (s, 0.9 H, CH_3').

^{13}C NMR (126 MHz, $CDCl_3$, 298 K): δ [ppm] = 167.9 (s, COO'), 166.5 (s, COO), 144.8 (s, C-11a'), 144.6 (s, C-11a), 137.4 (s, C-3b'), 137.3 (s, C-3b), 134.9 (s, C-7a), 134.8 (s, C-7a'), 133.9 (s, C-7b'), 133.8 (s, C-7b), 132.5 (s, C-*i*-Ph'), 132.5 (s, C-*i*-Ph), 129.9 (d, C-*p*-Ph'), 129.8 (d, C-*p*-Ph), 128.8 (d, 2 C, C-*m*-Ph'), 128.7 (d, 2 C, C-*m*-Ph), 128.1 (d, C-10'), 128.1 (d, C-10), 128.0 (d, C-5'), 128.0 (d, C-5), 127.8 (d, 2 C, C-*o*-Ph'), 127.3 (d, 2 C, C-*o*-Ph), 127.3 (d, C-9'), 127.2 (d, C-9), 127.0 (d, 2 C, C-6, C-6'), 125.4 (d, C-4'), 125.4 (d, C-4), 124.8 (d, C-8), 124.8 (d, C-8'), 124.1 (d, C-7), 124.1 (d, C-7'), 123.7 (d, C-11), 123.6 (d, C-11'), 84.4 (q, $^2J_{C\text{-}F}$ = 27.4 Hz, *C*(CF_3)), 82.4 (d, C-1), 82.3 (d, C-1'), 55.9 (q, OCH_3), 55.5 (q, OCH_3'), 46.8 (s, C-11b'), 46.7 (s, C-11b), 45.6 (d, C-3a'), 45.4 (d, C-3a), 29.5 (t, C-2), 29.5 (t, C-2'), 21.5 (t, C-3), 21.5 (t, C-3'), 14.3 (q, CH_3'), 14.2 (q, CH_3).

Due to the low sample quantity, signals of *C*(CF_3)', CF_3 and CF_3' were not observable by ^{13}C NMR spectroscopy.

HRMS (EI, 70 eV): calculated for $C_{28}H_{25}O_3F_3$ $[M]^+$: 466.1750, found: 466.1749.

Determination of Absolute Configuration by *Mosher* Ester Analysis

Mosher esters were prepared as outlined above, starting from enantioenriched product **11a** (91% *ee*), with both enantiomers of the *Mosher* acid chloride. The following products were obtained. Their absolute configuration was established as outlined below.

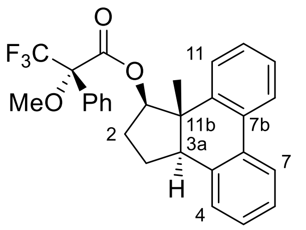

13a

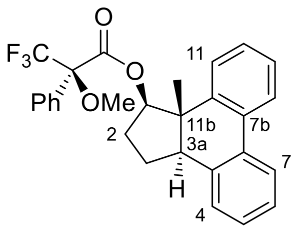

13b

Determination of absolute configuration by the method of *Mosher* is based on the observation that diastereomeric α-methoxy-α-trifluoromethylphenylacetic acid (MTPA) esters (*Mosher* esters) display different chemical shifts in ^{1}H NMR spectroscopy. This is due to the fact that the esters preferentially adopt an *s-trans* conformation of the O-CO bond, while the CF_3 group and the proton of the secondary alcohol moiety are arranged *syn*-coplanar with the carbonyl group.[5] As shown in Figure S1, this arrangement places the phenyl group of the MTPA moiety in proximity to aromatic protons H-11 and H-10 of **13a** (Figure S1, top), where its anisotropic effect imposes a shielding effect, and thus an upfield shift, of the respective ^{1}H NMR signals. In contrast to this, in the diastereomer **13b** (Figure S1, bottom), the phenyl group is arranged in the opposite direction, where it imposes an upfield shift on the methylene protons (most notably H-2) of the cyclopentanone ring instead.

In the recorded ^{1}H NMR spectra, the strong upfield shift of H-10 and H-11 signals of **13a** (Figure S1, right, blue) relative to the respective signals of **13b** (Figure S1, right, green), as well as the upfield shift of the H-2 signal of **13b** (Figure S1, left, dark green) relative to the respective signal of **13a** (Figure S1, left, dark blue), allows to derive the absolute configuration of alcohol **12a**, and thus **11a**.

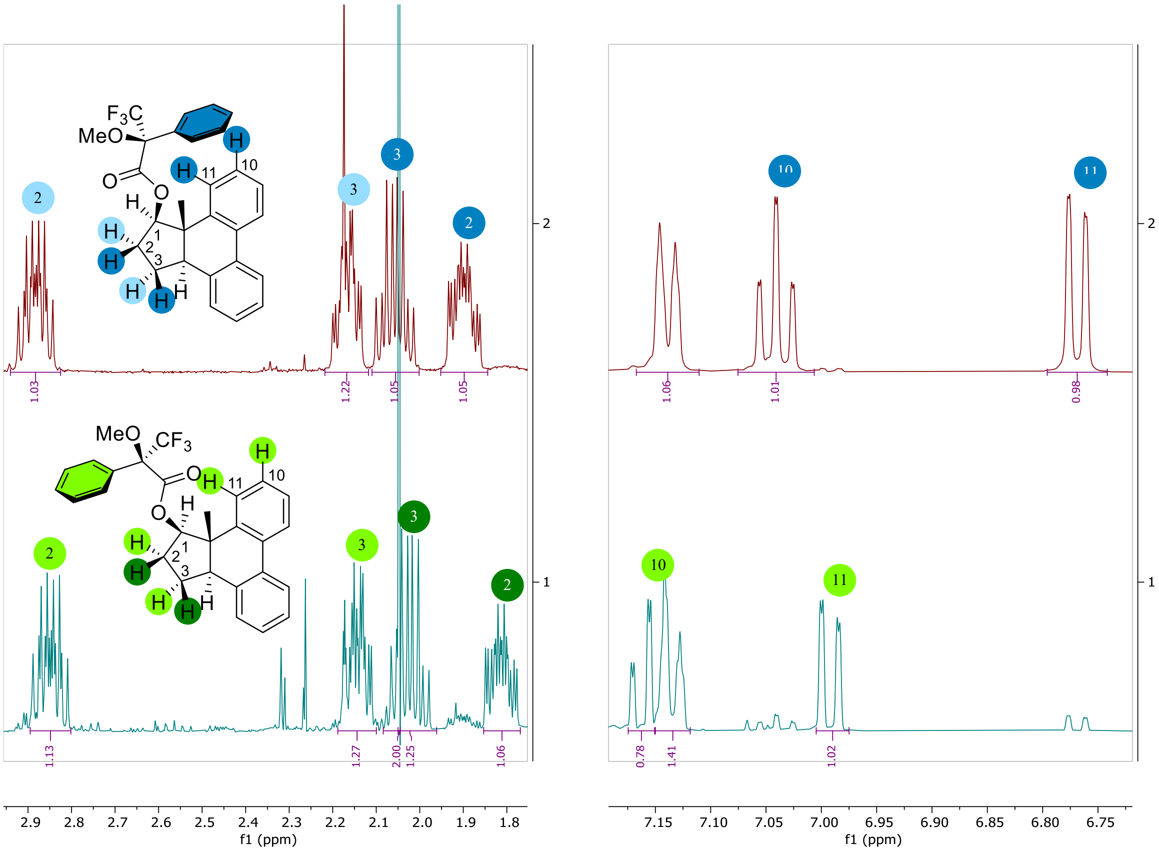

Figure S1: Excerpts of the ^{1}H NMR spectra of enantioenriched *Mosher* esters **13a** (top) and **13b** (bottom), prepared from chiral alcohol **12a** and (*R*)-*Mosher* chloride and (*S*)-*Mosher* chloride, respectively.

Determination of Absolute Configuration by SC-XRD

Crystals submitted to SC-XRD analysis were prepared from the enantioenriched ester **14** (93% *ee*) by suspending in hexanes, then slowly adding acetone until all material dissolved. The solution was then filtered through a syringe filter and left to slowly evaporate at room temperature, leaving colorless crystals. The following structure was determined by SC-XRD analysis (see detailed report below):

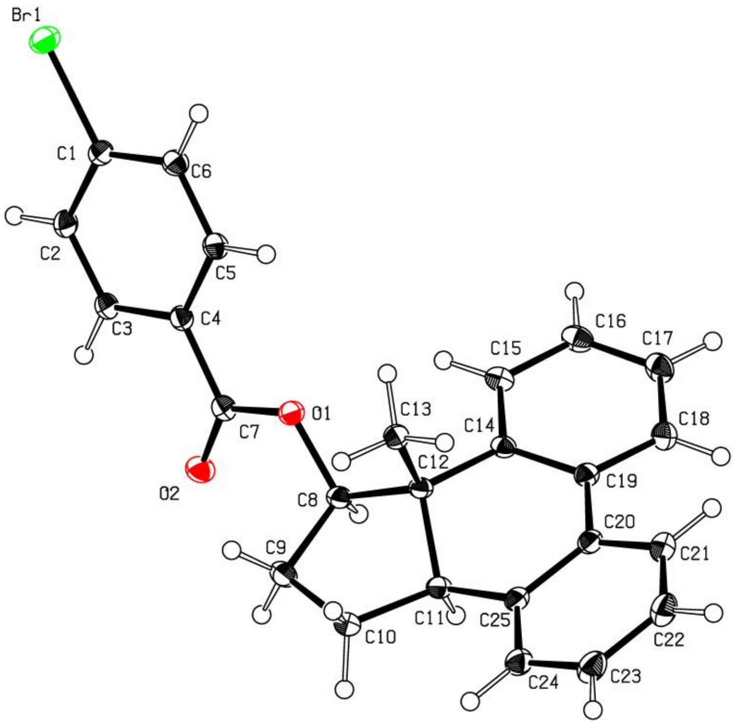

Chiral HPLC analysis of a sample of the submitted crystals revealed an *ee* of 99%, which was further corroborated by evaluating the Flack parameter, determined from anomalous-dispersion effects observed in diffraction measurements on the single-crystal.

Screening of Chiral Lewis Acids and Reaction Conditions

Table S1: Extended screening of 1,3,2-oxazaborolidine aryl groups and reaction conditions.

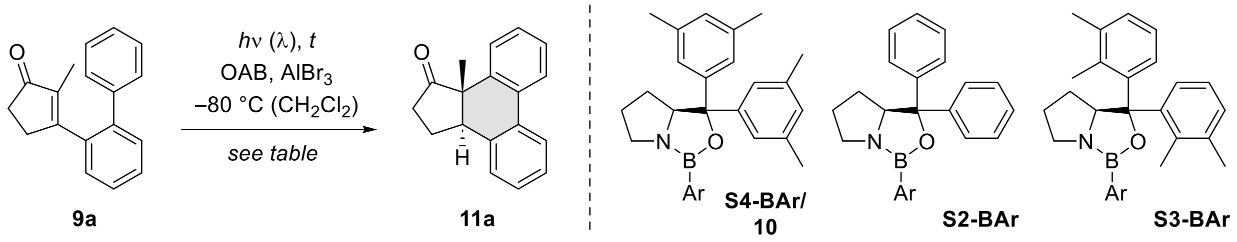

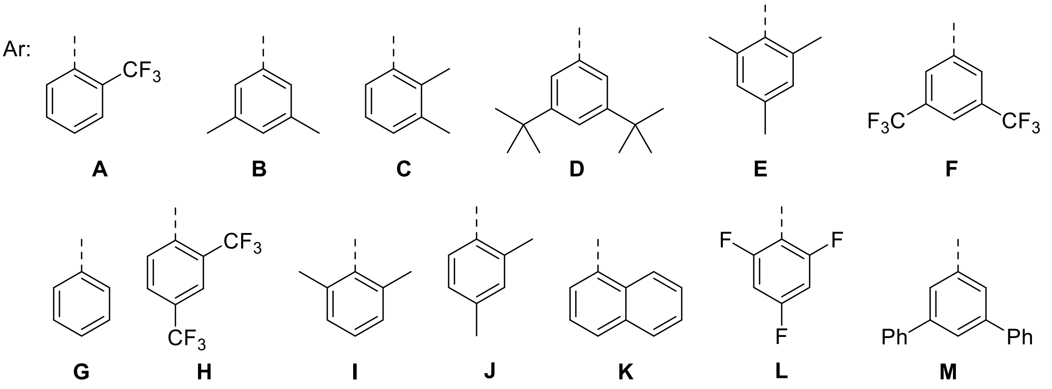

Entry	Prolinol	Ar	OAB (mol%)	$AlBr_3$ (mol%)	t (min)	λ (nm)	Yield (%)	*ee* (%)
1	**S2**	I	30	25	45	398	73	76
2	**S3**	A	30	25	45	398	81	84
3	**S3**	C	30	25	45	398	81	86
4	**S3**	G	30	25	45	398	52	83
5	**S3**	H	30	25	45	398	66	83
6	**S3**	I	30	25	60	398	84	75
7	**S3**	J	30	25	45	398	56	83
8	**S3**	K	30	25	45	398	65	79
9	**S3**	L	30	25	30	398	63	83
10	**10**	B	20	10	45	398	60	82
11	**10**	B	20	10	45	405	55	84
12	**10**	M	5	2.5	150	405	71	90
13	**10**	A	30	25	45	398	71	81
14	**10**	B	30	25	45	398	69	87
15	**10**	B	30	15	45	398	72	90
16	**10**	B	50	25	45	398	56	87
17	**10**	B	10	5	180	405	81	96
18	**10**	B	5	2.5	210	405	75	93
19	**10**	C	5	2.5	240	405	68	87
20	**10**	D	5	2.5	210	405	78	91
21	**10**	E	5	2.5	210	405	52	32
22	**10**	F	5	2.5	285	405	57	18

Deuterium Labeling Experiments

Table S2: Outcome of photocyclization reaction of deuterated substrate **9a**-d_5 under different reaction conditions. Ratios **11a**-d_5/**11a**-d_4 were determined from the relative integral of the corresponding proton signal in the ^{1}H NMR spectra.

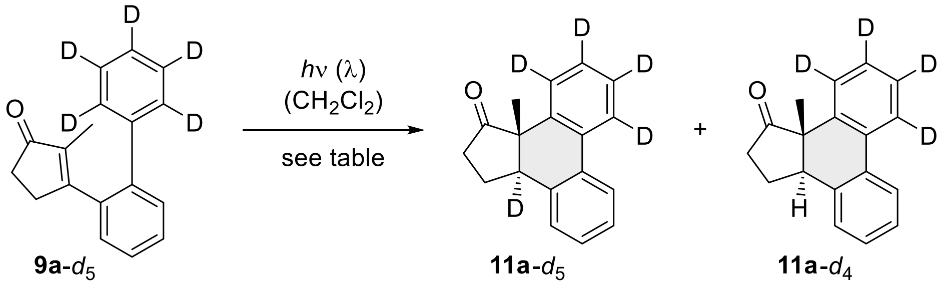

Entry	10b (mol%)	$AlBr_3$ (mol%)	λ (nm)	T (°C)	t (min)	reisol. 9a-d_5	Yield (%)	11a-d_5/11a-d_4
1	-	-	350	30	80	-	87	100/0
2	-	-	350	-80	80	84	6	54/46
3	-	10	366	30	25	-	89	81/19
4	-	5	368	-80	70	-	46	63/37
5	10	5	368	-80	90	-	30	24/76
6	10	5	405	-80	90	67	6	26/74

In line with previously reported results[2], the uncatalyzed [6π] photocyclization of deuterated substrate **9a**-d_5 at room temperature leads to complete deuterium incorporation (product **11a**-d_5), as is evident by the lack of the corresponding proton signal in the ^{1}H NMR spectrum. This supports the mechanistic understanding that formation of the final product from the cyclization intermediate proceeds by a suprafacial 1,5-hydride shift. However, at -80 °C and otherwise identical conditions (Table S2, entry 2), conversion becomes very slow, and only 6% yield is obtained within the same reaction time. Furthermore, this results in a ratio of approx. 50/50 of **11a**-d_5/**11a**-d_4, based on a relative NMR integral of 0.43. While the reaction is not sluggish in the presence of $AlBr_3$ at room temperature (entry 3), the Lewis acid seems to promote a partial incorporation of hydrogen instead of deuterium, resulting in a ratio of 81/19 of **11a**-d_5/**11a**-d_4. At -80 °C (entry 4), this ratio shifts even further and, though conversion is complete, the yield is significantly reduced. Interestingly, in the presence of the chiral Lewis acid, the ratio of **11a**-d_5/**11a**-d_4 inverts to favor hydrogen incorporation (entry 5). While this ratio does not seem to depend on wavelength, conversion slows down significantly at 405 nm over 368 nm (entry 6).

UV/VIS Spectra of Selected Substrates with and without Lewis Acid

CH_2Cl_2 was used as solvent for all samples.

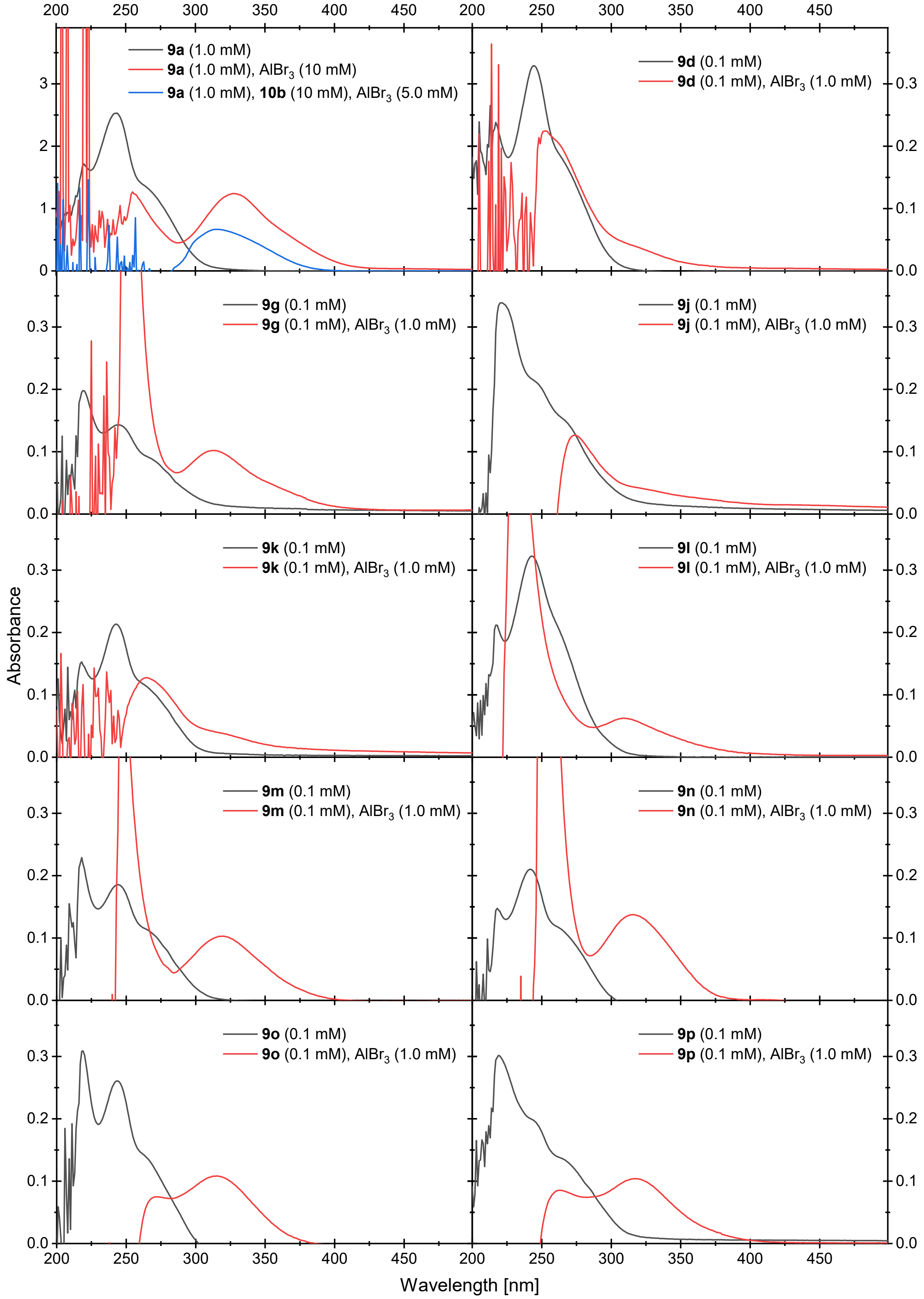

Chiral HPLC Traces

(3a*R*,11b*S*)-11b-methyl-2,3,3a,11b-tetrahydro-1*H*-cyclopenta[*l*]phenanthren-1-one (11a)

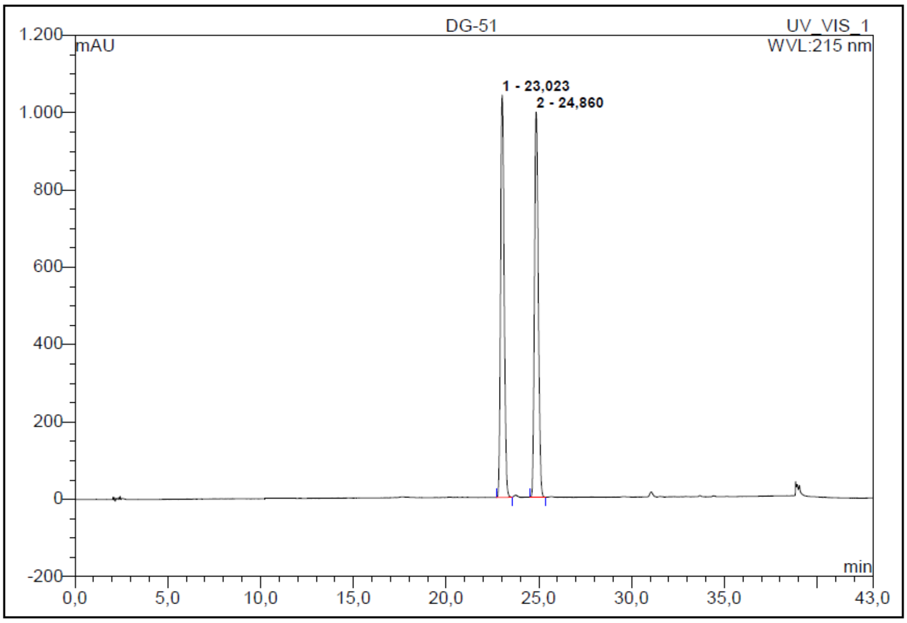

No.	Ret.Time min	Peak Name	Height mAU	Area mAU*min	Rel.Area %	Amount	Type
1	23,02	n.a.	1040,229	232,512	49,39	n.a.	BMB
2	24,86	n.a.	996,161	238,227	50,61	n.a.	BMB
Total:			2036,390	470,739	100,00	0,000	

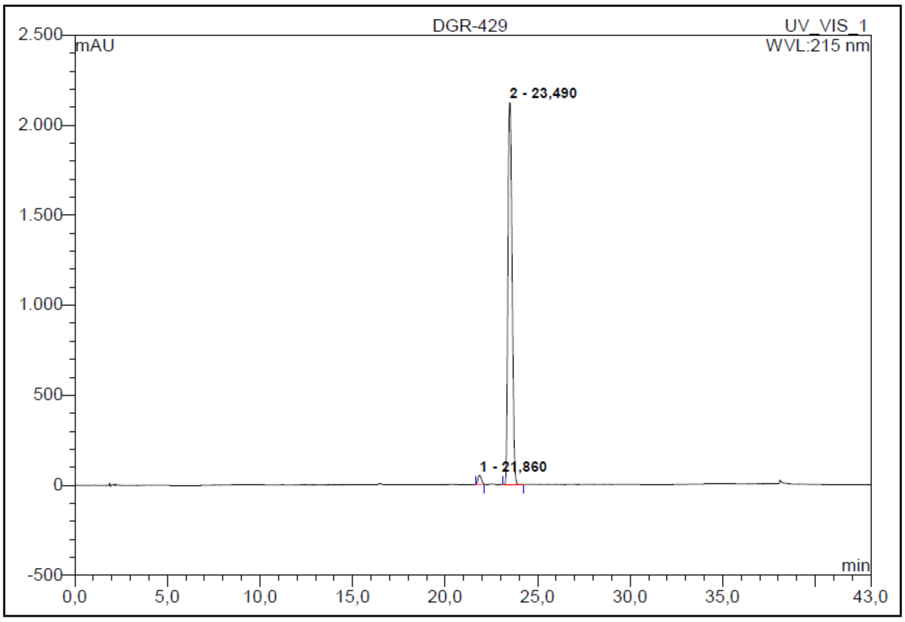

No.	Ret.Time min	Peak Name	Height mAU	Area mAU*min	Rel.Area %	Amount	Type
1	21,86	n.a.	51,266	11,103	1,96	n.a.	BMB*
2	23,49	n.a.	2121,785	554,420	98,04	n.a.	BMB*
Total:			2173,051	565,524	100,00	0,000	

(1*R*,3a*R*,11b*S*)-10,11b-dimethyl-2,3,3a,11b-tetrahydro-1*H*-cyclopenta[*l*]phenanthren-1-ol (12b)

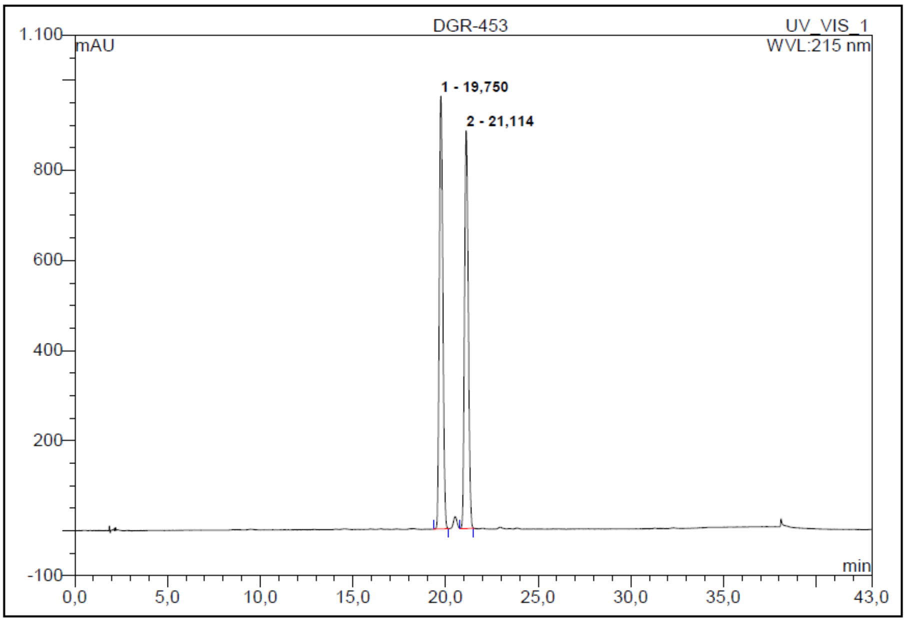

No.	Ret.Time min	Peak Name	Height mAU	Area mAU*min	Rel.Area %	Amount	Type
1	19,75	n.a.	960,776	214,367	50,76	n.a.	BMB
2	21,11	n.a.	883,458	207,987	49,24	n.a.	BMB
Total:			1844,235	422,354	100,00	0,000	

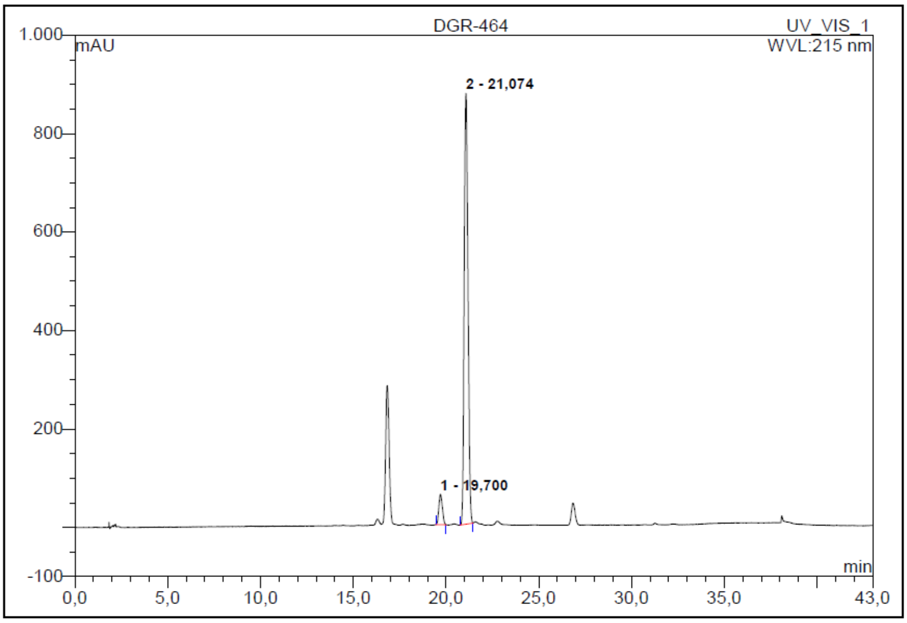

No.	Ret.Time min	Peak Name	Height mAU	Area mAU*min	Rel.Area %	Amount	Type
1	19,70	n.a.	61,742	13,576	6,21	n.a.	BMB
2	21,07	n.a.	875,608	204,966	93,79	n.a.	BMB
Total:			937,350	218,541	100,00	0,000	

(3a*R*,11b*S*)-10-fluoro-11b-methyl-2,3,3a,11b-tetrahydro-1*H*-cyclopenta[*l*]phenanthren-1-one (11c)

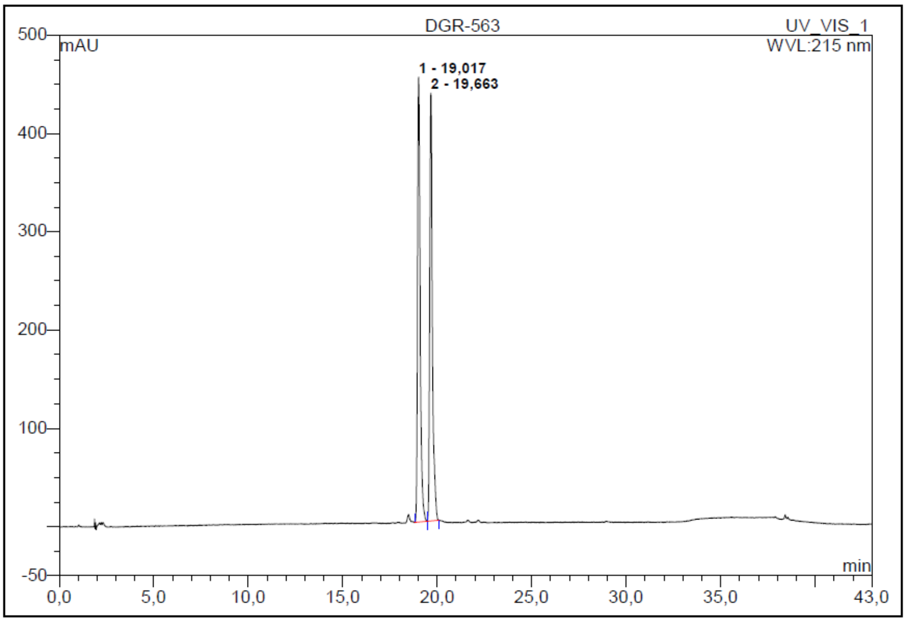

No.	Ret.Time min	Peak Name	Height mAU	Area mAU*min	Rel.Area %	Amount	Type
1	19,02	n.a.	452,852	72,367	50,49	n.a.	BM
2	19,66	n.a.	435,473	70,969	49,51	n.a.	MB
Total:			888,325	143,336	100,00	0,000	

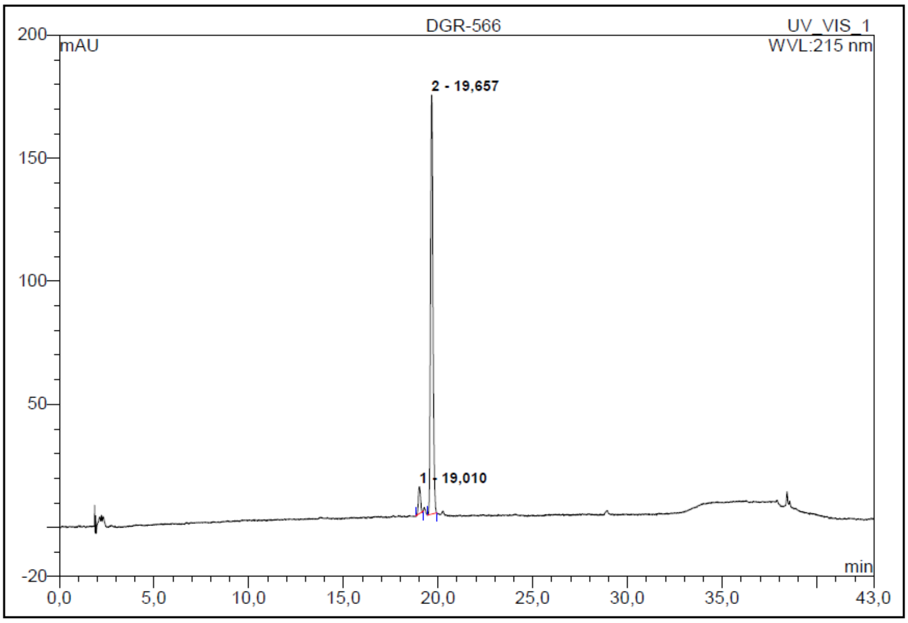

No.	Ret.Time min	Peak Name	Height mAU	Area mAU*min	Rel.Area %	Amount	Type
1	19,01	n.a.	10,952	1,400	5,29	n.a.	BMB*
2	19,66	n.a.	170,323	25,044	94,71	n.a.	BMB
Total:			181,275	26,444	100,00	0,000	

(3a*R*,11b*S*)-11b-methyl-10-(trifluoromethyl)-2,3,3a,11b-tetrahydro-1*H*-cyclopenta[*l*]phenanthren-1-one (11d)

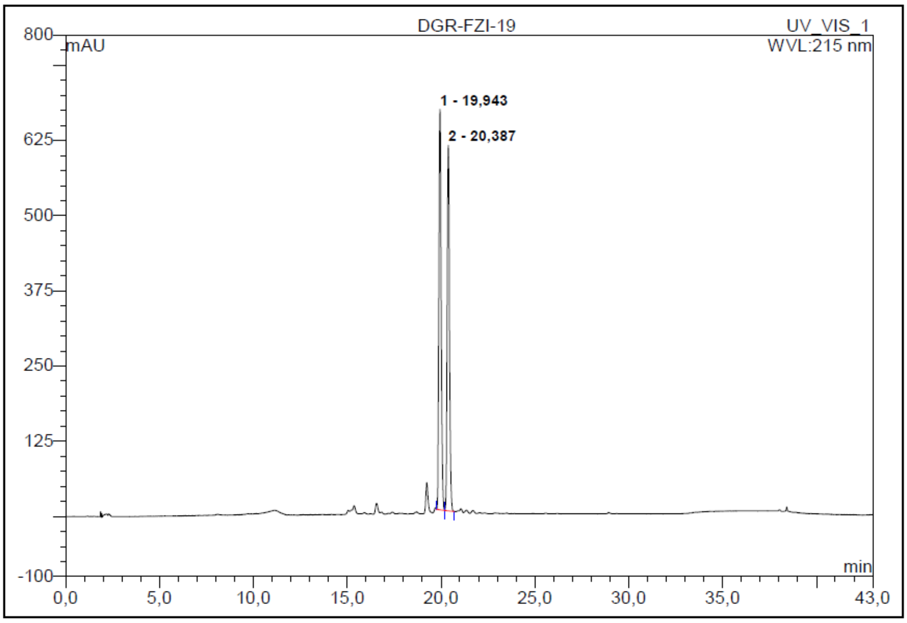

No.	Ret.Time min	Peak Name	Height mAU	Area mAU*min	Rel.Area %	Amount	Type
1	19,94	n.a.	666,014	93,729	51,64	n.a.	BMB
2	20,39	n.a.	607,881	87,781	48,36	n.a.	BMB
Total:			1273,895	181,510	100,00	0,000	

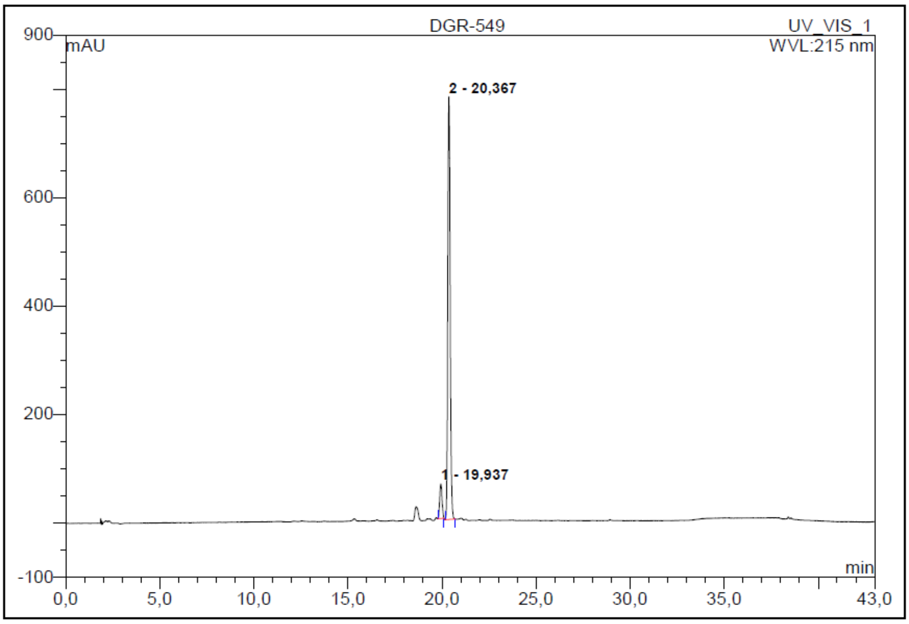

No.	Ret.Time min	Peak Name	Height mAU	Area mAU*min	Rel.Area %	Amount	Type
1	19,94	n.a.	63,515	9,233	7,45	n.a.	BMB*
2	20,37	n.a.	779,598	114,657	92,55	n.a.	BMB
Total:			843,113	123,890	100,00	0,000	

(3a*R*,11b*S*)-10-(*tert*-butyl)-11b-methyl-2,3,3a,11b-tetrahydro-1*H*-cyclopenta[*l*]phenanthren-1-one (11e)

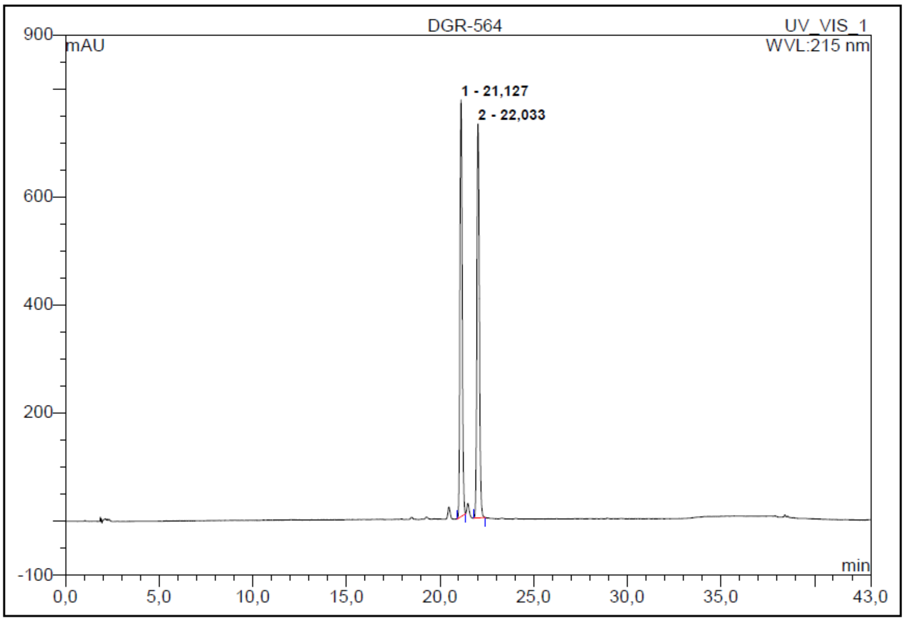

No.	Ret.Time min	Peak Name	Height mAU	Area mAU*min	Rel.Area %	Amount	Type
1	21,13	n.a.	771,360	108,484	49,38	n.a.	BMB
2	22,03	n.a.	729,550	111,224	50,62	n.a.	BMB
Total:			1500,910	219,708	100,00	0,000	

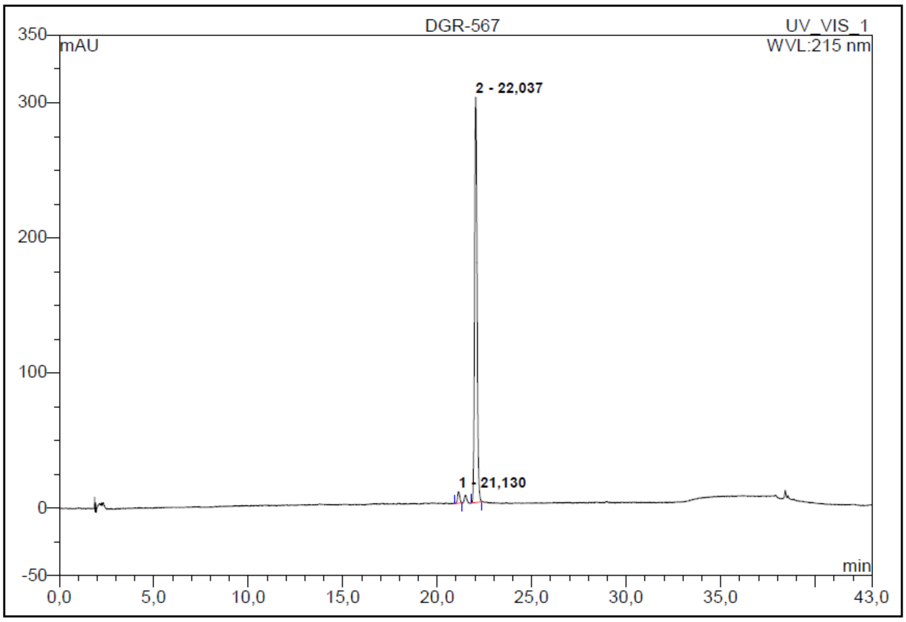

No.	Ret.Time min	Peak Name	Height mAU	Area mAU*min	Rel.Area %	Amount	Type
1	21,13	n.a.	8,509	1,178	2,55	n.a.	BMB*
2	22,04	n.a.	299,958	45,096	97,45	n.a.	BMB
Total:			308,467	46,274	100,00	0,000	

(3a*R*,11b*S*)-10-chloro-11b-methyl-2,3,3a,11b-tetrahydro-1*H*-cyclopenta[*l*]phenanthren-1-one (11f)

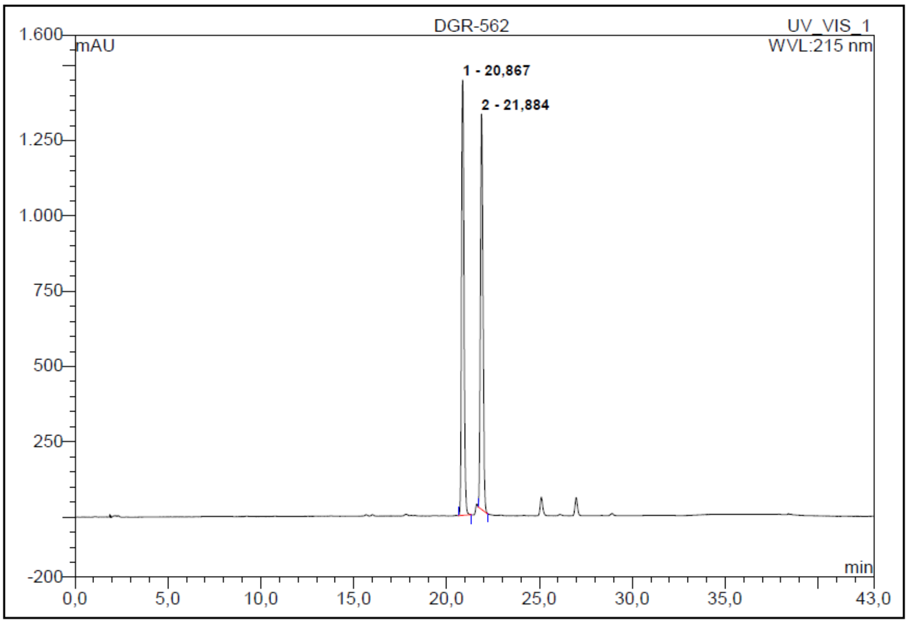

No.	Ret.Time min	Peak Name	Height mAU	Area mAU*min	Rel.Area %	Amount	Type
1	20,87	n.a.	1446,301	223,646	51,30	n.a.	BMB
2	21,88	n.a.	1313,550	212,336	48,70	n.a.	BMB
Total:			2759,851	435,982	100,00	0,000	

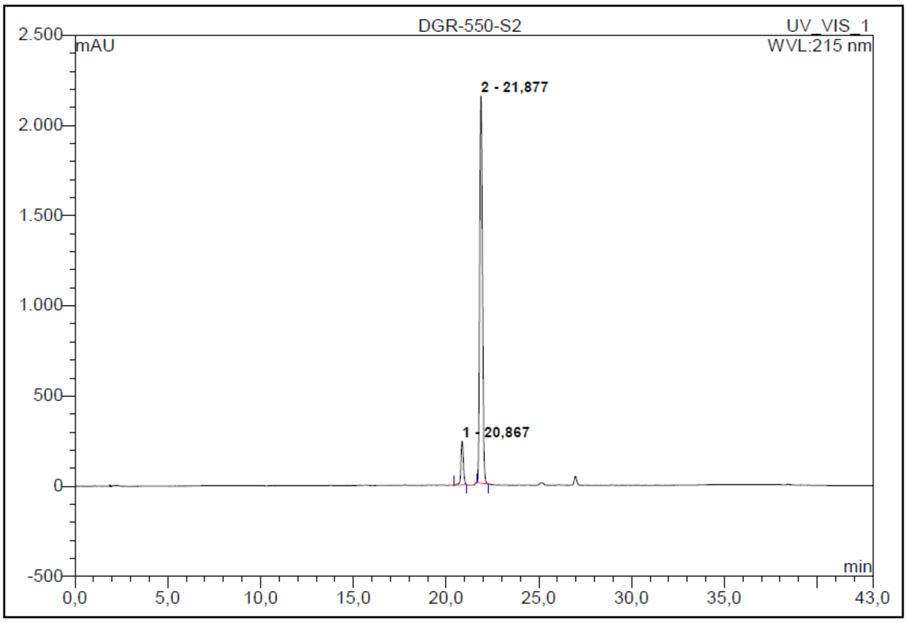

No.	Ret.Time min	Peak Name	Height mAU	Area mAU*min	Rel.Area %	Amount	Type
1	20,87	n.a.	242,518	37,604	9,16	n.a.	BMB
2	21,88	n.a.	2147,086	373,019	90,84	n.a.	BMB
Total:			2389,604	410,622	100,00	0,000	

(3a*R*,11b*S*)-9,11,11b-trimethyl-2,3,3a,11b-tetrahydro-1*H*-cyclopenta[*l*]phenanthren-1-one (11g)

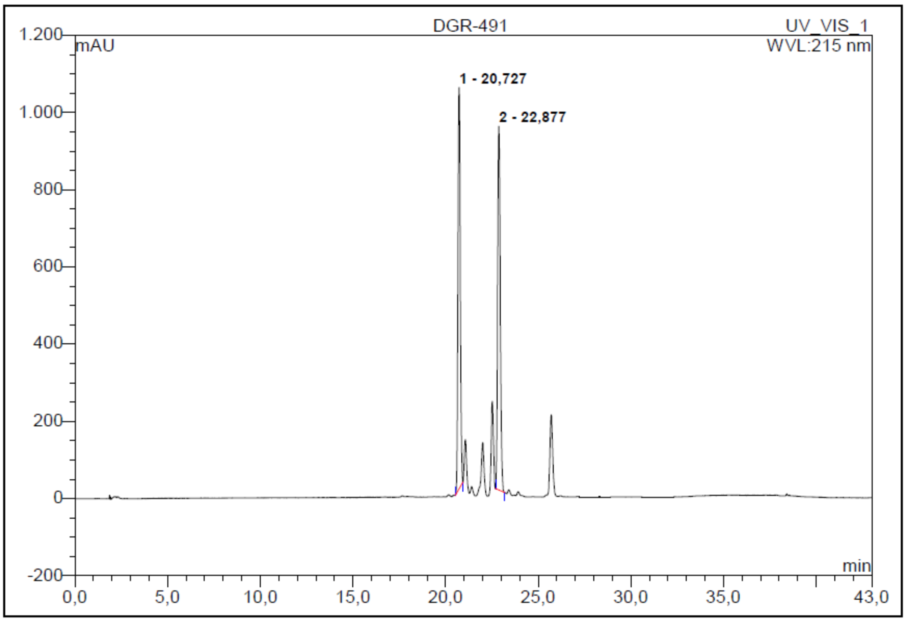

No.	Ret.Time min	Peak Name	Height mAU	Area mAU*min	Rel.Area %	Amount	Type
1	20,73	n.a.	1040,318	152,393	49,69	n.a.	BMB*
2	22,88	n.a.	942,046	154,300	50,31	n.a.	BMB*
Total:			1982,364	306,693	100,00	0,000	

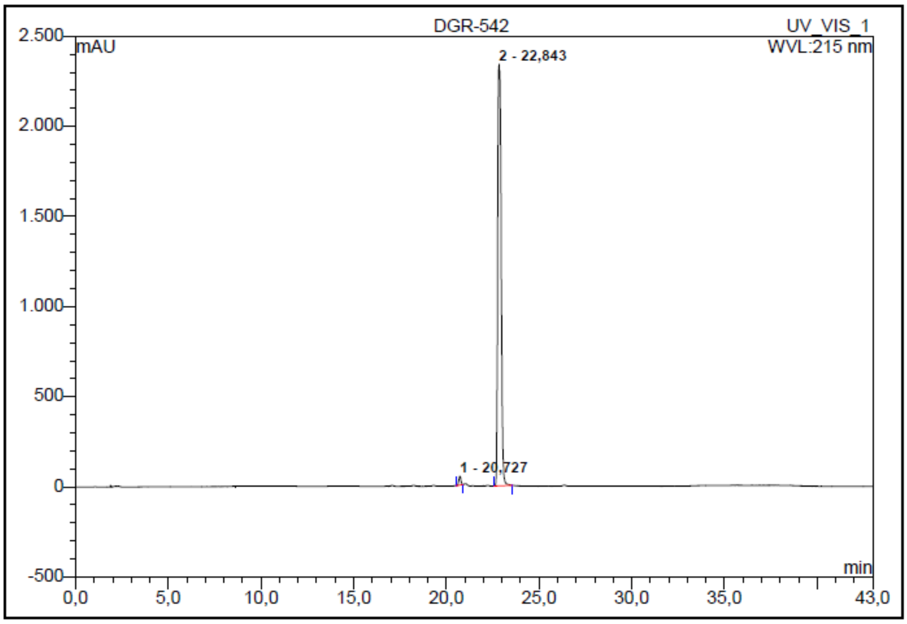

No.	Ret.Time min	Peak Name	Height mAU	Area mAU*min	Rel.Area %	Amount	Type
1	20,73	n.a.	49,685	6,565	1,29	n.a.	BMB*
2	22,84	n.a.	2338,593	501,146	98,71	n.a.	BMB
Total:			2388,278	507,711	100,00	0,000	

(3a*R*,11b*S*)-9,11-difluoro-11b-methyl-2,3,3a,11b-tetrahydro-1*H*-cyclopenta[*l*]phenanthren-1-one (11h)

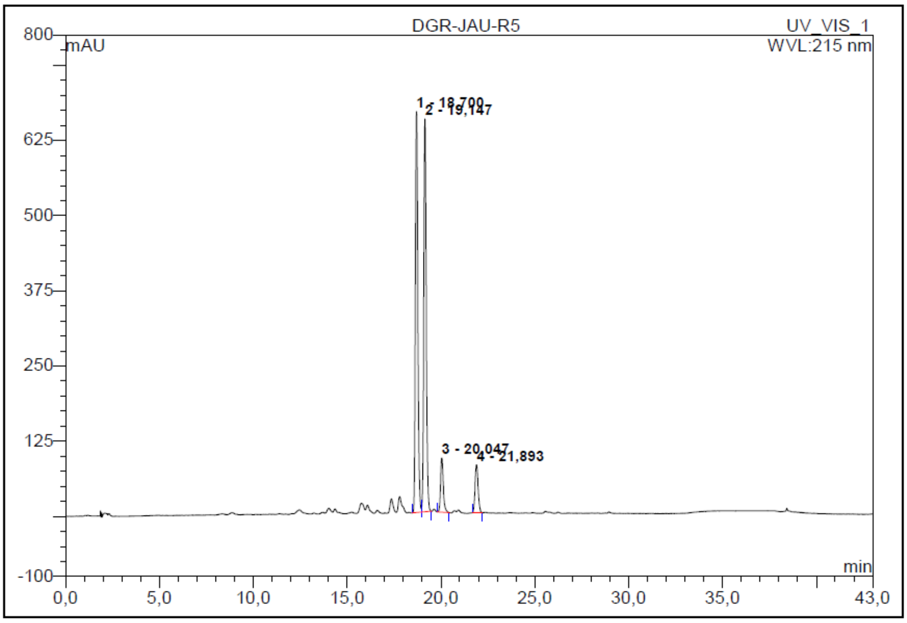

No.	Ret.Time min	Peak Name	Height mAU	Area mAU*min	Rel.Area %	Amount	Type
1	18,70	n.a.	666,369	100,526	43,28	n.a.	BM
2	19,15	n.a.	653,558	101,458	43,68	n.a.	MB
3	20,05	n.a.	90,144	15,109	6,51	n.a.	BMB
4	21,89	n.a.	80,084	15,167	6,53	n.a.	BMB
Total:			1490,156	232,261	100,00	0,000	

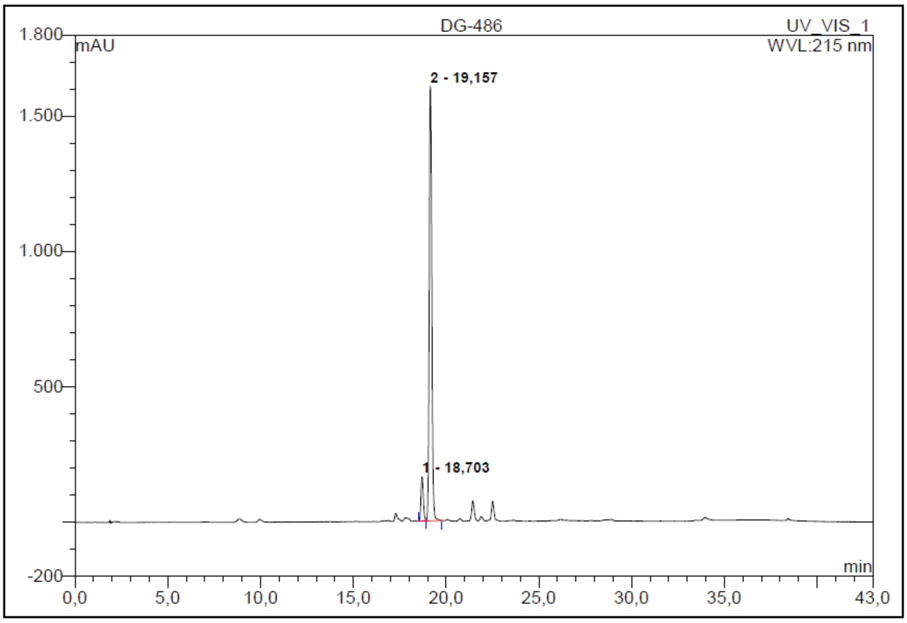

No.	Ret.Time min	Peak Name	Height mAU	Area mAU*min	Rel.Area %	Amount	Type
1	18,70	n.a.	162,029	23,947	8,41	n.a.	Ru
2	19,16	n.a.	1605,806	260,679	91,59	n.a.	BMB
Total:			1767,835	284,626	100,00	0,000	

(3a*R*,11b*S*)-9,11-dichloro-11b-methyl-2,3,3a,11b-tetrahydro-1*H*-cyclopenta[*l*]phen-anthren-1-one (11i)

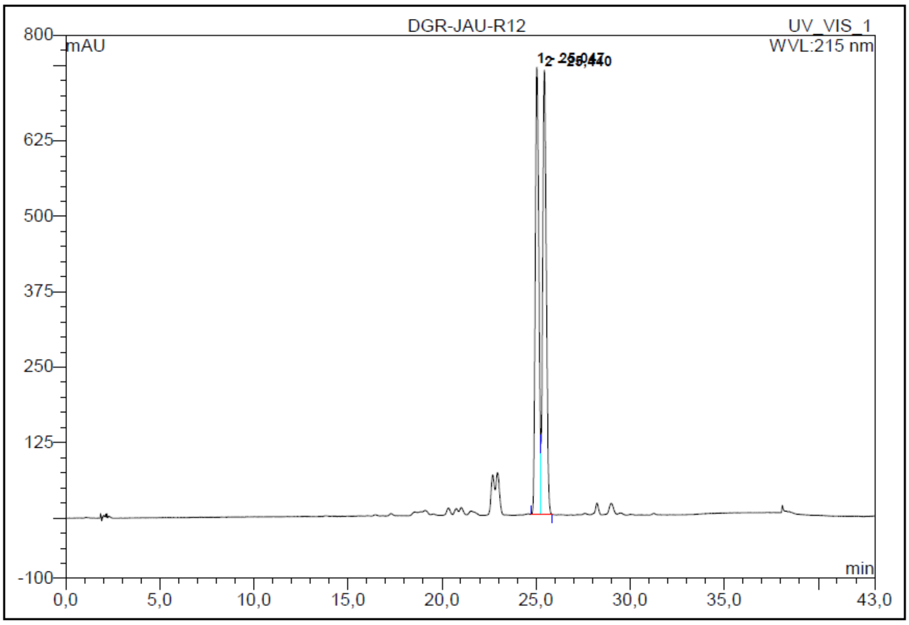

No.	Ret.Time min	Peak Name	Height mAU	Area mAU*min	Rel.Area %	Amount	Type
1	25,05	n.a.	741,145	163,216	49,49	n.a.	BM
2	25,44	n.a.	736,418	166,567	50,51	n.a.	MB
Total:			1477,563	329,783	100,00	0,000	

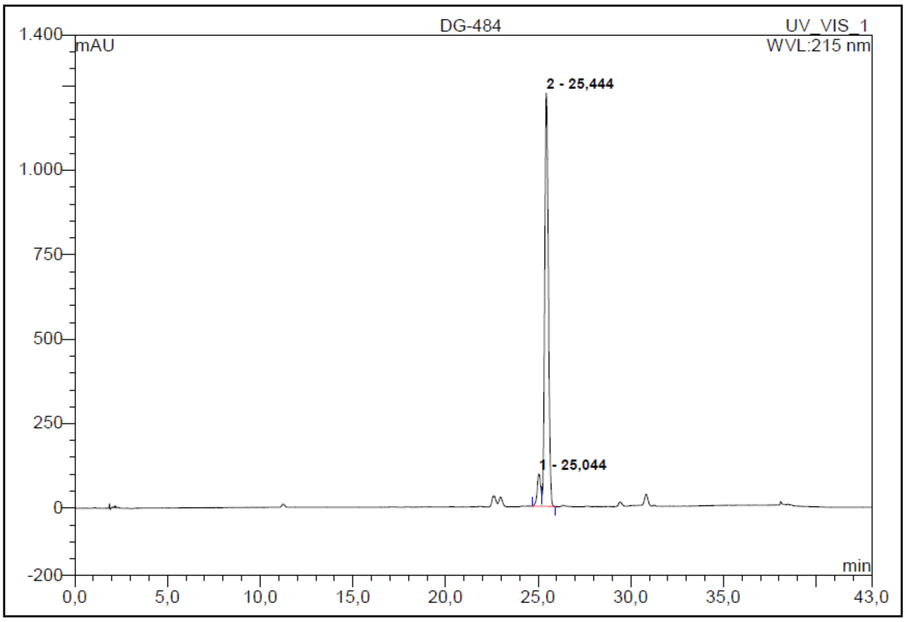

No.	Ret.Time min	Peak Name	Height mAU	Area mAU*min	Rel.Area %	Amount	Type
1	25,04	n.a.	95,574	21,501	7,18	n.a.	BM
2	25,44	n.a.	1223,480	278,019	92,82	n.a.	MB
Total:			1319,054	299,520	100,00	0,000	

(3a*R*,11b*S*)-9,11-dimethoxy-11b-methyl-2,3,3a,11b-tetrahydro-1*H*-cyclopenta[*l*]phenanthren-1-one (11j)

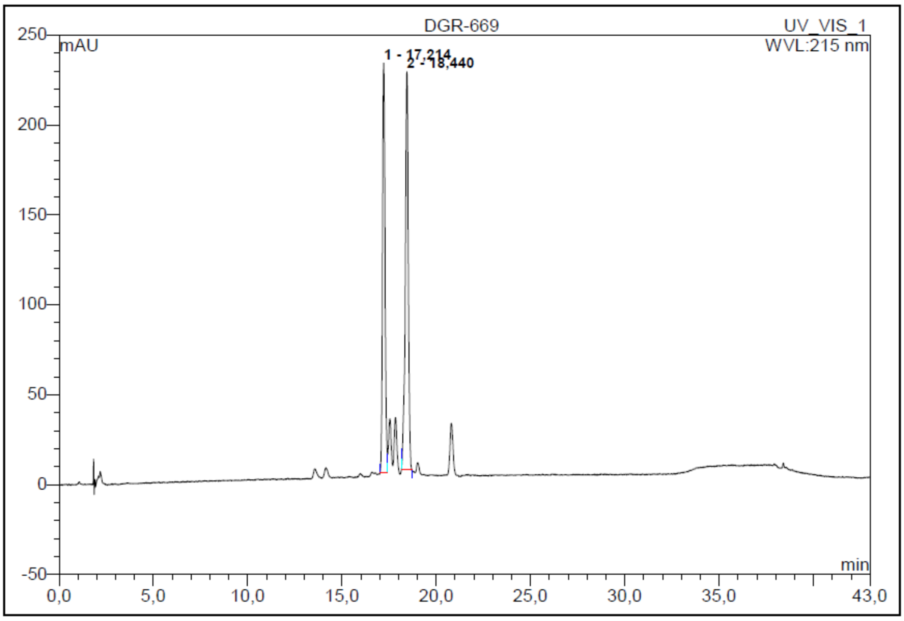

No.	Ret.Time min	Peak Name	Height mAU	Area mAU*min	Rel.Area %	Amount	Type
1	17,21	n.a.	227,516	35,438	46,34	n.a.	BM *
2	18,44	n.a.	221,358	41,040	53,66	n.a.	MB*
Total:			448,874	76,478	100,00	0,000	

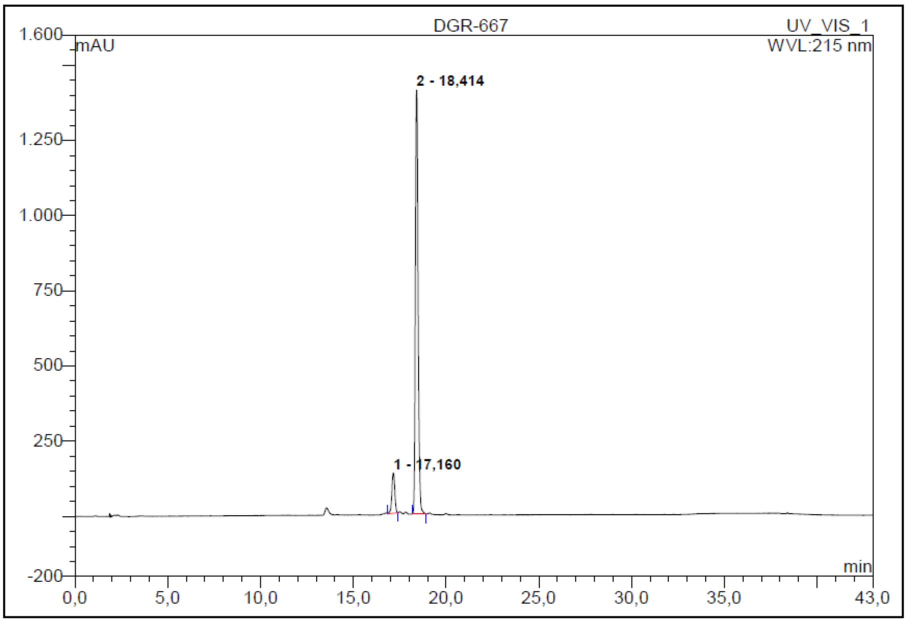

No.	Ret.Time min	Peak Name	Height mAU	Area mAU*min	Rel.Area %	Amount	Type
1	17,16	n.a.	134,345	24,606	9,27	n.a.	BMB
2	18,41	n.a.	1409,891	240,725	90,73	n.a.	BMB
Total:			1544,236	265,331	100,00	0,000	

(3a*S*,11b*S*)-9,11-dimethoxy-11b-methyl-2,3,3a,11b-tetrahydro-1*H*-cyclopenta[*l*]phenanthren-1-one (11j')

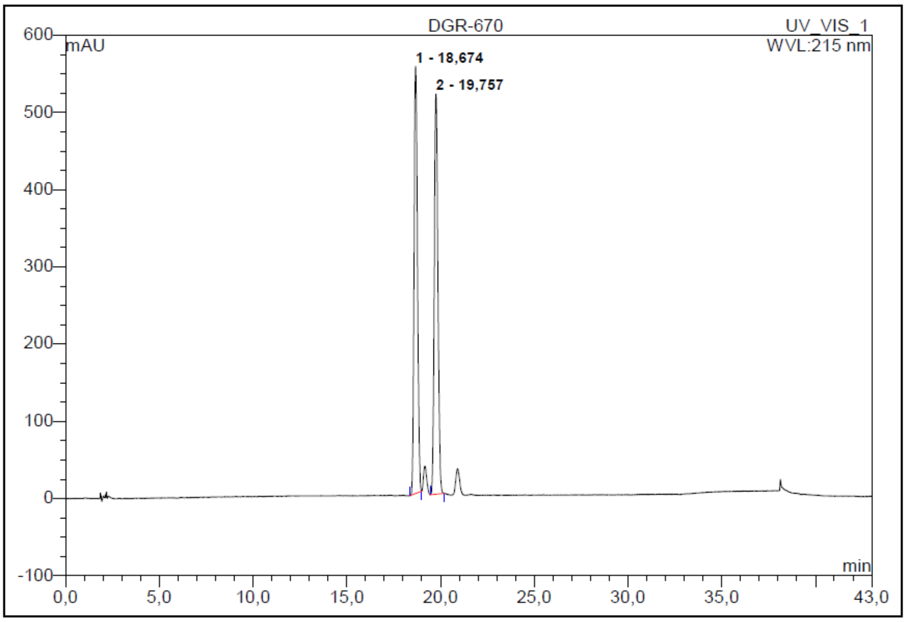

No.	Ret.Time min	Peak Name	Height mAU	Area mAU*min	Rel.Area %	Amount	Type
1	18,67	n.a.	553,269	116,812	49,81	n.a.	BMB
2	19,76	n.a.	518,295	117,725	50,19	n.a.	BMB
Total:			1071,565	234,537	100,00	0,000	

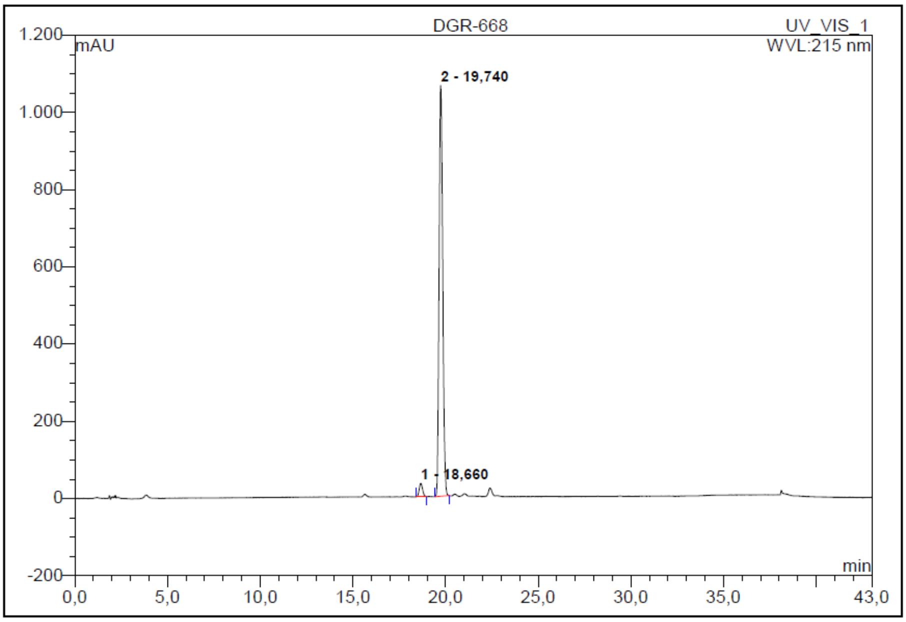

No.	Ret.Time min	Peak Name	Height mAU	Area mAU*min	Rel.Area %	Amount	Type
1	18,66	n.a.	34,556	7,243	2,90	n.a.	BMB*
2	19,74	n.a.	1063,612	242,845	97,10	n.a.	BMB
Total:			1098,167	250,089	100,00	0,000	

(3a*R*,11b*S*)-11b-methyl-9-(trifluoromethyl)-2,3,3a,11b-tetrahydro-1*H*-cyclopenta[*l*]phenanthren-1-one (11k)

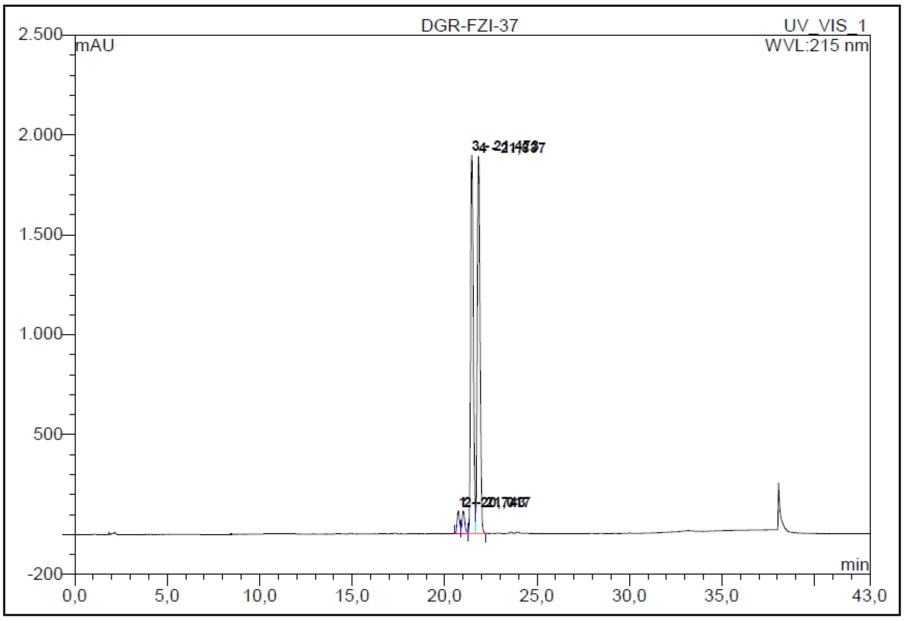

No.	Ret.Time min	Peak Name	Height mAU	Area mAU*min	Rel.Area %	Amount	Type
1	20,74	n.a.	114,791	18,166	2,62	n.a.	BM
2	21,02	n.a.	113,933	19,272	2,78	n.a.	M
3	21,47	n.a.	1894,790	325,777	47,06	n.a.	M
4	21,84	n.a.	1887,380	329,094	47,54	n.a.	MB
Total:			4010,893	692,309	100,00	0,000	

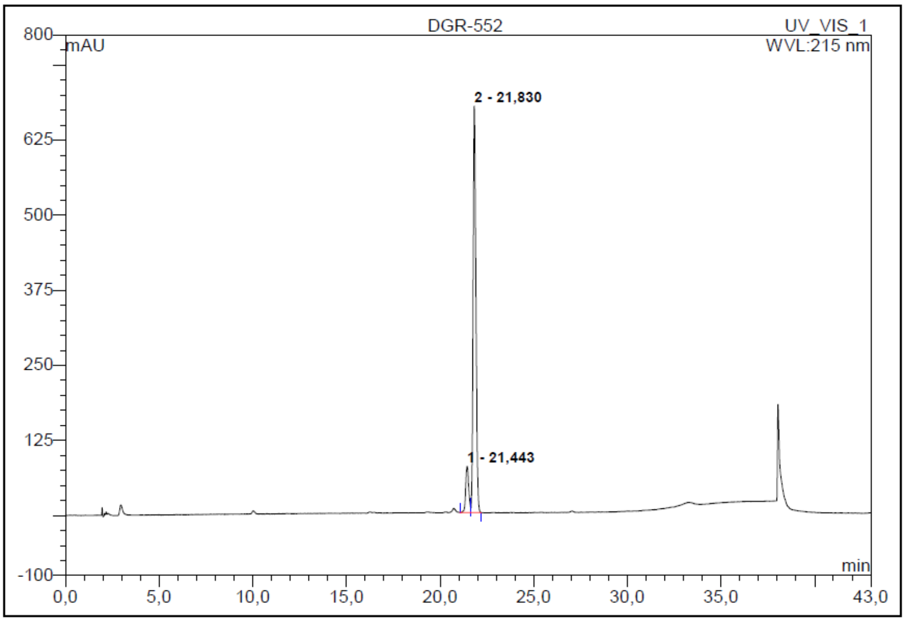

No.	Ret.Time min	Peak Name	Height mAU	Area mAU*min	Rel.Area %	Amount	Type
1	21,44	n.a.	77,093	14,642	11,10	n.a.	BM *
2	21,83	n.a.	677,028	117,272	88,90	n.a.	MB*
Total:			754,121	131,914	100,00	0,000	

(3a*R*,11b*S*)-11b-methyl-9-phenyl-2,3,3a,11b-tetrahydro-1*H*-cyclopenta[*l*]phenanthren-1-one (11l)

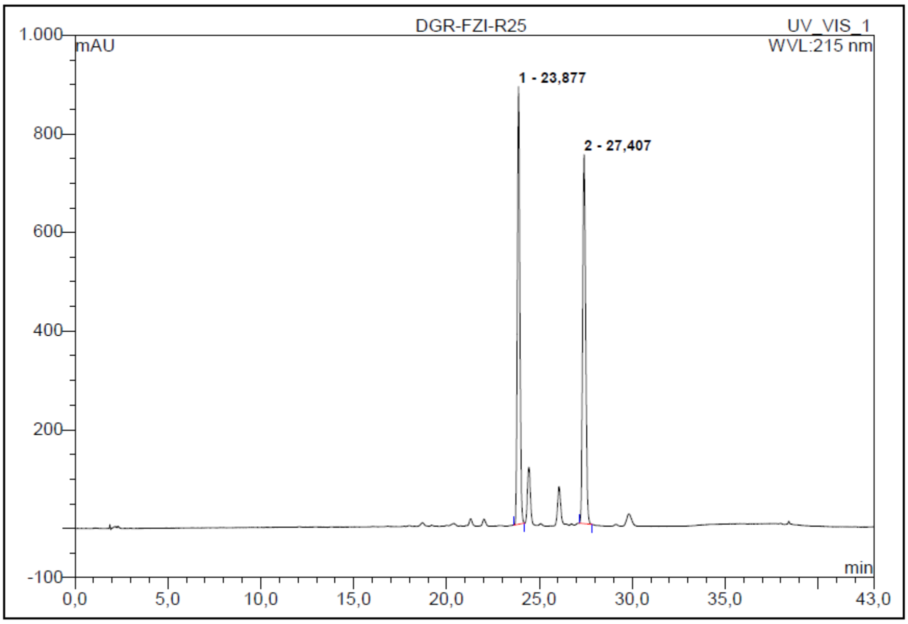

No.	Ret.Time min	Peak Name	Height mAU	Area mAU*min	Rel.Area %	Amount	Type
1	23,88	n.a.	887,417	140,787	49,88	n.a.	BMB*
2	27,41	n.a.	748,572	141,447	50,12	n.a.	BMB
Total:			1635,989	282,235	100,00	0,000	

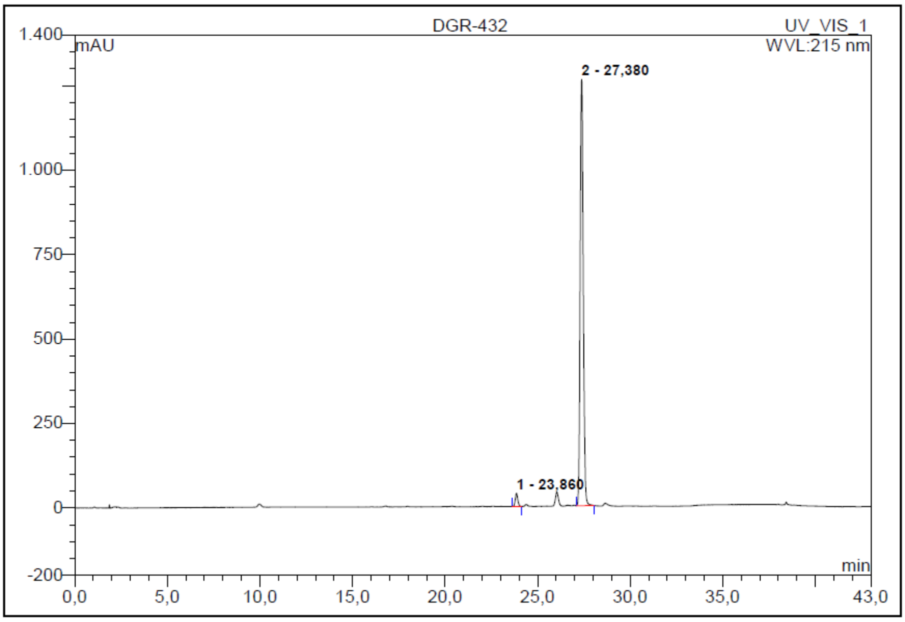

No.	Ret.Time min	Peak Name	Height mAU	Area mAU*min	Rel.Area %	Amount	Type
1	23,86	n.a.	38,984	6,090	2,43	n.a.	BMB*
2	27,38	n.a.	1262,401	244,516	97,57	n.a.	BMB
Total:			1301,386	250,605	100,00	0,000	

(3a*R*,11b*S*)-9,11b-dimethyl-2,3,3a,11b-tetrahydro-1*H*-cyclopenta[*l*]phenanthren-1-one (11m)

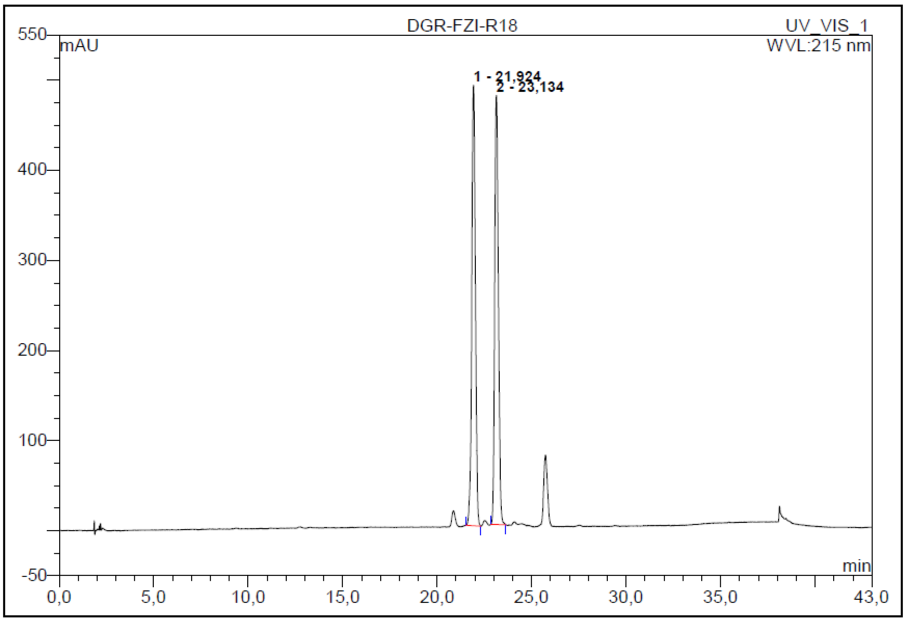

No.	Ret.Time min	Peak Name	Height mAU	Area mAU*min	Rel.Area %	Amount	Type
1	21,92	n.a.	488,983	109,108	50,27	n.a.	BMB
2	23,13	n.a.	476,394	107,927	49,73	n.a.	BMB
Total:			965,376	217,035	100,00	0,000	

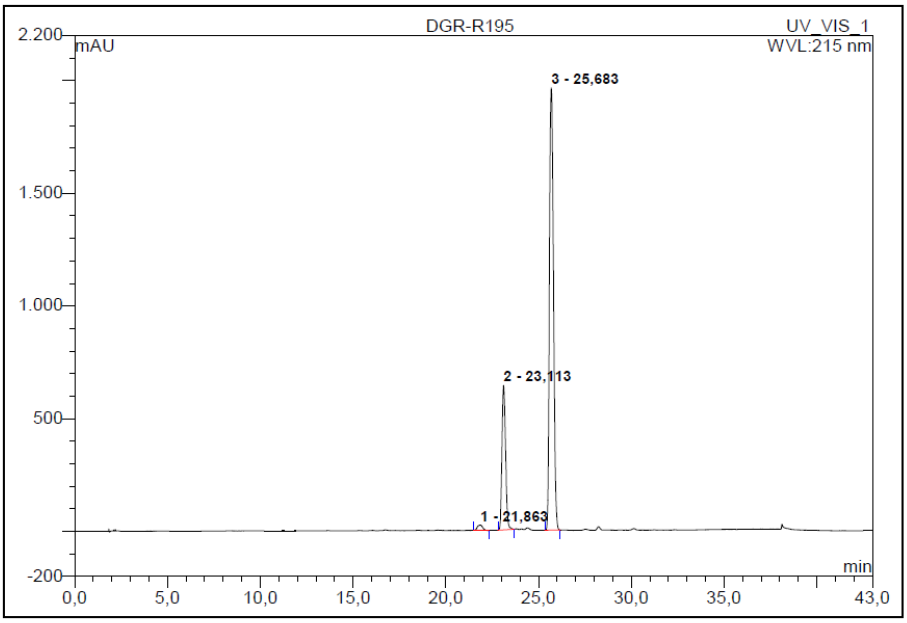

No.	Ret.Time min	Peak Name	Height mAU	Area mAU*min	Rel.Area %	Amount	Type
1	21,86	n.a.	22,872	7,182	1,11	n.a.	BMB*
2	23,11	n.a.	641,142	145,698	22,48	n.a.	BMB
3	25,68	n.a.	1962,239	495,324	76,41	n.a.	BMB
Total:			2626,253	648,205	100,00	0,000	

(1*S*,3a*R*,11b*S*)-11,11b-dimethyl-2,3,3a,11b-tetrahydro-1*H*-cyclopenta[*l*]phenanthren-1-ol ((*S*)-12m')

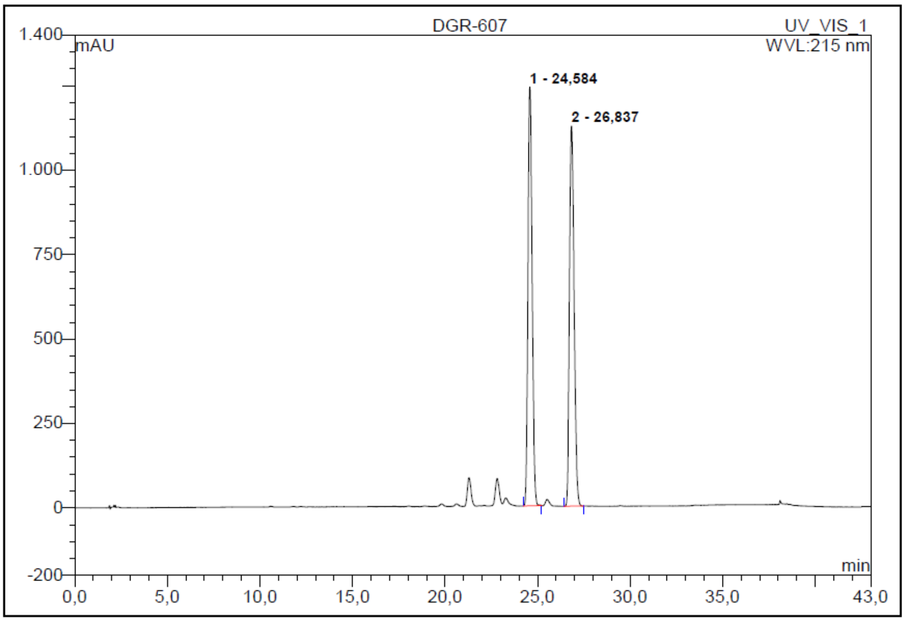

No.	Ret.Time min	Peak Name	Height mAU	Area mAU*min	Rel.Area %	Amount	Type
1	24,58	n.a.	1240,224	327,488	49,88	n.a.	BMB
2	26,84	n.a.	1125,787	329,071	50,12	n.a.	BMB
Total:			2366,011	656,559	100,00	0,000	

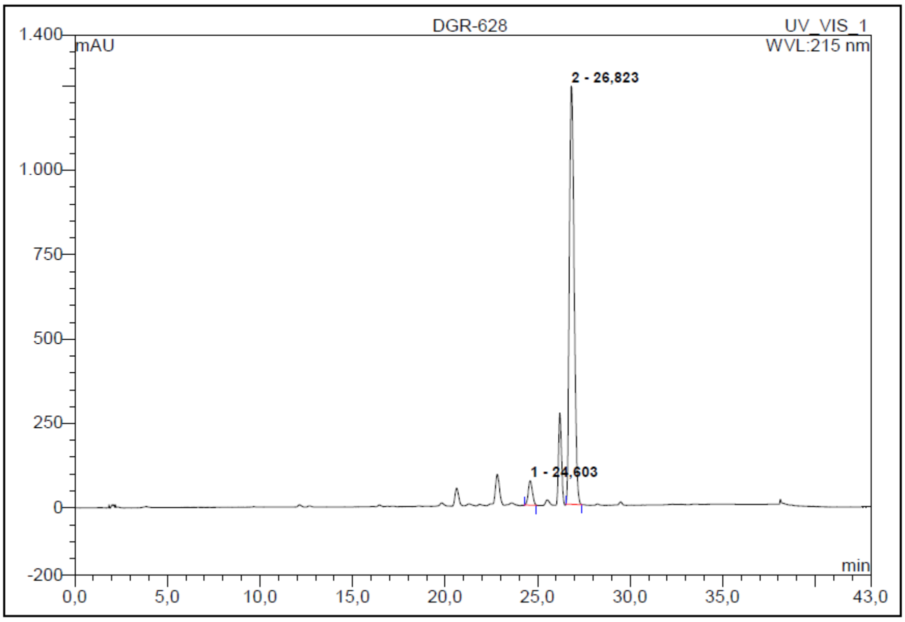

No.	Ret.Time min	Peak Name	Height mAU	Area mAU*min	Rel.Area %	Amount	Type
1	24,60	n.a.	72,493	18,863	4,84	n.a.	BMB*
2	26,82	n.a.	1238,348	371,245	95,16	n.a.	BMB*
Total:			1310,841	390,108	100,00	0,000	

(1*R*,3a*R*,11b*S*)-11,11b-dimethyl-2,3,3a,11b-tetrahydro-1*H*-cyclopenta[*l*]phenanthren-1-ol ((*R*)-12m')

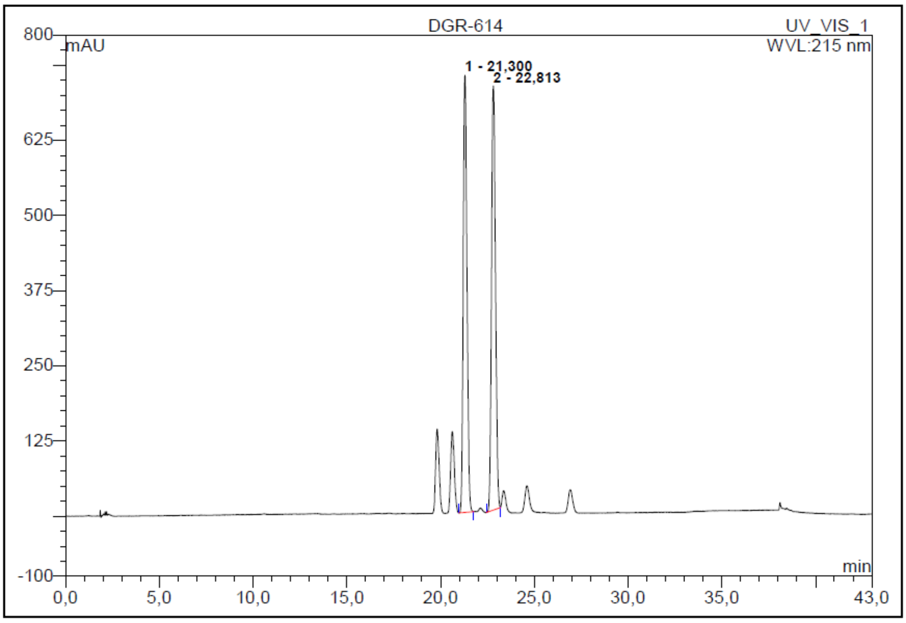

No.	Ret.Time min	Peak Name	Height mAU	Area mAU*min	Rel.Area %	Amount	Type
1	21,30	n.a.	727,169	168,297	48,93	n.a.	BMB
2	22,81	n.a.	705,469	175,691	51,07	n.a.	BMB
Total:			1432,638	343,988	100,00	0,000	

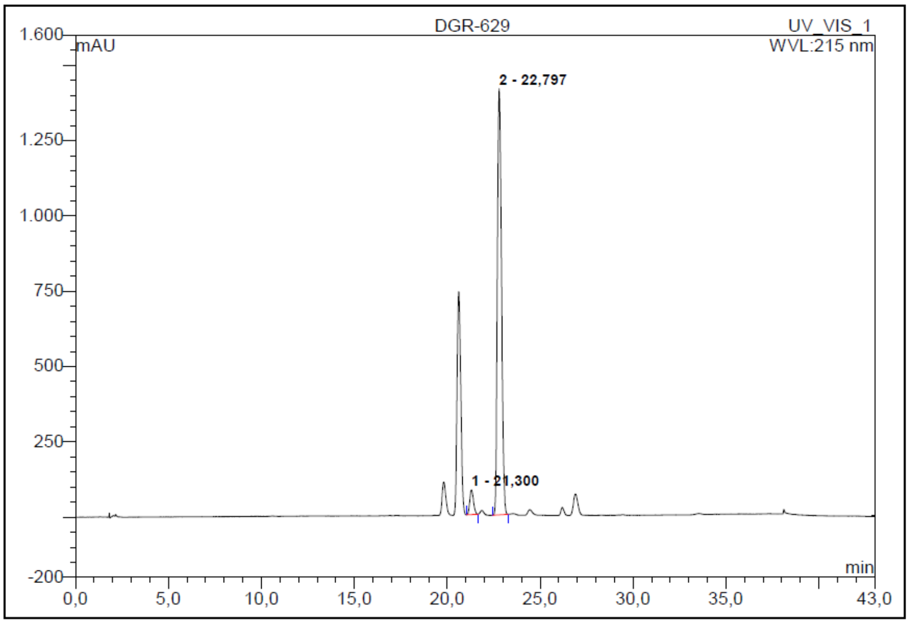

No.	Ret.Time min	Peak Name	Height mAU	Area mAU*min	Rel.Area %	Amount	Type
1	21,30	n.a.	81,879	19,231	4,99	n.a.	BMB
2	22,80	n.a.	1415,421	365,903	95,01	n.a.	BMB
Total:			1497,300	385,134	100,00	0,000	

(3a*R*,11b*S*)-9-fluoro-11b-methyl-2,3,3a,11b-tetrahydro-1*H*-cyclopenta[*l*]phenanthren-1-one (11n)

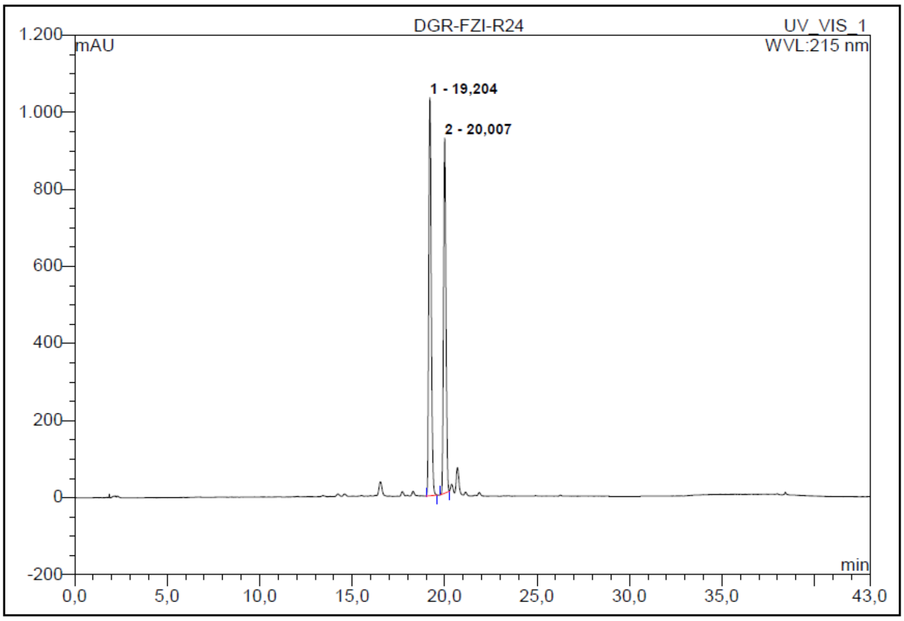

No.	Ret.Time min	Peak Name	Height mAU	Area mAU*min	Rel.Area %	Amount	Type
1	19,20	n.a.	1033,254	159,383	53,15	n.a.	BMB
2	20,01	n.a.	921,100	140,478	46,85	n.a.	BMB
Total:			1954,354	299,861	100,00	0,000	

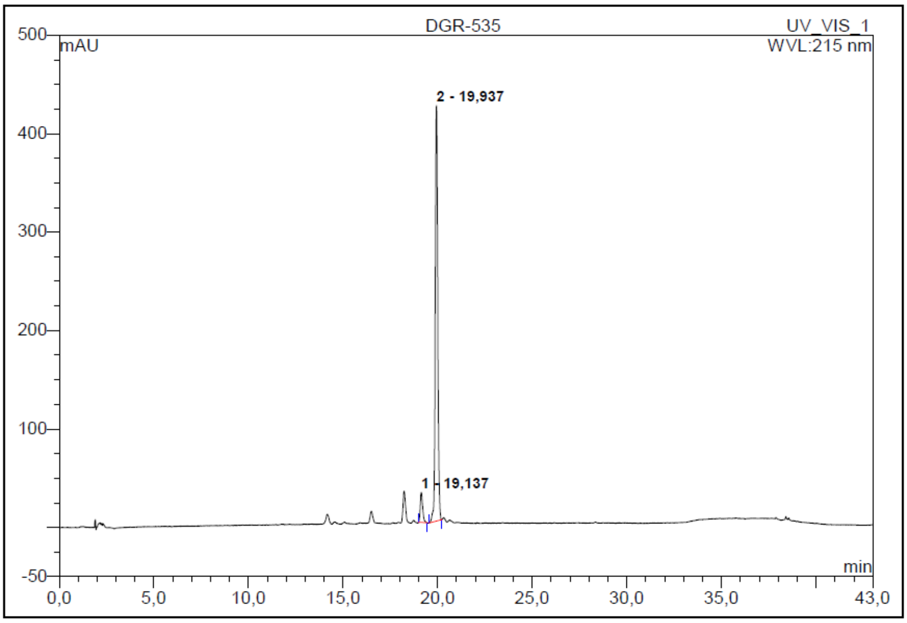

No.	Ret.Time min	Peak Name	Height mAU	Area mAU*min	Rel.Area %	Amount	Type
1	19,14	n.a.	30,363	4,520	6,52	n.a.	BMB*
2	19,94	n.a.	422,159	64,857	93,48	n.a.	BMB
Total:			452,522	69,377	100,00	0,000	

(3a*R*,11b*S*)-11-fluoro-11b-methyl-2,3,3a,11b-tetrahydro-1*H*-cyclopenta[*l*]phenanthren-1-one (11n')

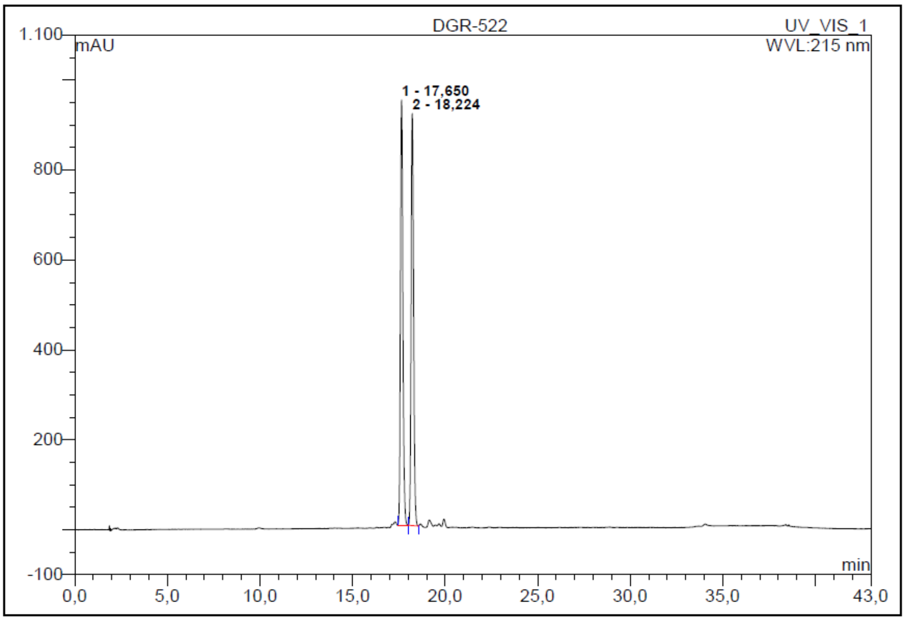

No.	Ret.Time min	Peak Name	Height mAU	Area mAU*min	Rel.Area %	Amount	Type
1	17,65	n.a.	945,484	147,388	50,40	n.a.	BMB
2	18,22	n.a.	916,488	145,060	49,60	n.a.	BMB
Total:			1861,972	292,447	100,00	0,000	

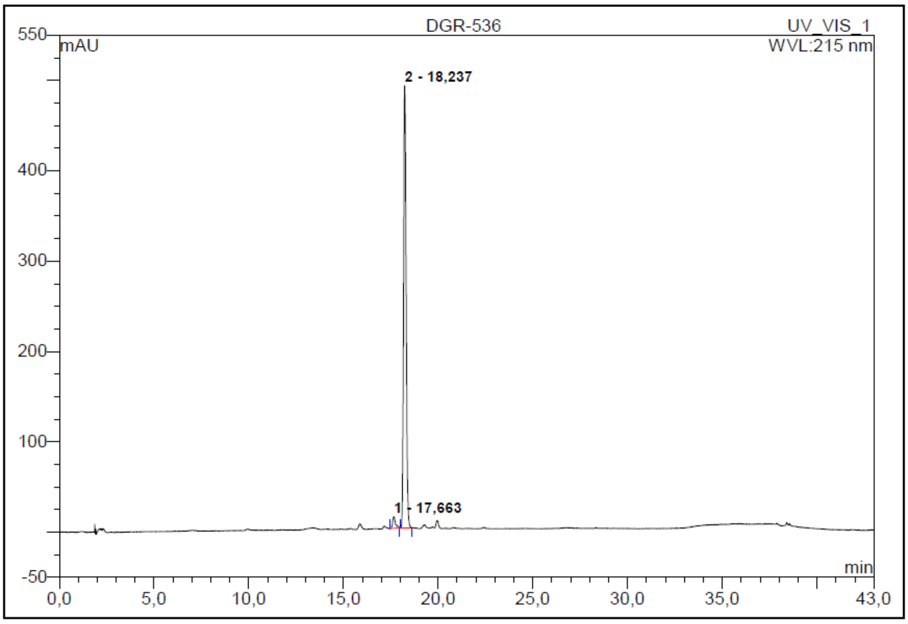

No.	Ret.Time min	Peak Name	Height mAU	Area mAU*min	Rel.Area %	Amount	Type
1	17,66	n.a.	12,606	2,109	2,68	n.a.	BMB*
2	18,24	n.a.	489,684	76,636	97,32	n.a.	BMB
Total:			502,290	78,746	100,00	0,000	

(1*R*,3a*R*,11b*S*)-9-chloro-11b-methyl-2,3,3a,11b-tetrahydro-1*H*-cyclopenta[*l*]phenanthren-1-ol (12o)

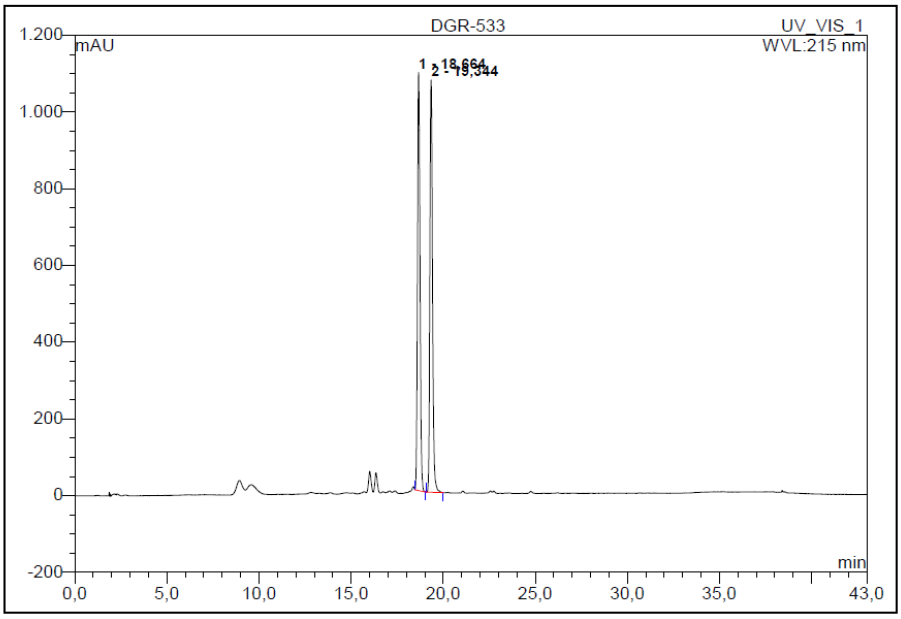

No.	Ret.Time min	Peak Name	Height mAU	Area mAU*min	Rel.Area %	Amount	Type
1	18,66	n.a.	1090,188	172,119	49,17	n.a.	BMB
2	19,34	n.a.	1074,821	177,928	50,83	n.a.	BMB
Total:			2165,008	350,046	100,00	0,000	

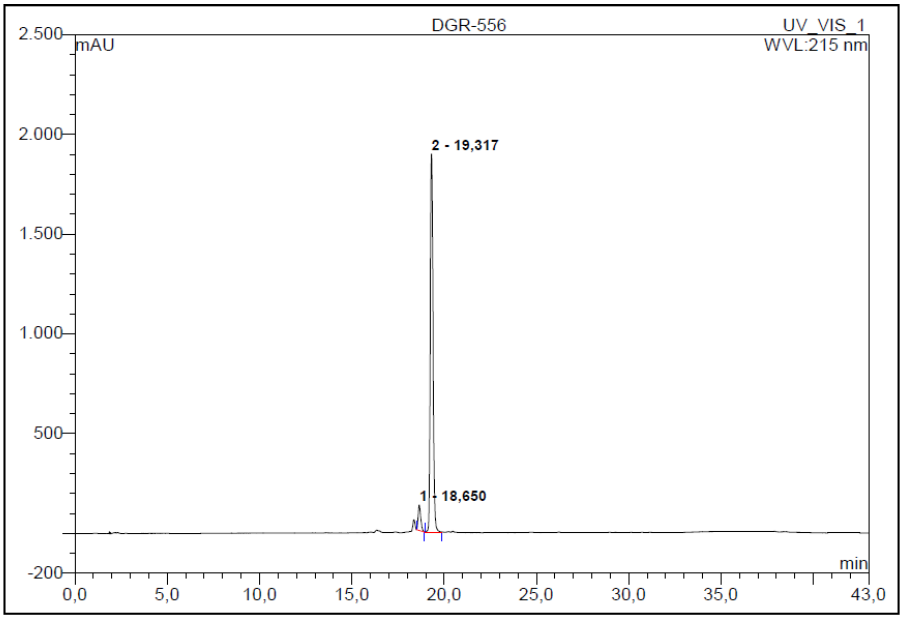

No.	Ret.Time min	Peak Name	Height mAU	Area mAU*min	Rel.Area %	Amount	Type
1	18,65	n.a.	127,964	20,627	5,90	n.a.	BMB
2	19,32	n.a.	1894,846	328,793	94,10	n.a.	BMB
Total:			2022,810	349,420	100,00	0,000	

(1*S*,3a*R*,11b*S*)-11-chloro-11b-methyl-2,3,3a,11b-tetrahydro-1*H*-cyclopenta[*l*]phenanthren-1-ol (12o')

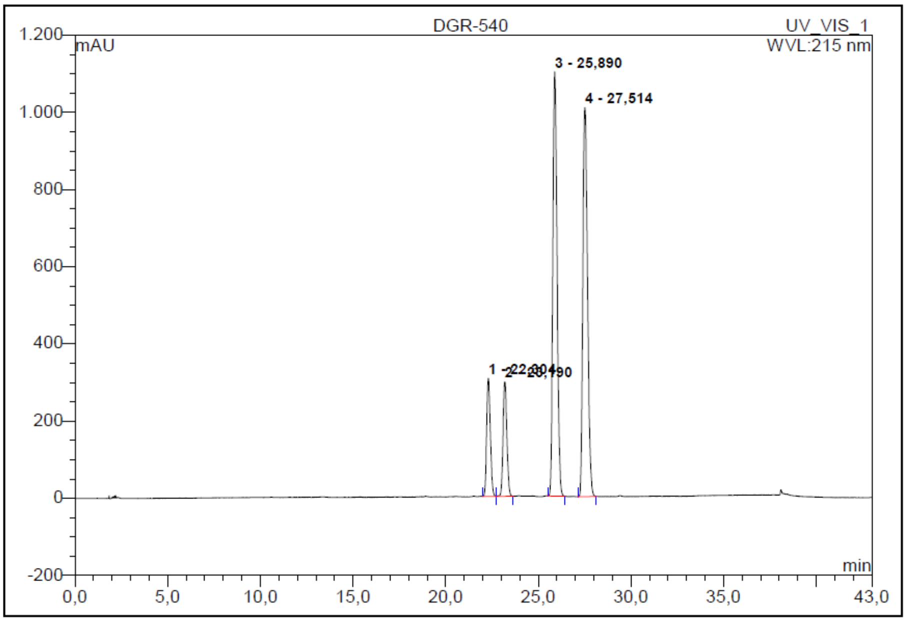

No.	Ret.Time min	Peak Name	Height mAU	Area mAU*min	Rel.Area %	Amount	Type
1	22,30	n.a.	306,359	70,796	9,70	n.a.	BM
2	23,19	n.a.	296,657	70,719	9,69	n.a.	MB
3	25,89	n.a.	1099,397	293,223	40,18	n.a.	BMB
4	27,51	n.a.	1007,700	295,010	40,43	n.a.	BMB
Total:			2710,113	729,748	100,00	0,000	

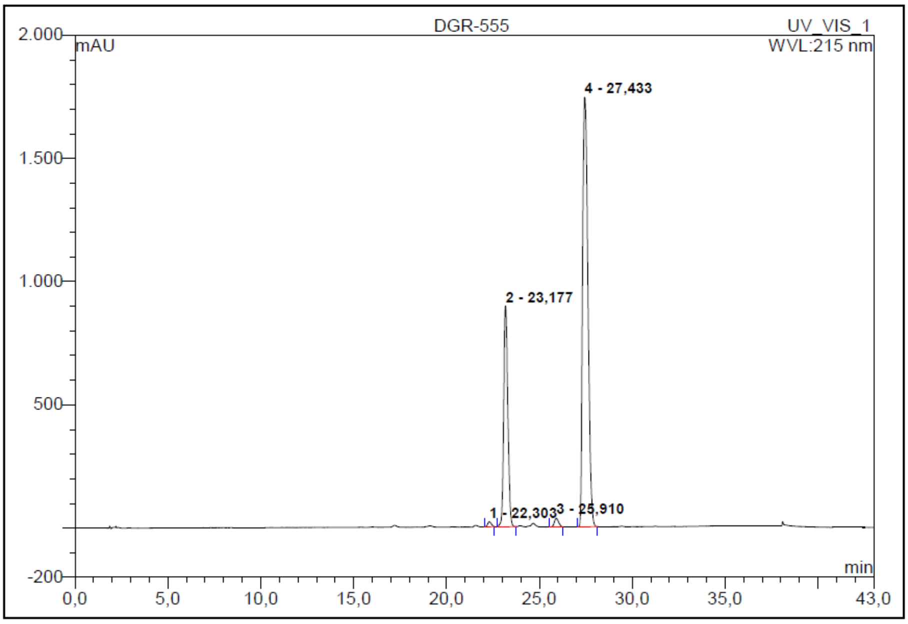

No.	Ret.Time min	Peak Name	Height mAU	Area mAU*min	Rel.Area %	Amount	Type
1	22,30	n.a.	20,093	4,572	0,55	n.a.	BMB*
2	23,18	n.a.	896,315	230,371	27,93	n.a.	BMB
3	25,91	n.a.	34,369	9,018	1,09	n.a.	BMB*
4	27,43	n.a.	1743,958	580,918	70,42	n.a.	BMB*
Total:			2694,735	824,879	100,00	0,000	

(3a*R*,11b*S*)-9-methoxy-11b-methyl-2,3,3a,11b-tetrahydro-1*H*-cyclopenta[*l*]phenanthren-1-one (11p)

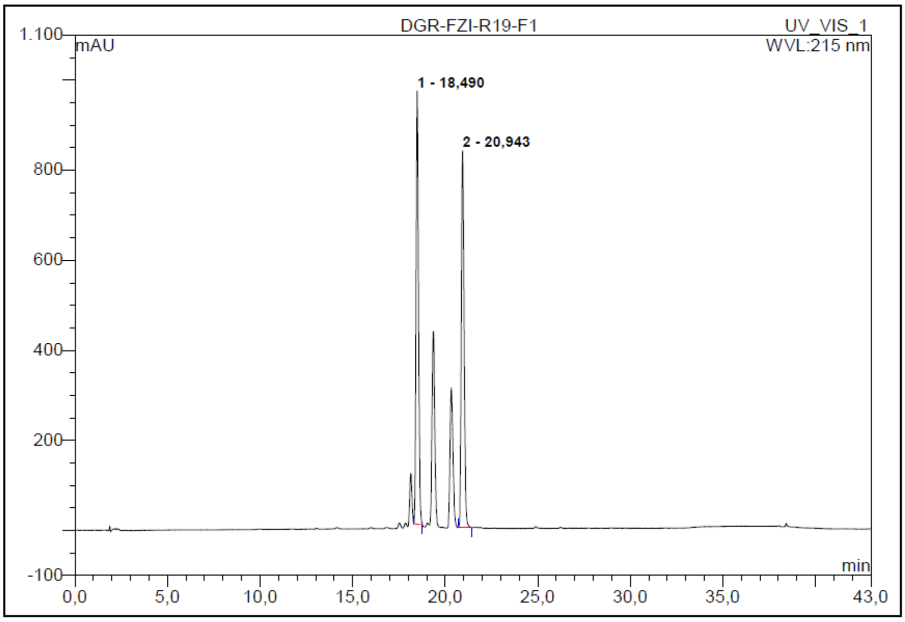

No.	Ret.Time min	Peak Name	Height mAU	Area mAU*min	Rel.Area %	Amount	Type
1	18,49	n.a.	961,822	146,045	49,98	n.a.	BMB*
2	20,94	n.a.	837,070	146,175	50,02	n.a.	BMB*
Total:			1798,892	292,220	100,00	0,000	

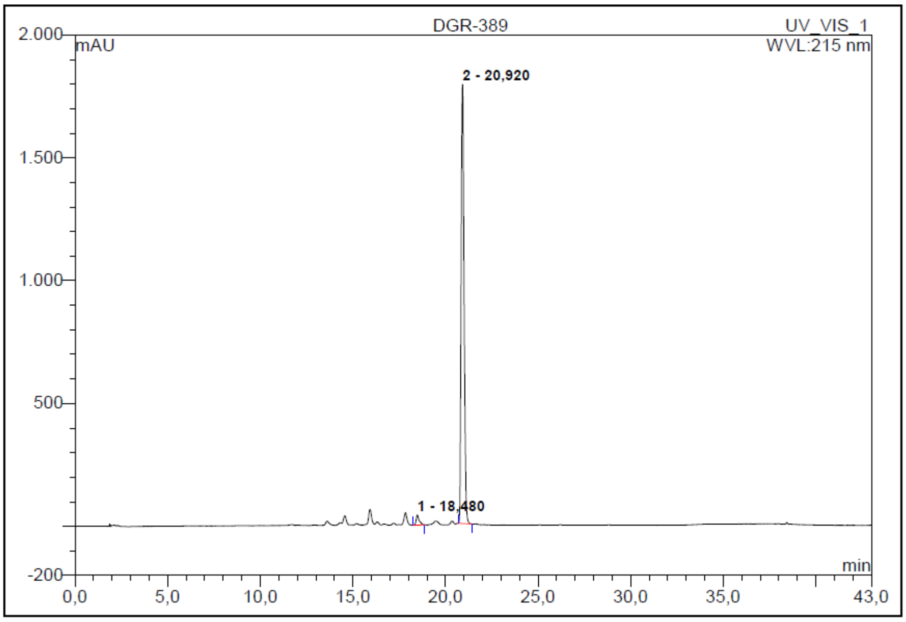

No.	Ret.Time min	Peak Name	Height mAU	Area mAU*min	Rel.Area %	Amount	Type
1	18,48	n.a.	41,164	7,766	2,32	n.a.	BMB*
2	20,92	n.a.	1788,513	327,205	97,68	n.a.	BMB
Total:			1829,677	334,971	100,00	0,000	

(3a*R*,11b*S*)-11-methoxy-11b-methyl-2,3,3a,11b-tetrahydro-1*H*-cyclopenta[*l*]phenanthren-1-one (11p')

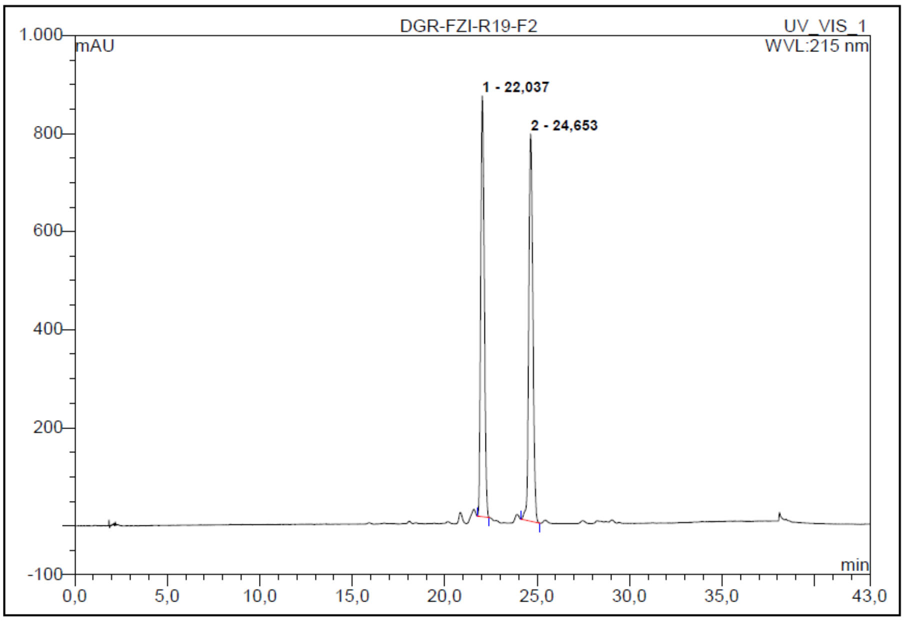

No.	Ret.Time min	Peak Name	Height mAU	Area mAU*min	Rel.Area %	Amount	Type
1	22,04	n.a.	858,665	194,216	48,89	n.a.	BMB
2	24,65	n.a.	790,285	203,060	51,11	n.a.	BMB
Total:			1648,950	397,276	100,00	0,000	

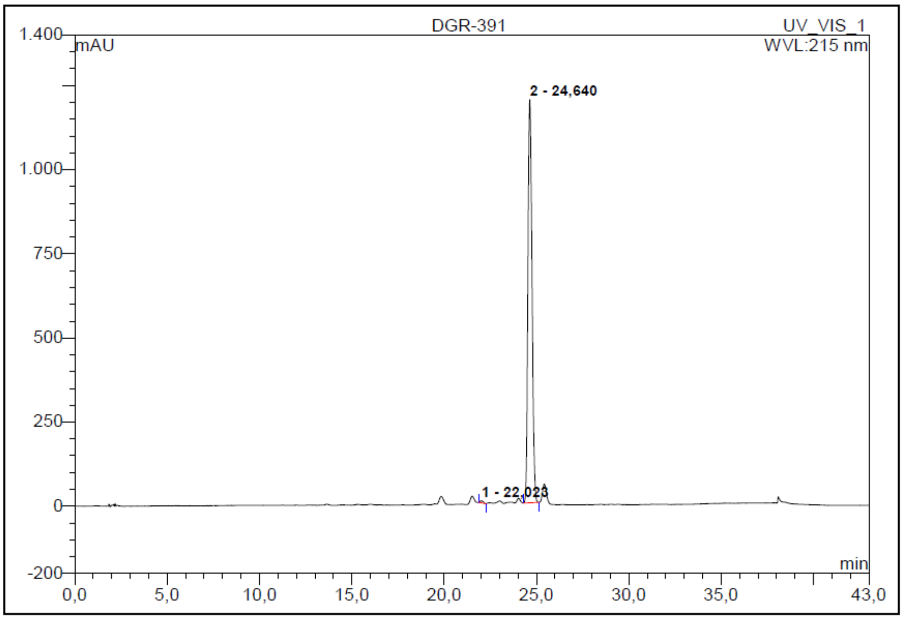

No.	Ret.Time min	Peak Name	Height mAU	Area mAU*min	Rel.Area %	Amount	Type
1	22,02	n.a.	6,127	1,065	0,34	n.a.	BMB*
2	24,64	n.a.	1198,213	308,445	99,66	n.a.	BMB*
Total:			1204,340	309,510	100,00	0,000	

(1*R*,3a*R*,11b*S*)-11b-methyl-2,3,3a,11b-tetrahydro-1*H*-cyclopenta[*l*]phenanthren-1-yl 4-bromobenzoate (14)

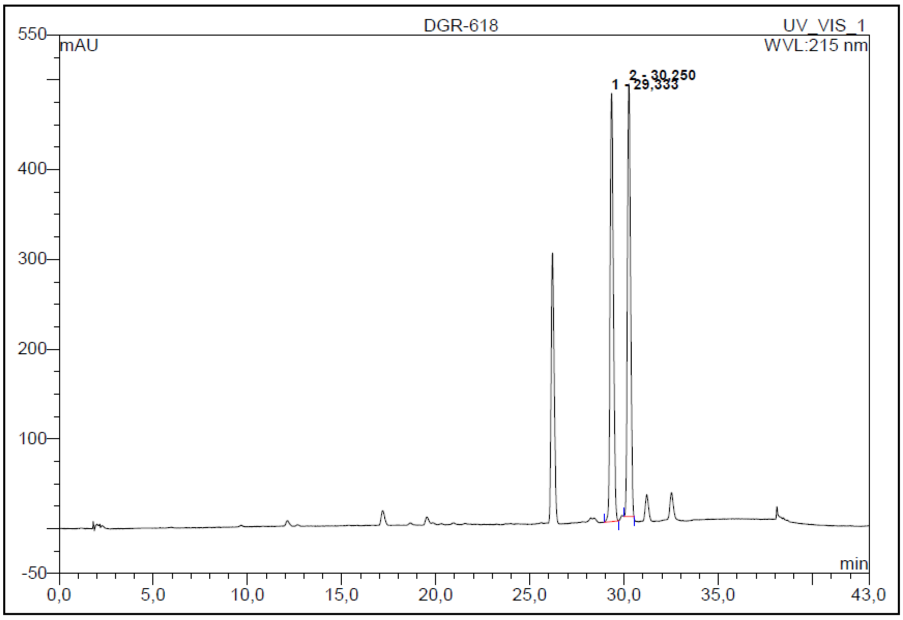

No.	Ret.Time min	Peak Name	Height mAU	Area mAU*min	Rel.Area %	Amount	Type
1	29,33	n.a.	477,198	96,896	51,00	n.a.	BMB
2	30,25	n.a.	482,120	93,101	49,00	n.a.	BMB*
Total:			959,318	189,997	100,00	0,000	

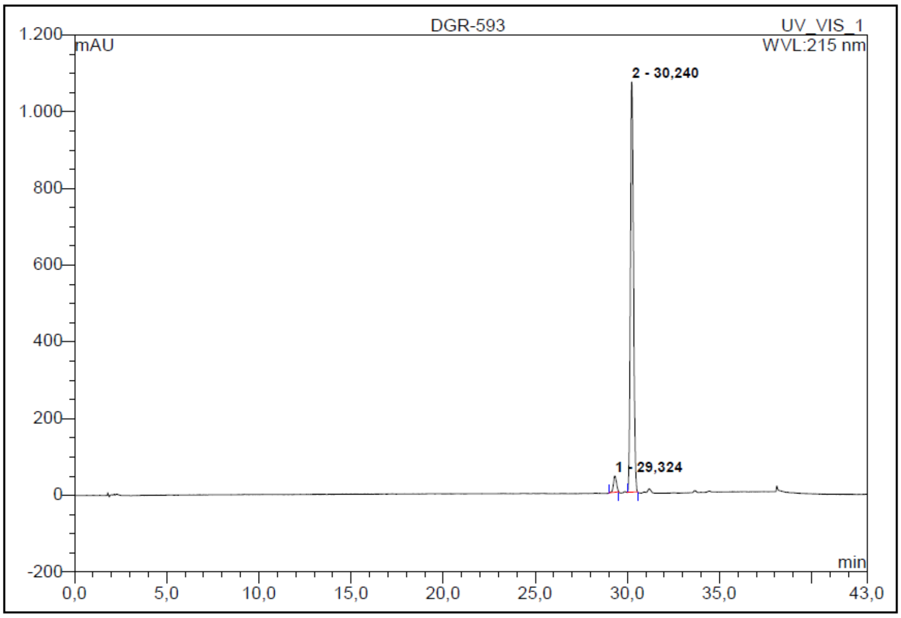

No.	Ret.Time min	Peak Name	Height mAU	Area mAU*min	Rel.Area %	Amount	Type
1	29,32	n.a.	42,078	8,178	3,75	n.a.	BMB*
2	30,24	n.a.	1068,723	210,038	96,25	n.a.	BMB*
Total:			1110,802	218,216	100,00	0,000	

Crystal of **14** used for SC-XRD:

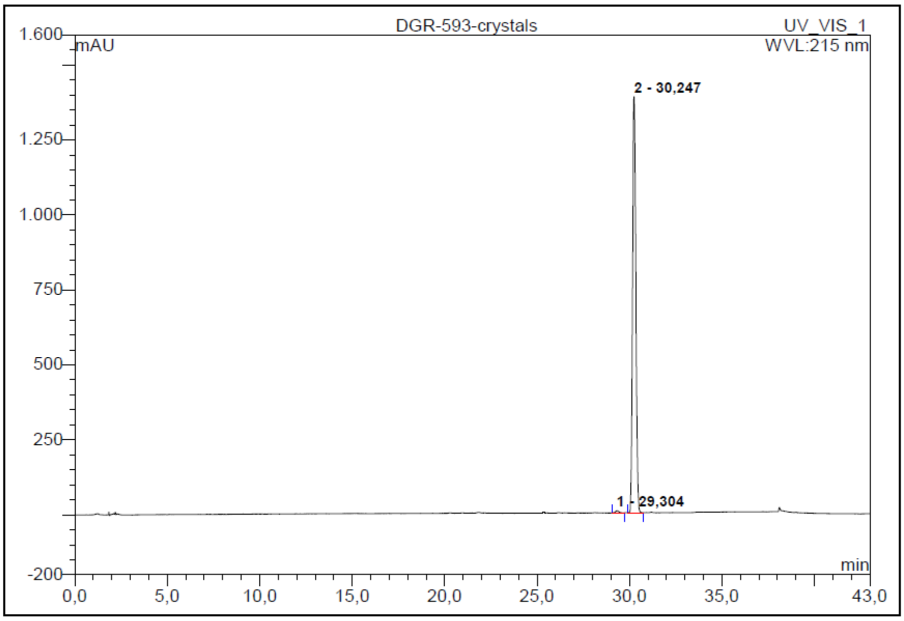

No.	Ret.Time min	Peak Name	Height mAU	Area mAU*min	Rel.Area %	Amount	Type
1	29,30	n.a.	7,319	1,531	0,53	n.a.	BMB*
2	30,25	n.a.	1388,496	288,956	99,47	n.a.	BMB*
Total:			1395,815	290,487	100,00	0,000	

NMR Spectra of New Compounds

3-(2-bromophenyl)-2-methylcyclopent-2-en-1-one (S6)

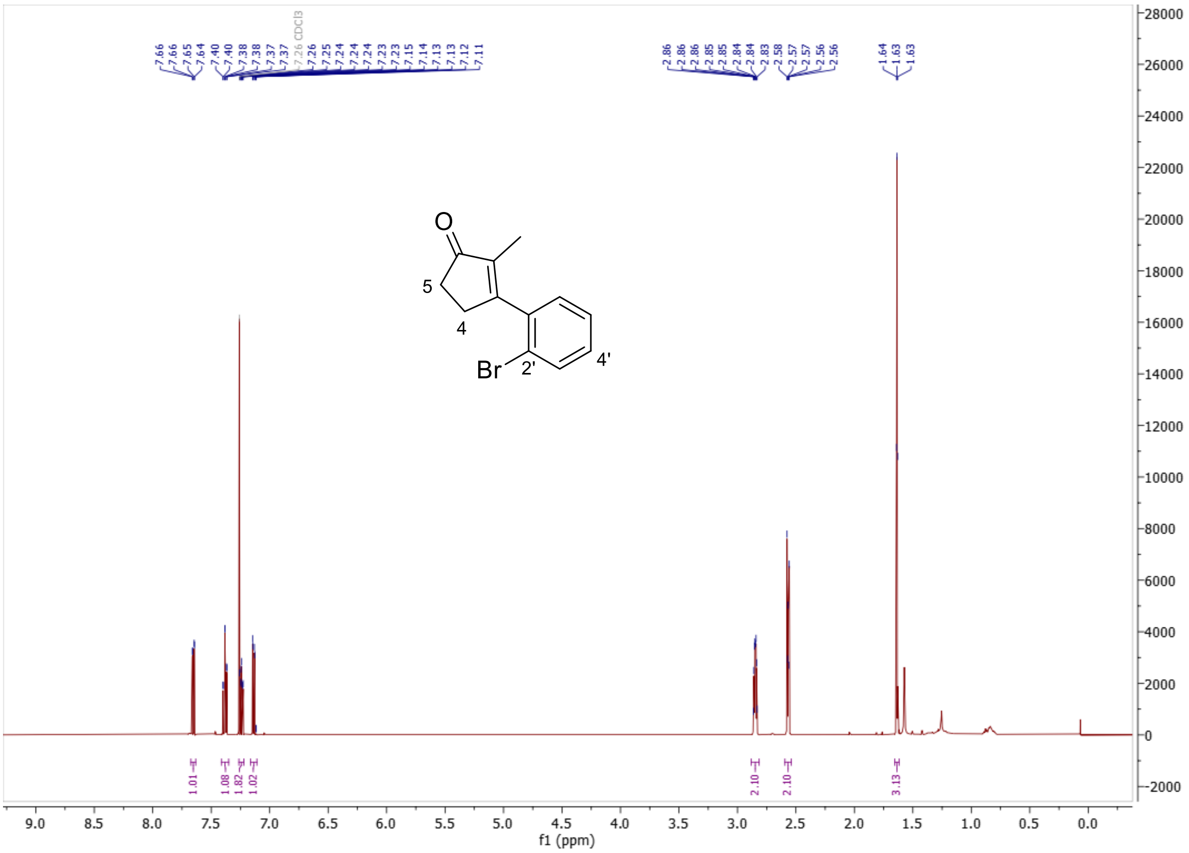

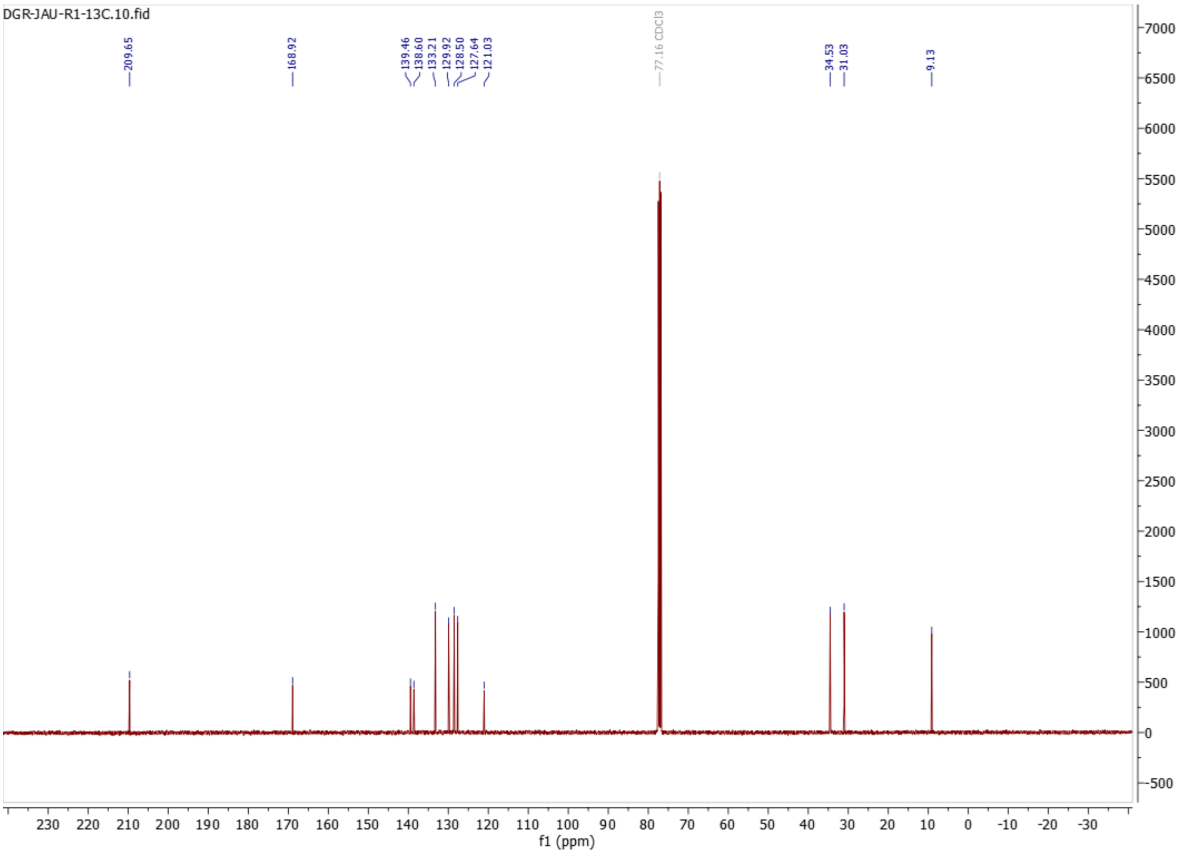

3-([1,1'-biphenyl]-2-yl-2',3',4',5',6'-d_5)-2-methylcyclopent-2-en-1-one (9a-d_5)

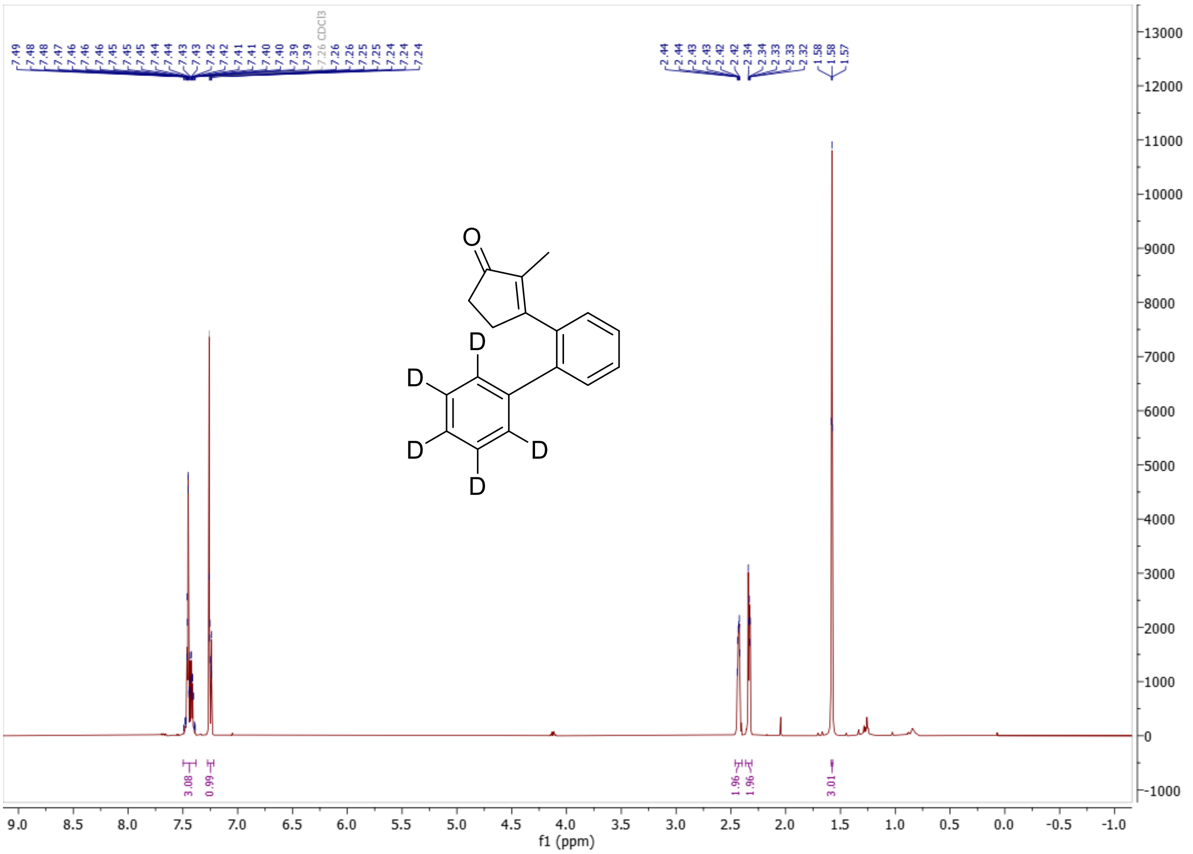

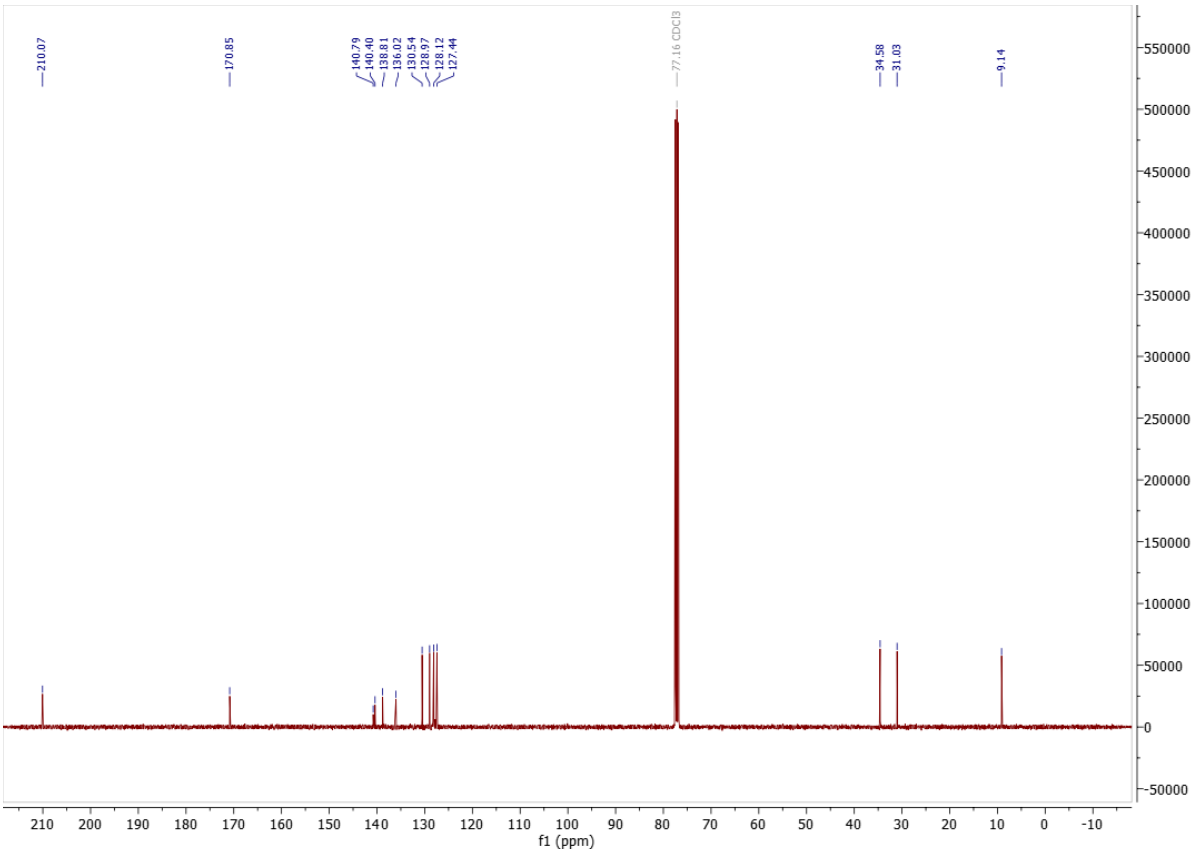

2-methyl-3-(4'-methyl-[1,1'-biphenyl]-2-yl)cyclopent-2-en-1-one (9b)

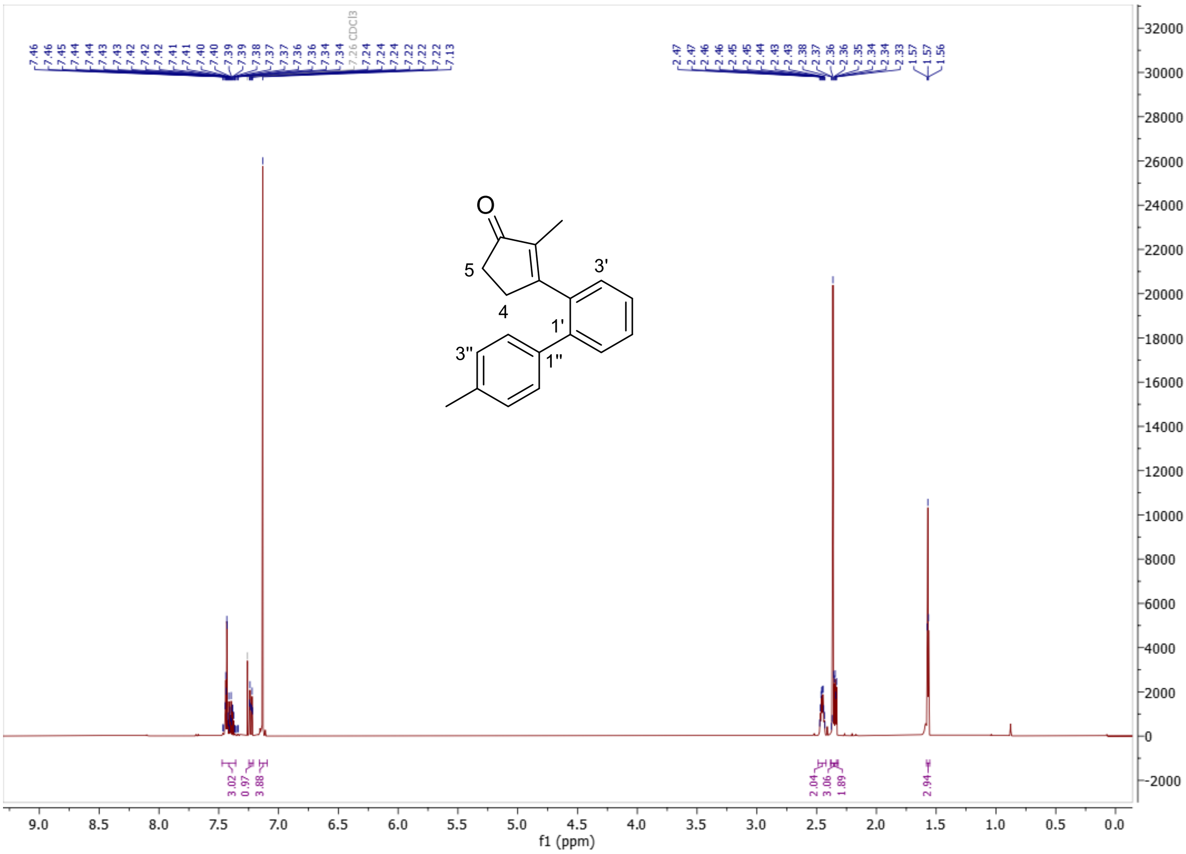

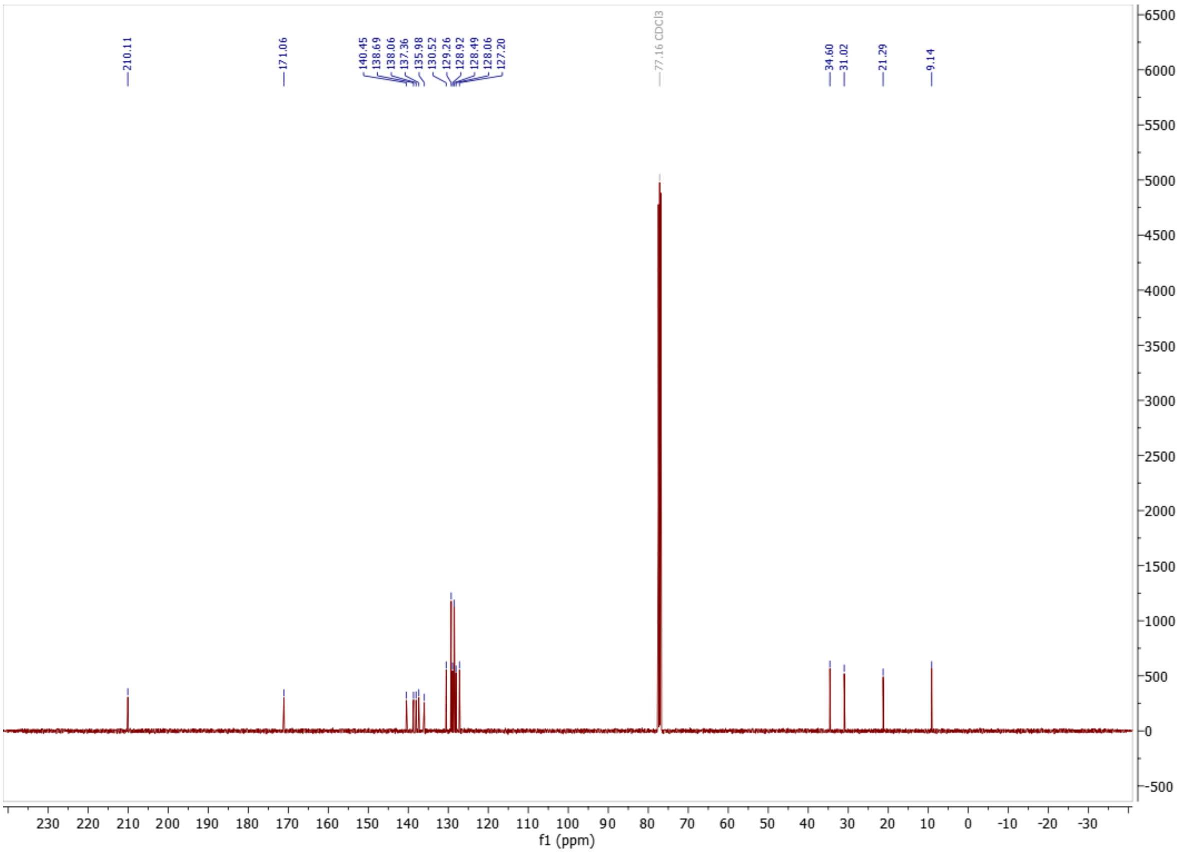

3-(4'-fluoro-[1,1'-biphenyl]-2-yl)-2-methylcyclopent-2-en-1-one (9c)

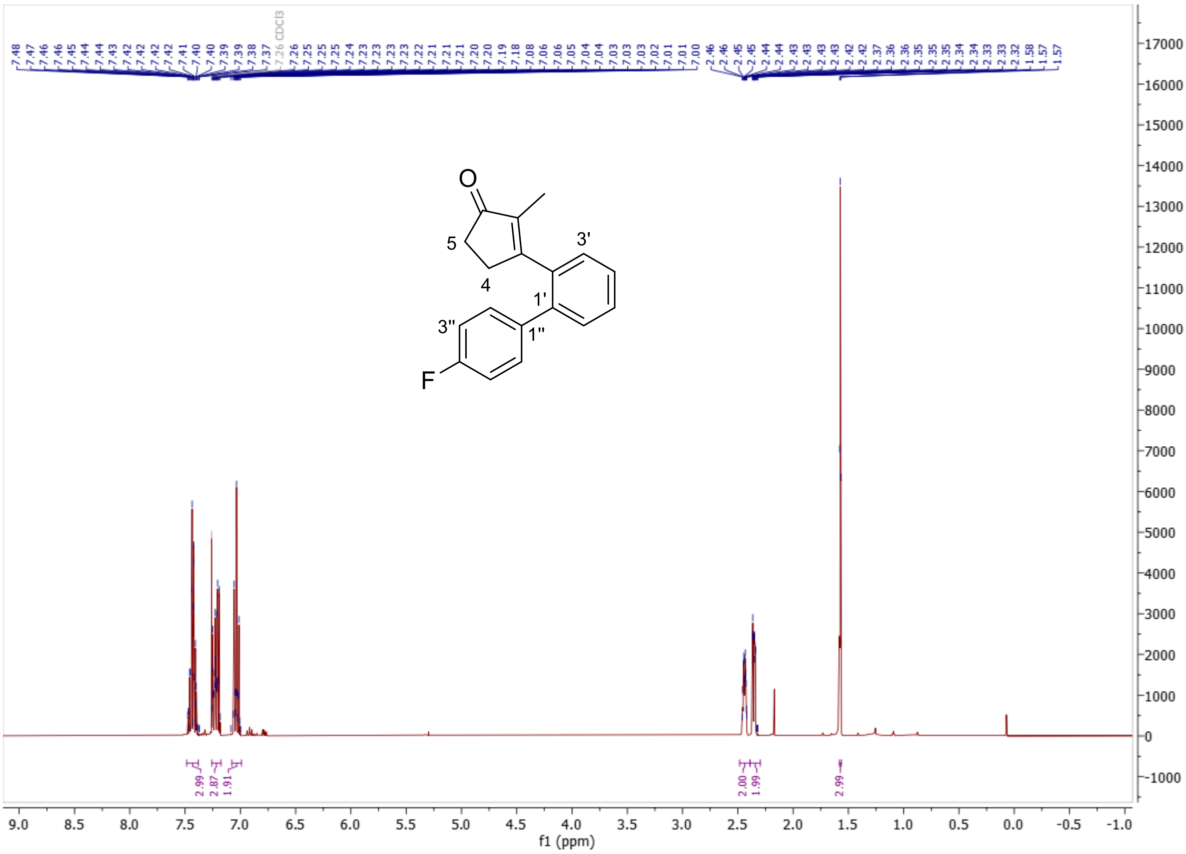

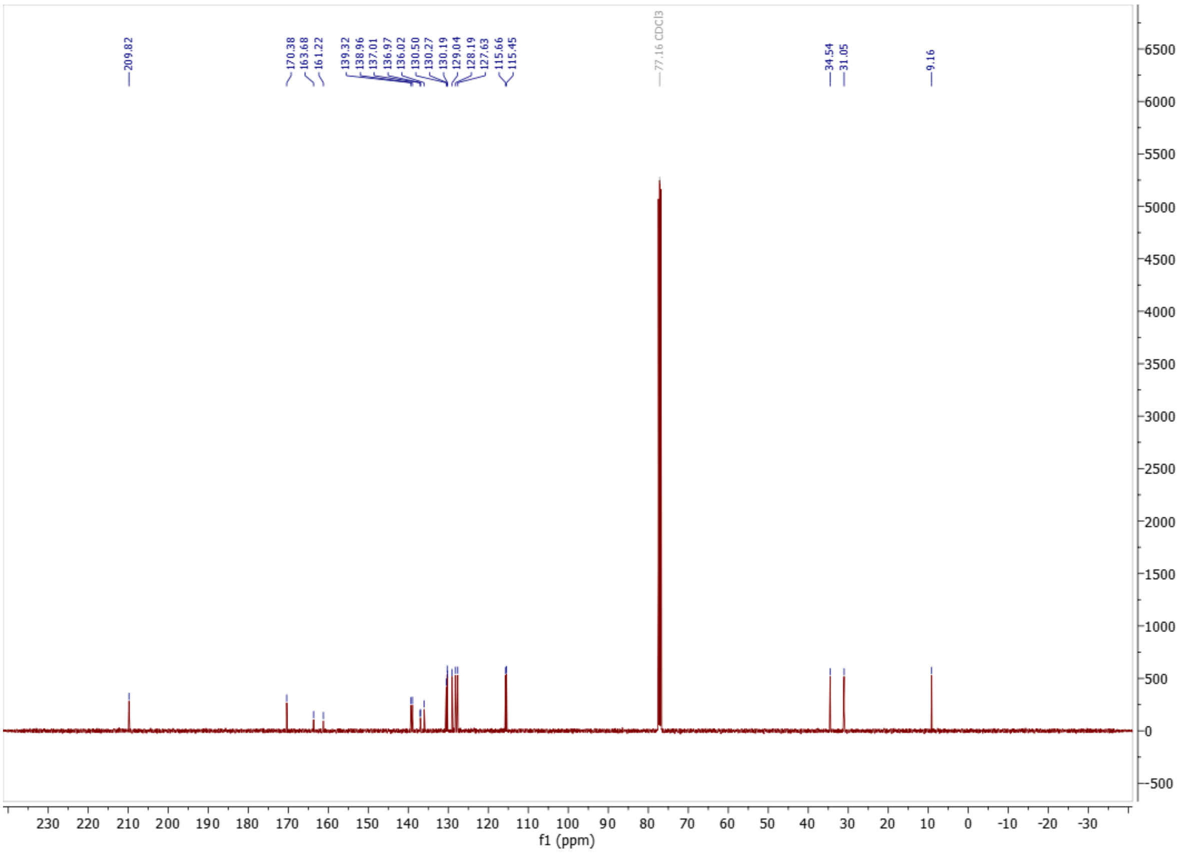

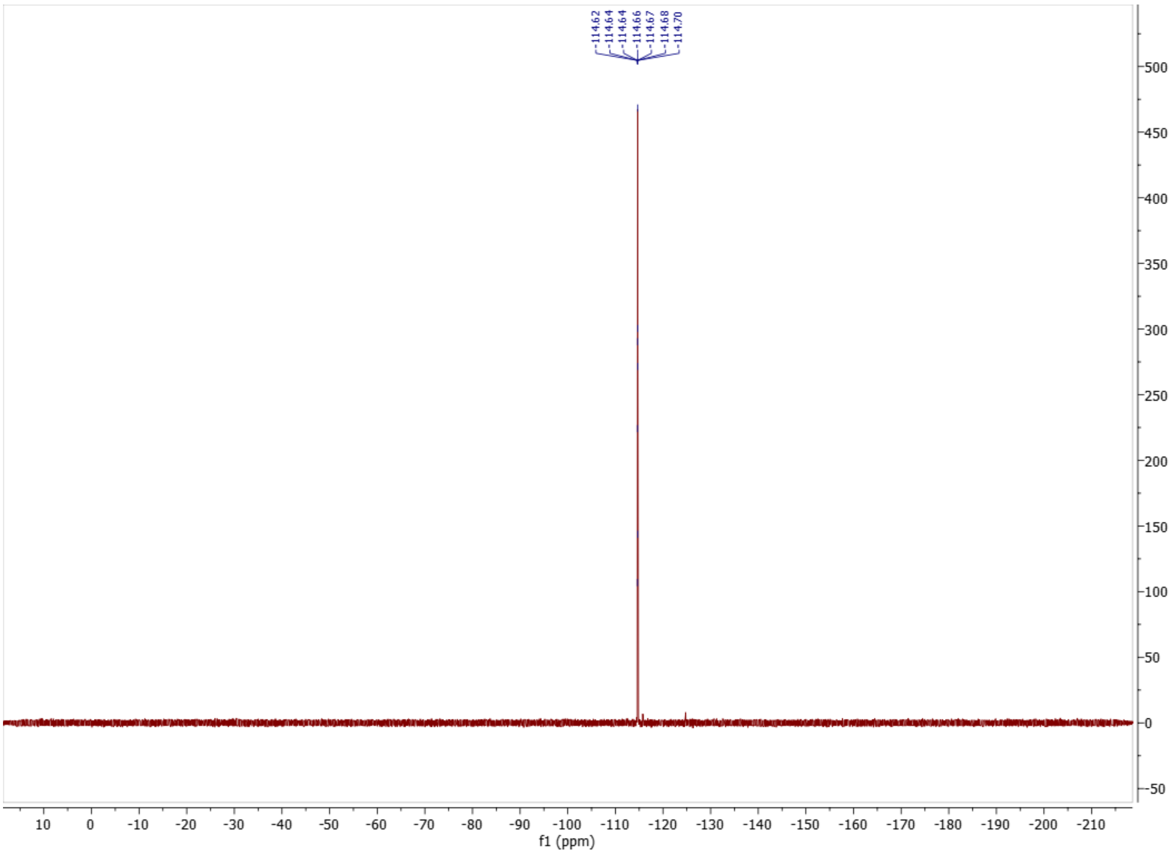

-114.62
-114.64
-114.64
-114.66
-114.67
-114.68
-114.70
f1 (ppm)

2-methyl-3-(4'-(trifluoromethyl)-[1,1'-biphenyl]-2-yl)cyclopent-2-en-1-one (9d)

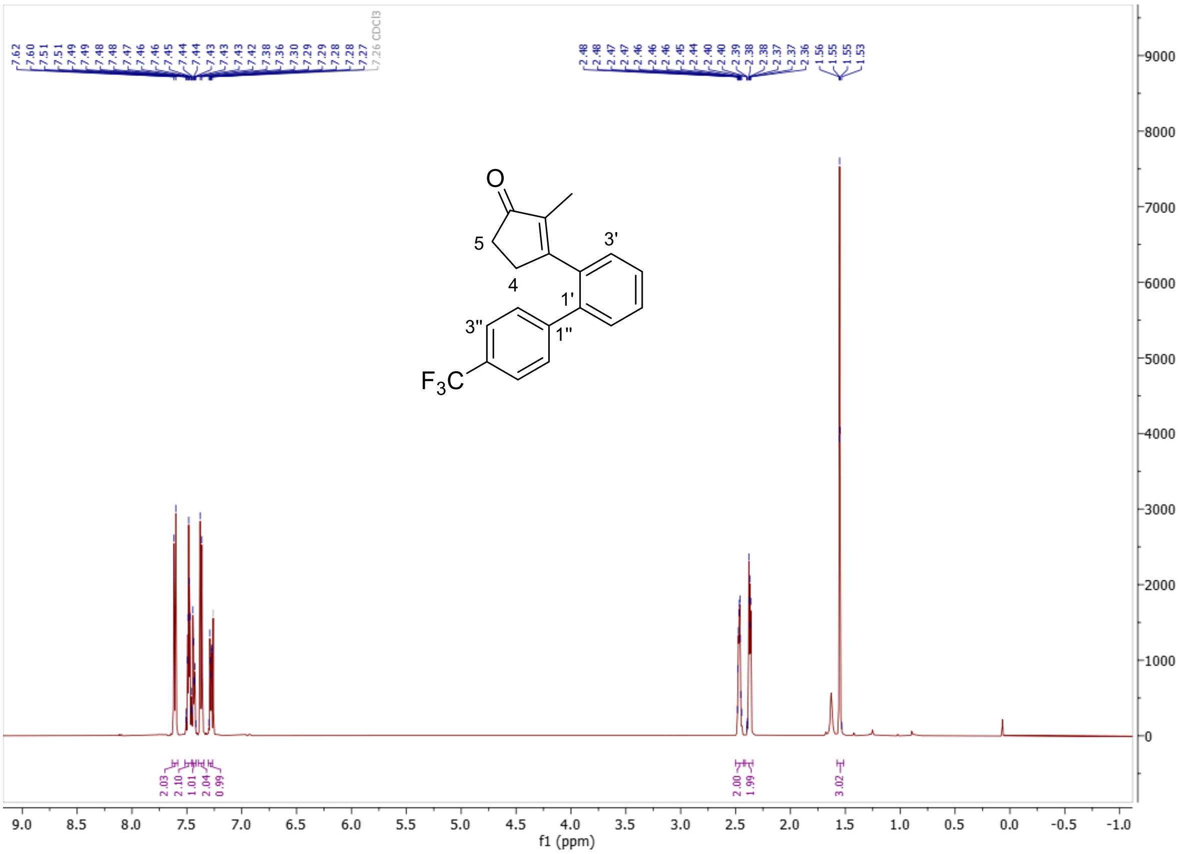

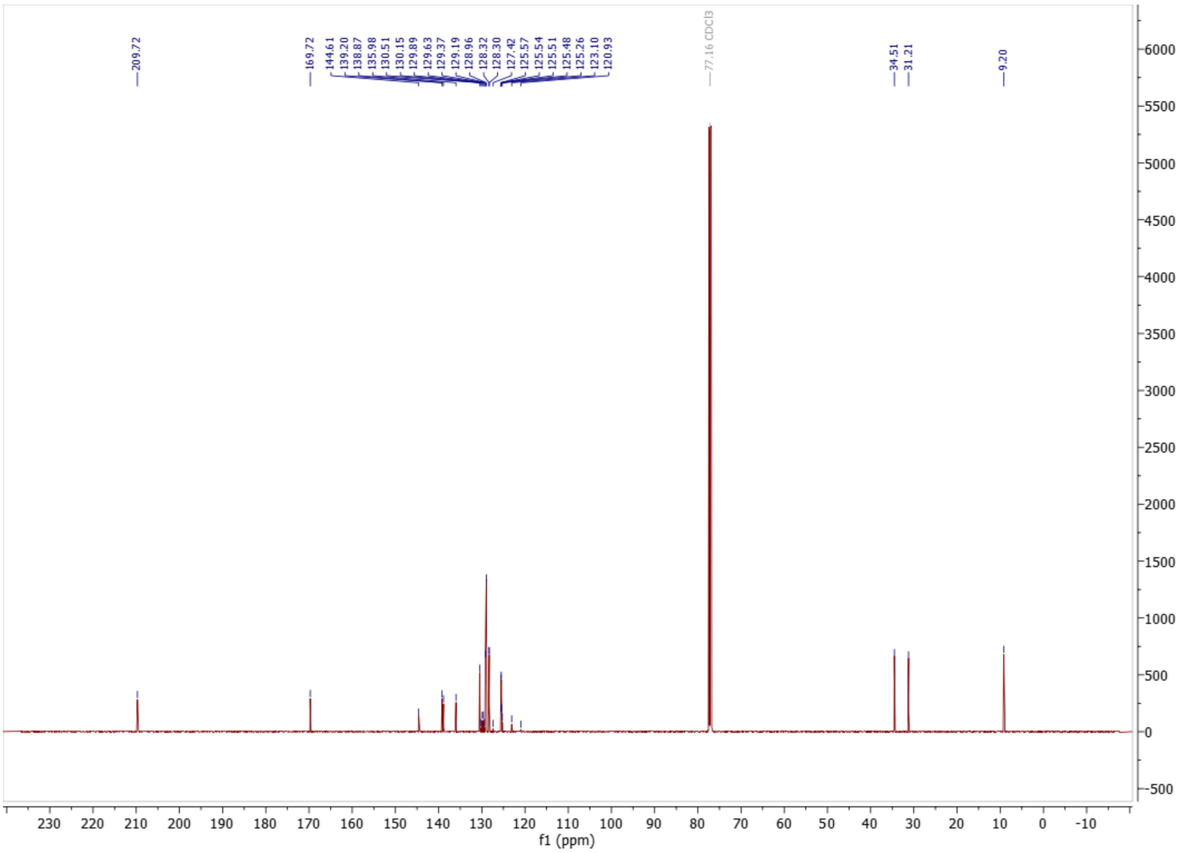

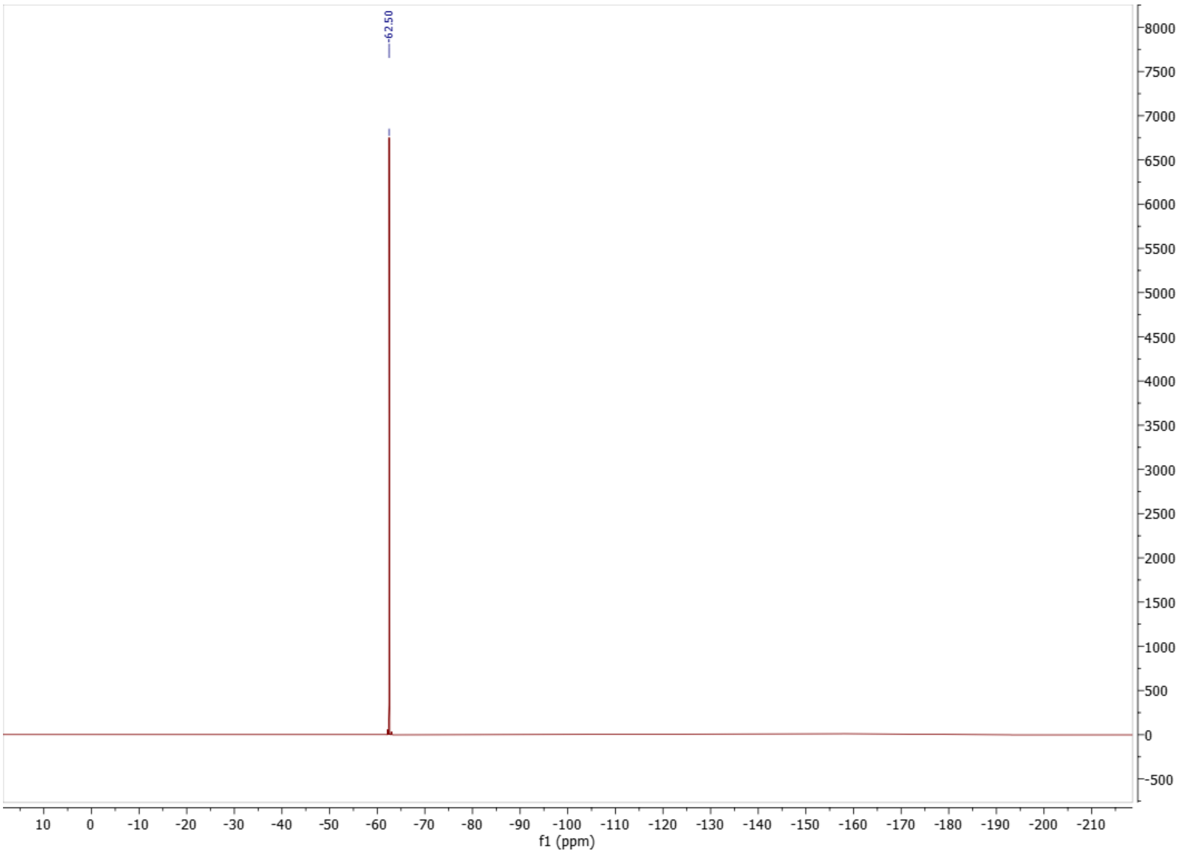
-62.50
8000
7500
7000
6500
6000
5500
5000
4500
4000
3500
3000
2500
2000
1500
1000
500
0
-500
10 0 -10 -20 -30 -40 -50 -60 -70 -80 -90 -100 -110 -120 -130 -140 -150 -160 -170 -180 -190 -200 -210
f1 (ppm)

3-(4'-(*tert*-butyl)-[1,1'-biphenyl]-2-yl)-2-methylcyclopent-2-en-1-one (9e)

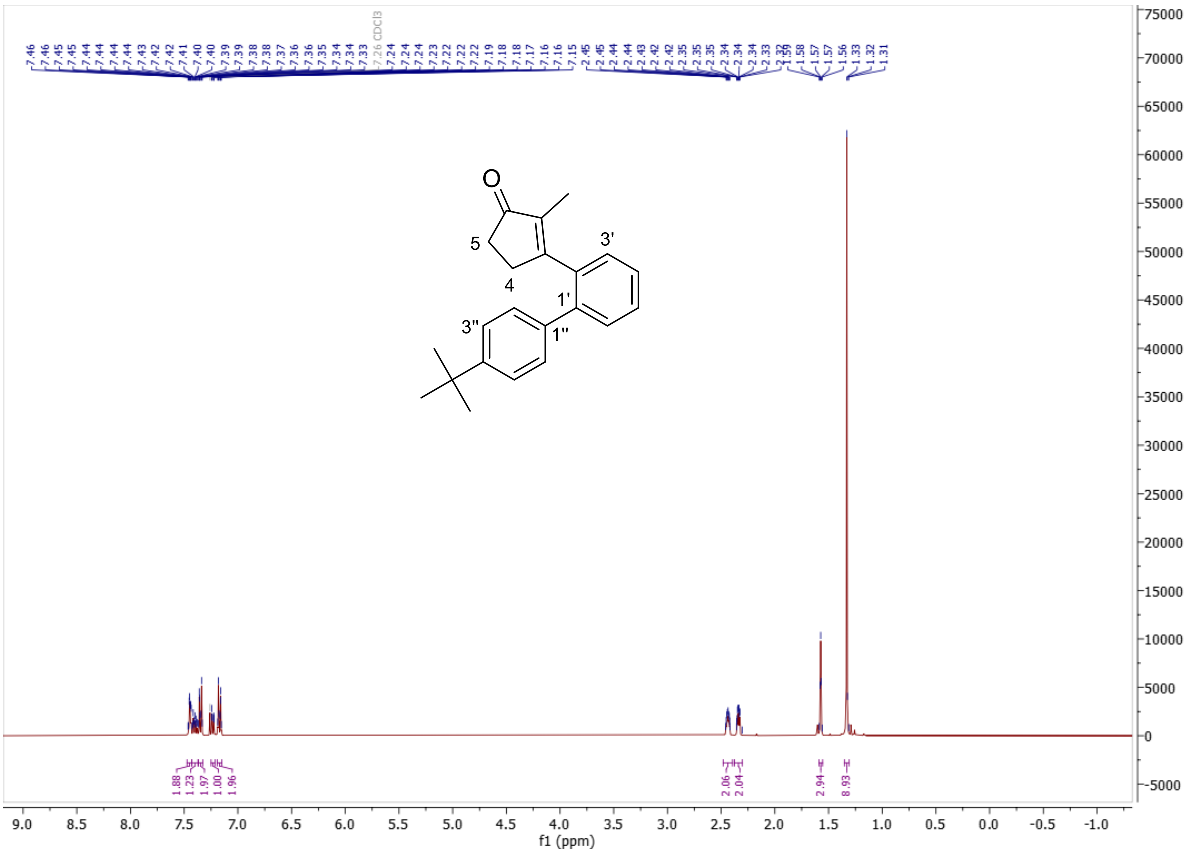

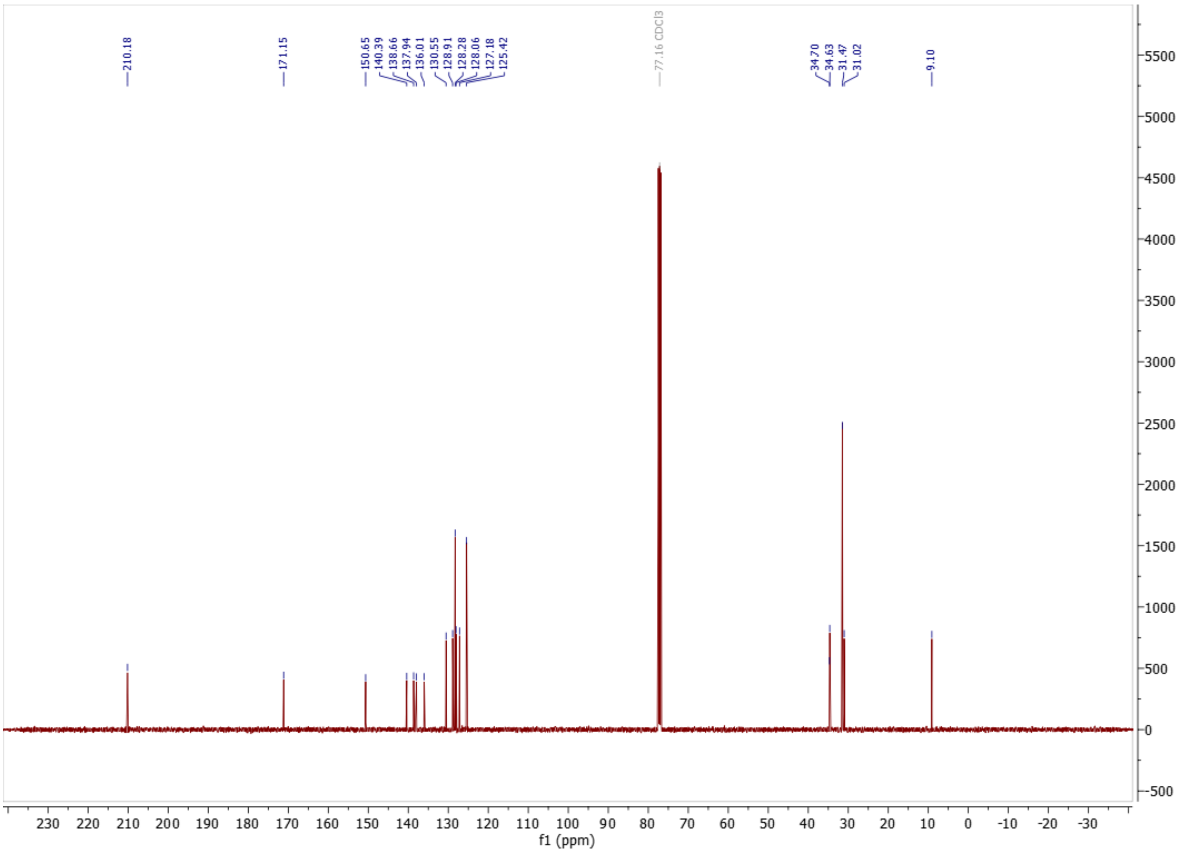

3-(4'-chloro-[1,1'-biphenyl]-2-yl)-2-methylcyclopent-2-en-1-one (9f)

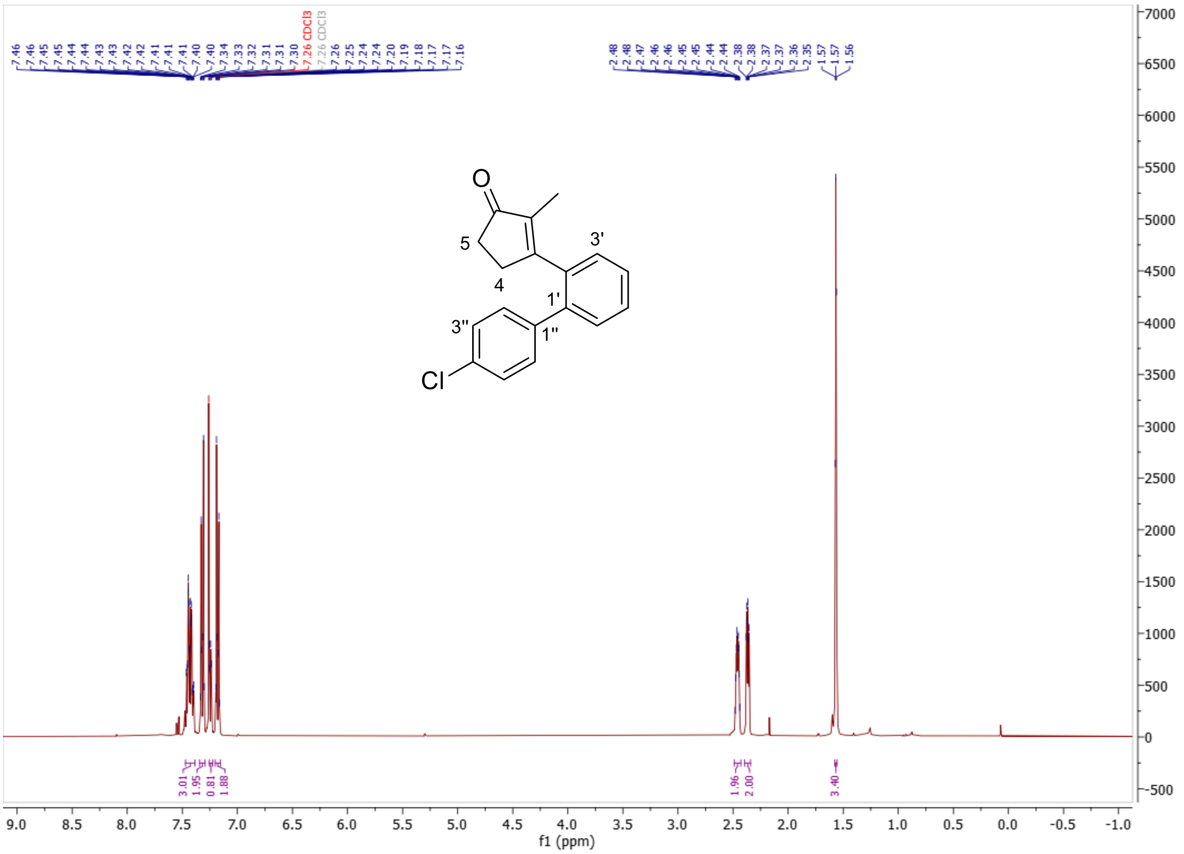

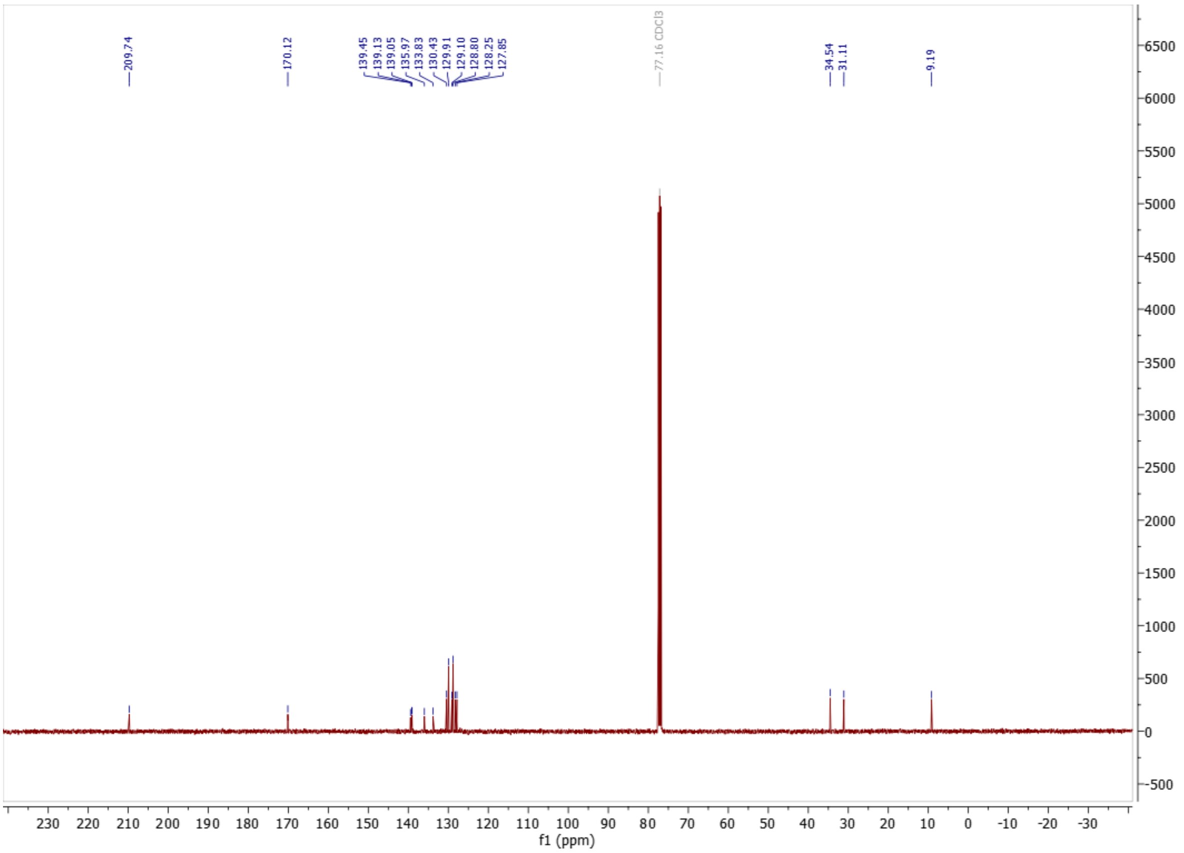

3-(3',5'-dimethyl-[1,1'-biphenyl]-2-yl)-2-methylcyclopent-2-en-1-one (9g)

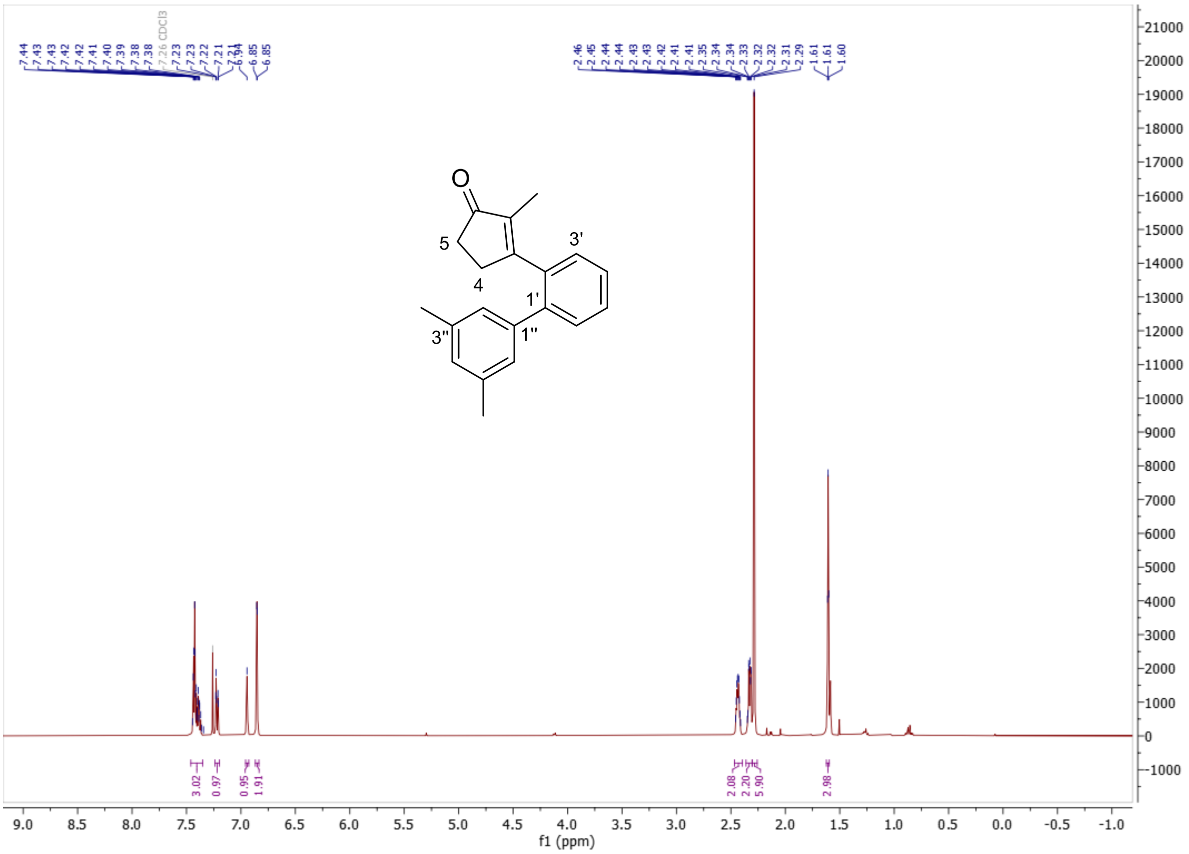

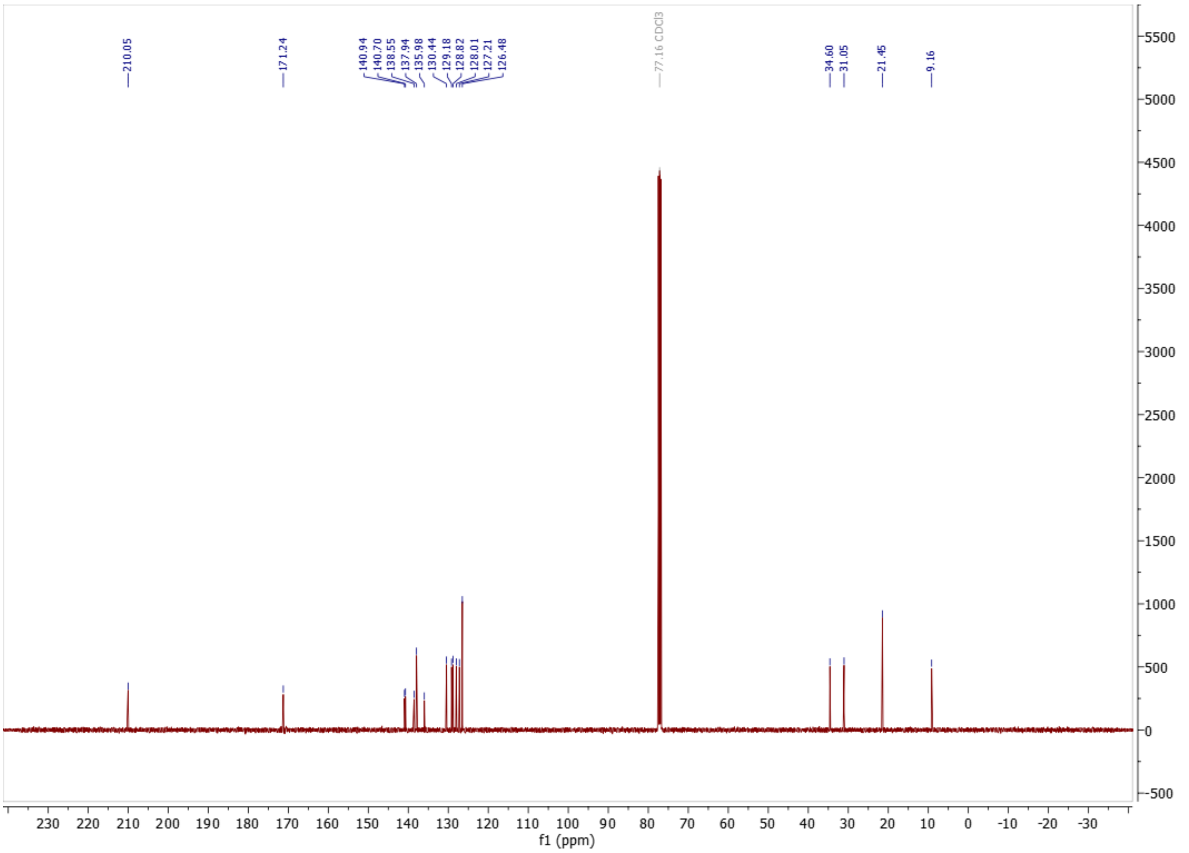

3-(3',5'-difluoro-[1,1'-biphenyl]-2-yl)-2-methylcyclopent-2-en-1-one (9h)

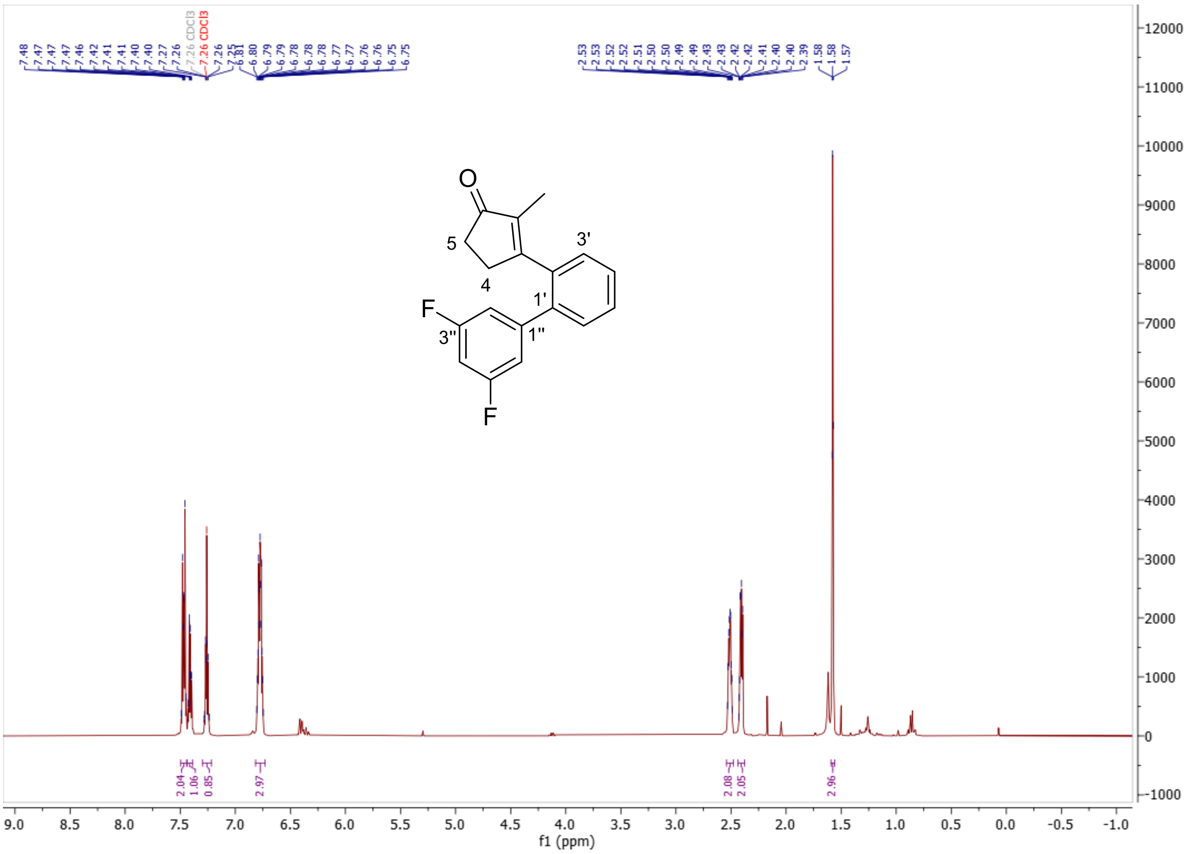

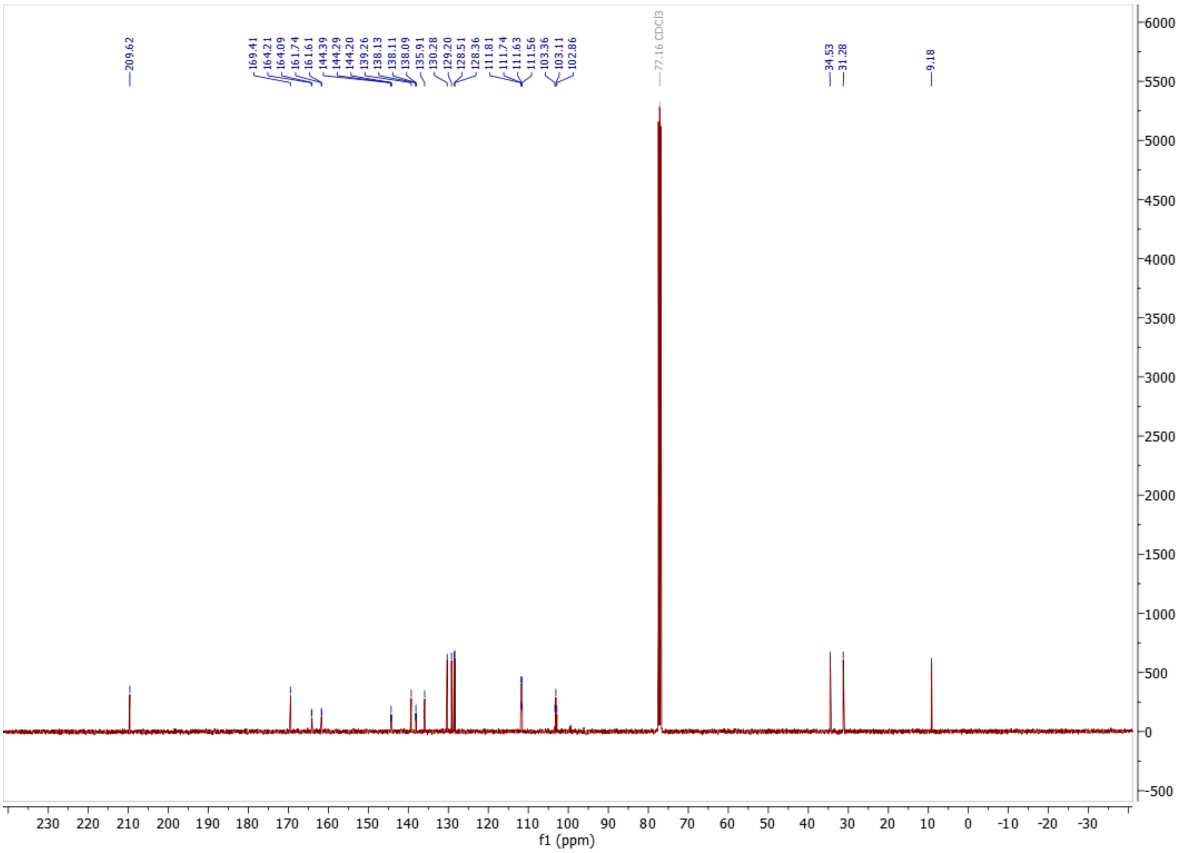

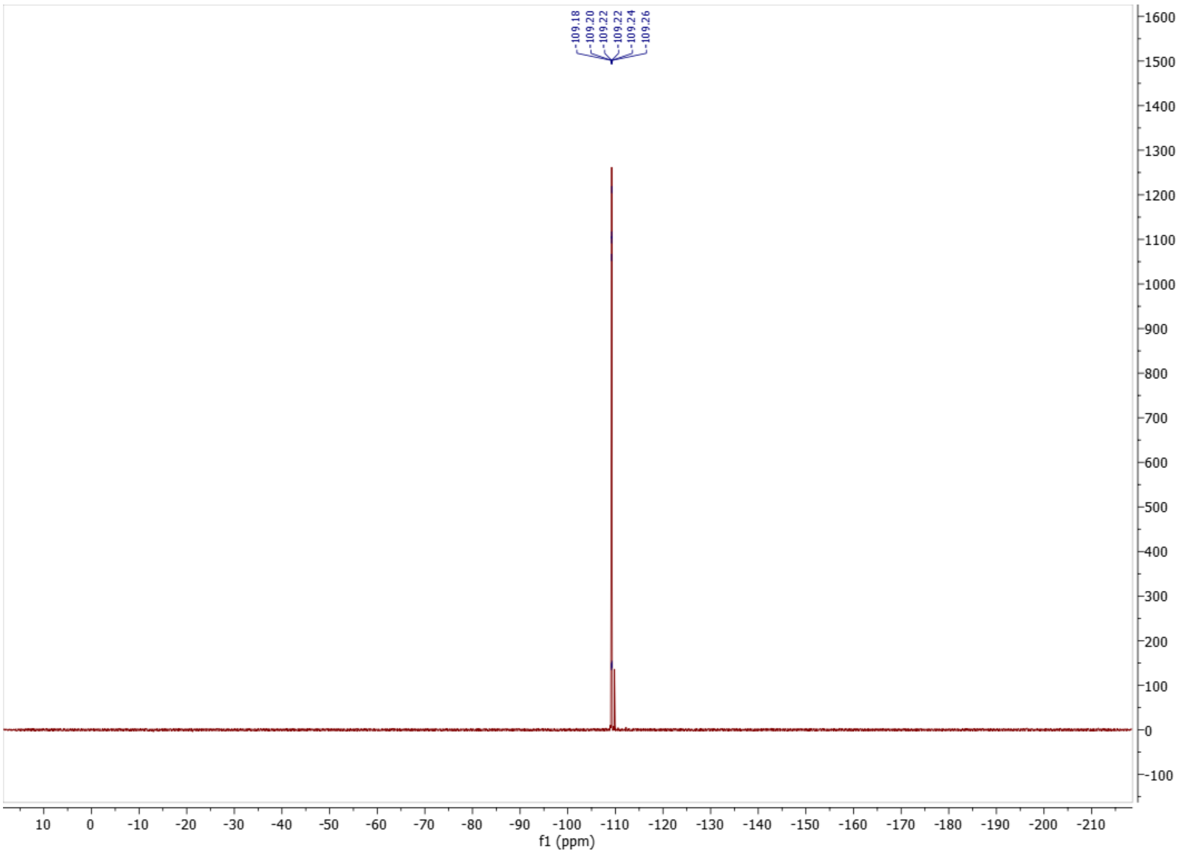

-109.18
-109.20
-109.22
-109.22
-109.24
-109.26
1600
1500
1400
1300
1200
1100
1000
900
800
700
600
500
400
300
200
100
0
-100
10 0 -10 -20 -30 -40 -50 -60 -70 -80 -90 -100 -110 -120 -130 -140 -150 -160 -170 -180 -190 -200 -210
f1 (ppm)

3-(3',5'-dichloro-[1,1'-biphenyl]-2-yl)-2-methylcyclopent-2-en-1-one (9i)

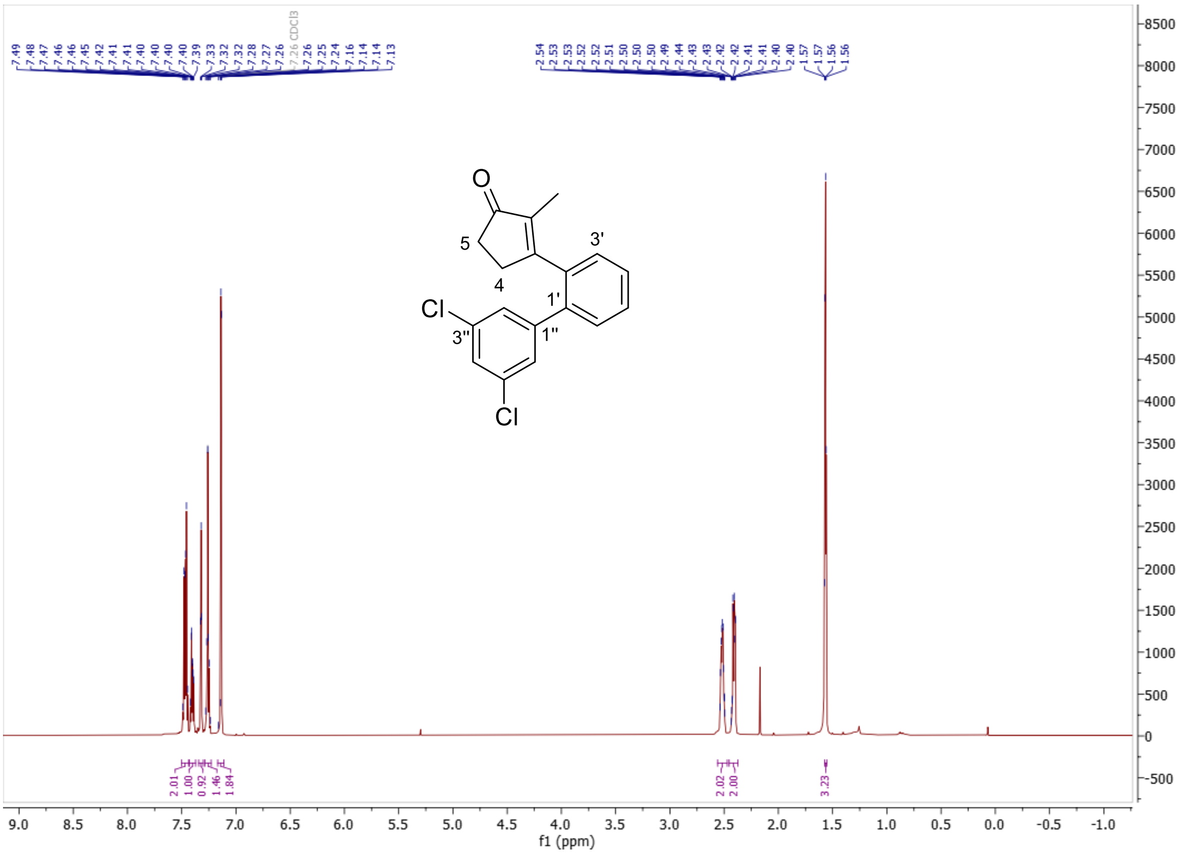

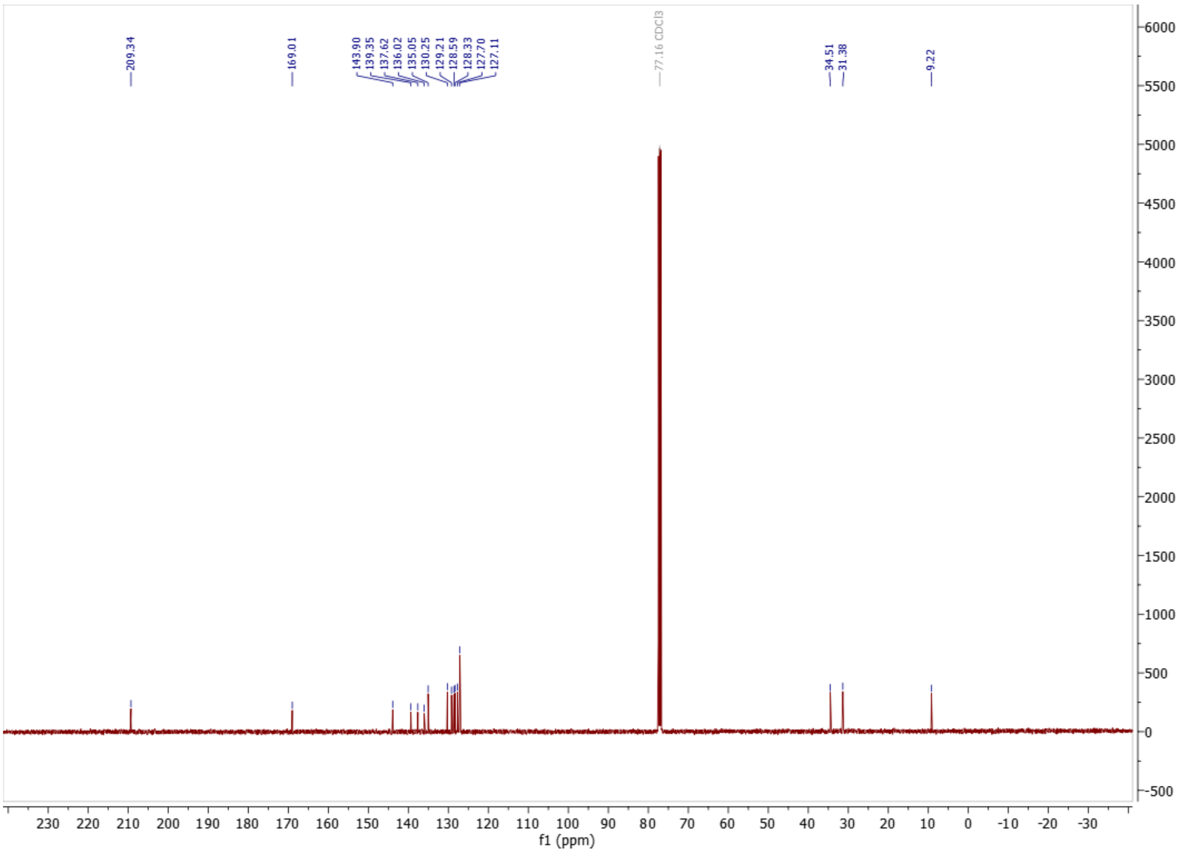

3-(3',5'-dimethoxy-[1,1'-biphenyl]-2-yl)-2-methylcyclopent-2-en-1-one (9j)

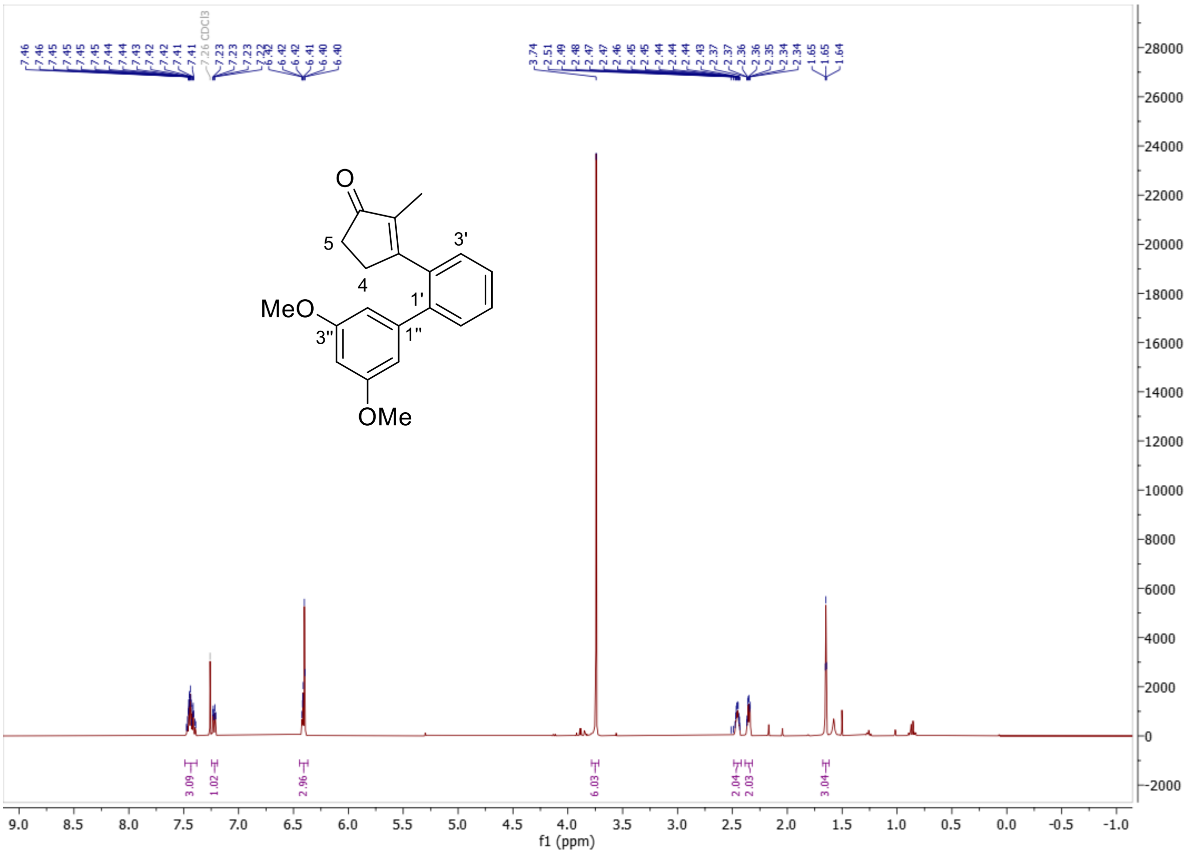

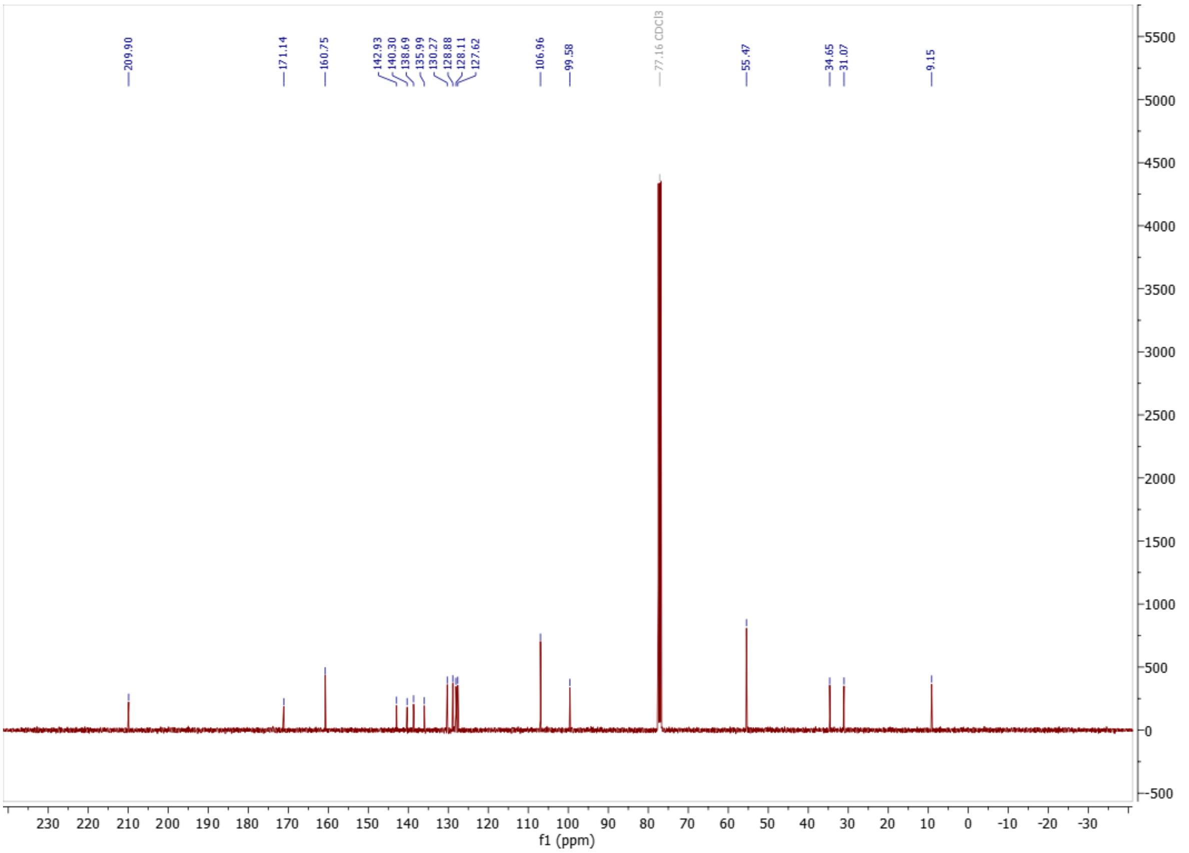

2-methyl-3-(3'-(trifluoromethyl)-[1,1'-biphenyl]-2-yl)cyclopent-2-en-1-one (9k)

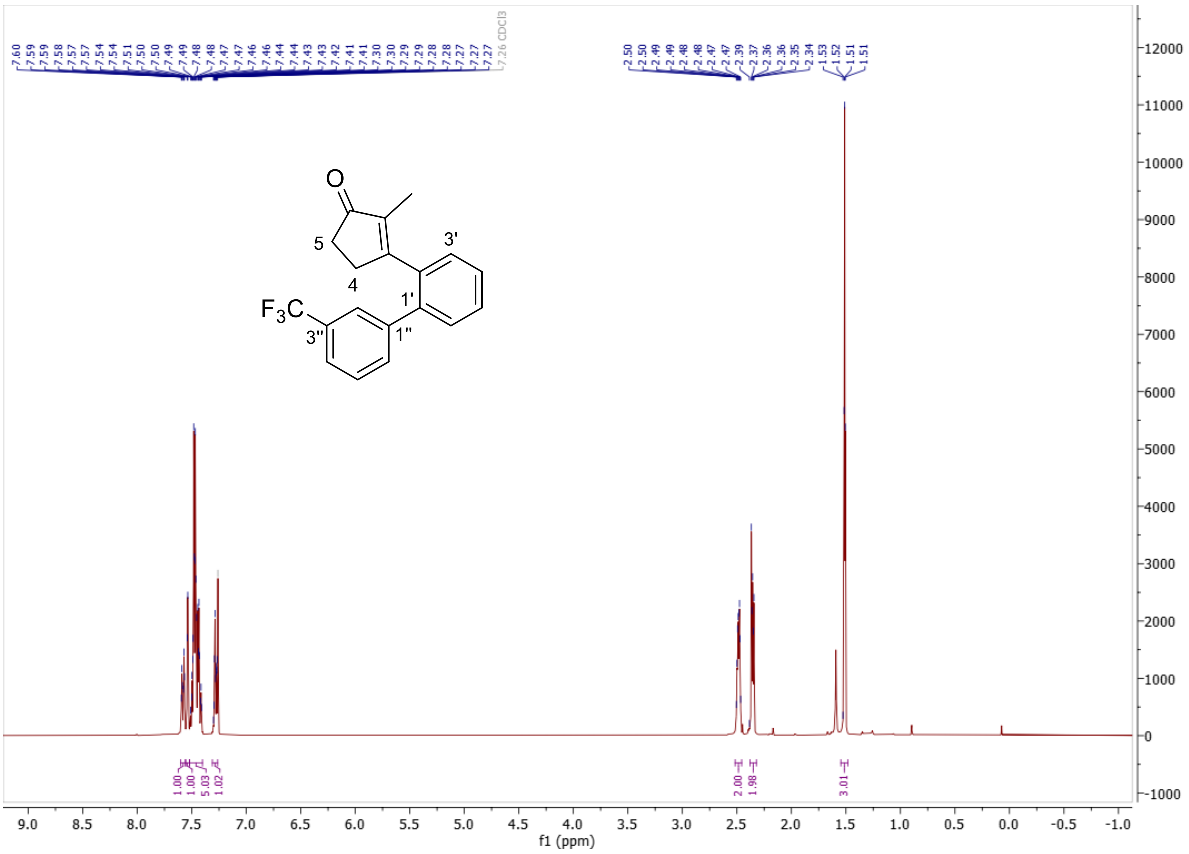

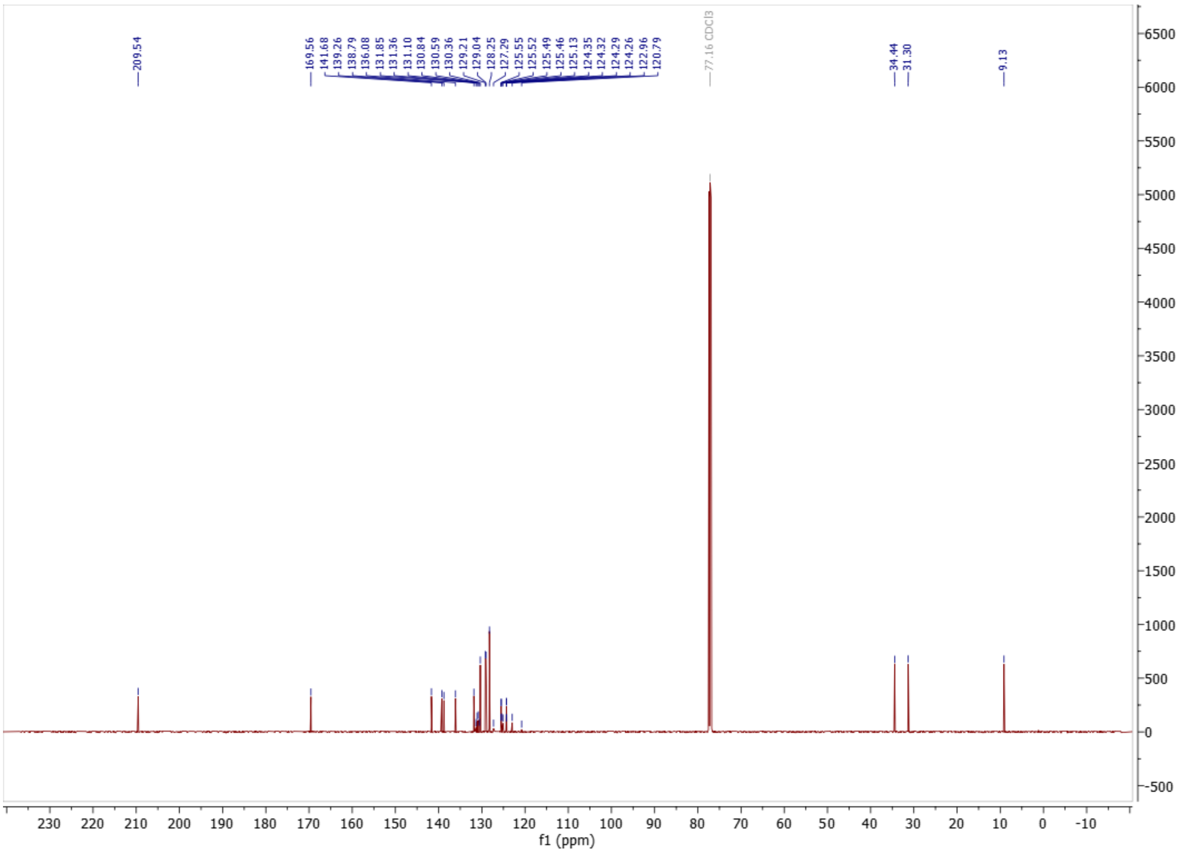

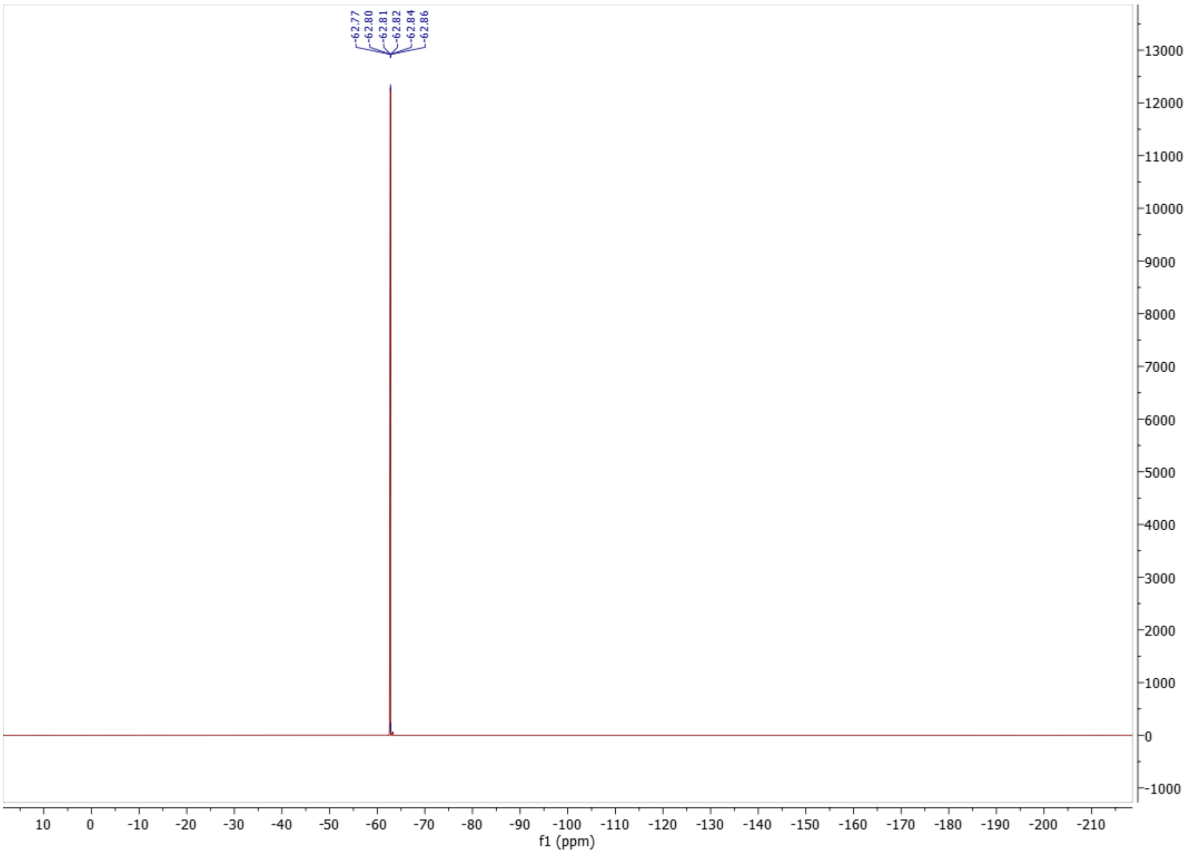

-62.77
-62.80
-62.81
-62.82
-62.84
-62.86
f1 (ppm)

3-([1,1':3',1''-terphenyl]-2-yl)-2-methylcyclopent-2-en-1-one (9l)

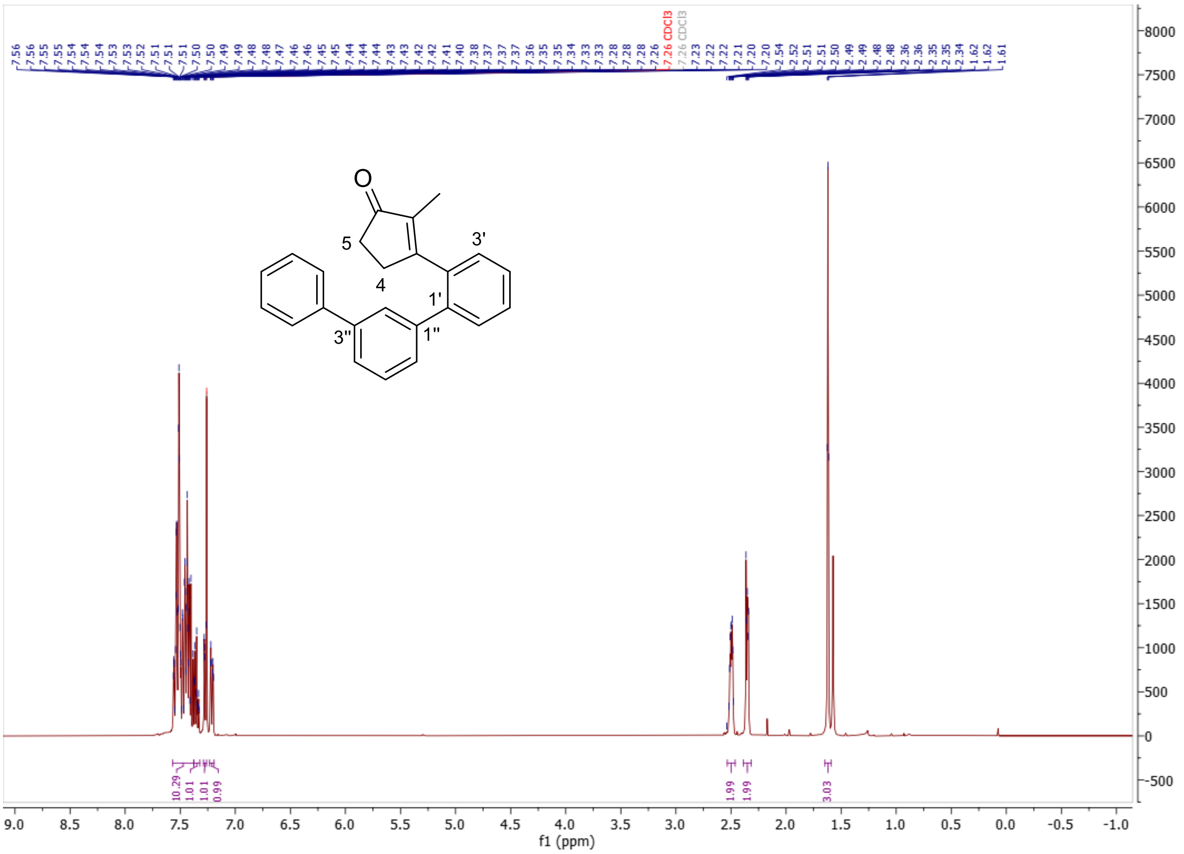

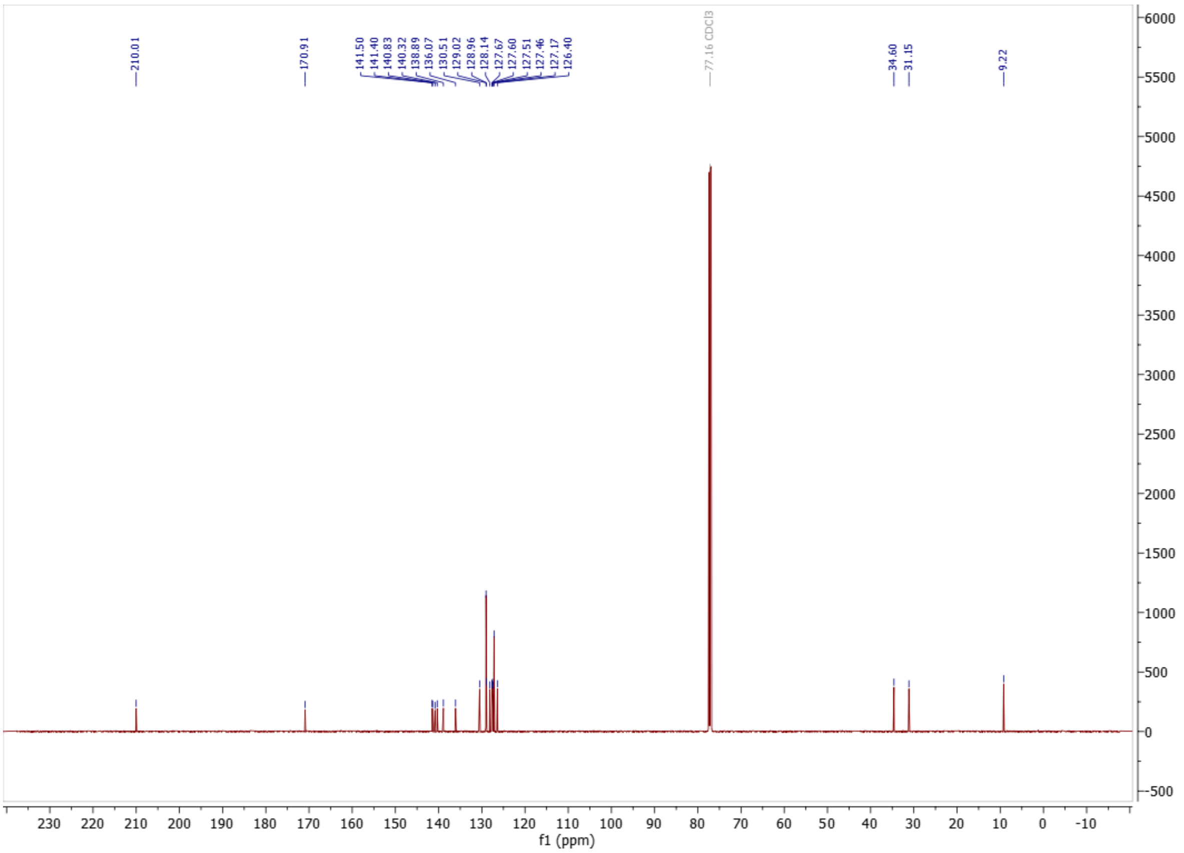

2-methyl-3-(3'-methyl-[1,1'-biphenyl]-2-yl)cyclopent-2-en-1-one (9m)

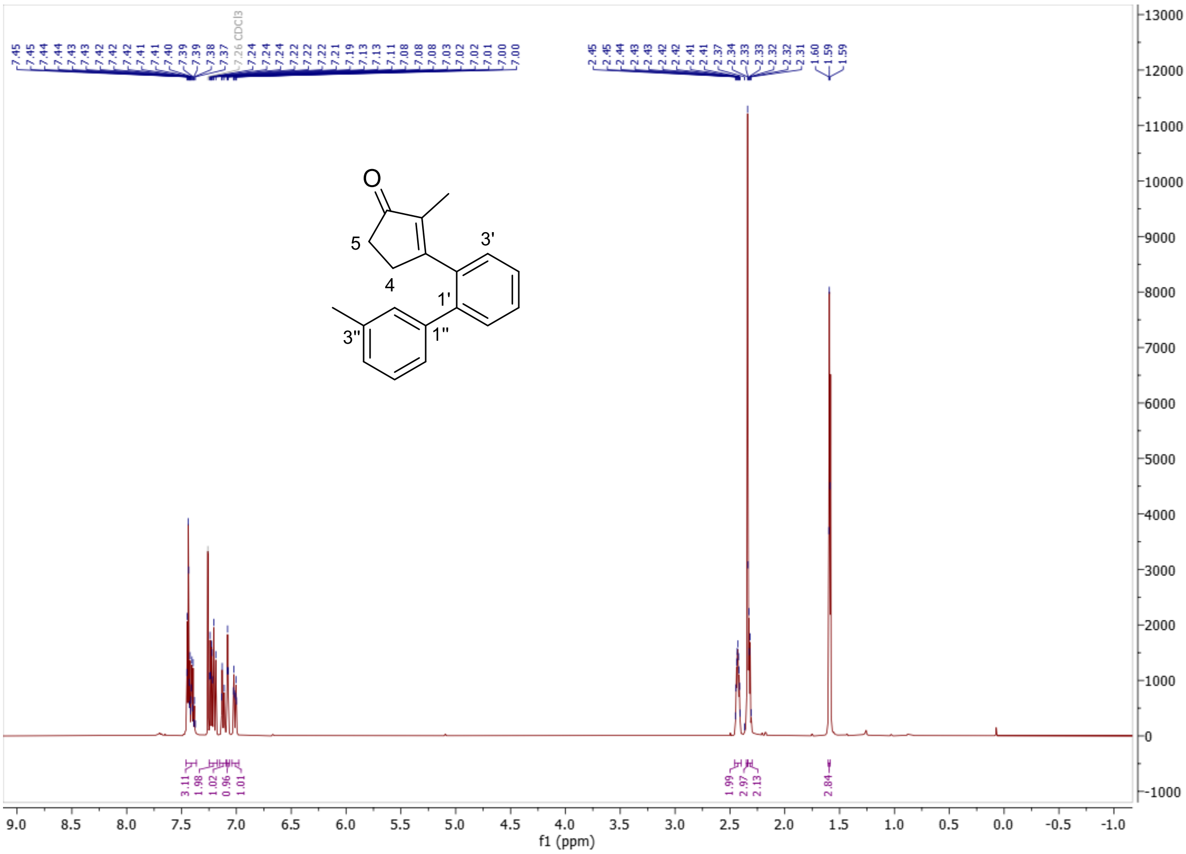

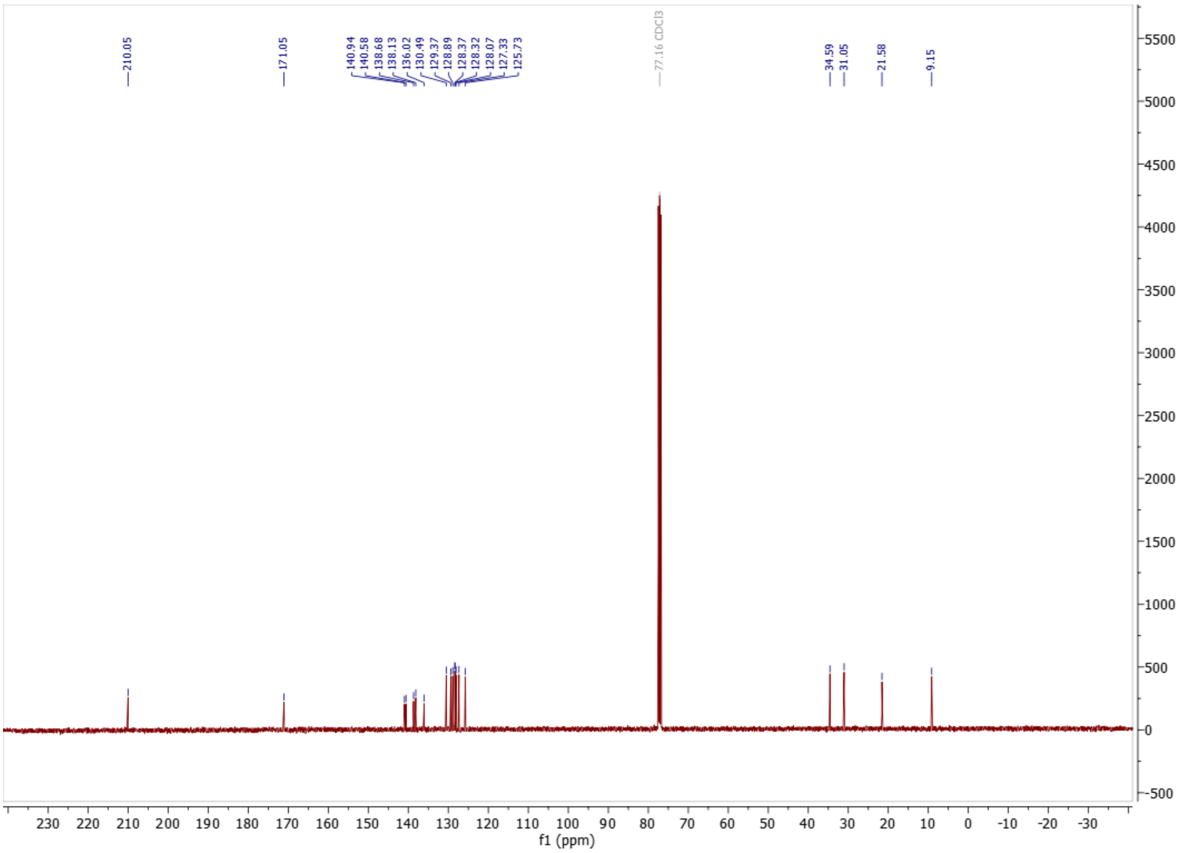

3-(3'-fluoro-[1,1'-biphenyl]-2-yl)-2-methylcyclopent-2-en-1-one (9n)

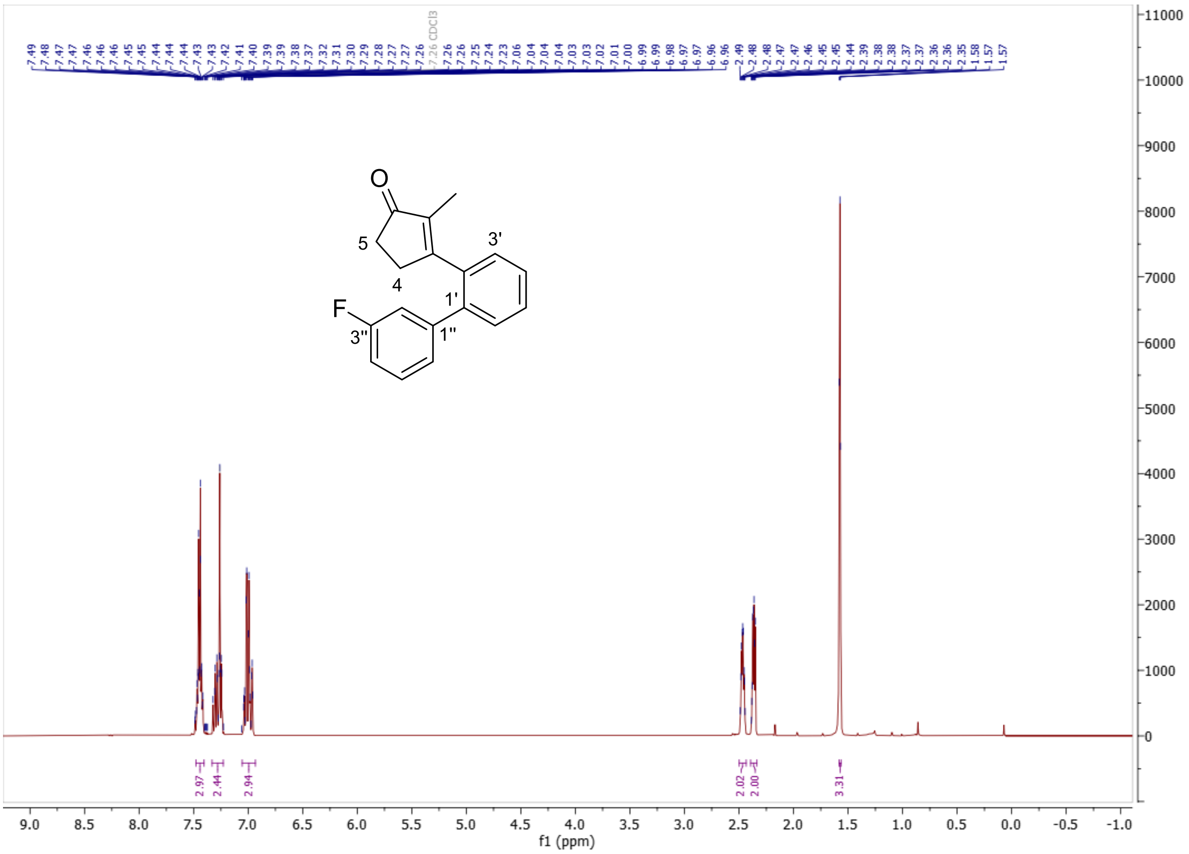

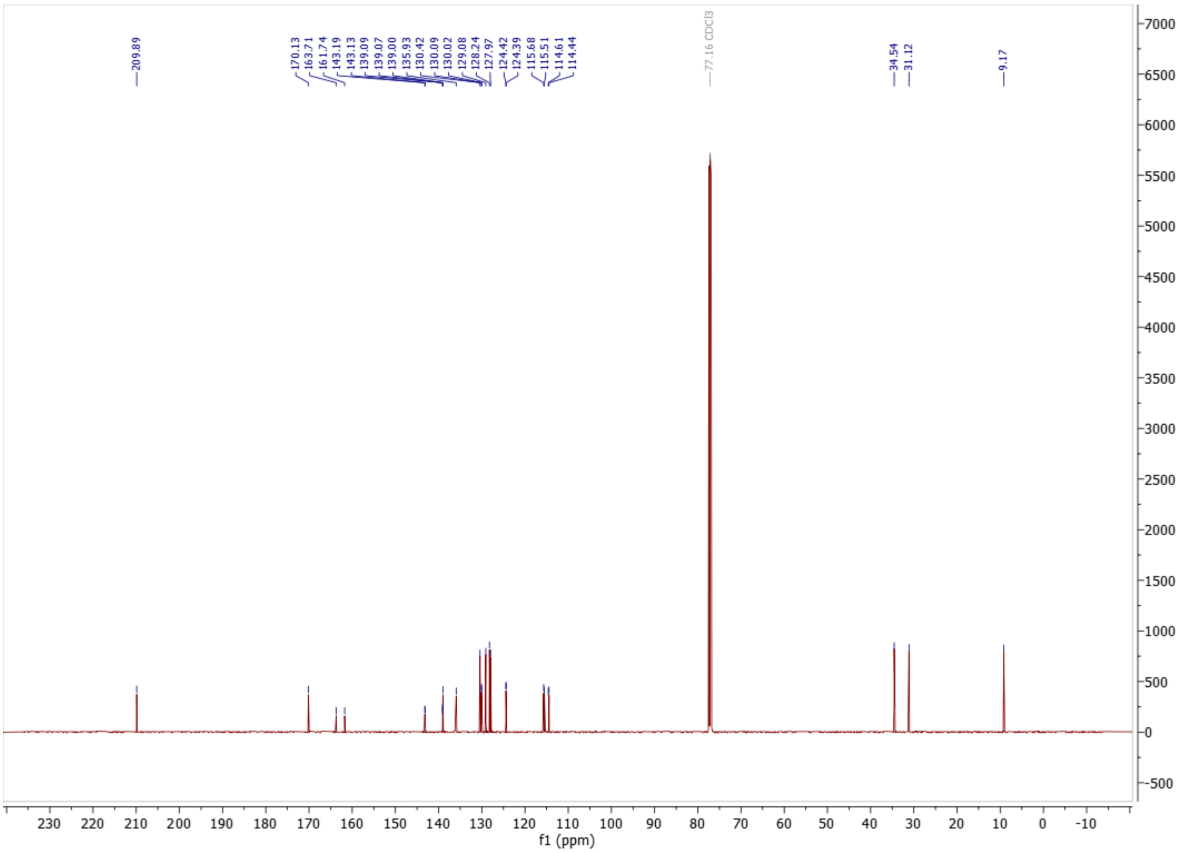

3-(3'-chloro-[1,1'-biphenyl]-2-yl)-2-methylcyclopent-2-en-1-one (9o)

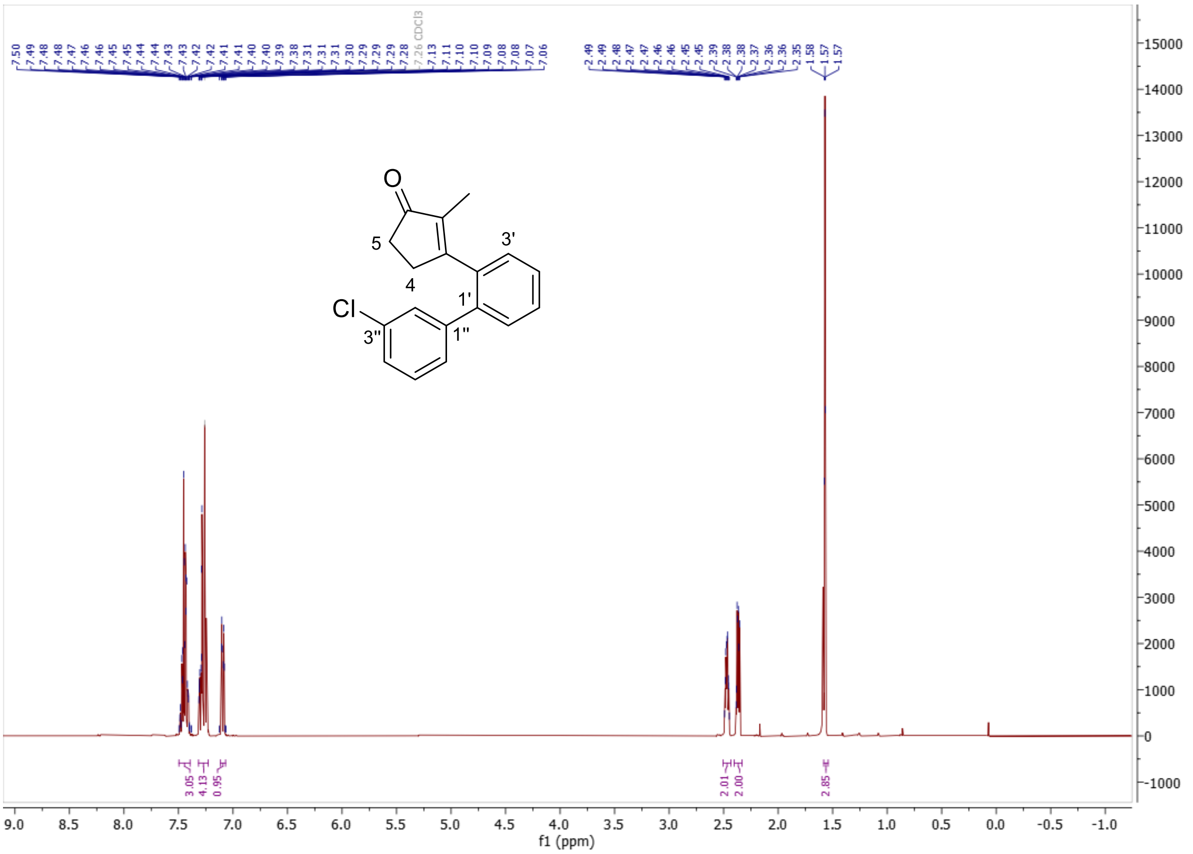

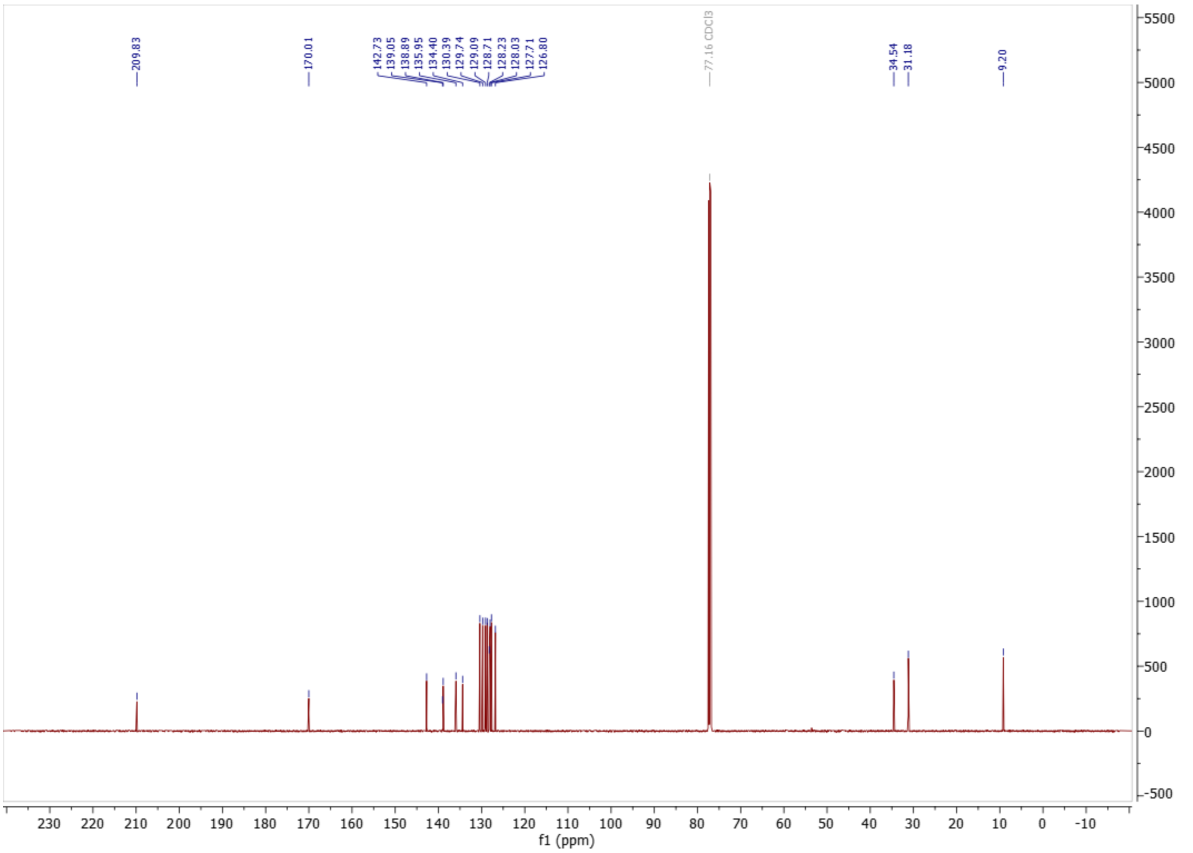

3-(3'-methoxy-[1,1'-biphenyl]-2-yl)-2-methylcyclopent-2-en-1-one (9p)

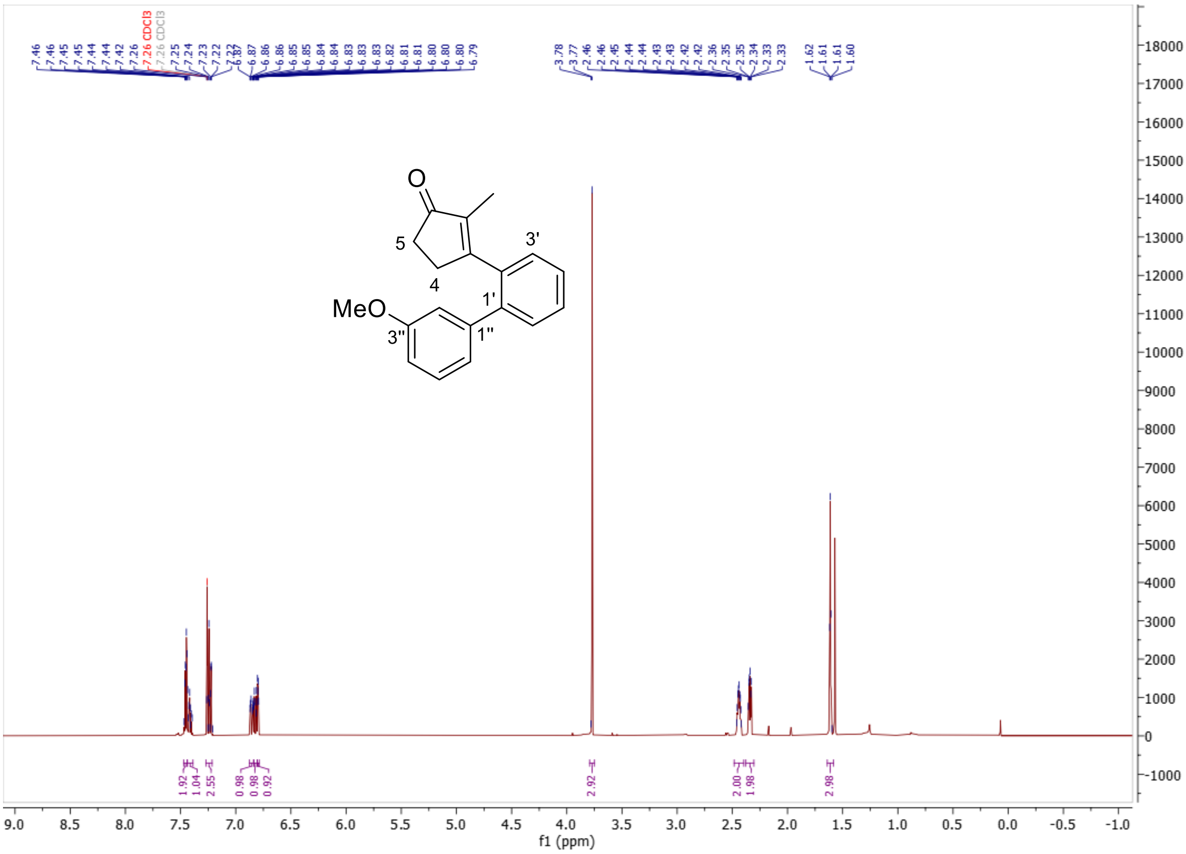

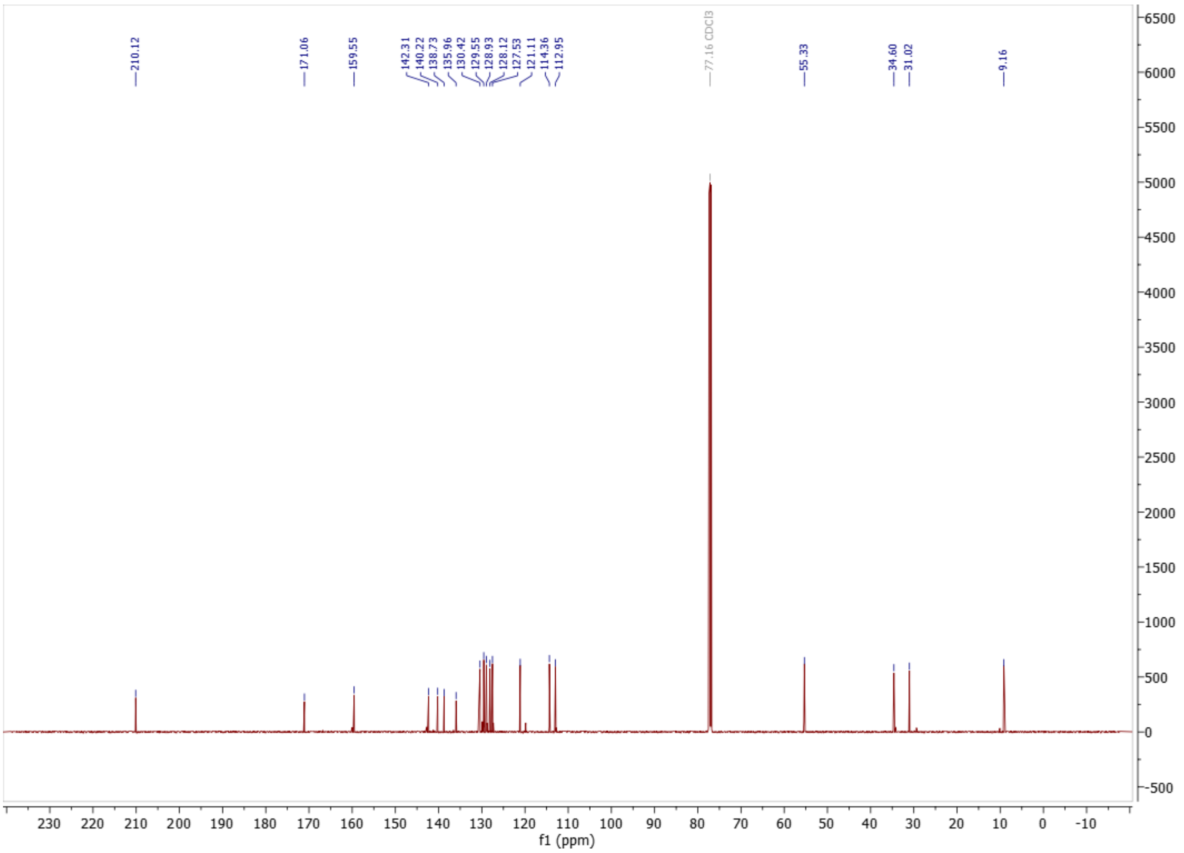

(3a*R*,11b*S*)-11b-Methyl-2,3,3a,11b-tetrahydro-1*H*-cyclopenta[*l*]phenanthren-1-one-3a,8,9,10,11-*d*$_5$ (11a-*d*$_5$) and (3a*R*,11b*S*)-11b-methyl-2,3,3a,11b-tetrahydro-1*H*-cyclopenta[*l*]phenanthren-1-one-8,9,10,11-*d*$_5$ (11a-*d*$_4$)

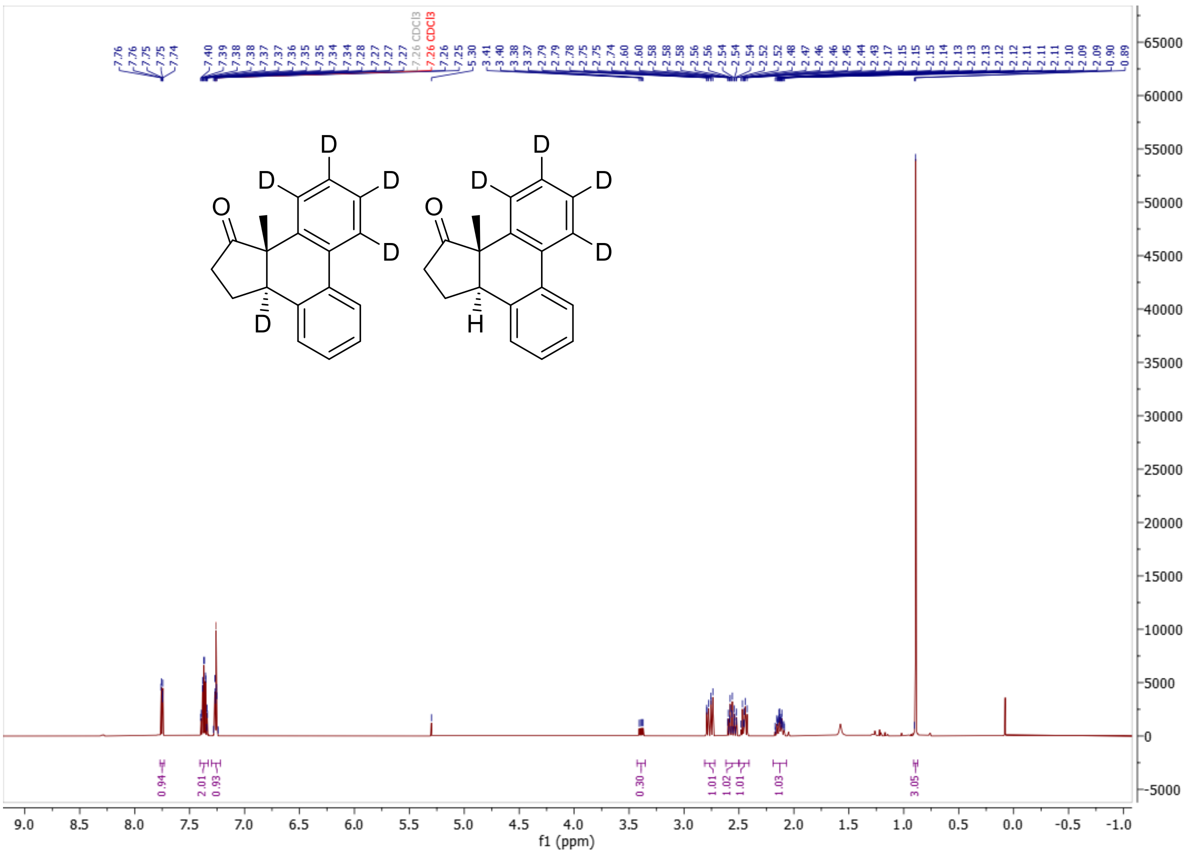

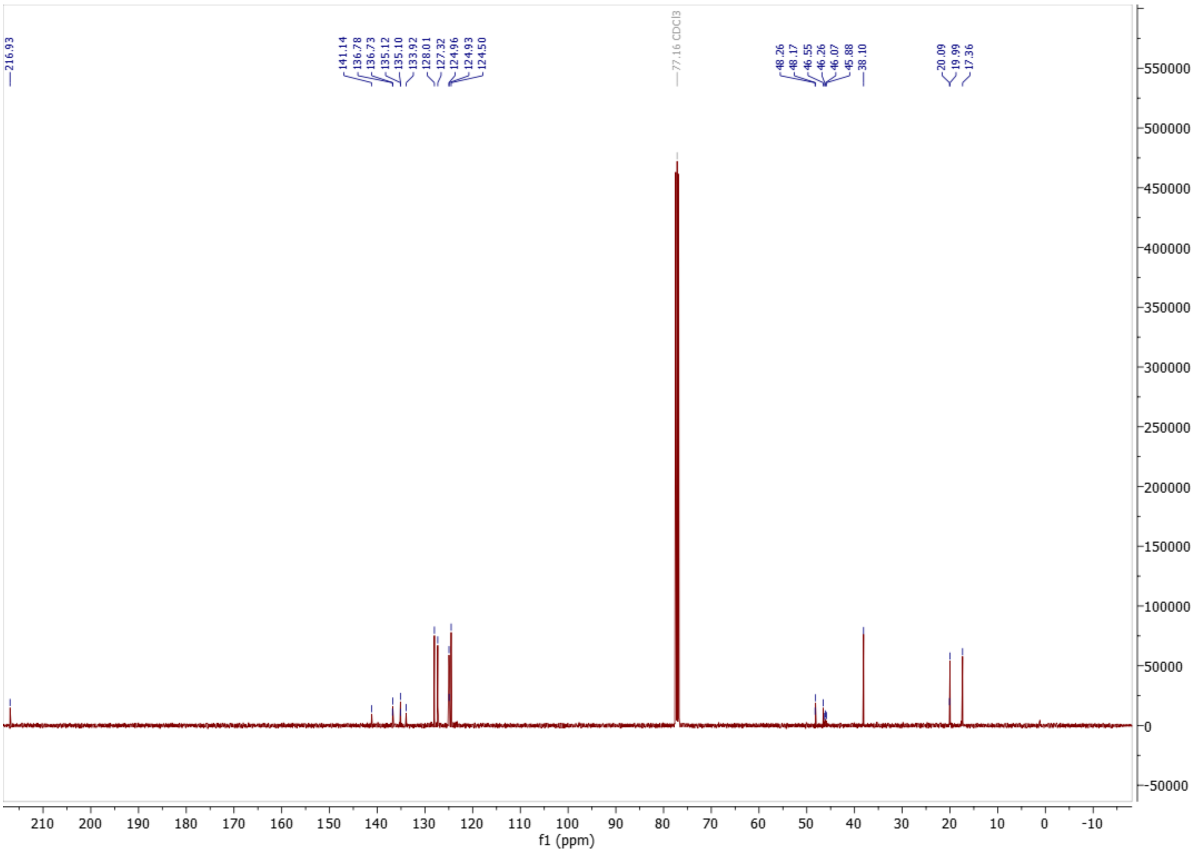

(3a*R*,11b*S*)-10,11b-dimethyl-2,3,3a,11b-tetrahydro-1*H*-cyclopenta[*l*]phenanthren-1-one (11b)

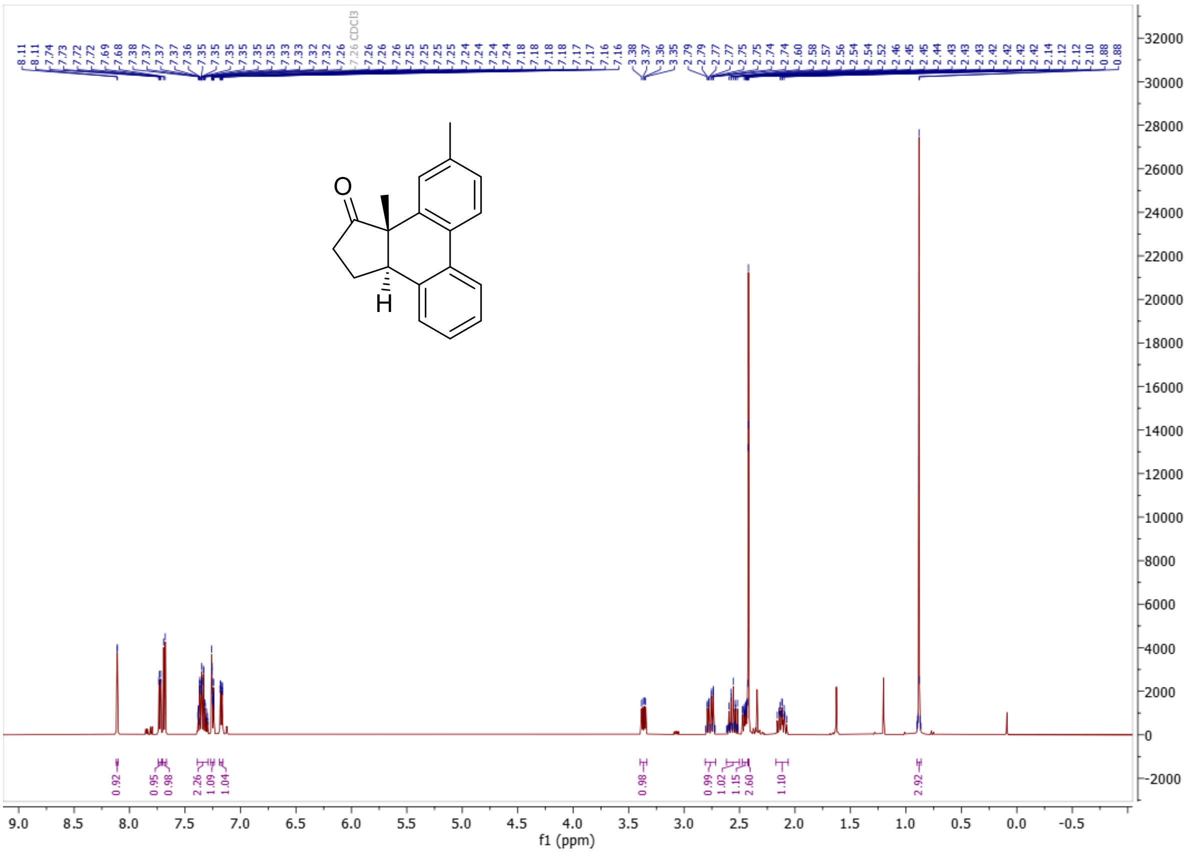

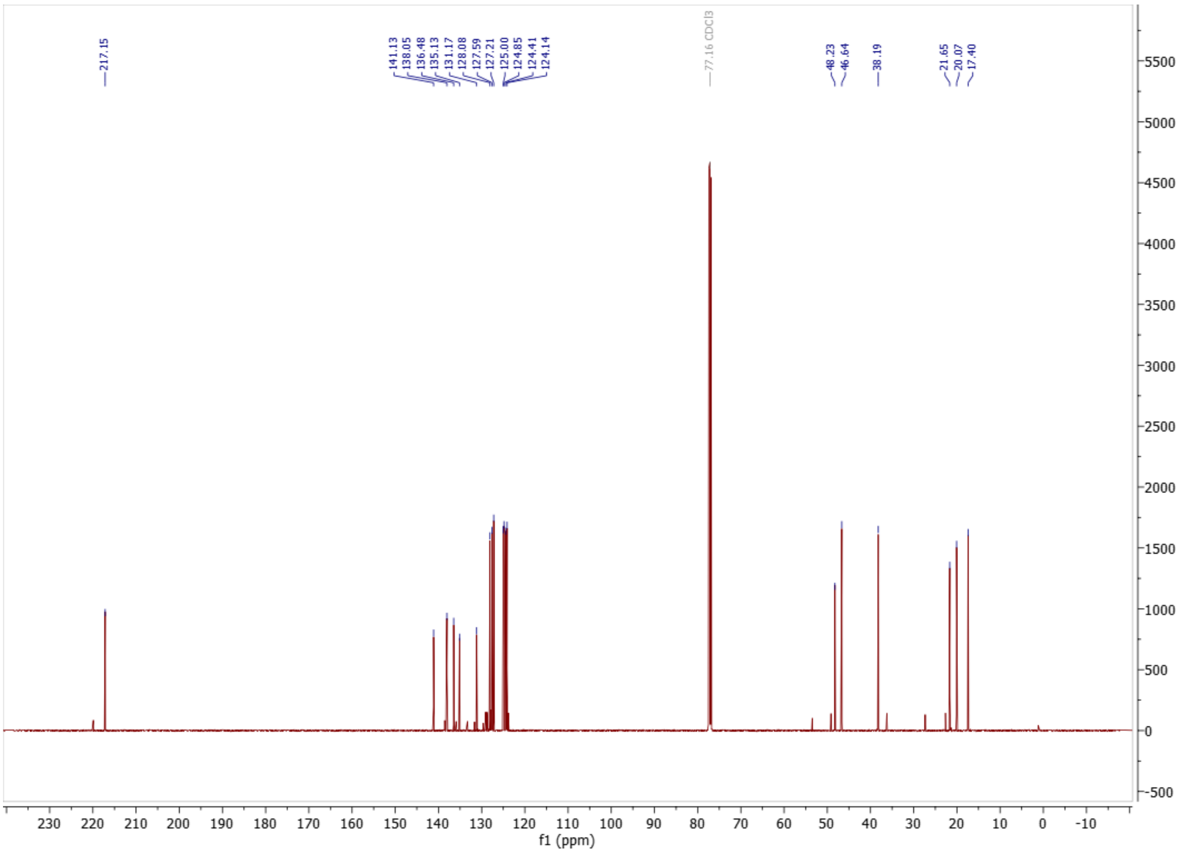

(1*R*,3a*R*,11b*S*)-10,11b-dimethyl-2,3,3a,11b-tetrahydro-1*H*-cyclopenta[*l*]phenanthren-1-ol (12b)

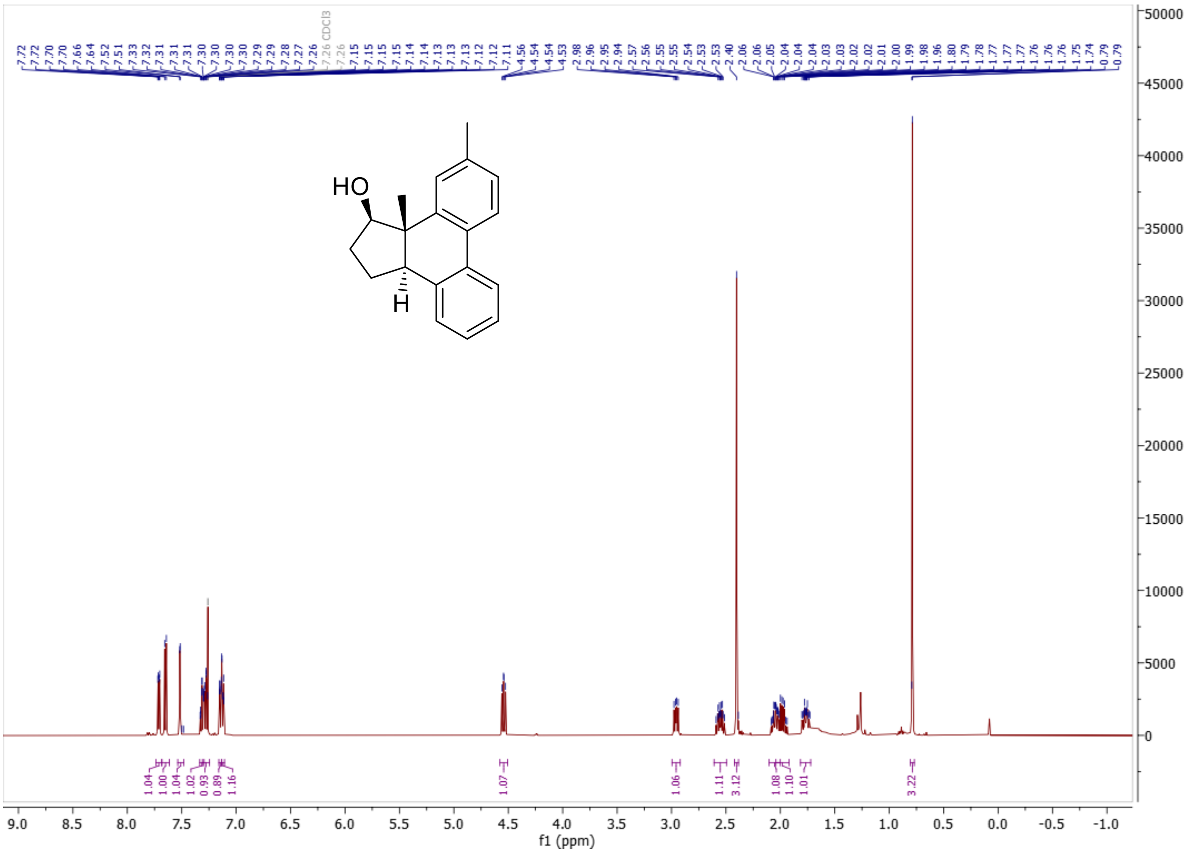

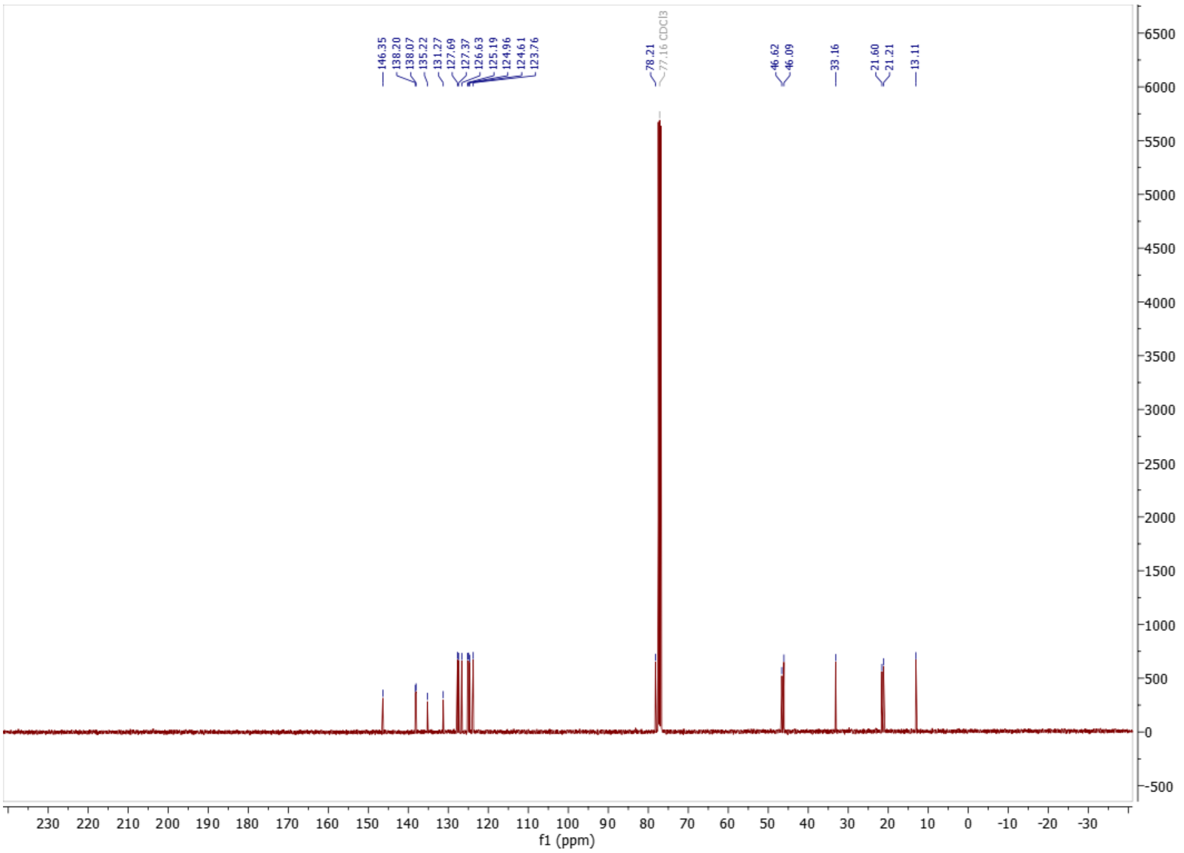

(3a*R*,11b*S*)-10-fluoro-11b-methyl-2,3,3a,11b-tetrahydro-1*H*-cyclopenta[*l*]phenanthren-1-one (11c)

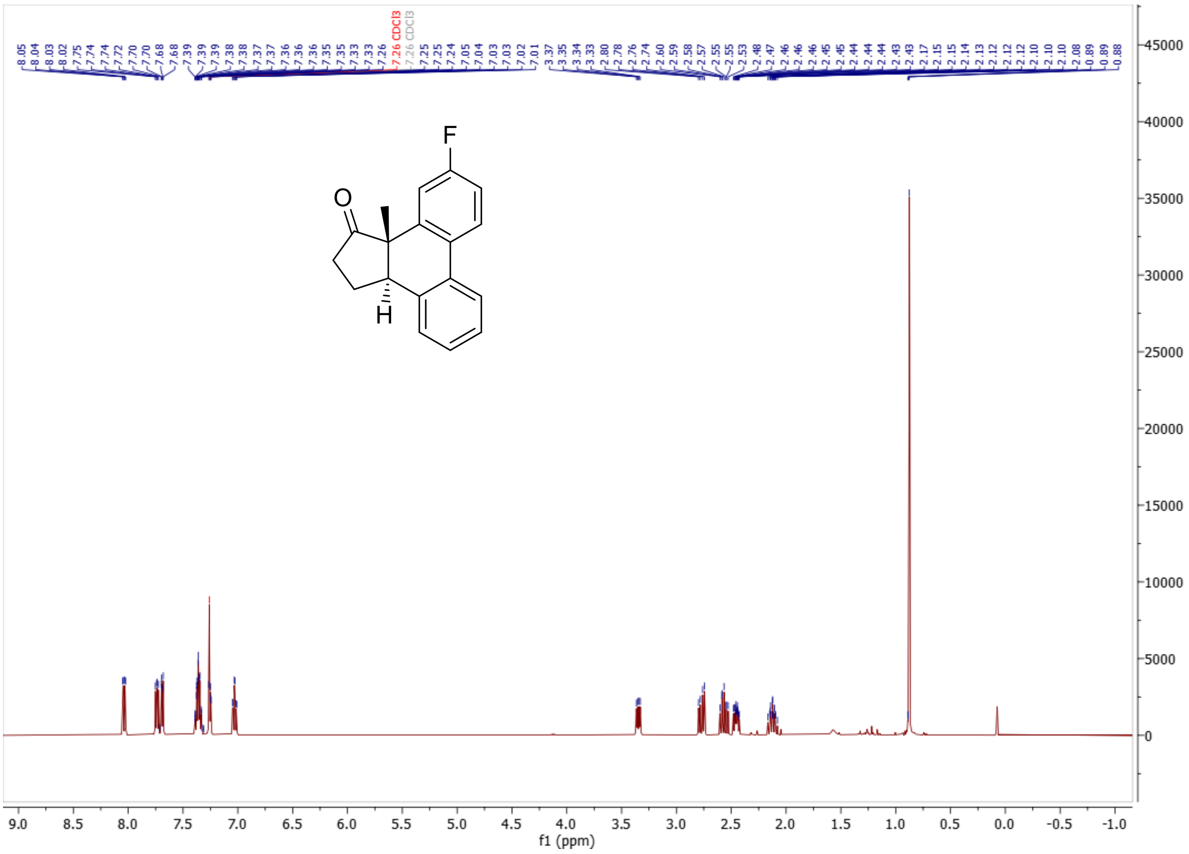

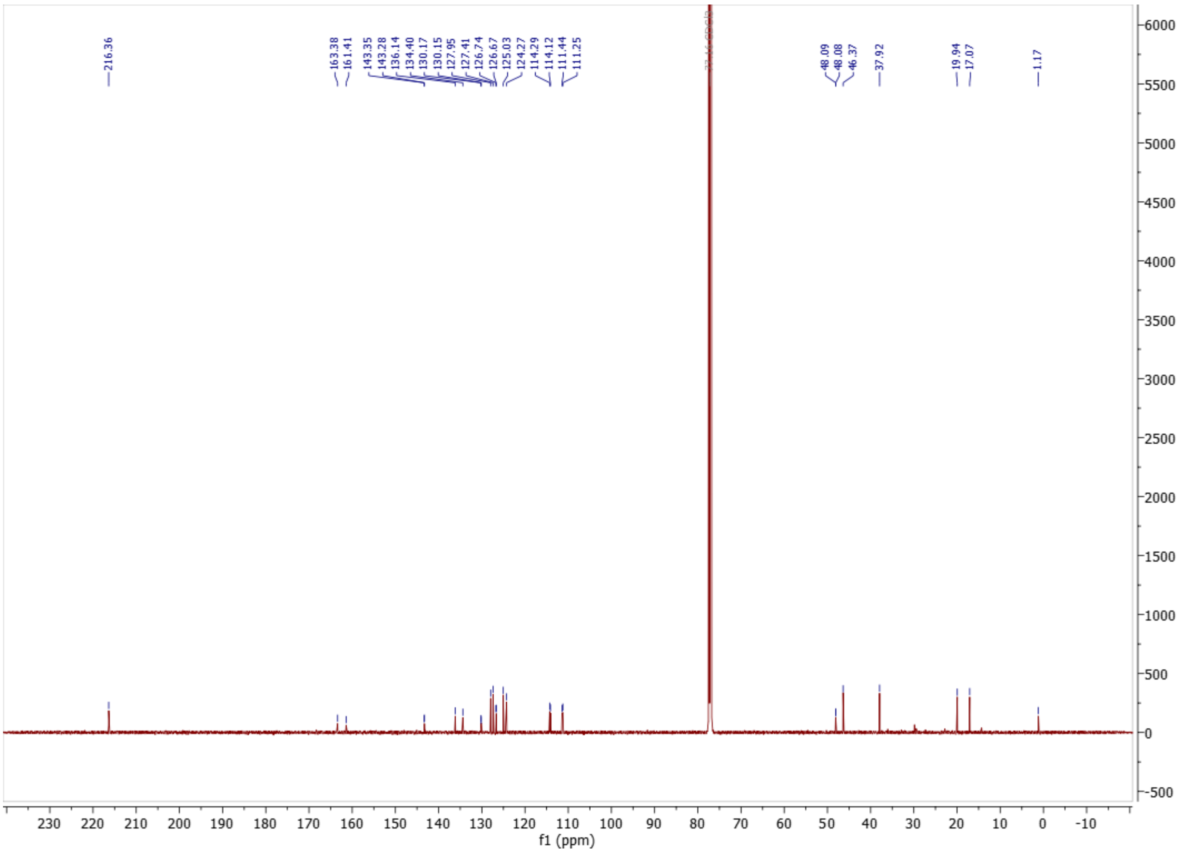

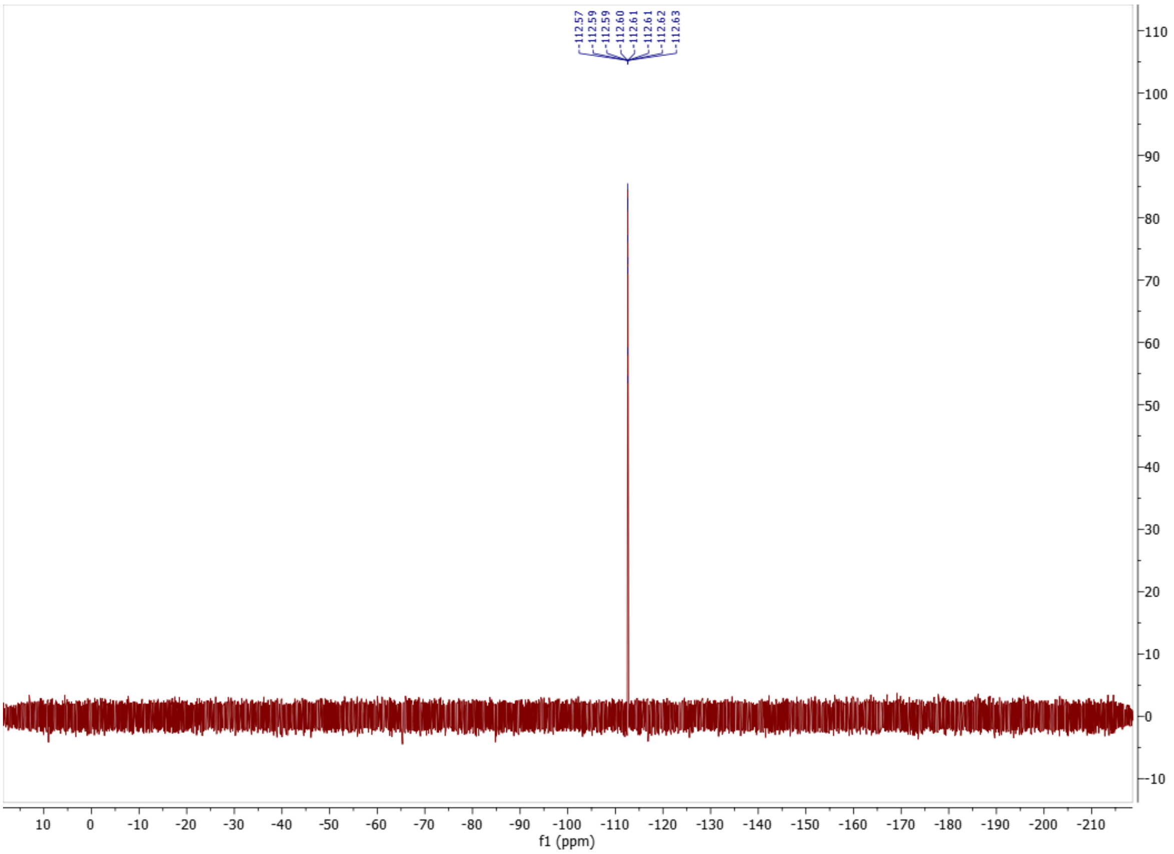

-112.57
-112.59
-112.59
-112.60
-112.61
-112.61
-112.62
-112.63
110
100
90
80
70
60
50
40
30
20
10
0
-10
10
0
-10
-20
-30
-40
-50
-60
-70
-80
-90
-100
-110
-120
-130
-140
-150
-160
-170
-180
-190
-200
-210
f1 (ppm)

(3a*R*,11b*S*)-11b-methyl-10-(trifluoromethyl)-2,3,3a,11b-tetrahydro-1*H*-cyclopenta[*l*]phenanthren-1-one (11d)

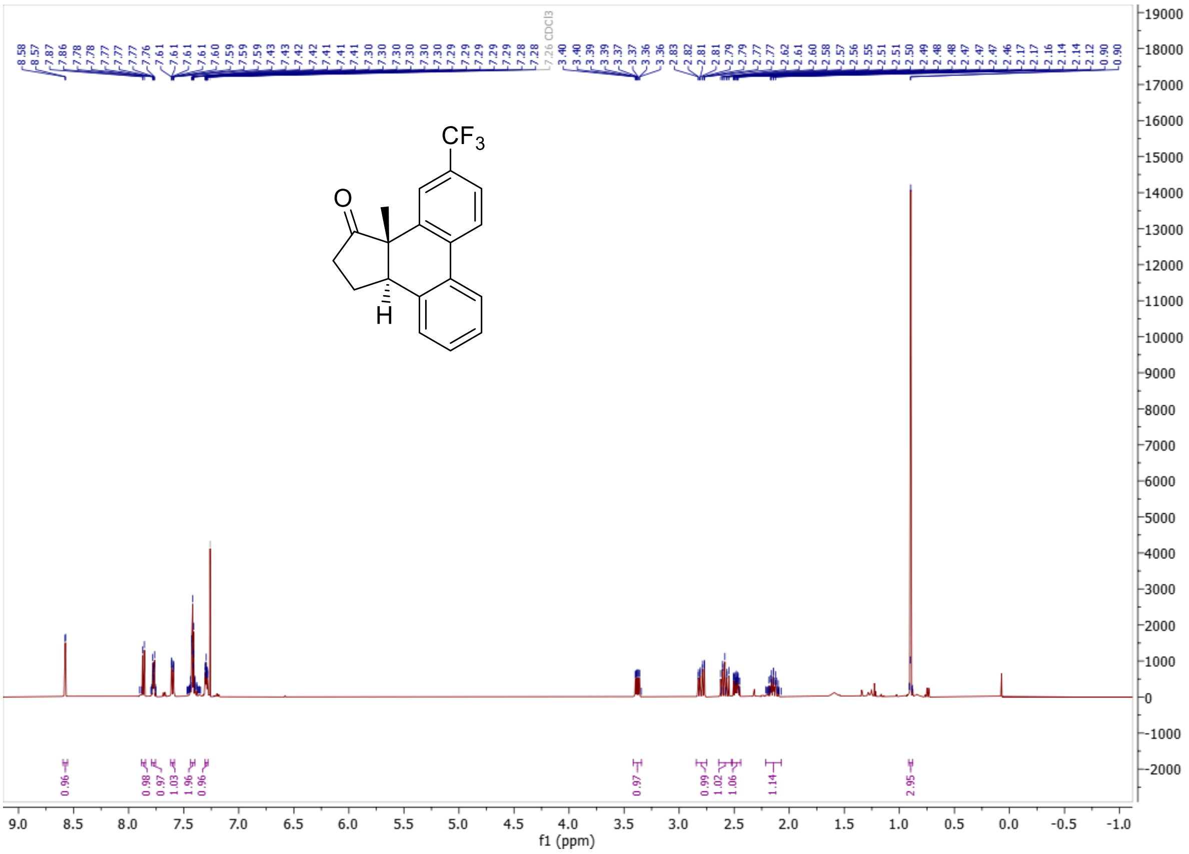

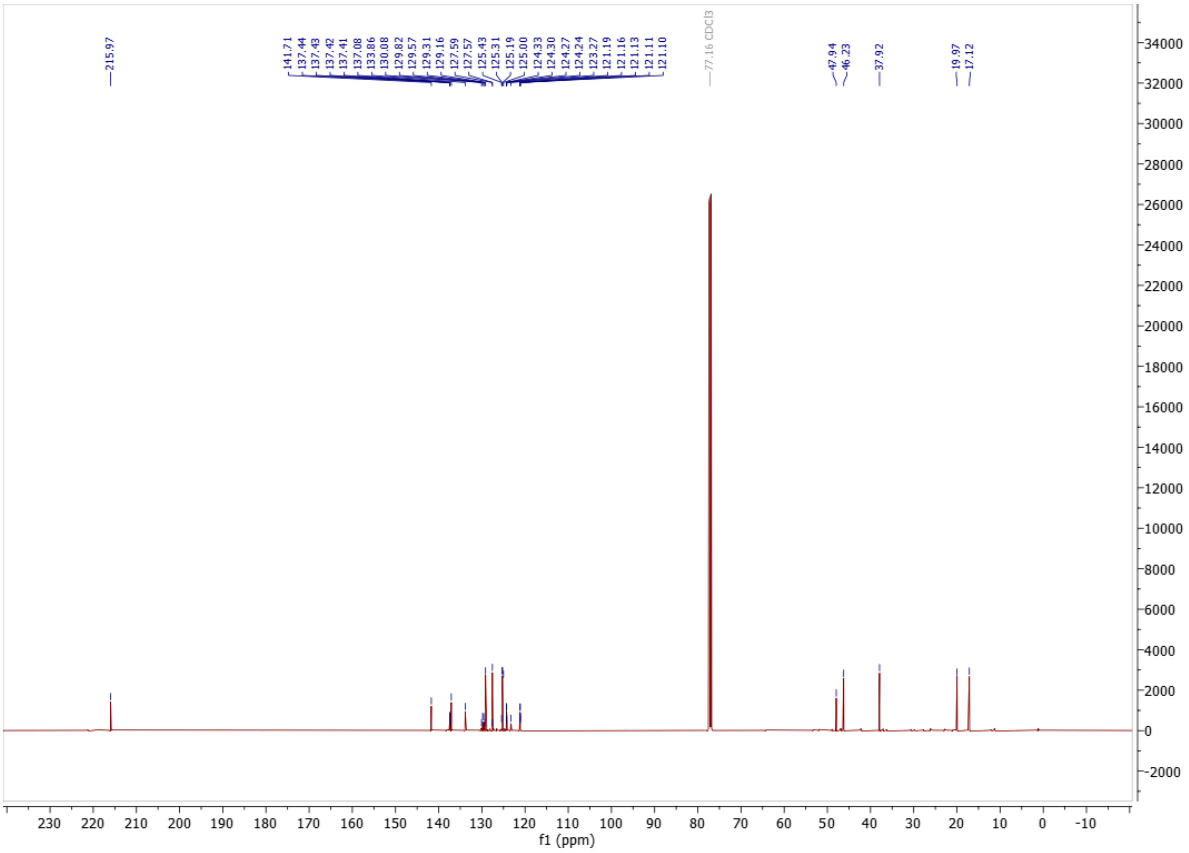

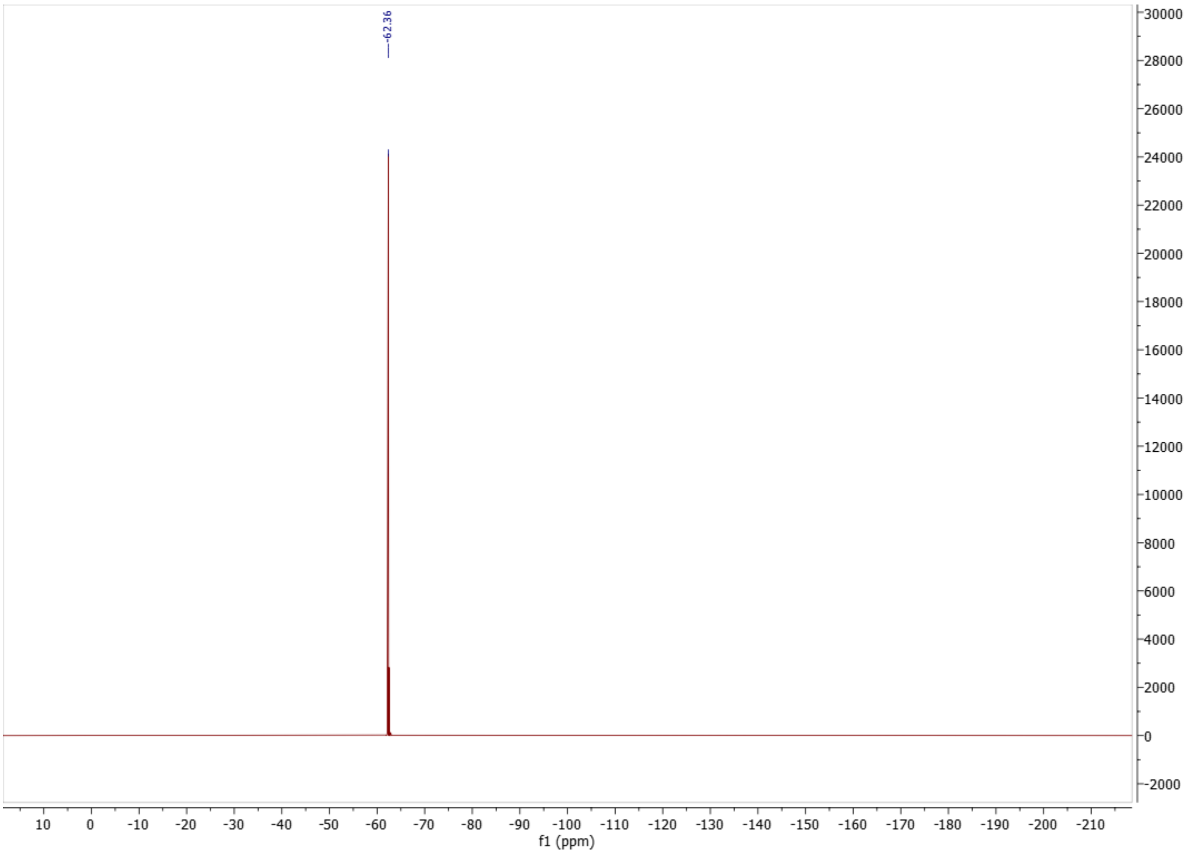

-62.36
30000
28000
26000
24000
22000
20000
18000
16000
14000
12000
10000
8000
6000
4000
2000
0
-2000
10 0 -10 -20 -30 -40 -50 -60 -70 -80 -90 -100 -110 -120 -130 -140 -150 -160 -170 -180 -190 -200 -210
f1 (ppm)

(3a*R*,11b*S*)-10-(*tert*-butyl)-11b-methyl-2,3,3a,11b-tetrahydro-1*H*-cyclopenta[*l*]phenanthren-1-one (11e)

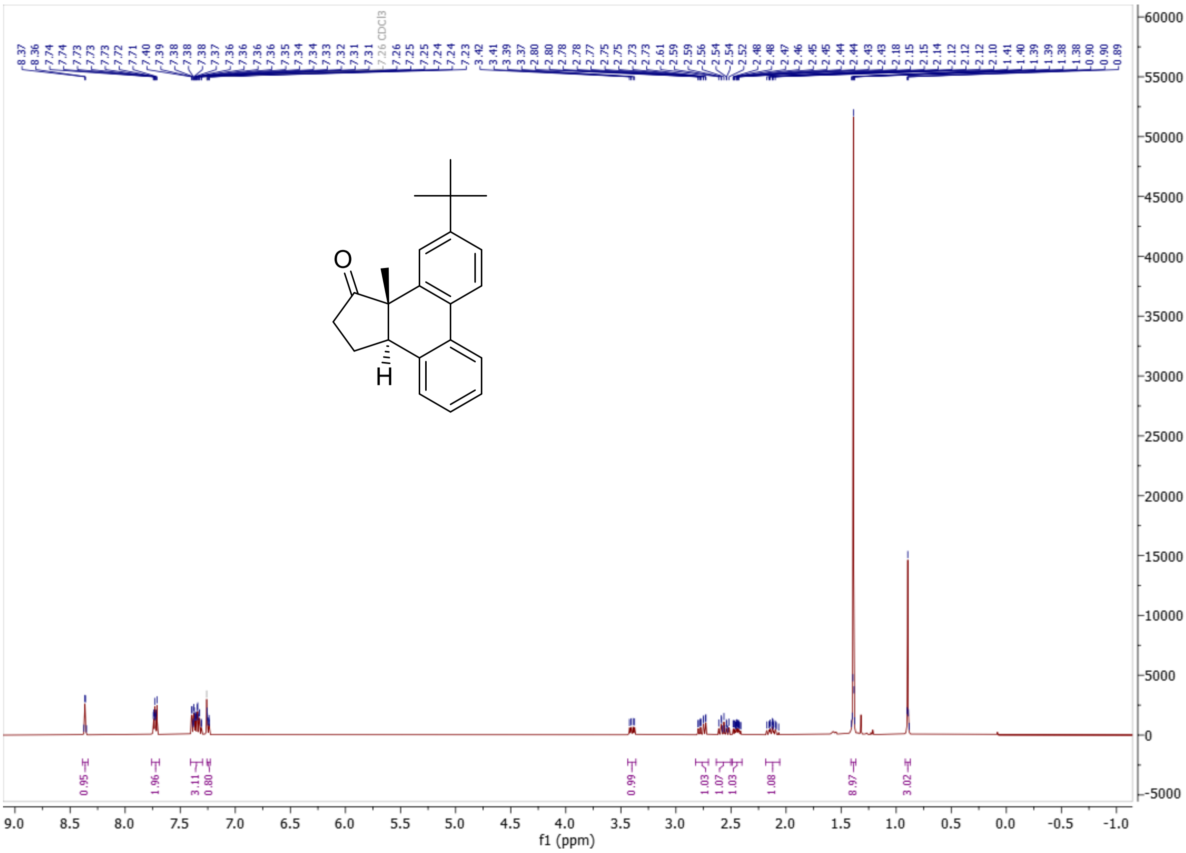

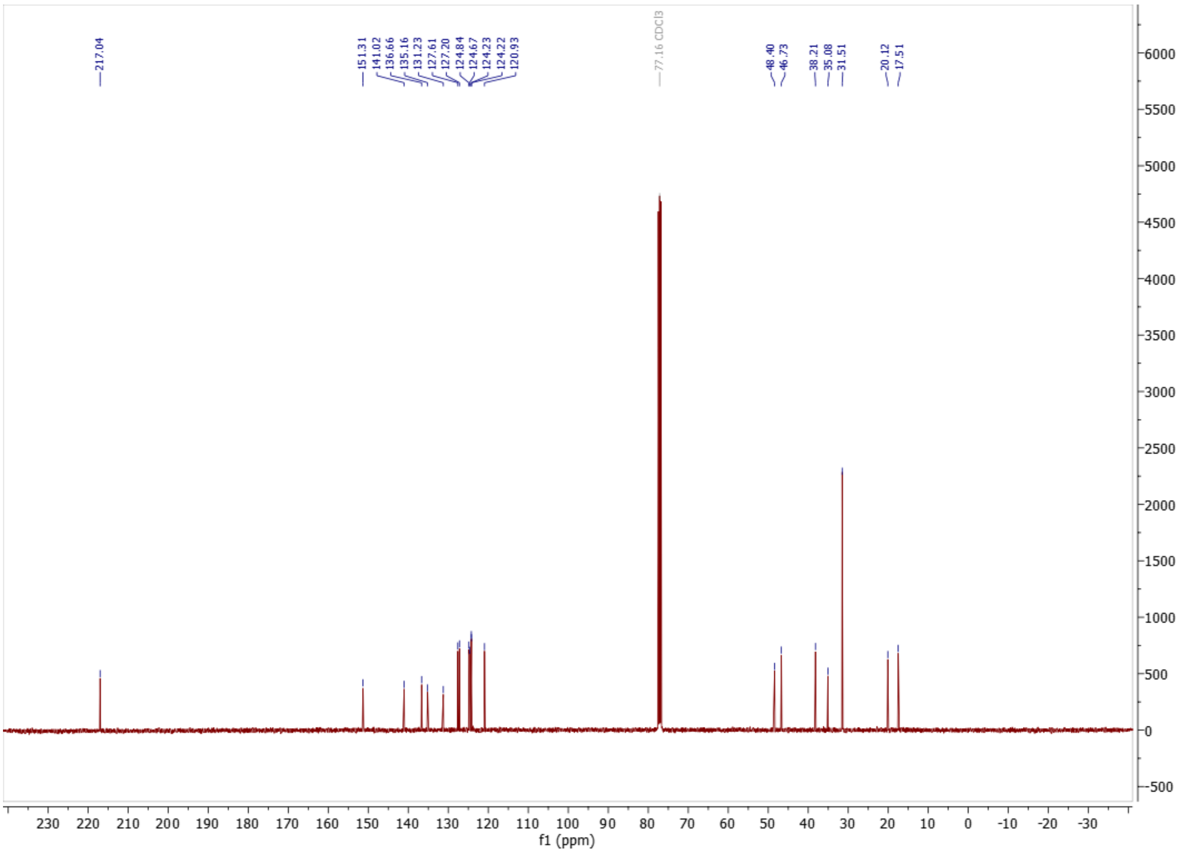

(3a*R*,11b*S*)-10-chloro-11b-methyl-2,3,3a,11b-tetrahydro-1*H*-cyclopenta[*l*]phenanthren-1-one (11f)

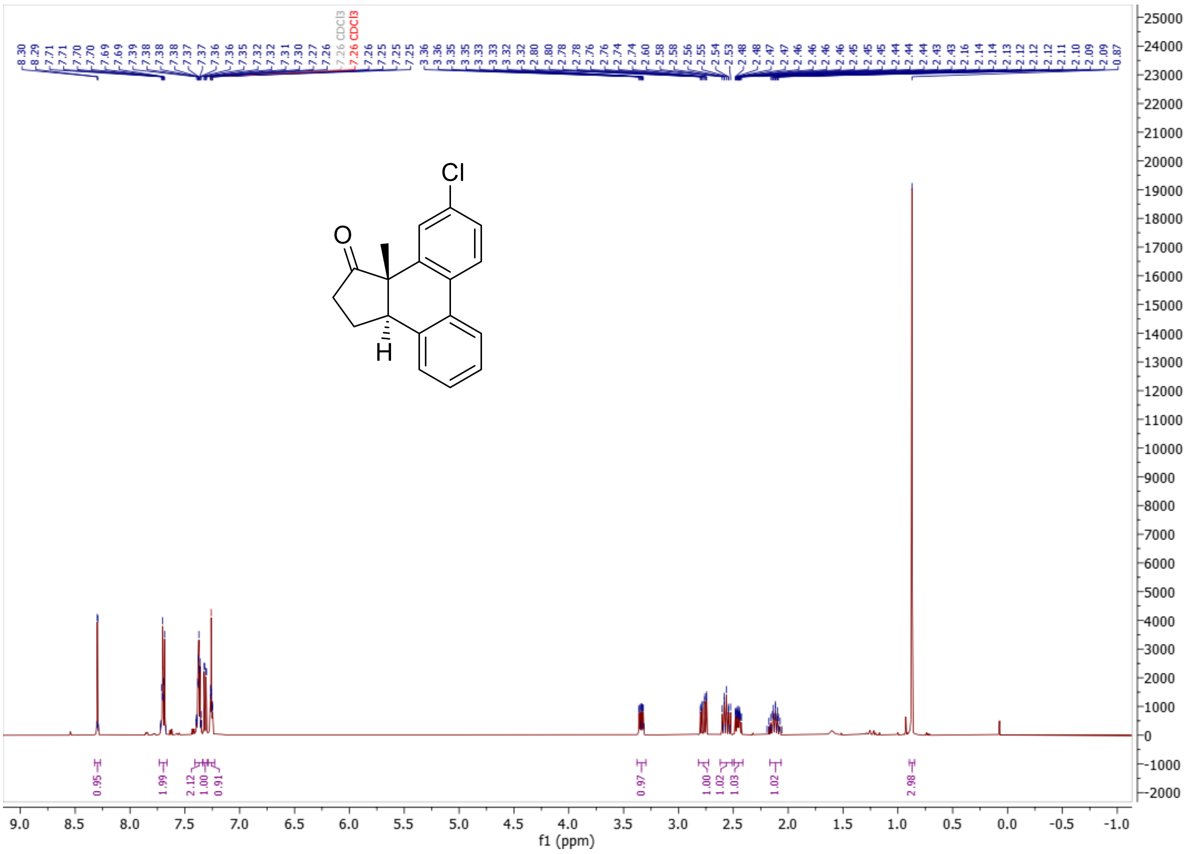

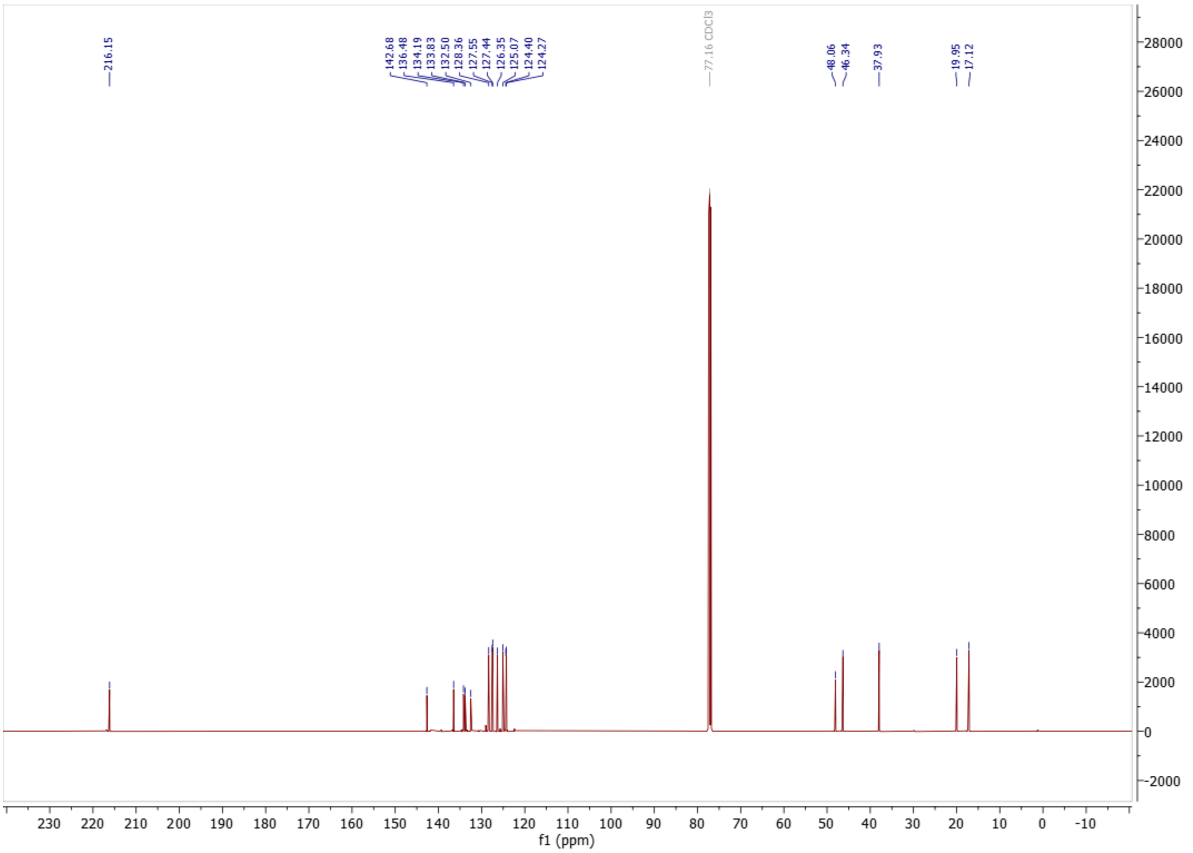

(3a*R*,11b*S*)-9,11,11b-trimethyl-2,3,3a,11b-tetrahydro-1*H*-cyclopenta[*l*]phenanthren-1-one (11g)

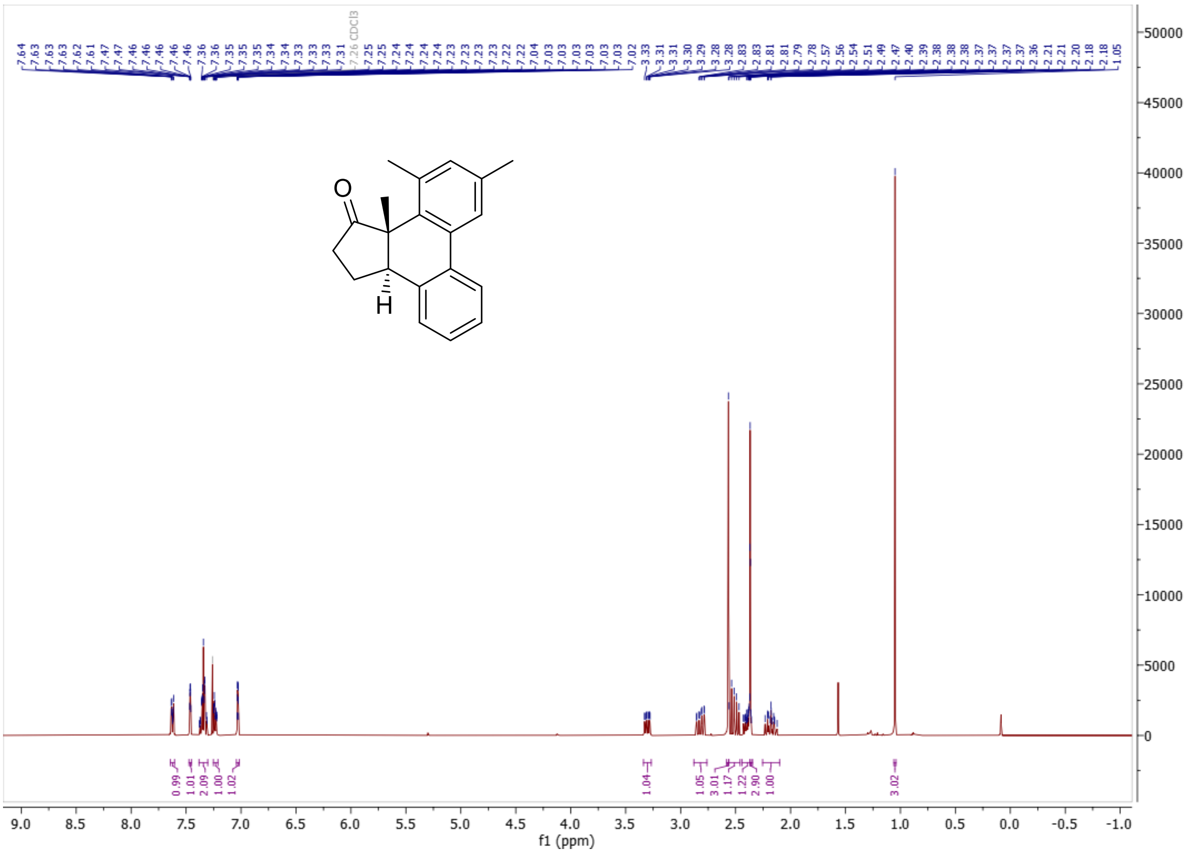

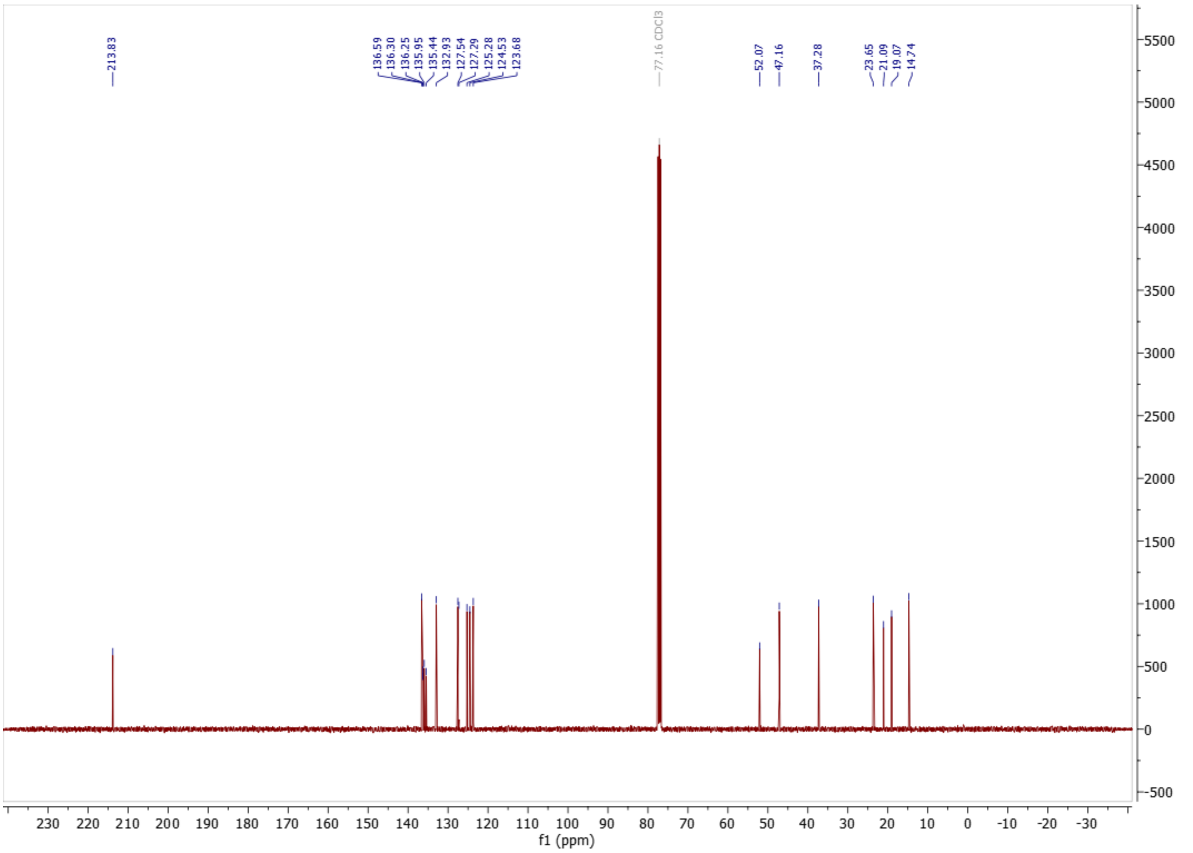

(3a*R*,11b*S*)-9,11-difluoro-11b-methyl-2,3,3a,11b-tetrahydro-1*H*-cyclopenta[*l*]phenanthren-1-one (11h)

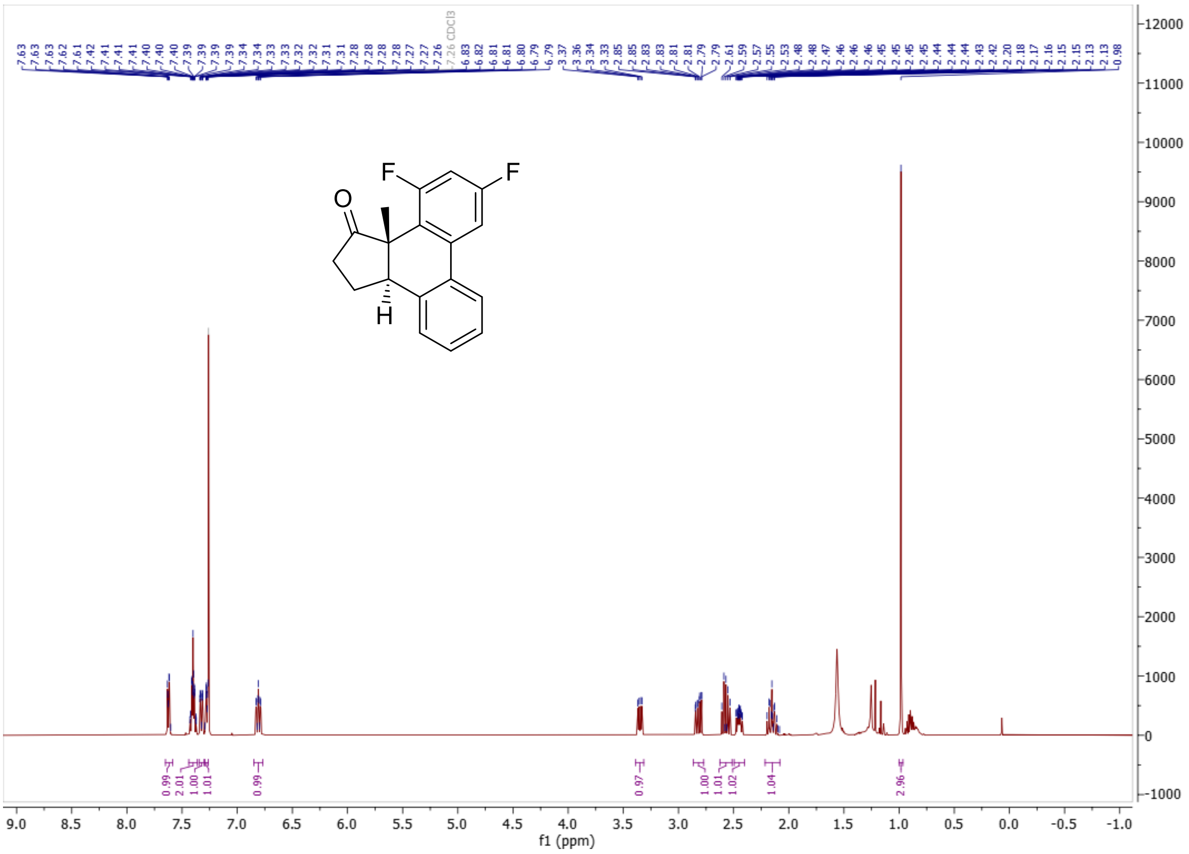

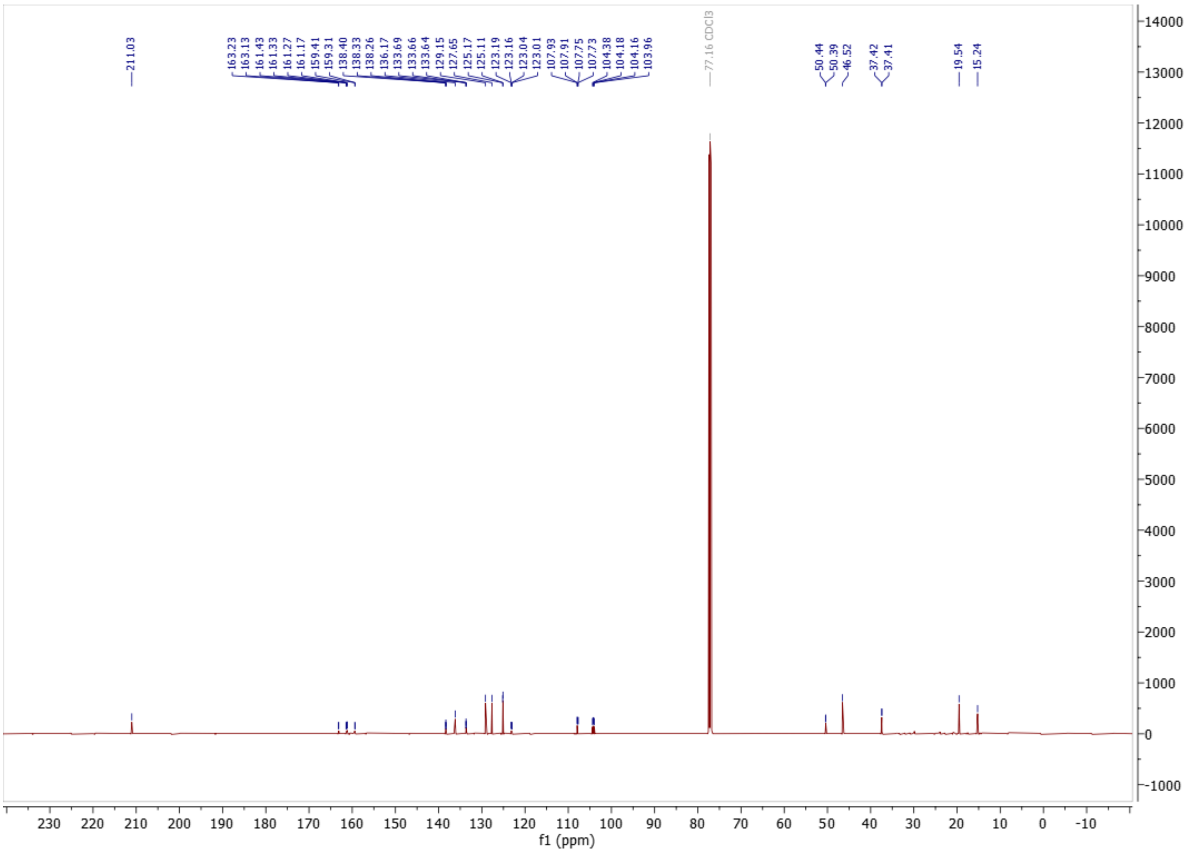

(3a*R*,11b*S*)-9,11-dichloro-11b-methyl-2,3,3a,11b-tetrahydro-1*H*-cyclopenta[*l*]phenanthren-1-one (11i)

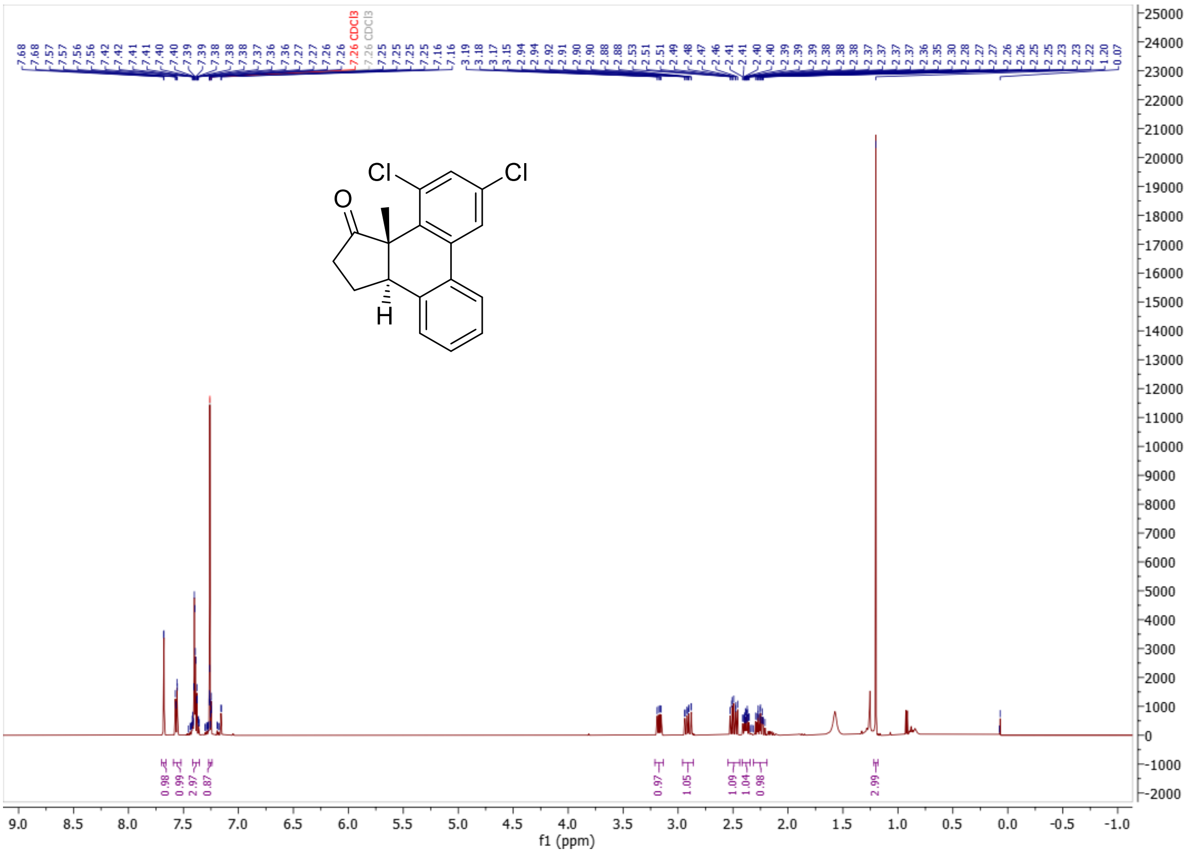

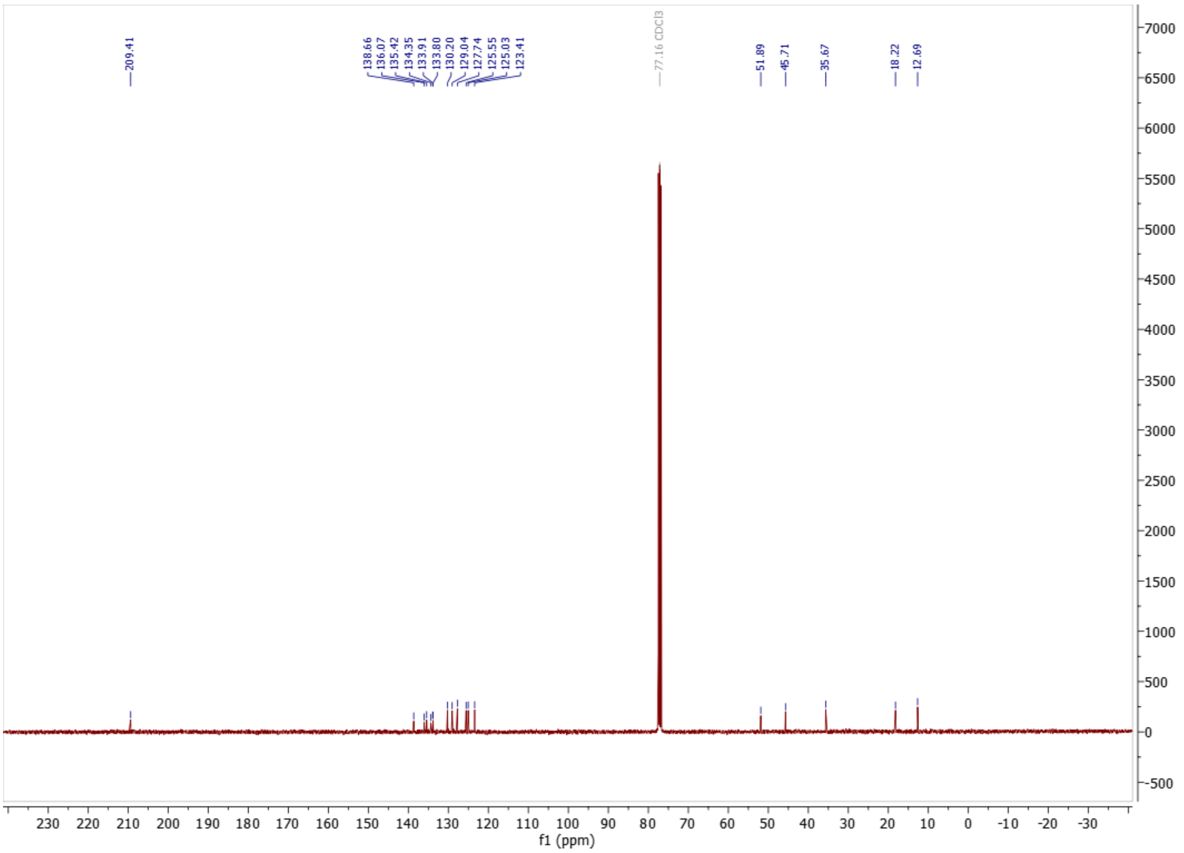

(3a*R*,11b*S*)-9,11-dimethoxy-11b-methyl-2,3,3a,11b-tetrahydro-1*H*-cyclopenta[*l*]phenanthren-1-one (11j)

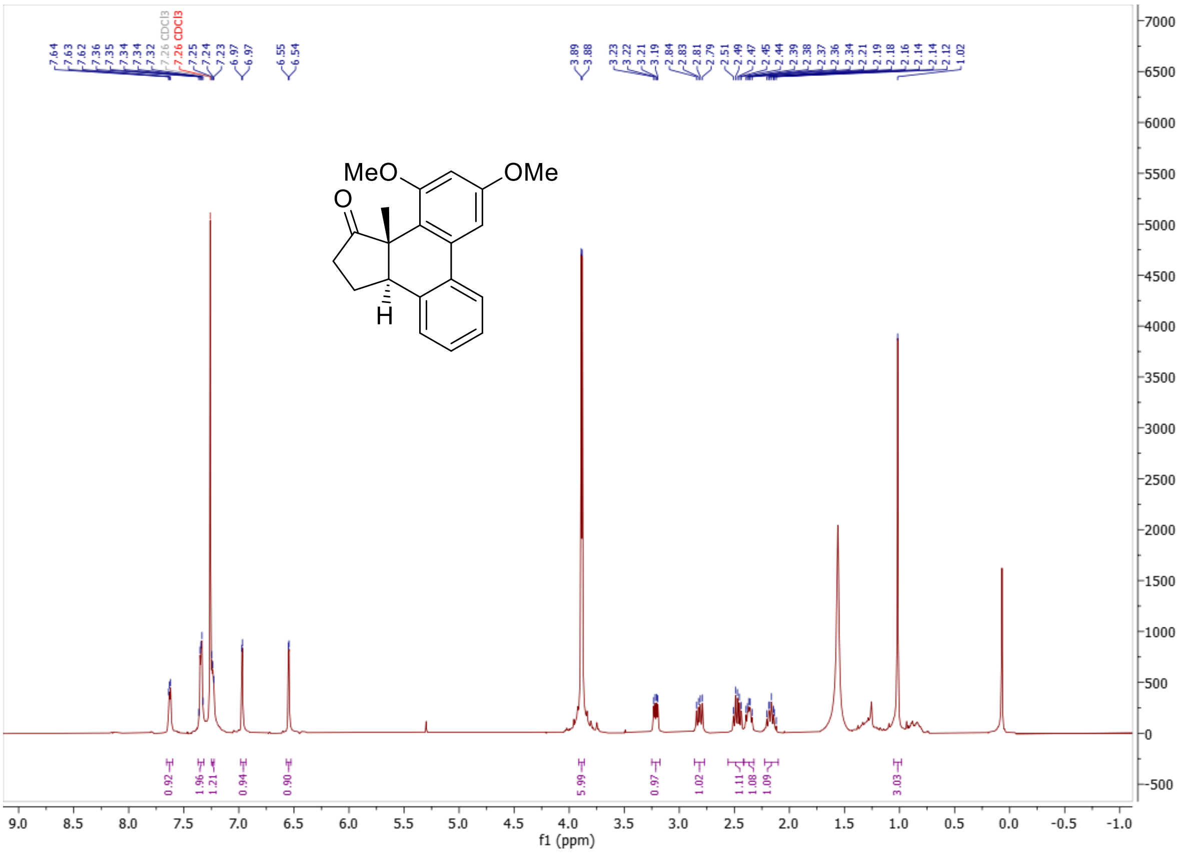

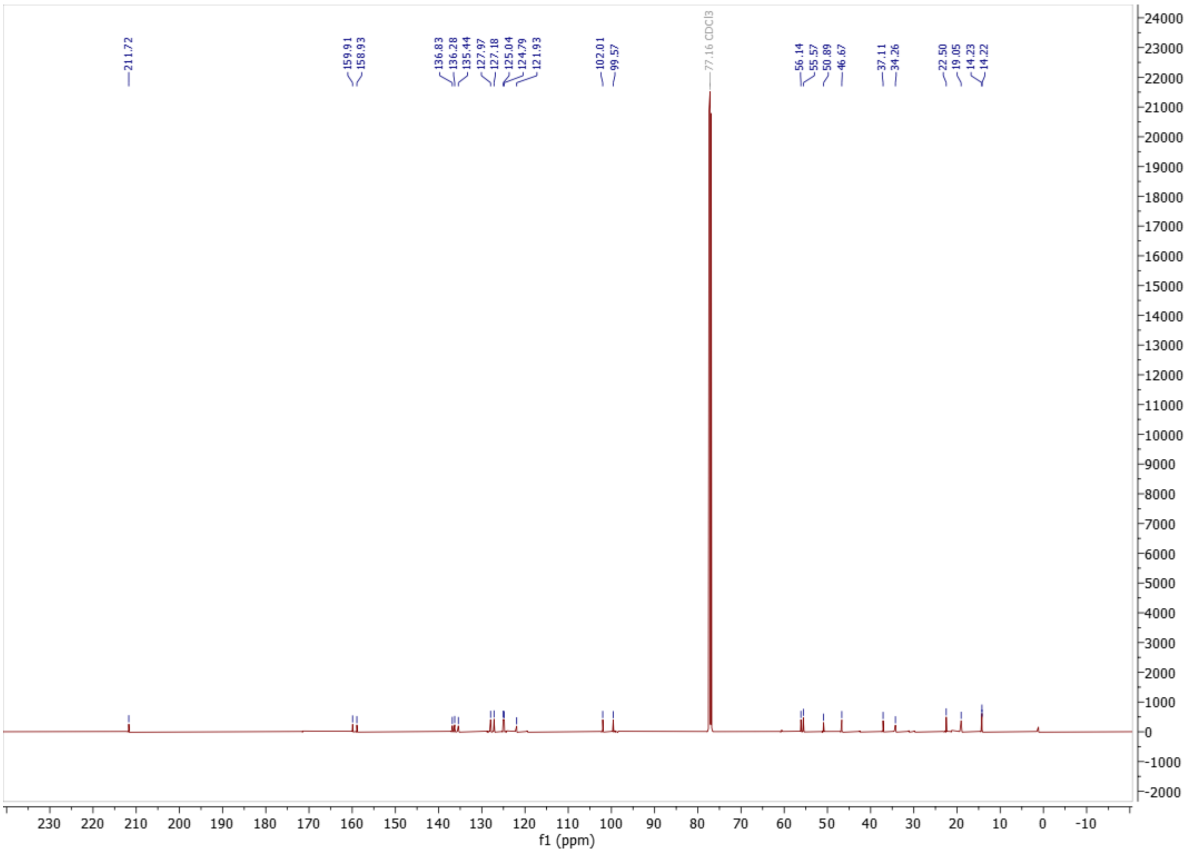

(3a*S*,11b*S*)-9,11-dimethoxy-11b-methyl-2,3,3a,11b-tetrahydro-1*H*-cyclopenta[*l*]phenanthren-1-one (11j')

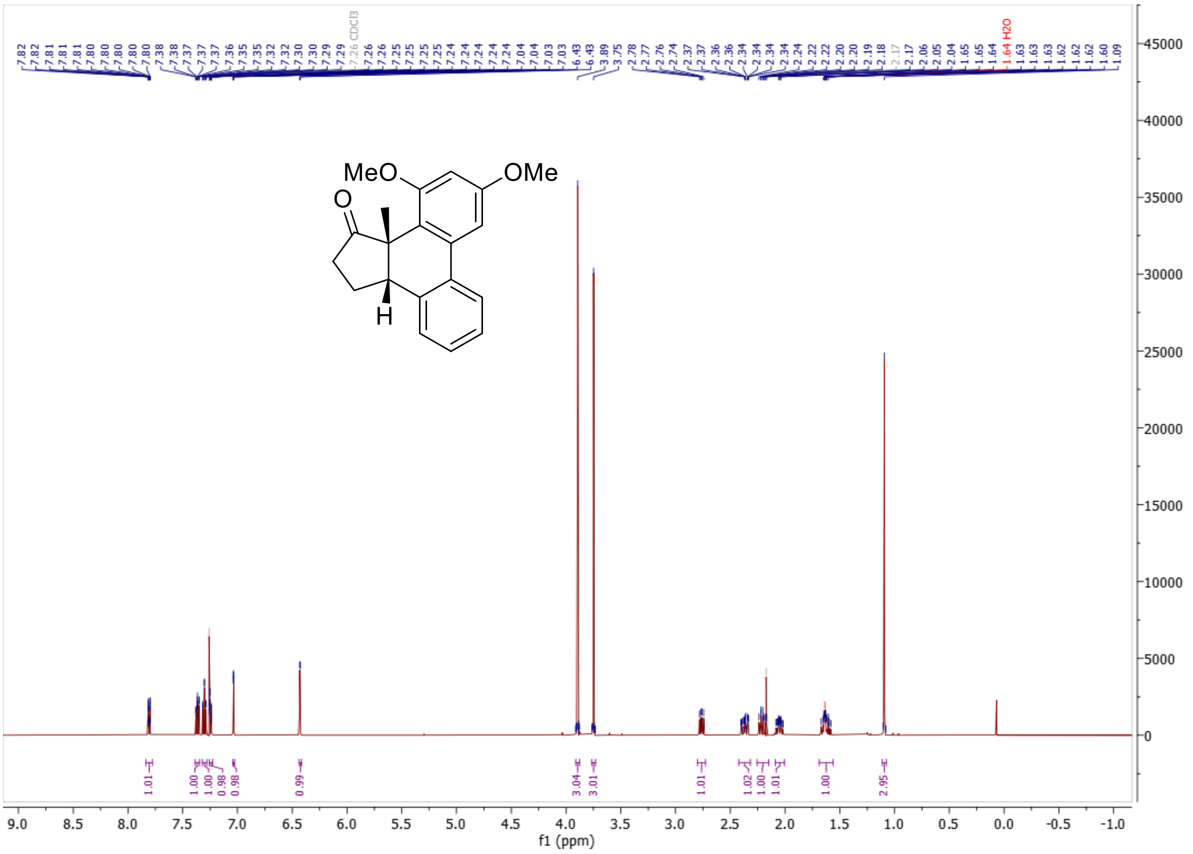

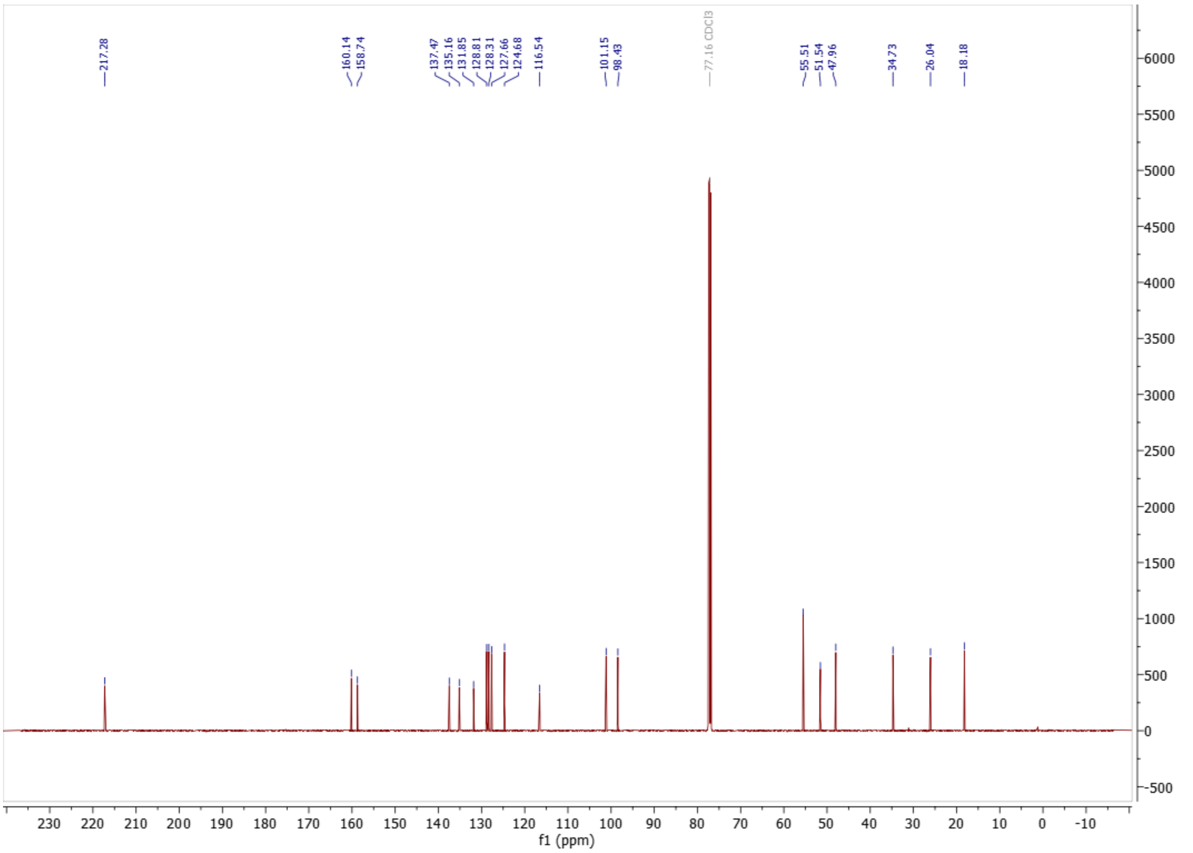

(3a*R*,11b*S*)-11b-methyl-9-(trifluoromethyl)-2,3,3a,11b-tetrahydro-1*H*-cyclopenta[*l*]phenanthren-1-one (11k)

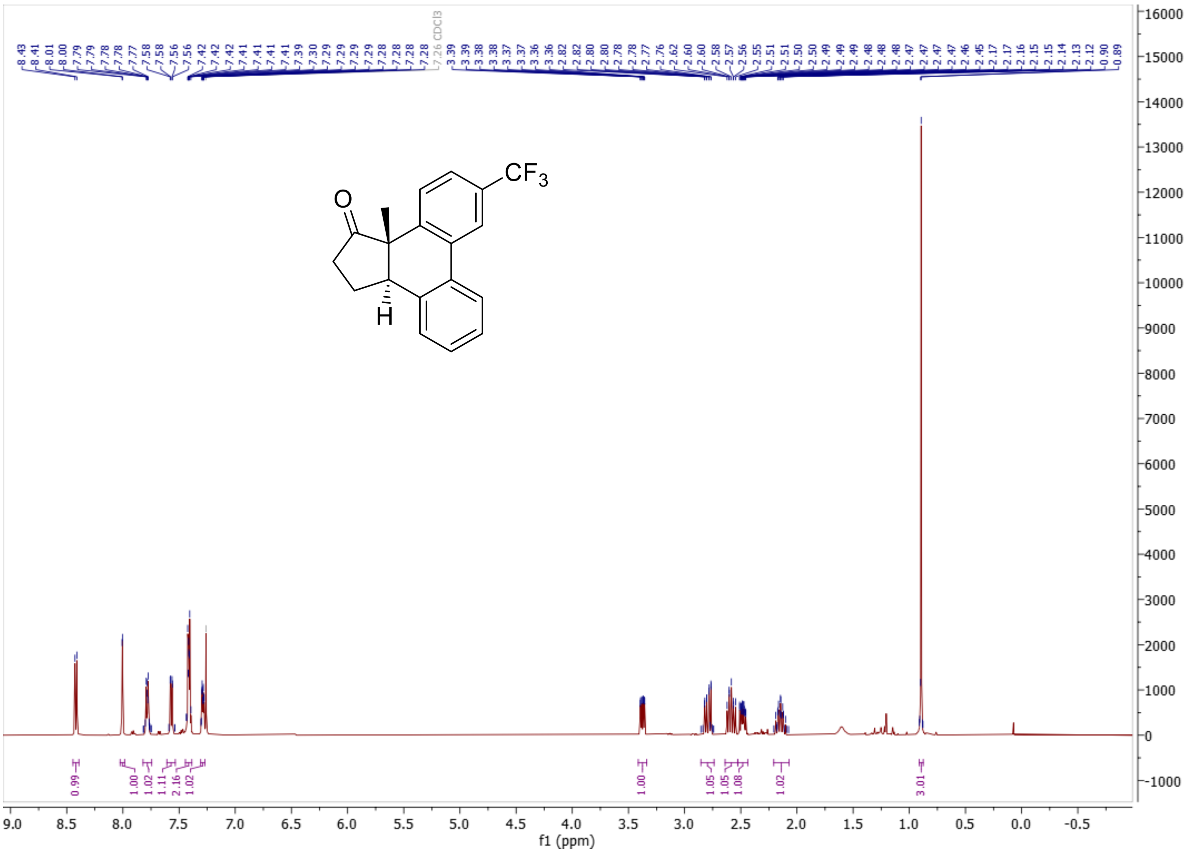

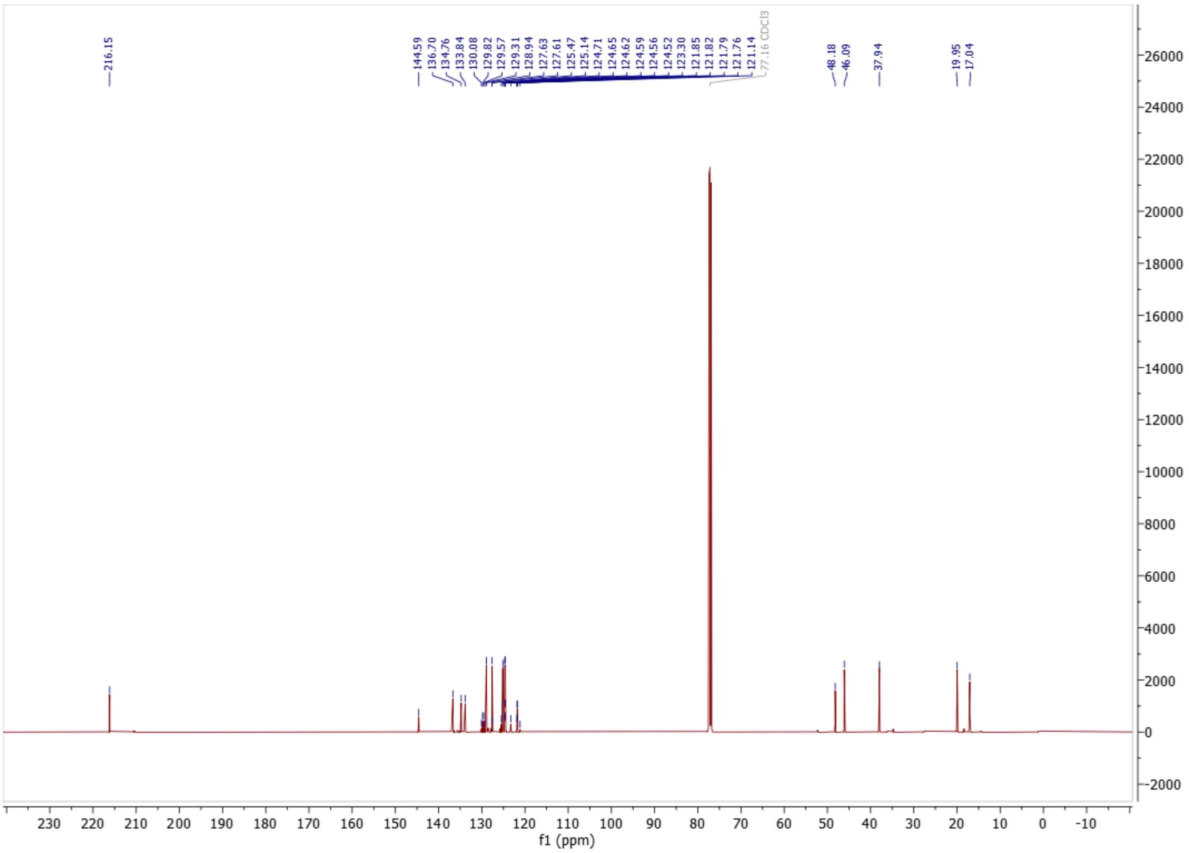

(3a*R*,11b*S*)-11b-methyl-9-phenyl-2,3,3a,11b-tetrahydro-1*H*-cyclopenta[*l*]phenanthren-1-one (11l)

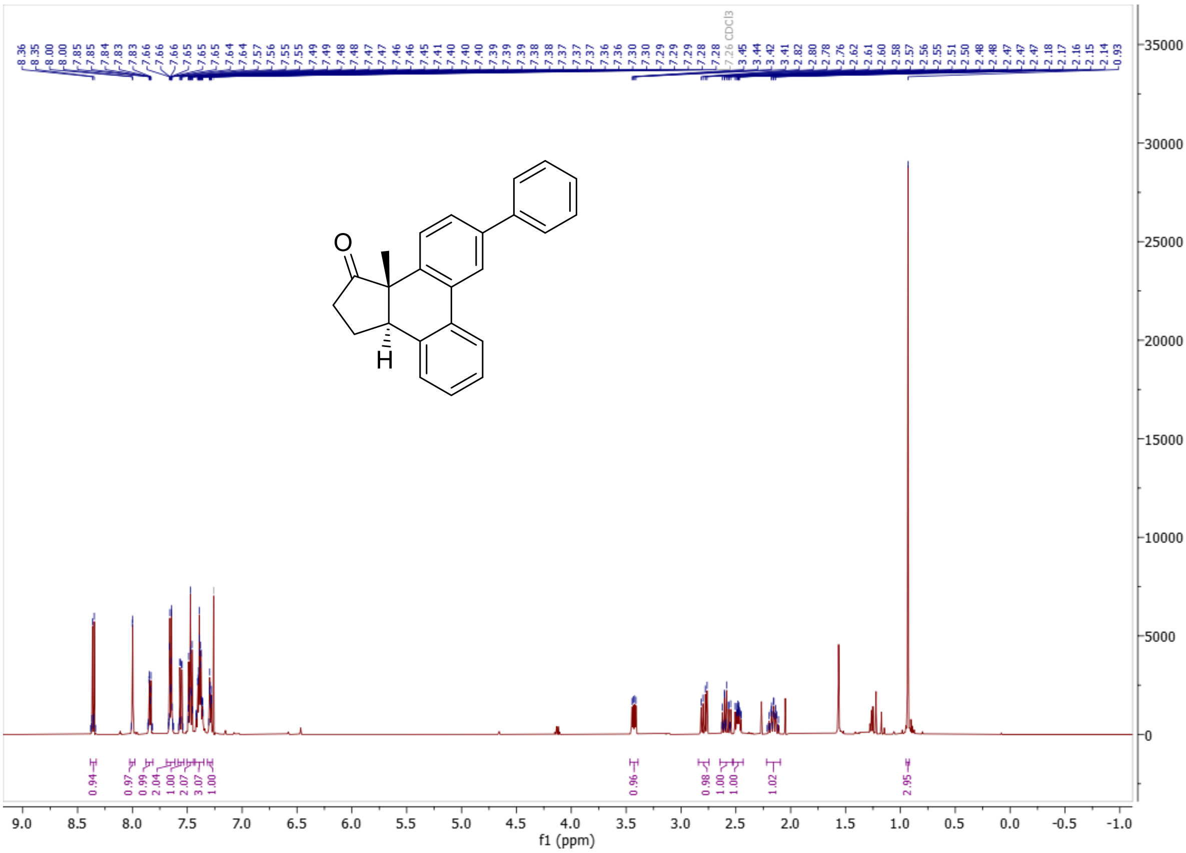

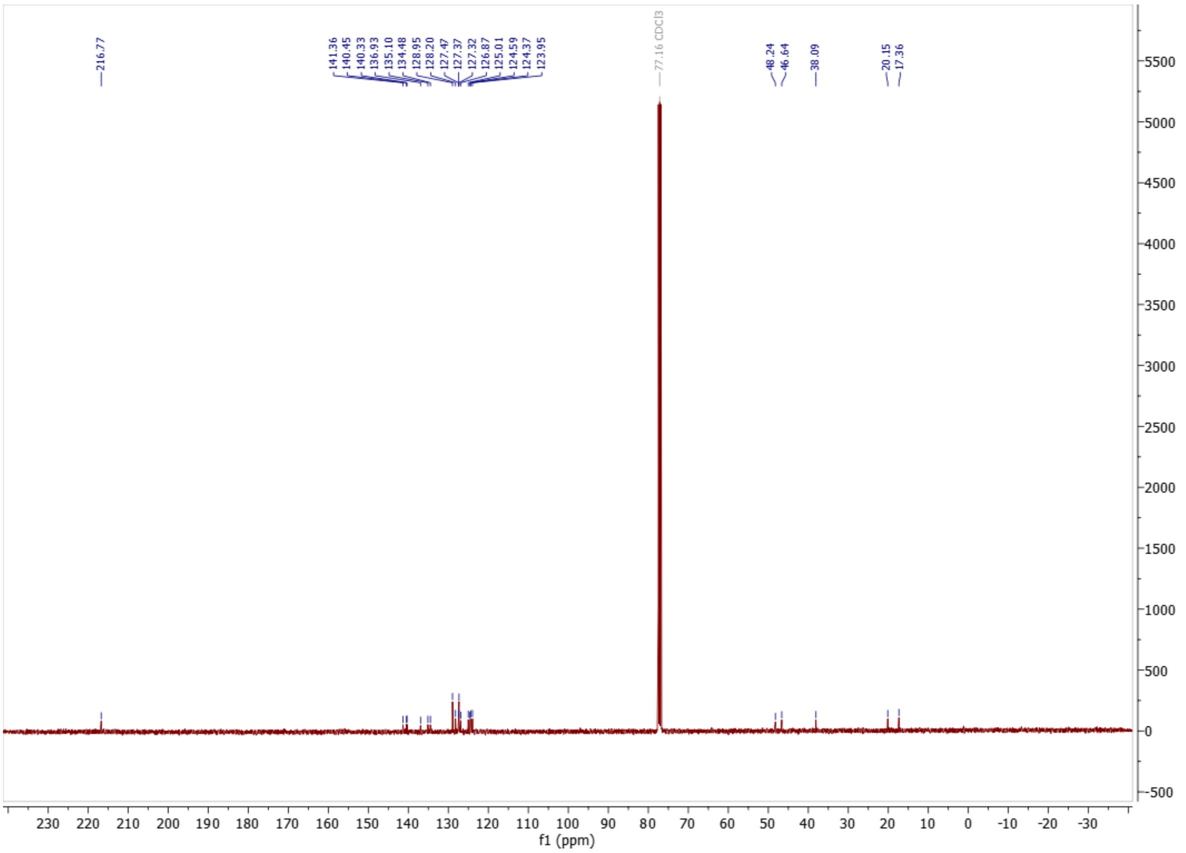

(3a*R*,11b*S*)-9,11b-dimethyl-2,3,3a,11b-tetrahydro-1*H*-cyclopenta[*l*]phenanthren-1-one (11m) and (3a*R*,11b*S*)-11,11b-dimethyl-2,3,3a,11b-tetrahydro-1*H*-cyclopenta[*l*]phenanthren-1-one (11m')

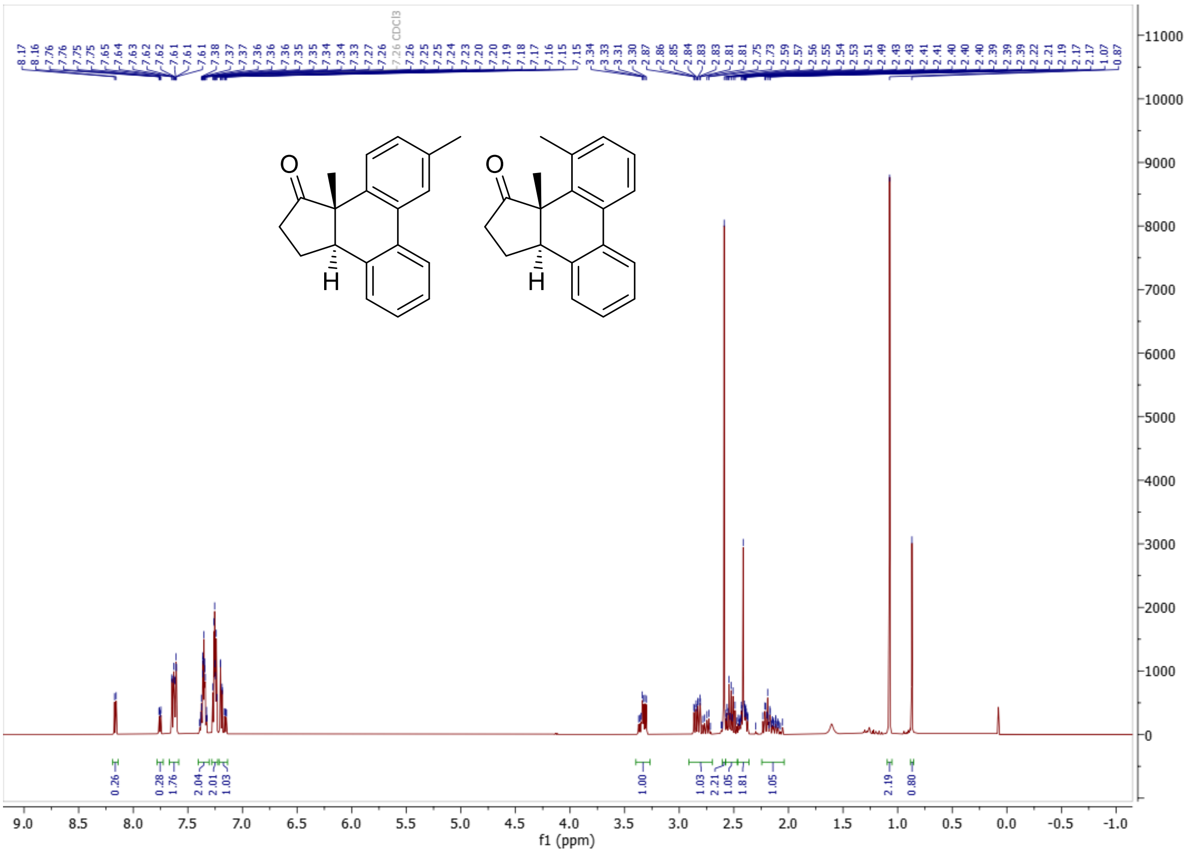

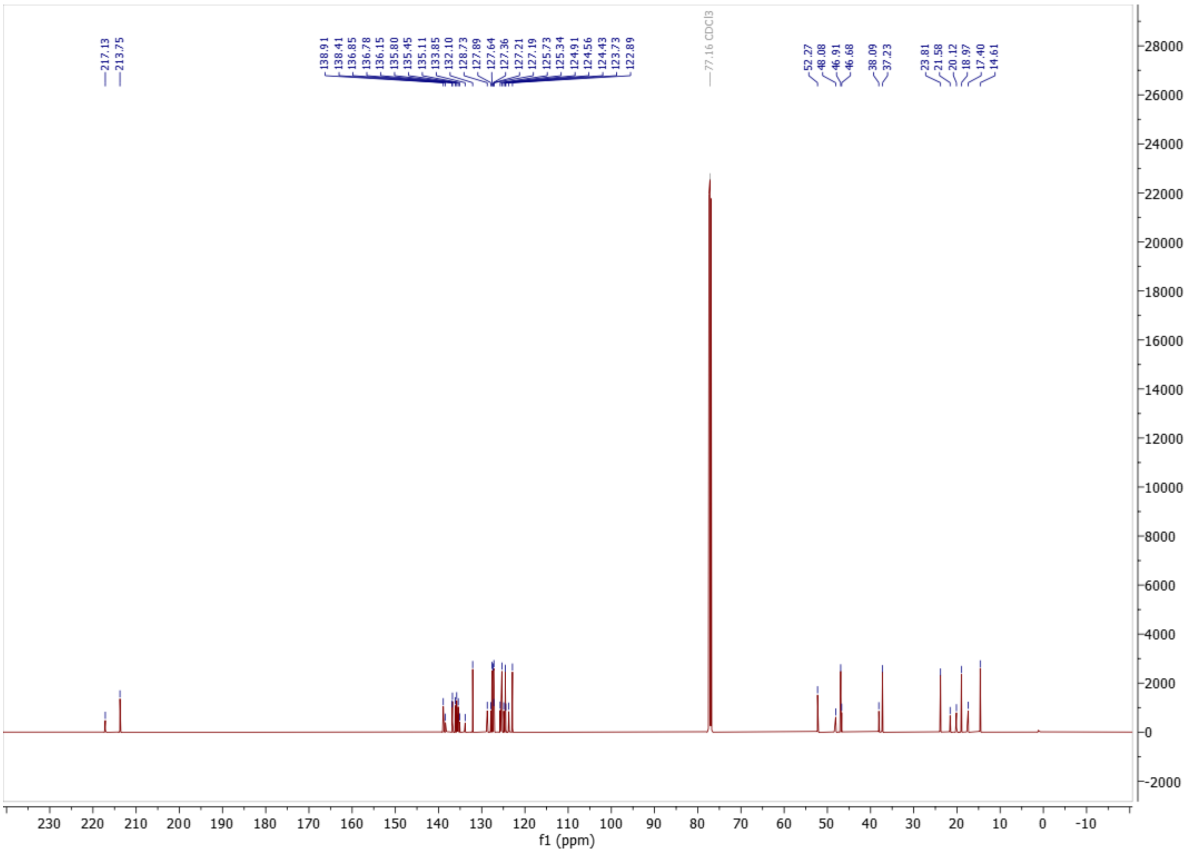

(1*S*,3a*R*,11b*S*)-9,11b-dimethyl-2,3,3a,11b-tetrahydro-1*H*-cyclopenta[*l*]phenanthren-1-ol ((*S*)-12m')

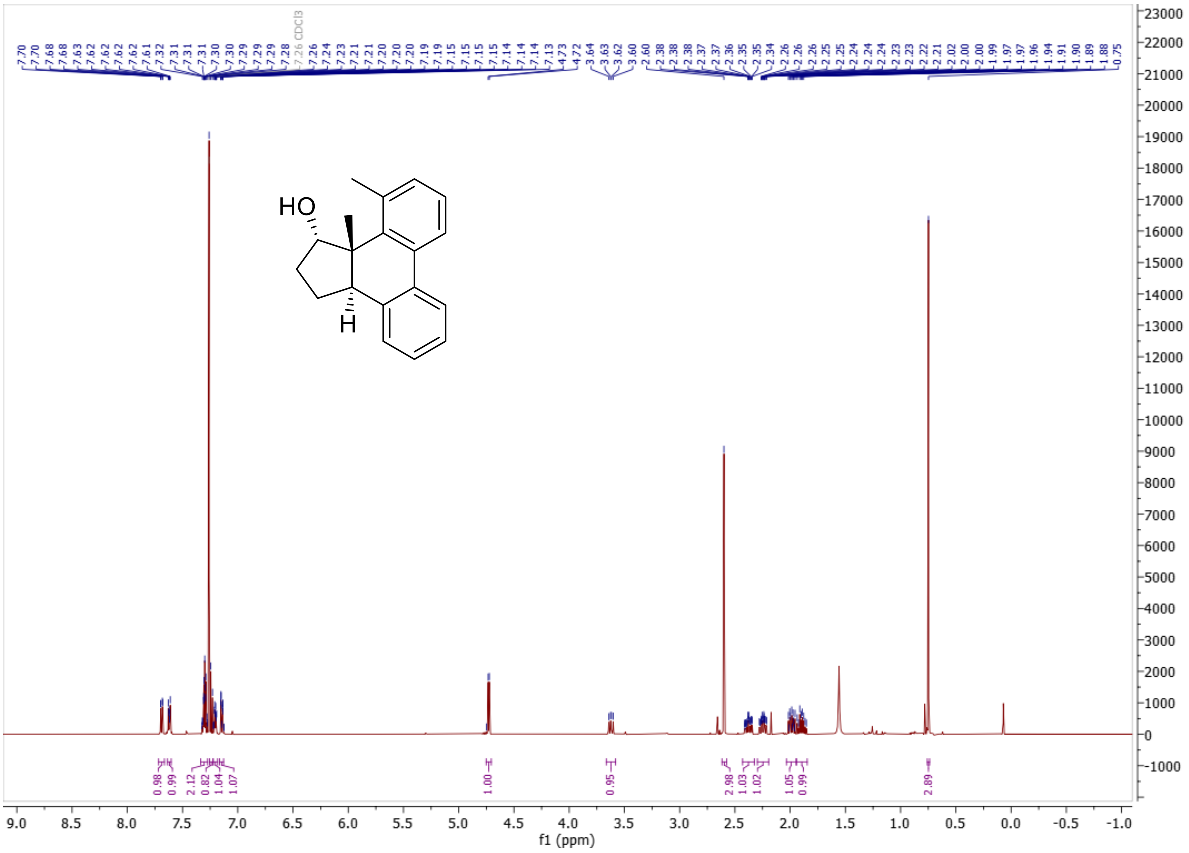

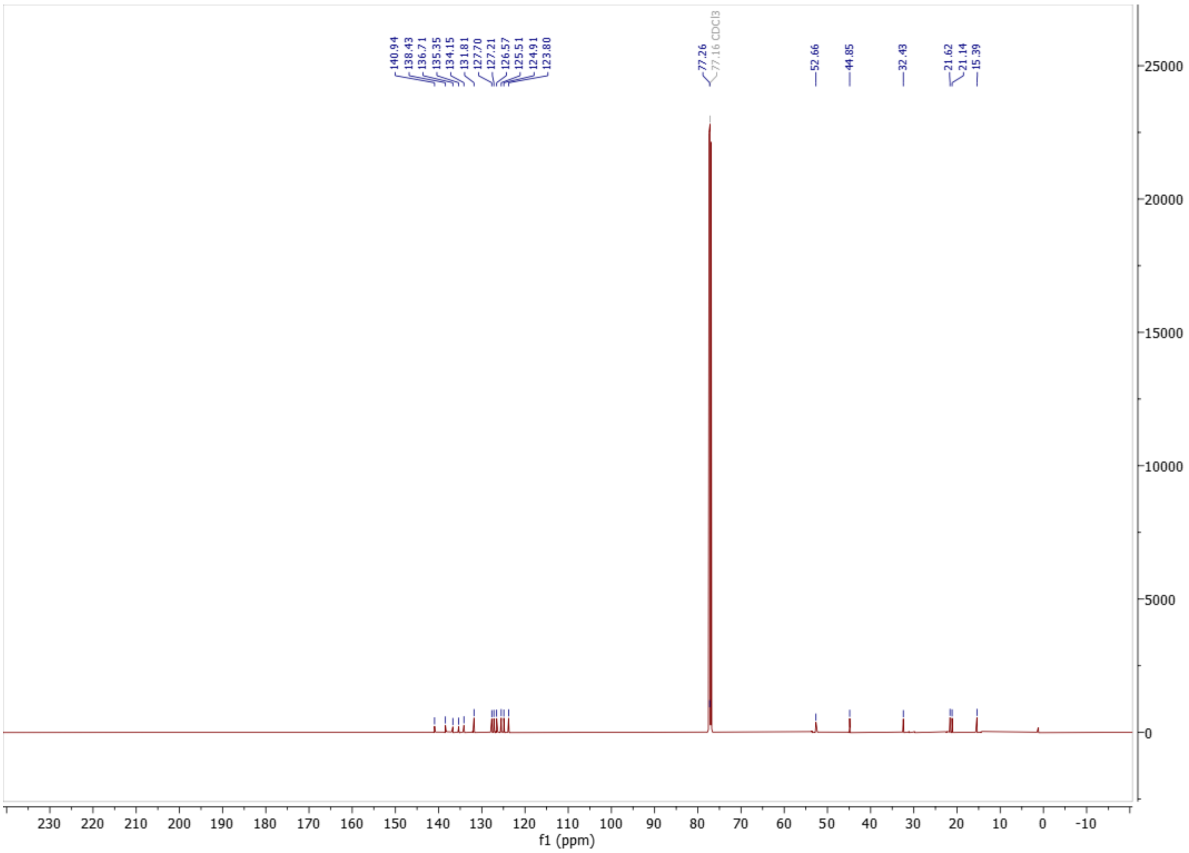

(1*R*,3a*R*,11b*S*)-9,11b-dimethyl-2,3,3a,11b-tetrahydro-1*H*-cyclopenta[*l*]phenanthren-1-ol ((*R*)-12m')

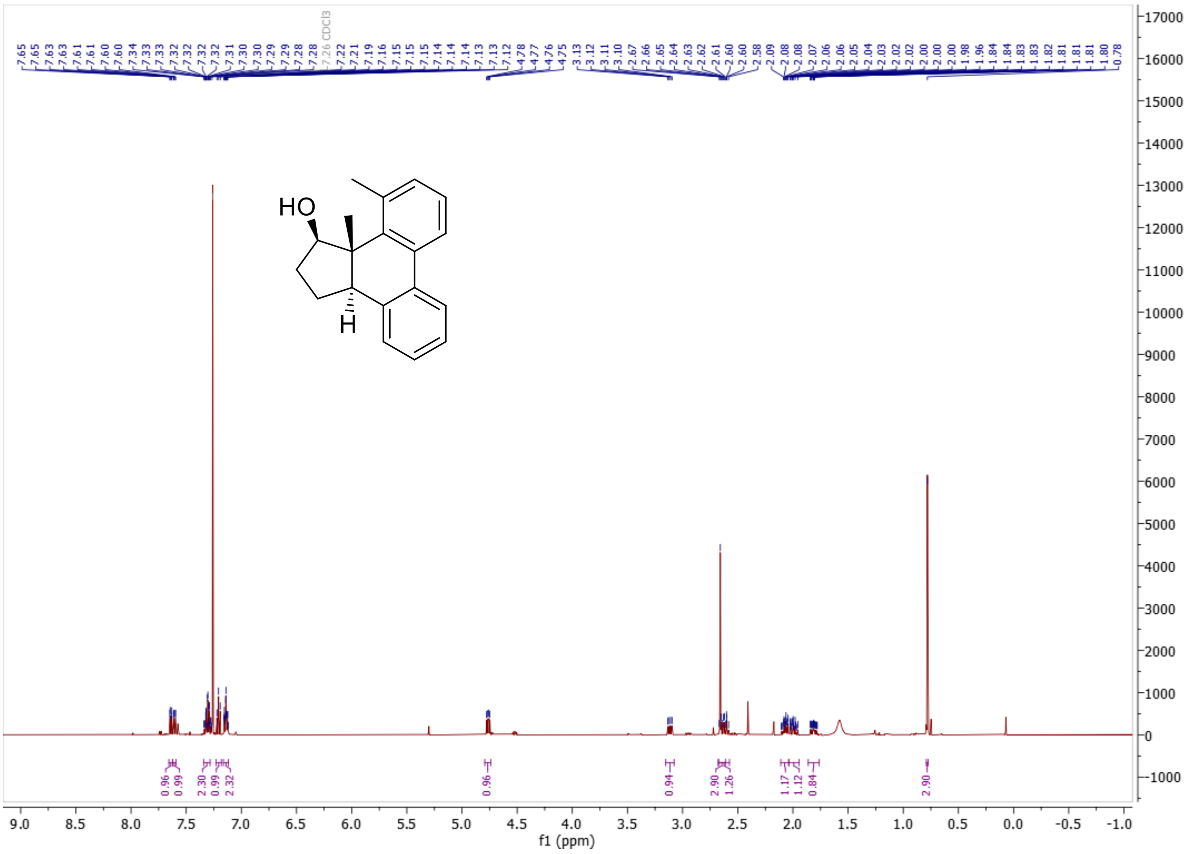

(1*R*,3a*R*,11b*S*)-11,11b-dimethyl-2,3,3a,11b-tetrahydro-1*H*-cyclopenta[*l*]phenanthren-1-ol ((*S*)-12m)

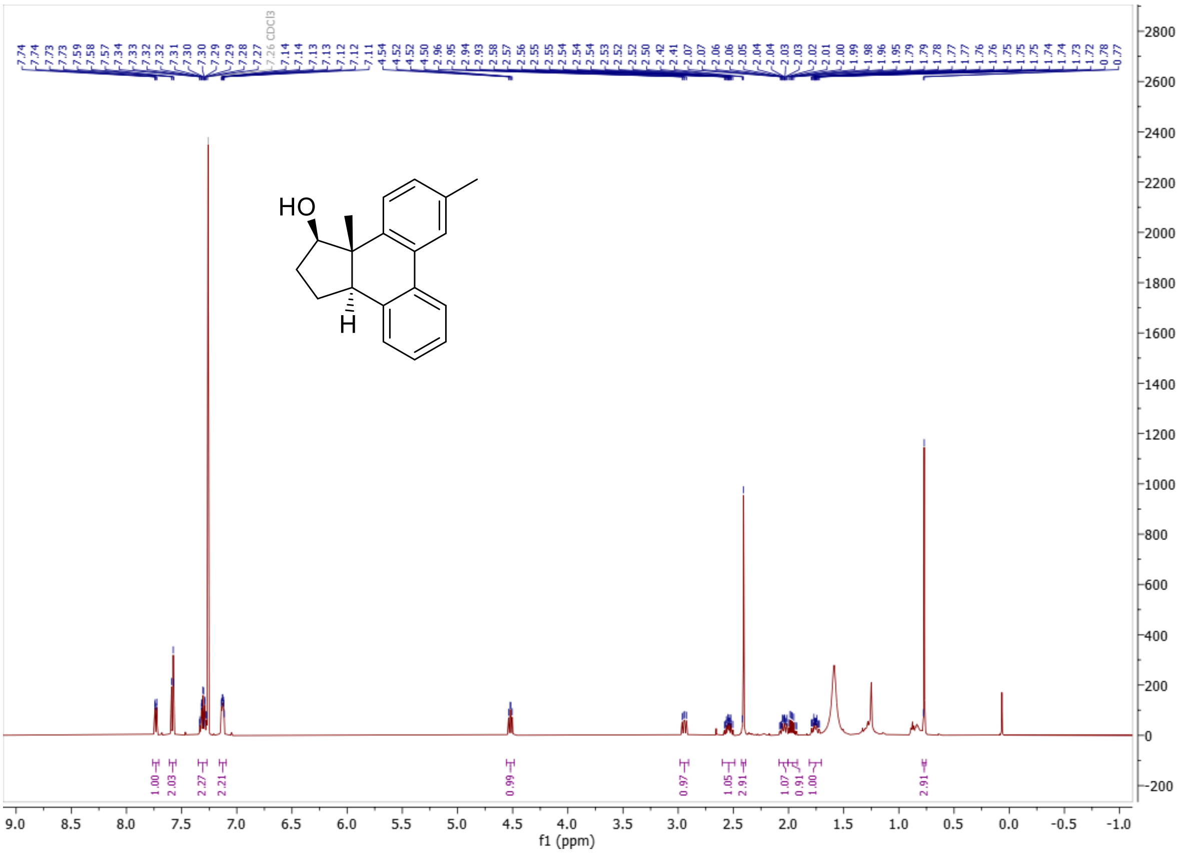

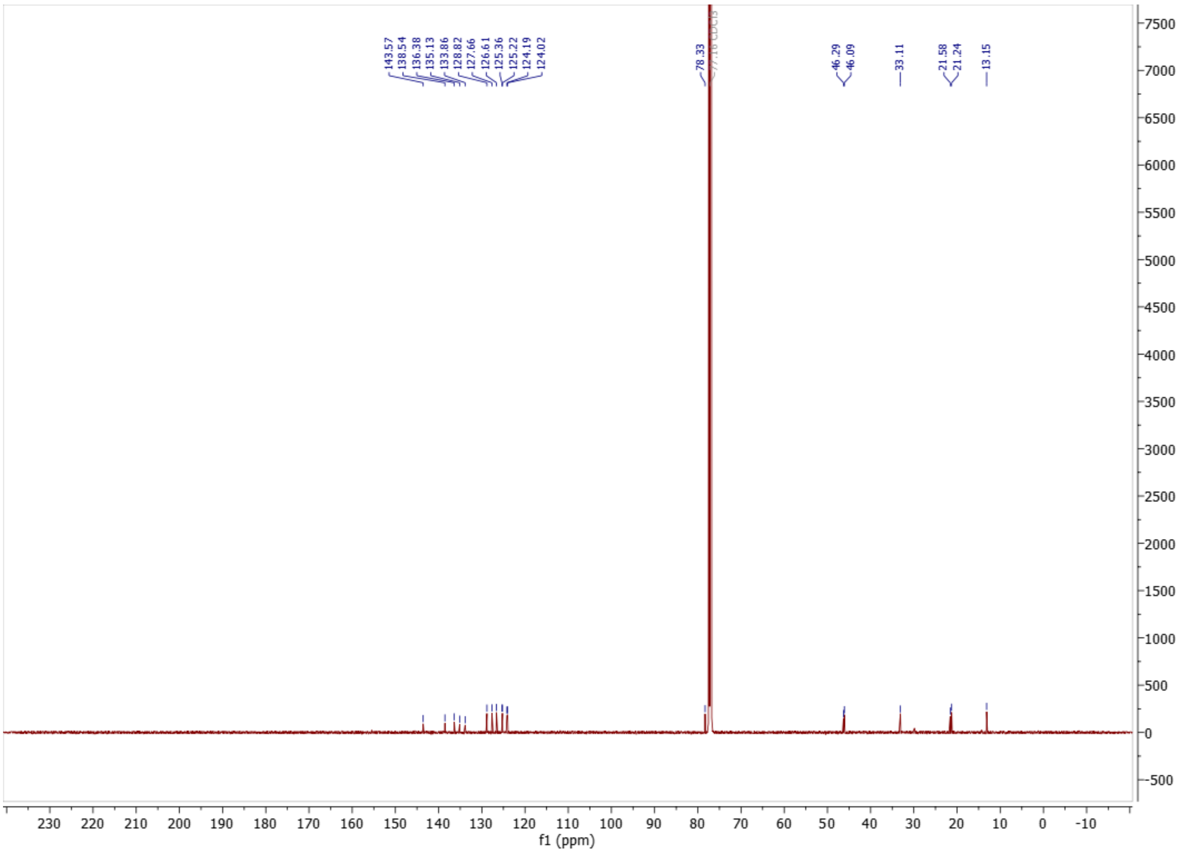

(3a*R*,11b*S*)-9-fluoro-11b-methyl-2,3,3a,11b-tetrahydro-1*H*-cyclopenta[*l*]phenanthren-1-one (11n)

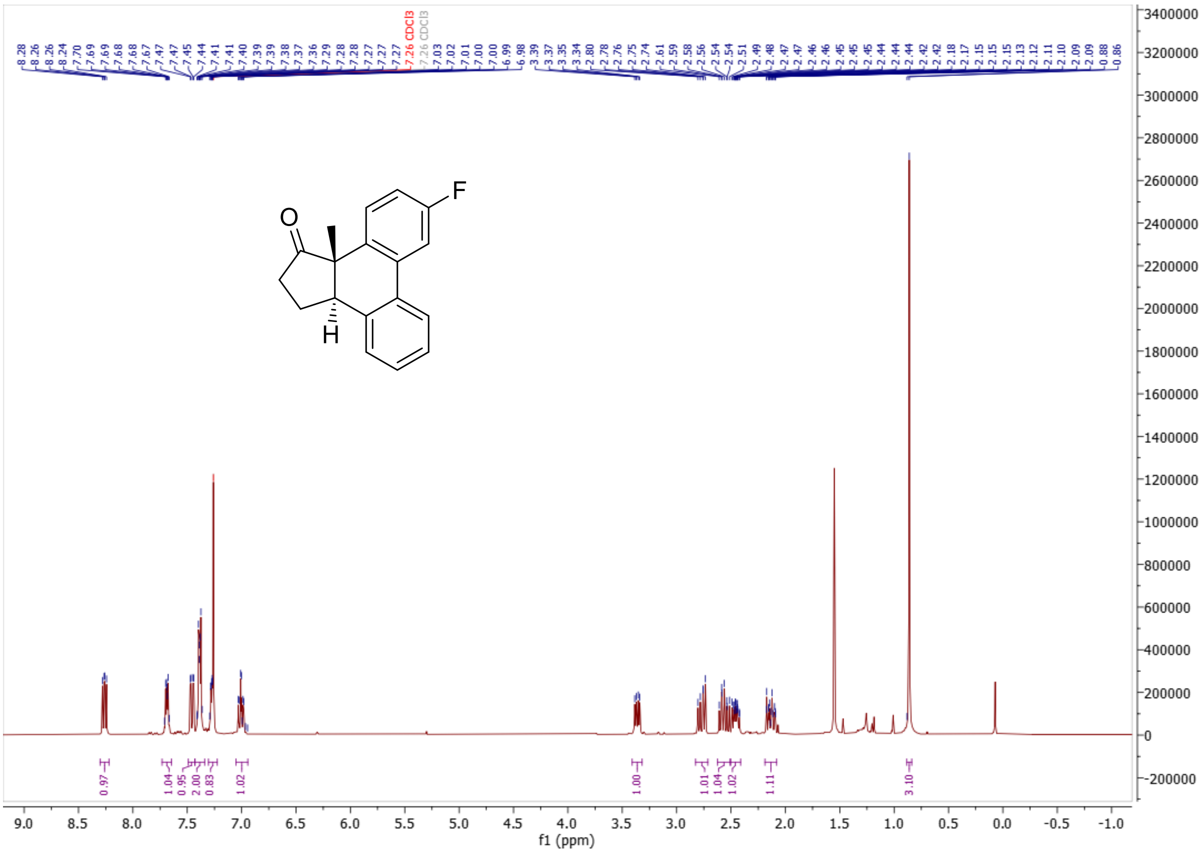

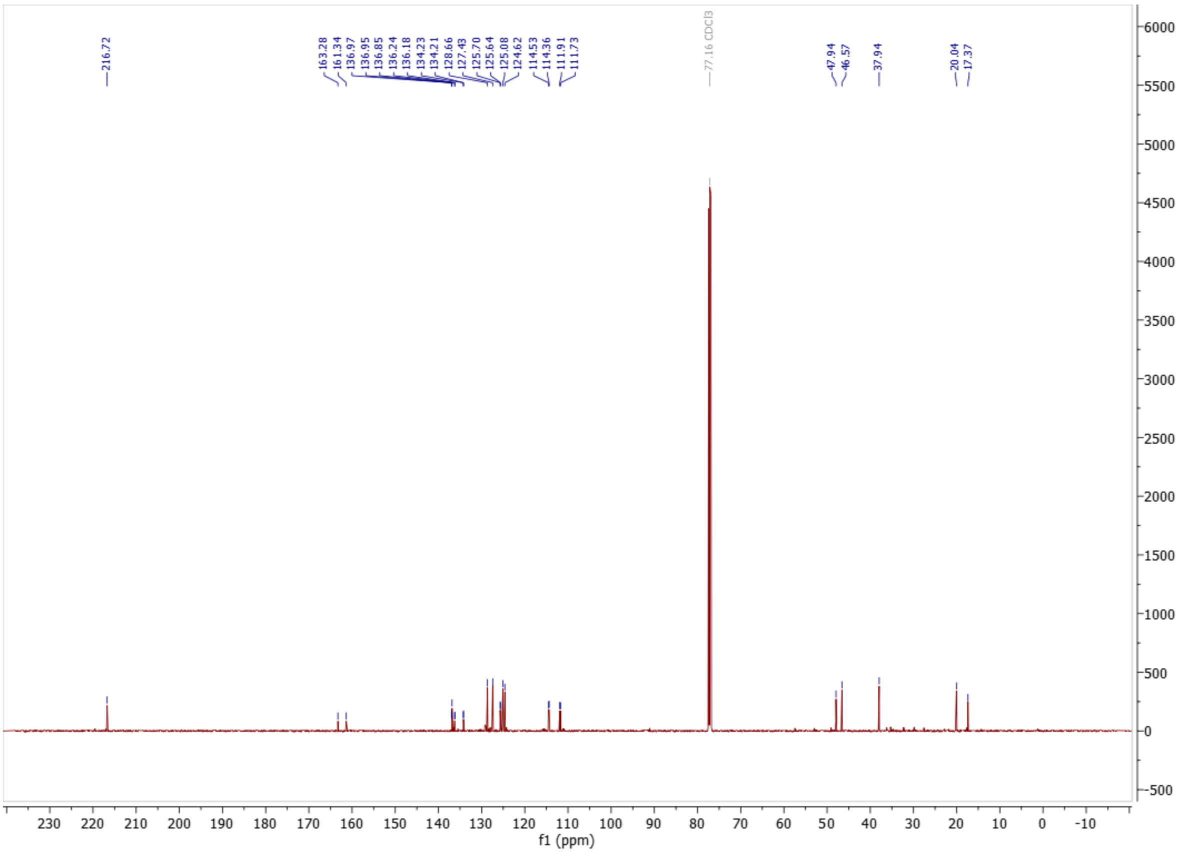

(3aR,11bS)-11-fluoro-11b-methyl-2,3,3a,11b-tetrahydro-1H-cyclopenta[l]phenanthren-1-one (11n')

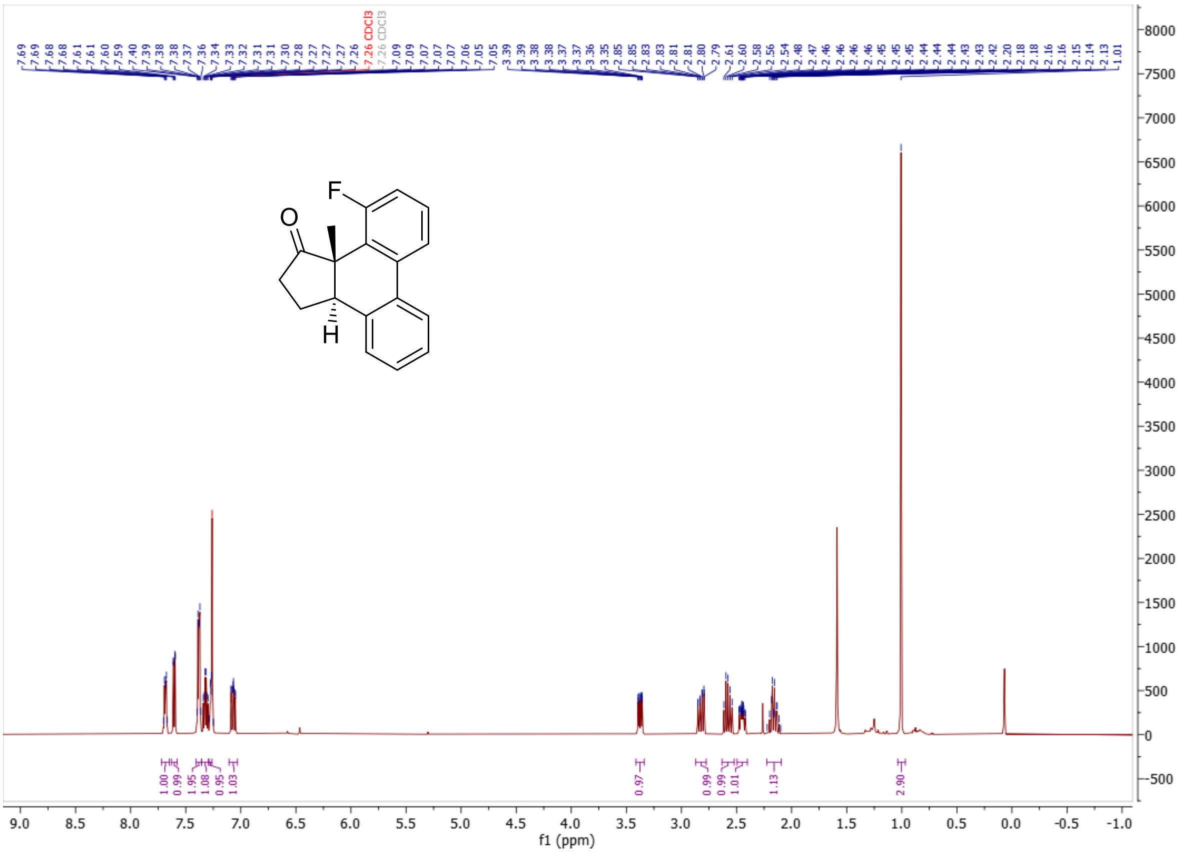

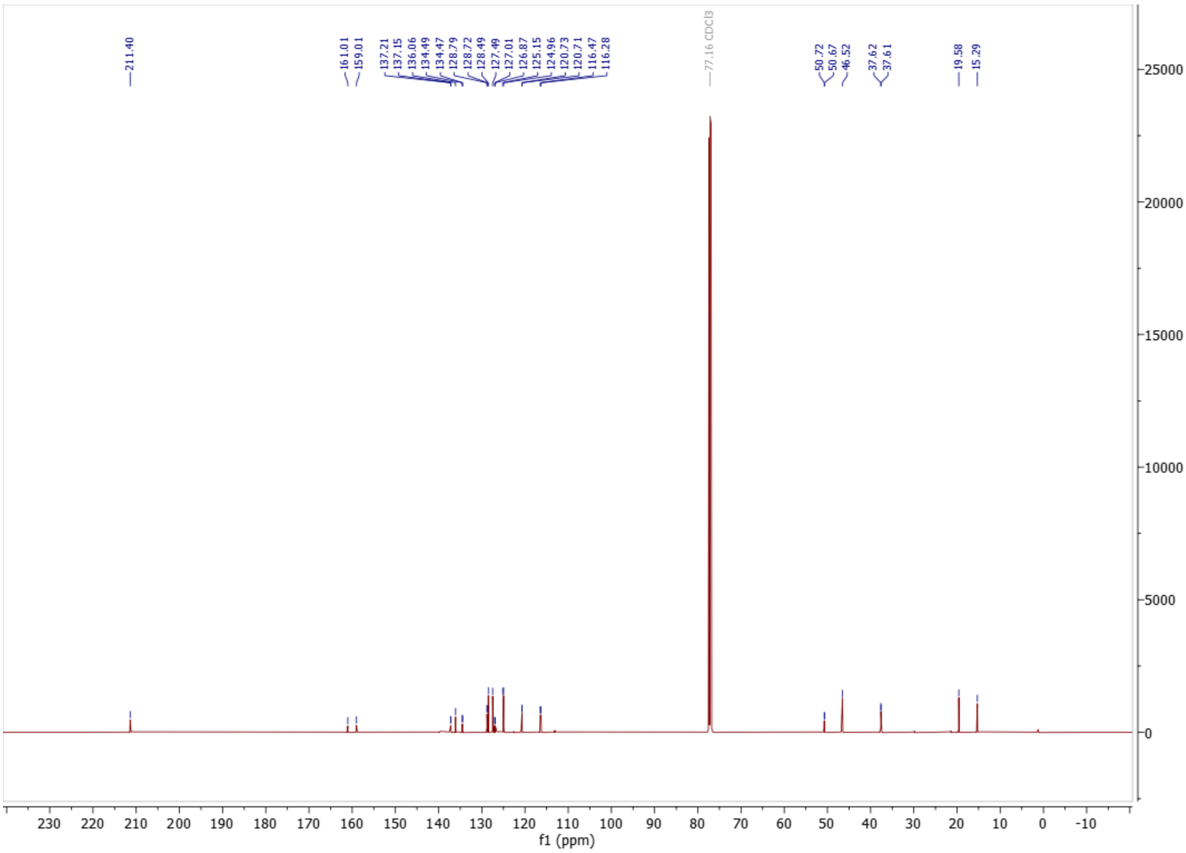

(3a*R*,11b*S*)-9-chloro-11b-methyl-2,3,3a,11b-tetrahydro-1*H*-cyclopenta[*l*]phenanthren-1-one (11o) and (3a*R*,11b*S*)-11-chloro-11b-methyl-2,3,3a,11b-tetrahydro-1*H*-cyclopenta[*l*]phenanthren-1-one (11o')

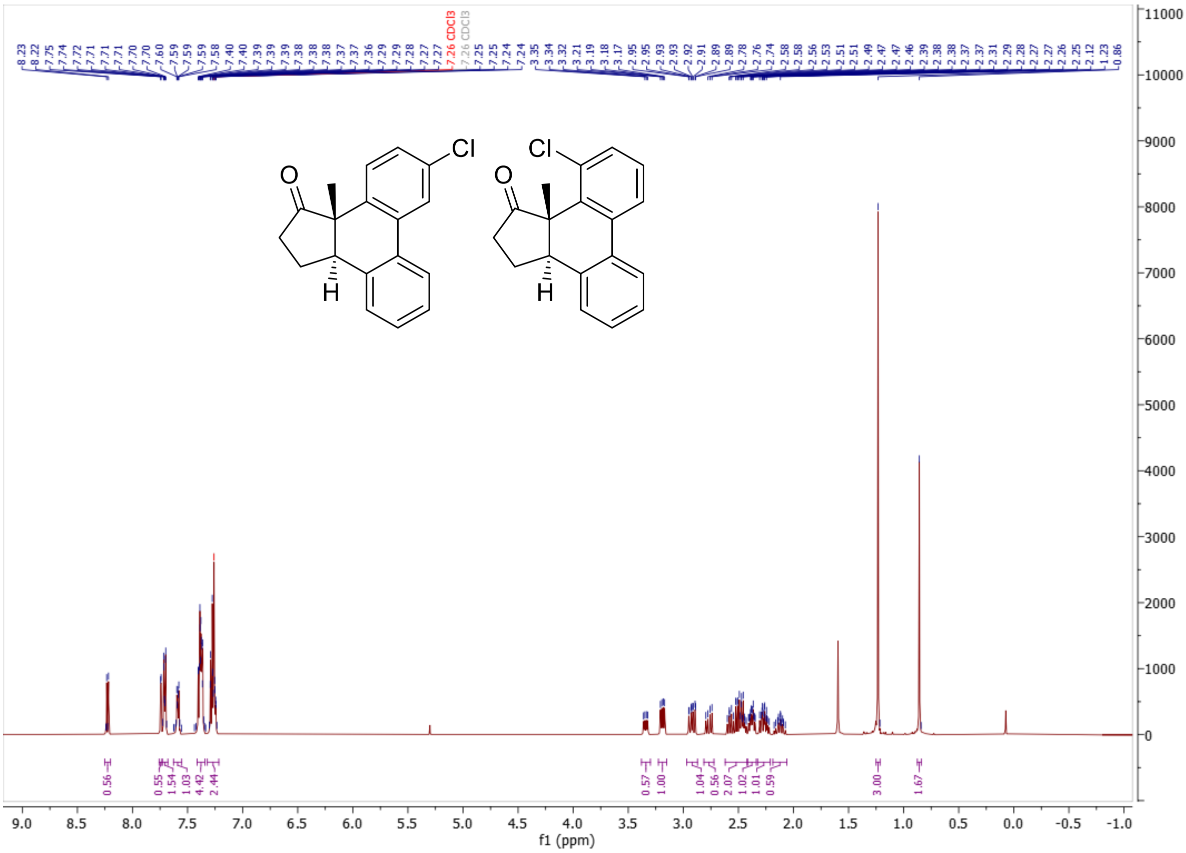

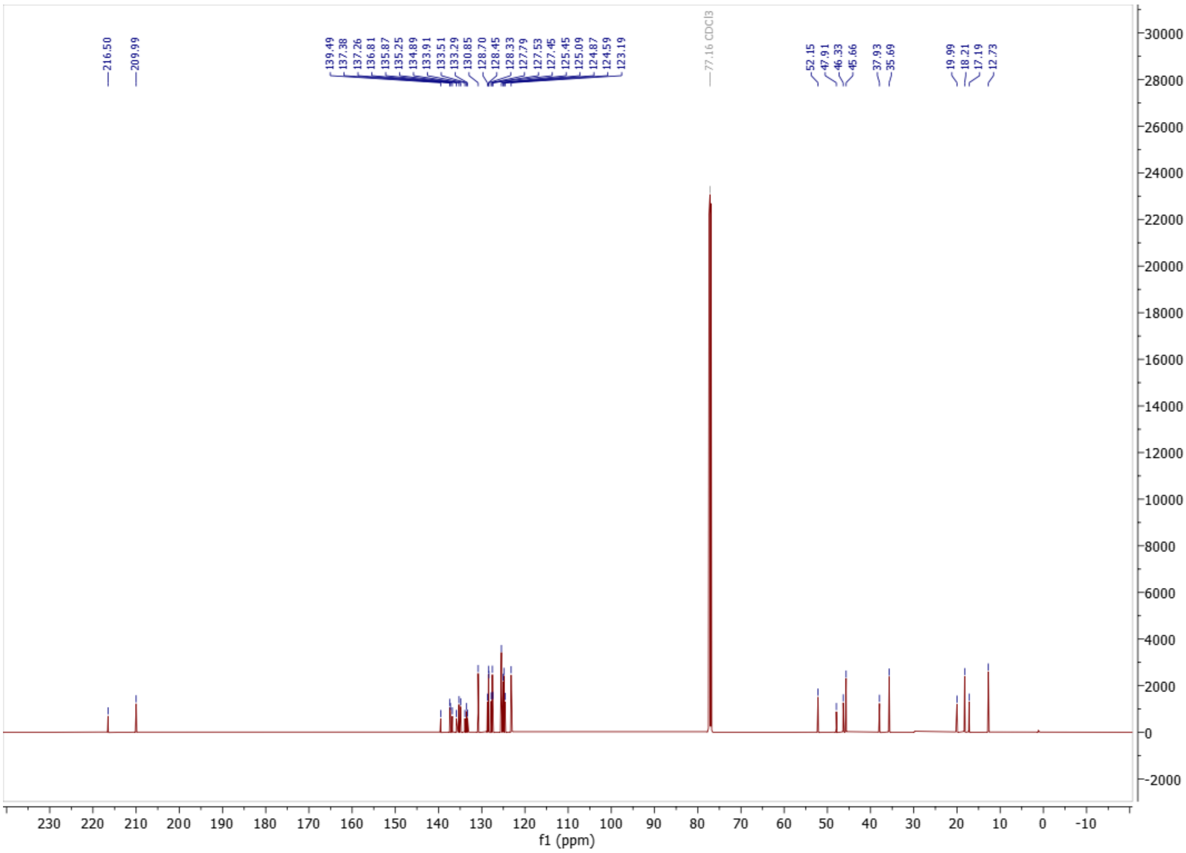

(1*R*,3a*R*,11b*S*)-9-chloro-11b-methyl-2,3,3a,11b-tetrahydro-1*H*-cyclopenta[*l*]phenanthren-1-ol (12o)

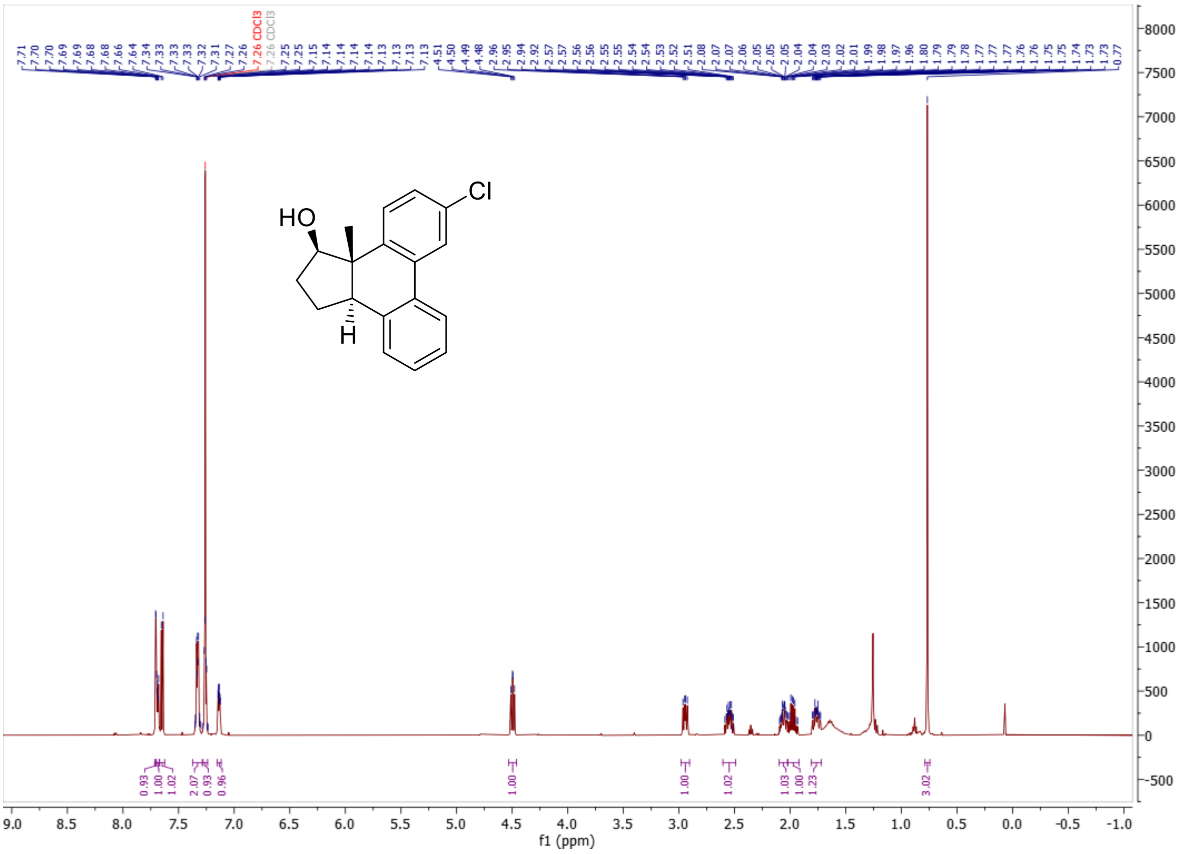

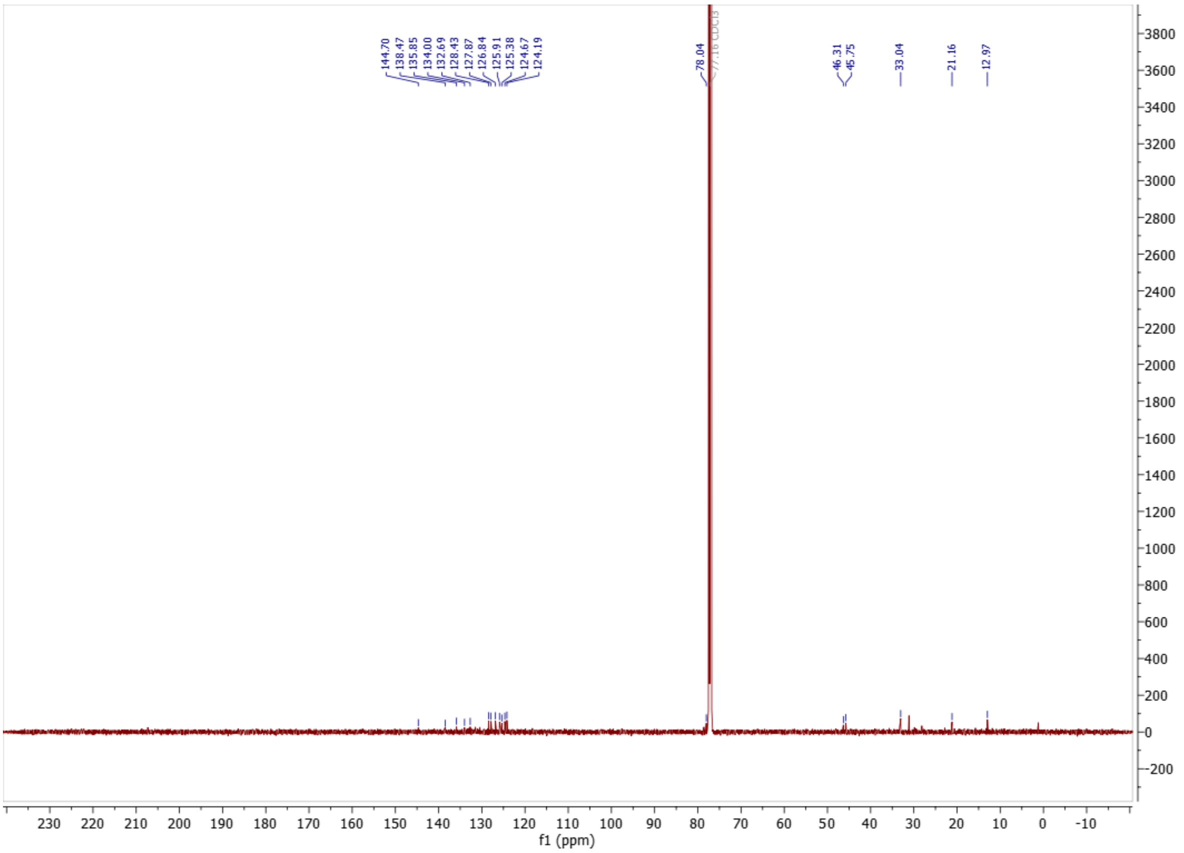

(1*S*,3a*R*,11b*S*)-9-chloro-11b-methyl-2,3,3a,11b-tetrahydro-1*H*-cyclopenta[*l*]phenanthren-1-ol (12o')

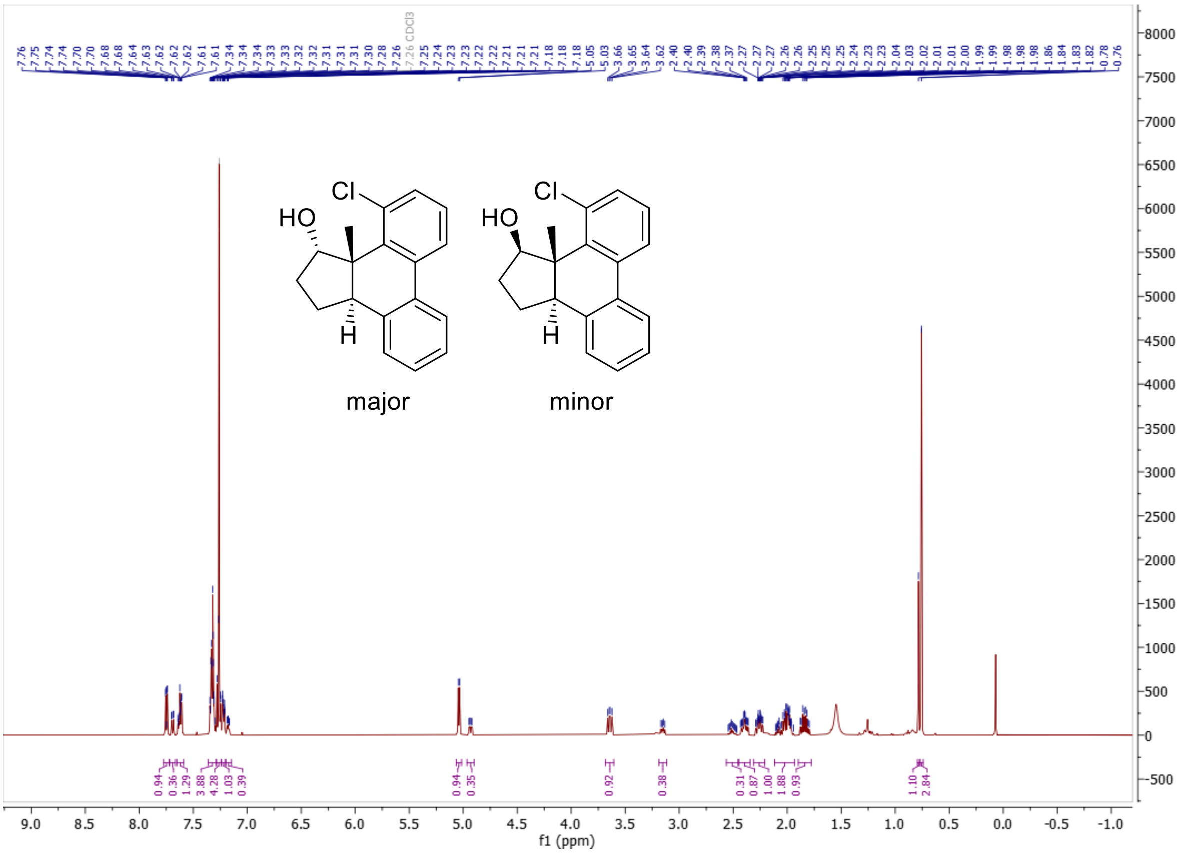

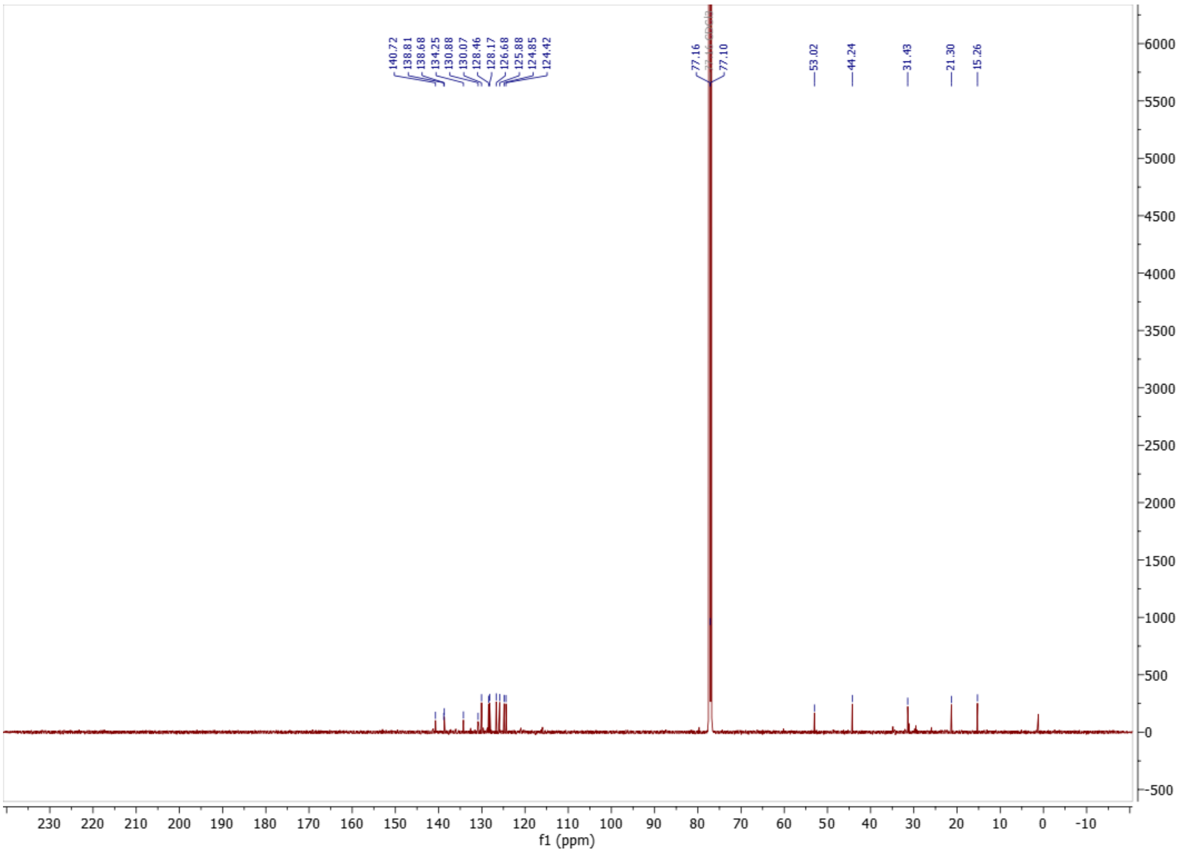

(3a*R*,11b*S*)-9-methoxy-11b-methyl-2,3,3a,11b-tetrahydro-1*H*-cyclopenta[*l*]phenanthren-1-one (11p)

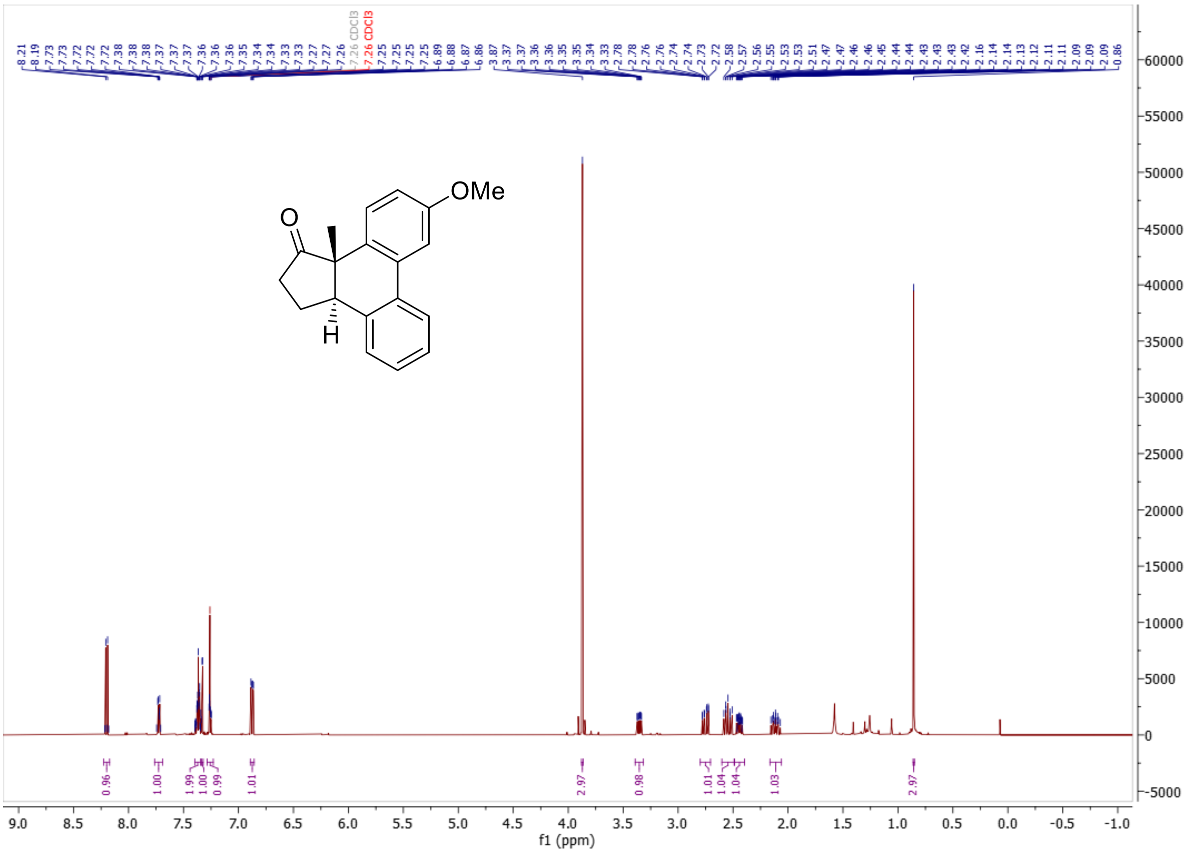

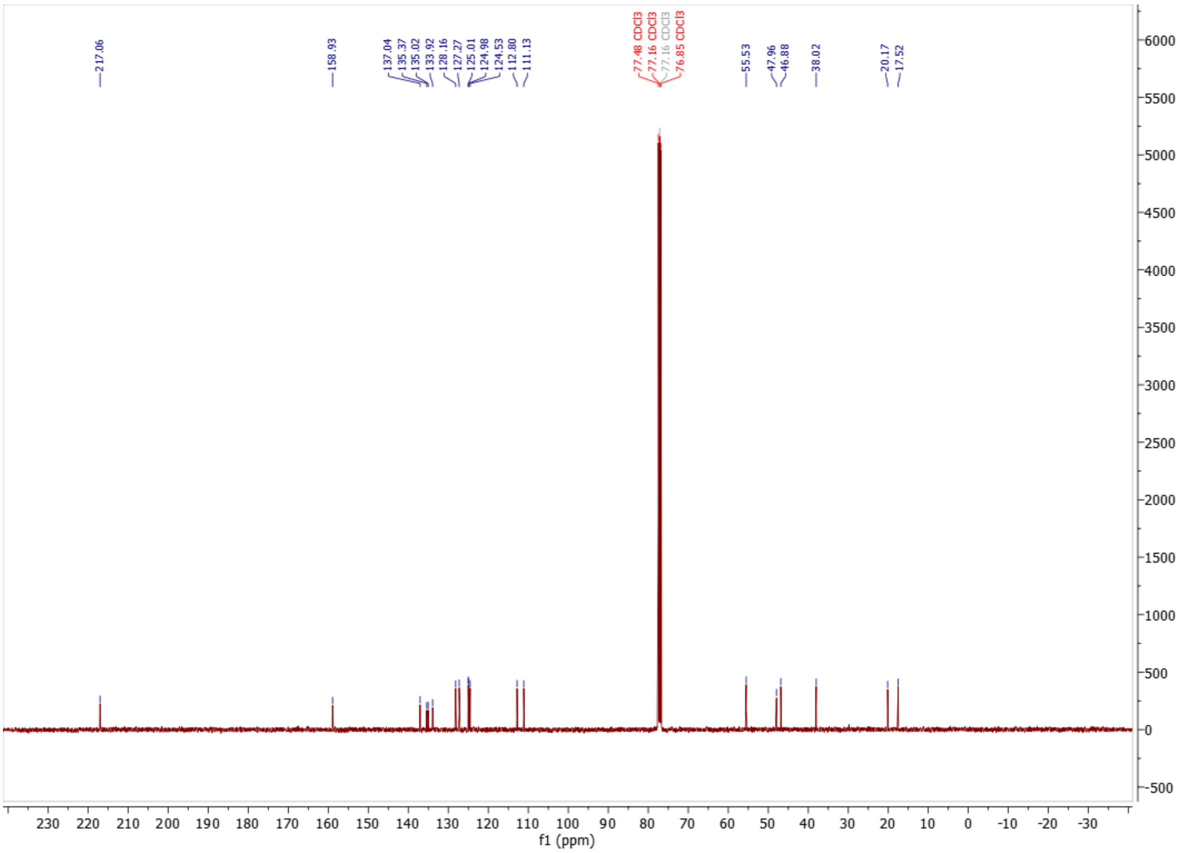

(3a*R*,11b*S*)-11-methoxy-11b-methyl-2,3,3a,11b-tetrahydro-1*H*-cyclopenta[*l*]phenanthren-1-one (11p)

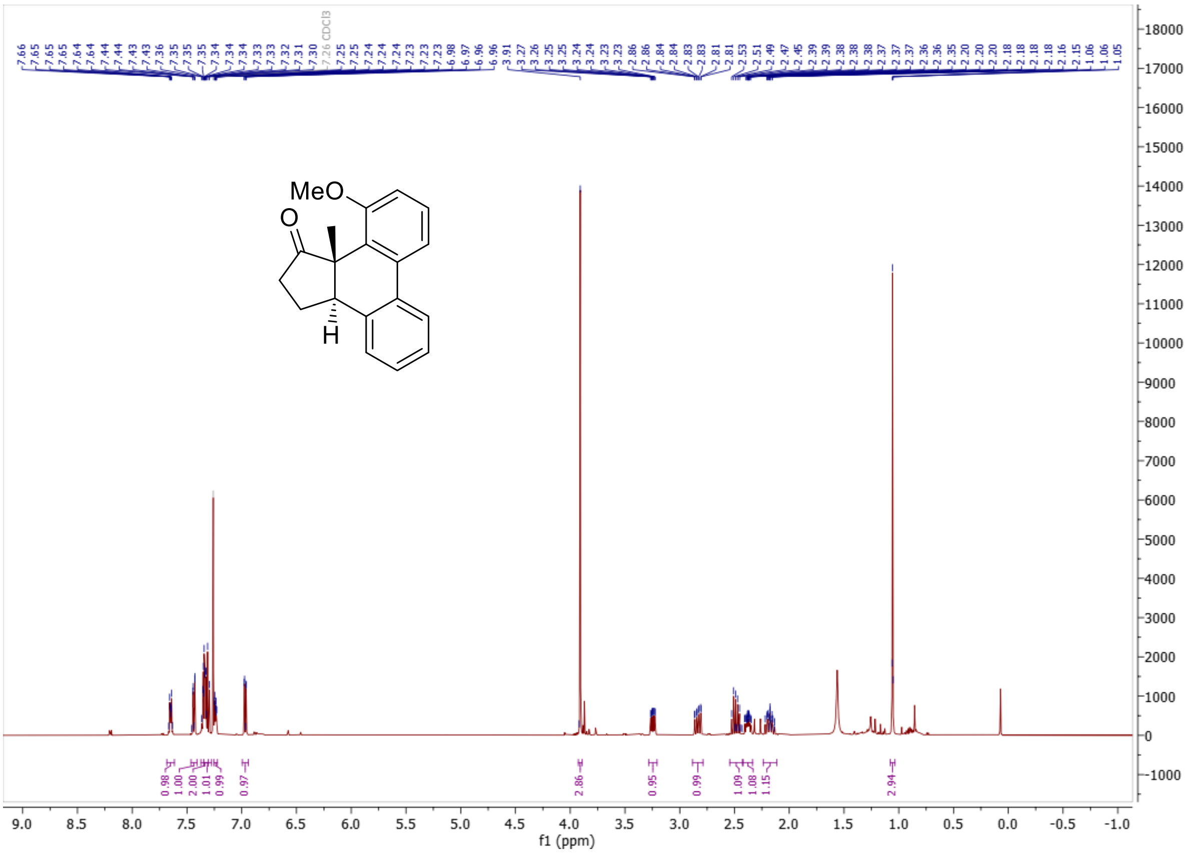

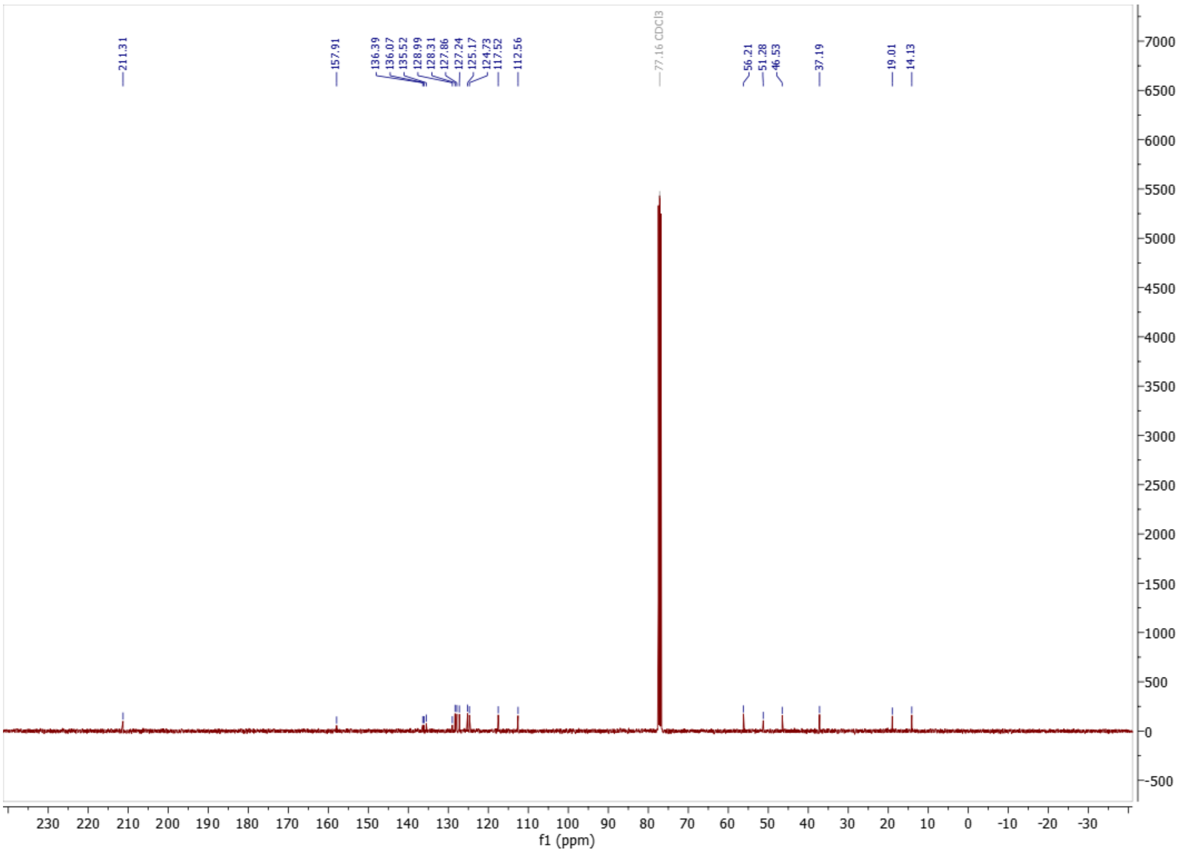

(1*R*,3a*R*,11b*S*)-11b-methyl-2,3,3a,11b-tetrahydro-1*H*-cyclopenta[*l*]phenanthren-1-ol (12a)

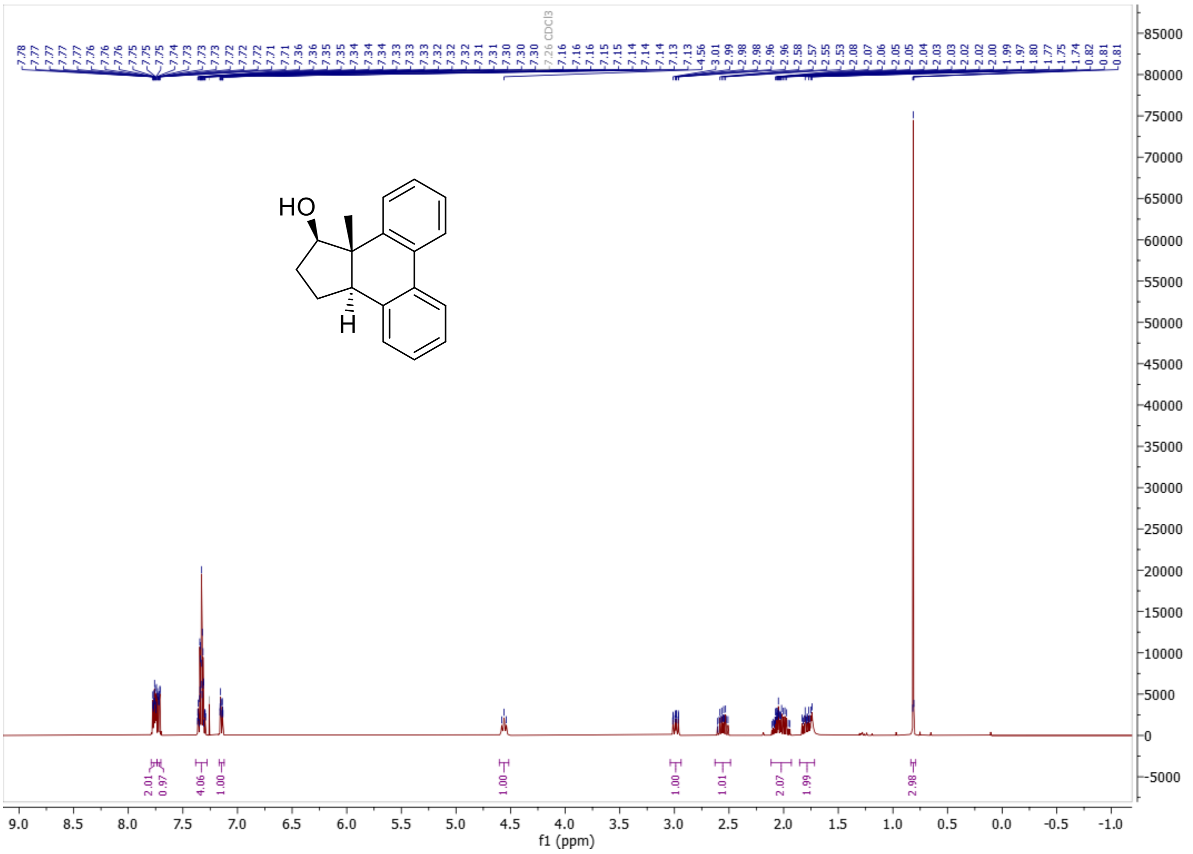

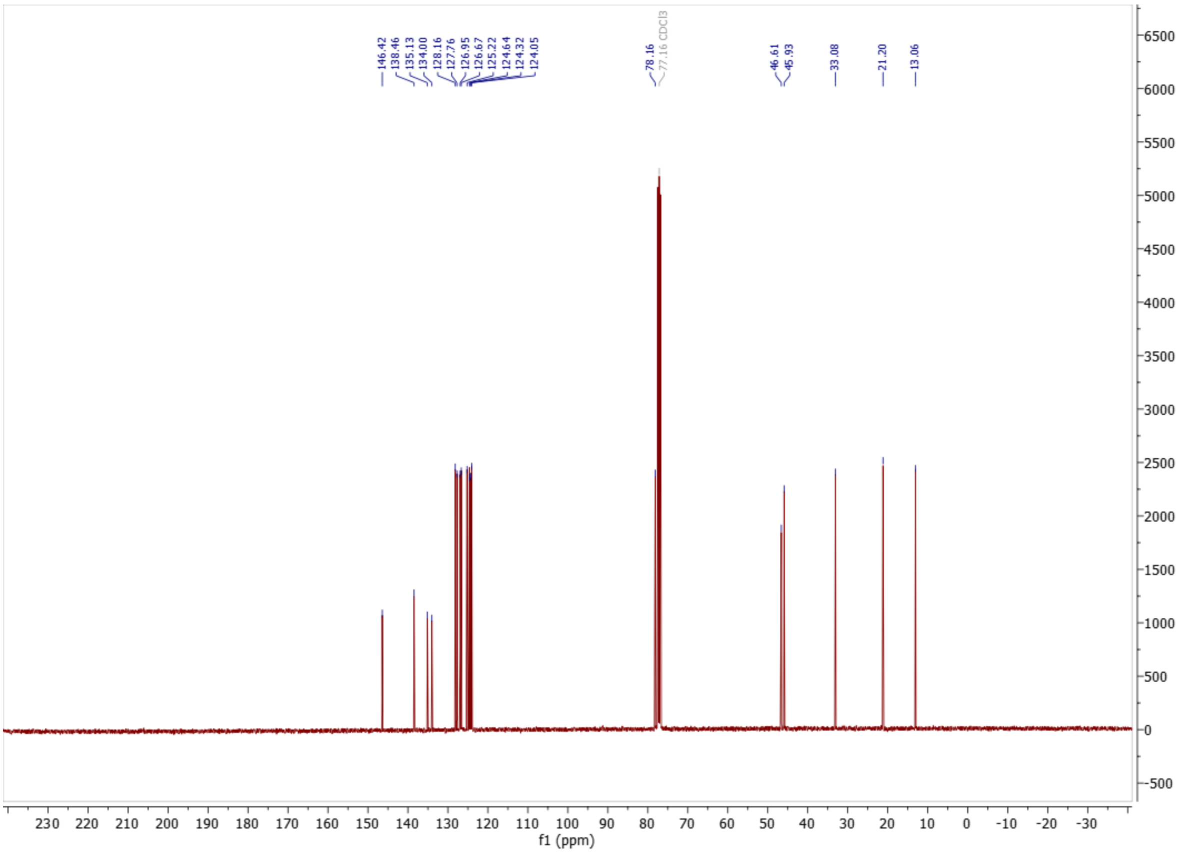

(1*R*,3a*R*,11b*S*)-11b-methyl-2,3,3a,11b-tetrahydro-1*H*-cyclopenta[*l*]phenanthren-1-yl 4-bromobenzoate (14)

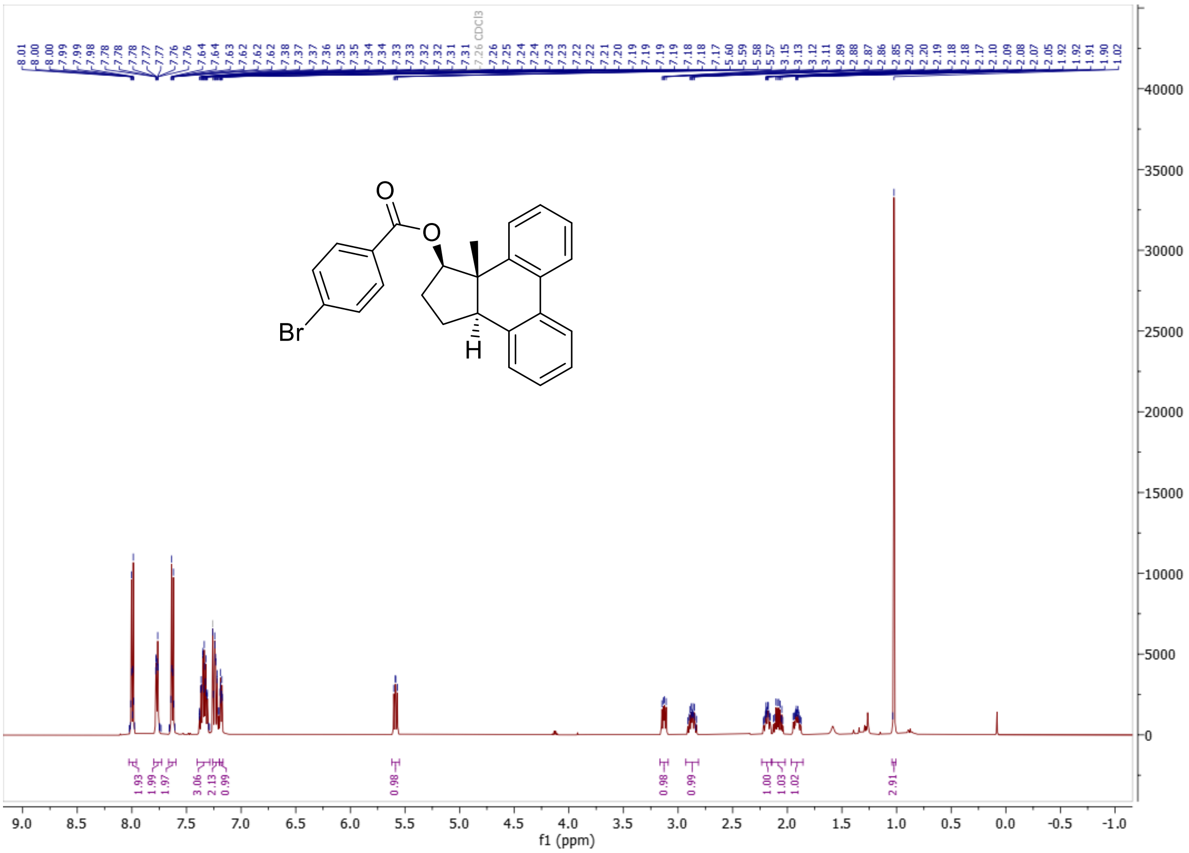

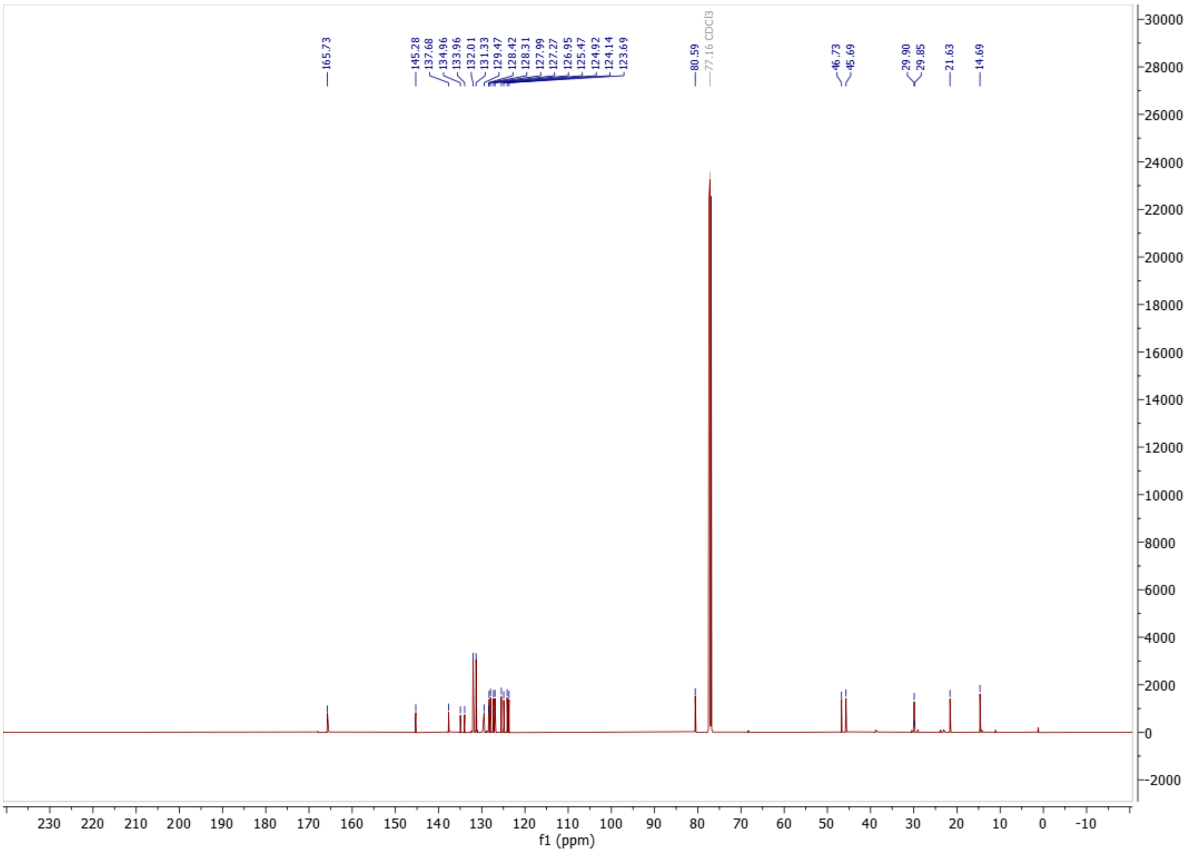

13a and *ent*-13b

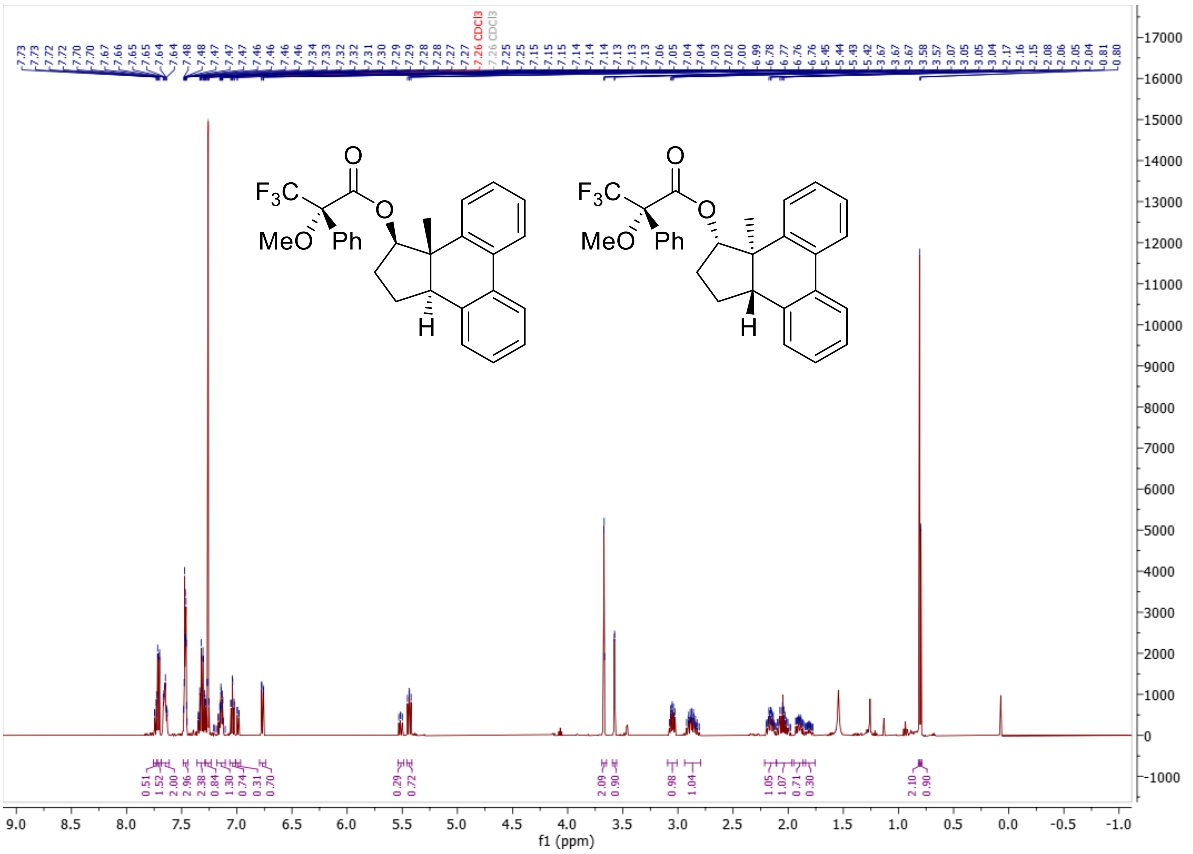

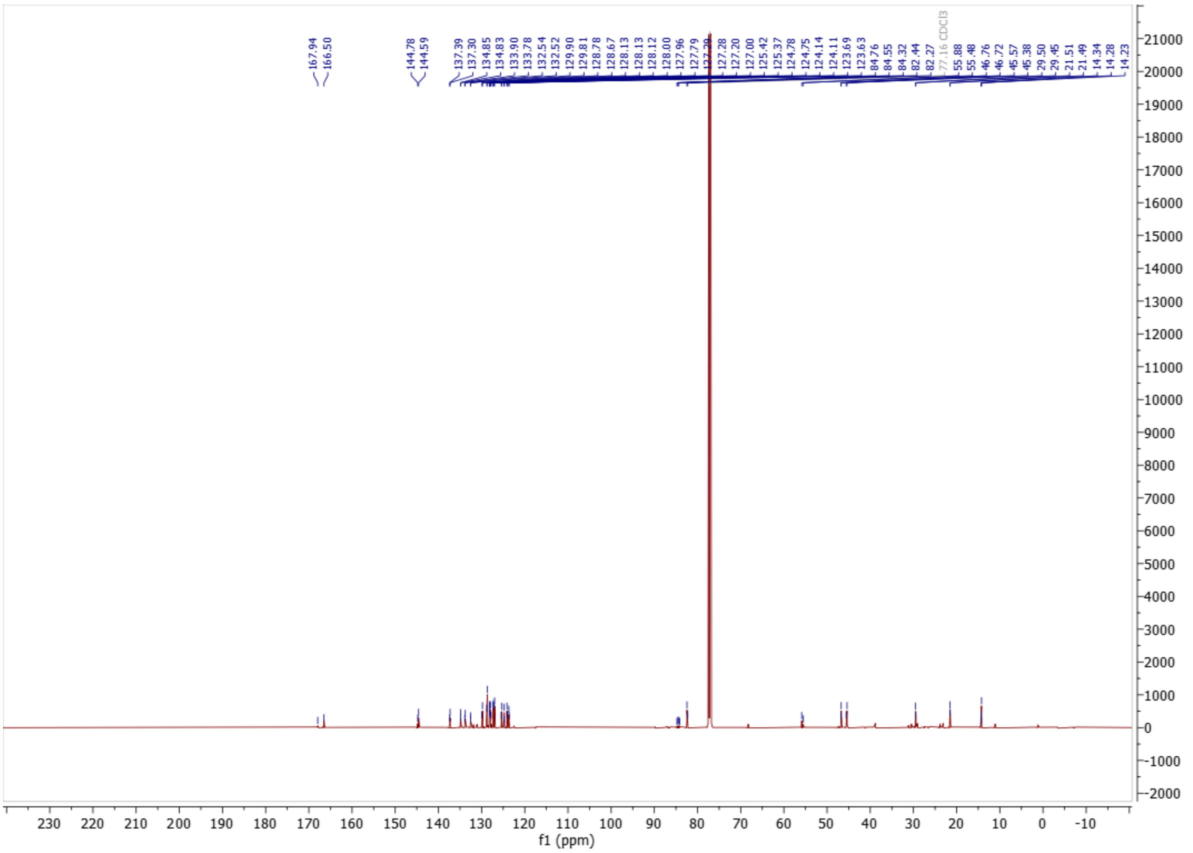

Details of DFT Calculations

Free Energy Calculations - Methods

To obtain accurate free energies, we employed a composite computational protocol. First, the electronic energies were evaluated at the DFT level using the ωB97X-D3/def2-TZVP method (E_{DFT}). These values were subsequently refined by single-point calculations using DLPNO-CCSD(T) (E_{CC}). The cc-pVTZ basis set and the ccpVTZ/C auxiliary basis sets were employed. The resulting electronic energies were then converted into Gibbs free energies by incorporating the following corrections:

i) Solvation free energy correction (ΔE_{corr}(CPCM)): Solvation effects were accounted for by single-point energy calculations on the DFT-optimized vacuum geometries using the Conductor-like Polarizable Continuum Model (CPCM) with dichloromethane (DCM, ε = 9.08) as the solvent.

ii) Finite-temperature corrections (ΔG_{th}): Thermal contributions to the free energy were obtained from a harmonic frequency analysis at the ωB97X-D3/def2-TZVP level. All corrections were evaluated at the reaction temperature (-80ºC) within the rigid-rotor/harmonic-oscillator approximation.

The final free energy can then be obtained using the following equation:

$$\Delta G = E_{\mathrm{CC}} + \Delta E_{\mathrm{corr}}(\mathrm{CPCM}) + \Delta G_{\mathrm{th}}$$

We employ the DFT electronic energy for the triplet calculations since the coupled-cluster spin contamination exceeded a value of 1.0 for all structures. All the contributions were computed with ORCA 5.0.4. References for all methods and programs employed are cited in the main manuscript.

The individual contributions to the free energy calculations are presented in Table S3 to S8.

Free Energy Calculations - Results

Table S3: Free energy values for concave conformers (substrate binding to the concave face of the catalyst). $\Delta\Delta G = \boldsymbol{\Delta G}($**C1** minimum structure) - $\boldsymbol{\Delta G}($**C2** minimum structure).

	**C1** minimum structure	**C2** minimum structure	$\Delta\Delta G$ [kJ/mol]
E_{DFT}	-10016.8428 E_h	-10016.8453 E_h	6.48
ΔE_{corr}(CPCM)	-108.51 kJ/mol	-106.57 kJ/mol	-1.93
ΔG_{th}	2160.19 kJ/mol	2162.54 kJ/mol	-2.35
E_{CC}	-10007.9276 E_h	-10007.9307 E_h	8.10
$\boldsymbol{\Delta G}$	**-10007.1462 E_h**	**-10007.1476 E_h**	**3.81**

Table S4: Free energy values for convex conformers (substrate binding to the convex face of the catalyst). $\Delta\Delta G = \boldsymbol{\Delta G}$(Conformer 1) - $\boldsymbol{\Delta G}$(Conformer 2).

	Conformer 1	Conformer 2	$\Delta\Delta G$ [kJ/mol]
E_{DFT}	-10016.8295 E_h	-10016.8315 E_h	5.25
ΔE_{corr}(CPCM)	-93.91 kJ/mol	-90.79 kJ/mol	-3.11
ΔG_{th}	2171.70 kJ/mol	2169.17 kJ/mol	2.53
E_{CC}	-10007.9167 E_h	-10007.9202 E_h	9.34
$\boldsymbol{\Delta G}$	**-10007.1253 E_h**	**-10007.1286 E_h**	**8.75**

Table S5: Details of the barrier calculation for the photocyclization reaction pathway on T_1 for the uncatalyzed substrate (see Figure 4 in the main manuscript). Here, due to significant spin-contamination in the DLPNO-CCSD(T) calculation, the final free energy is approximated as: $\Delta G \approx E_{\mathrm{DFT}} + \Delta E_{\mathrm{corr}}(\mathrm{CPCM}) + \Delta G_{\mathrm{th}}$.

	T_1^{min} (**9a**)	T_1^{TS} (**15a**)	$\Delta\Delta G$ (T_1^{TS} - T_1^{min}) [kJ/mol]
E_{DFT}	-770.7475 E_h	-770.7361 E_h	29.82
DFT spin contamination	0.0636	0.0702	
ΔE_{corr}(CPCM)	-42.45 kJ/mol	-39.64 kJ/mol	2.82
ΔG_{th}	689.91 kJ/mol	692.55 kJ/mol	2.64
E_{CC}	-769.2673 E_h	-769.2612 E_h	15.86
CC spin contamination	1.1572	1.0352	
$\boldsymbol{\Delta G}$	**-769.0207 E_h**	**-769.0125 E_h**	**35.28**

Table S6: Details of the barrier calculation for the 1,5-hydrogen shift (see Figure 4 in the main manuscript).

	S_0^{min} (**15a**)	S_0^{TS} (**11a**)	ΔΔG ($S_0^{TS} - S_0^{min}$) [kJ/mol]
E_{DFT}	-770.7753 E_h	-770.7475 E_h	72.88
ΔE_{corr}(CPCM)	-35.09 kJ/mol	-34.98 kJ/mol	0.11
ΔG_{th}	703.65 kJ/mol	693.85 kJ/mol	-9.79
E_{CC}	-769.3069 E_h	-769.2791 E_h	72.90
$\boldsymbol{\Delta G}$	**-769.0523 E_h**	**-769.0282 E_h**	**63.22**

Table S7: Details of the barrier calculation for the photocyclization on T_1 for **C1**. Here, due to significant spin-contamination in the DLPNO-CCSD(T) calculation, the final free energy is approximated as:

$$\Delta G \approx E_{\mathrm{DFT}} + \Delta E_{\mathrm{corr}}(\mathrm{CPCM}) + \Delta G_{\mathrm{th}}.$$

	T_1^{min} (**C1**)	T_1^{TS} (**C1**)	ΔΔG ($T_1^{TS} - T_1^{min}$) [kJ/mol]
E_{DFT}	-10016.7529 E_h	-10016.7406 E_h	32.52
ΔE_{corr}(CPCM)	-112.17 kJ/mol	-108.50 kJ/mol	3.67
ΔG_{th}	2152.68 kJ/mol	2156.11 kJ/mol	3.43
$\boldsymbol{\Delta G}$	**-10015.9757 E_h**	**-10015.9607** E_h	**39.62**

Table S8: Details of the barrier calculation for the photocyclization on T_1 for **C2**. Here, due to significant spin-contamination in the DLPNO-CCSD(T) calculation, the final free energy is approximated as:

$$\Delta G \approx E_{\mathrm{DFT}} + \Delta E_{\mathrm{corr}}(\mathrm{CPCM}) + \Delta G_{\mathrm{th}}.$$

	T_1^{min} (**C2**)	T_1^{TS} (**C2**)	ΔΔG ($T_1^{TS} - T_1^{min}$) [kJ/mol]
E_{DFT}	-10016.7542 E_h	-10016.7392 E_h	39.20
ΔE_{corr}(CPCM)	-108.89 kJ/mol	-109.86 kJ/mol	-0.97
ΔG_{th}	2152.88	2151.33 kJ/mol	-1.55
$\boldsymbol{\Delta G}$	**-10015.9757** E_h	**-10015.9617** E_h	**36.68**

Calculation of Crossover Temperature

The crossover temperature is given by,

$$T_C = \frac{\hbar\omega}{2\pi k_B}$$

where, ω is the absolute value of the imaginary frequency of the transition mode.

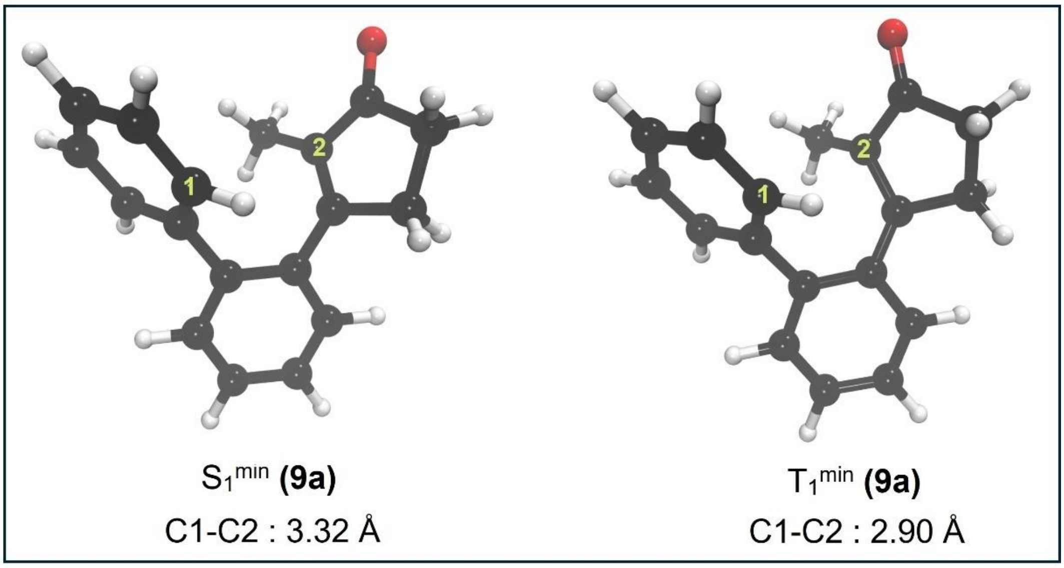

Figure S2: Optimized structures of **9a** in the S_1 and T_1 state. S_1 is optimized at TDA-ωB97X-D3 and T_1 with wB97X-D3 in combination with the def2-TZVP basis set. The distance between the bond-forming carbon atoms during photocyclization is also shown.

Cartesian Coordinates of all Equilibrium Structures

S_0^{min} (**9a**)

C	-0.45548640544766	1.09111420665607	1.08102627870027
C	0.91109777121827	1.21160626109852	1.31896313490347
C	1.52517522517368	2.45281915274636	1.30615738929382
C	0.78125027540065	3.59708638212259	1.05729654083034
C	-0.58158330968175	3.49126877995285	0.82943983953133
C	-1.19389093985367	2.24776111623123	0.84480456714678
C	-1.13216179578997	-0.23301949929906	1.09493447742169
C	-0.69324436288572	-1.31536284195923	0.31767561870126
C	-1.36794508995926	-2.53281440606544	0.40925836854802
C	-2.46980123528844	-2.68714744425984	1.23056396774282
C	-2.90972381165146	-1.61571523764291	1.99168656110987
C	-2.24193744084677	-0.40649102908106	1.92127324510481
H	1.49700587996580	0.32374077163495	1.52631045748326
H	2.58945325169157	2.52832474439905	1.49363356200429
H	1.26341876366371	4.56683561953255	1.04286924792365
H	-1.17072189223743	4.37834711850393	0.63063648439053
H	-2.25677550181114	2.16687405912245	0.64708738402200
H	-1.03471828294461	-3.36550127260196	-0.19949816172709
H	-2.98353711335127	-3.63961882702969	1.27393818233830
H	-3.76701760473007	-1.72304513937067	2.64490072973397
H	-2.56872567236708	0.42623426208388	2.53321185662412
C	0.44764778392839	-1.22354186916556	-0.62110807406703
C	0.55032098056033	-0.41238856812144	-1.68130482430326

C	1.64550557028430	-2.13021589984472	-0.45716313569071
C	1.84060647478557	-0.65935373486271	-2.36473182027072
C	2.58437931749379	-1.74119567246530	-1.59661946896460
H	1.34326549295502	-3.17978355580297	-0.50424670968845
H	2.09424104230229	-1.98981108642549	0.53019240672500
H	3.53505852537403	-1.33745405274595	-1.24555535093174
H	2.81575060441498	-2.56517606469777	-2.27239140834720
C	-0.40782609384689	0.60109448294927	-2.19799399282909
H	-0.07723213327959	1.60836281009133	-1.93266664942978
H	-0.44713846070862	0.55185087010987	-3.28701937490025
H	-1.40820049276288	0.45349150596430	-1.79156410499597
O	2.23109585150605	-0.10578698580110	-3.36291213983350

T_1^{min} (**9a**)

C	-0.53533738912569	1.10744698635541	0.96780505190534
C	0.85036791713584	1.23702170703791	1.07399464957085
C	1.45845143115013	2.47877480546158	0.97183827789487
C	0.69134919158849	3.61409481526208	0.76879439065853
C	-0.69100517848927	3.50247702664106	0.68721269610502
C	-1.29711027863886	2.26319586691304	0.79163880249900
C	-1.17647654022195	-0.22037660891153	1.06661522972531
C	-0.64622559345515	-1.36676996654568	0.38206595361566
C	-1.17689495240103	-2.64069056057305	0.75083446024641
C	-2.22777794989551	-2.76304907880768	1.62101893669636
C	-2.79485913580044	-1.62678318726190	2.20371376082622

C	-2.25128240263118	-0.38116123389154	1.93035212167585
H	1.45264671140353	0.35495859219412	1.25662281396125
H	2.53600713078874	2.55647120587791	1.04738564592988
H	1.16597245441770	4.58332378565788	0.67808455821281
H	-1.29744625695241	4.38590485490094	0.52788969913342
H	-2.37358727046461	2.17981486898861	0.69589351482235
H	-0.77389526055804	-3.52772908607099	0.27930467481031
H	-2.62351135765870	-3.74535200445963	1.84933216668125
H	-3.62494616767208	-1.71893685238711	2.89238758342426
H	-2.63494644627489	0.49107507399771	2.44715588004915
C	0.29997963971014	-1.30624796424738	-0.64614782956423
C	0.72896880718457	-0.14026915003854	-1.47449515768081
C	1.20754894746820	-2.43888515517669	-1.06043770120280
C	2.08708874983589	-0.35991279047910	-1.90248481274457
C	2.53116502449274	-1.72388708056699	-1.38620558770694
H	0.82858577788115	-2.92879871030469	-1.96664793773089
H	1.31219973064997	-3.20128410783284	-0.28942000924182
H	3.13479746583814	-1.58497308077406	-0.48360815354528
H	3.14724473896677	-2.23603805094483	-2.12360446168009
C	-0.13304497051456	0.89136106943684	-2.09489616652904
H	0.16610024422008	1.89081663505598	-1.76352403802787
H	0.00005953042722	0.87792834019139	-3.17990969353936
H	-1.18445190490329	0.74638392130372	-1.85378004521934
O	2.74503872237860	0.38292651605974	-2.61642227355735

T_1^{min} (**15a**)

C	-0.68982891402643	1.02335549745971	0.75995505826839
C	0.74595287240603	1.04582840062645	0.30418685938099
C	1.27540405773616	2.42550082680357	0.07117025316317
C	0.51239508141907	3.53313116501744	0.23023500263643
C	-0.83893399426401	3.44112965195642	0.63036951121193
C	-1.40582966784957	2.17737002426228	0.87943483533859
C	-1.27947753805308	-0.30306454256050	1.00762593890350
C	-0.76953888095092	-1.42601595596488	0.29230430166302
C	-1.34858226743384	-2.68989422251570	0.53041152945105
C	-2.37602028102032	-2.84641969748937	1.43779182665269
C	-2.85195868503499	-1.75014779257690	2.14590511635259
C	-2.29618967812947	-0.49419696980720	1.92875346723689
H	1.35953514200746	0.57184549294011	1.08721407889148
H	2.30956134354337	2.50564905083731	-0.23694311705326
H	0.94660515952267	4.50976889776798	0.04901574136163
H	-1.44027682230650	4.33406098762756	0.73750216755119
H	-2.45229456612384	2.12248446762826	1.15852279470644
H	-0.98806987046018	-3.55059994849002	-0.01912264829227
H	-2.80634646305445	-3.82739194163464	1.59956769326219
H	-3.64475163133722	-1.87186464819425	2.87338871941013
H	-2.65067620226401	0.35008087791953	2.50797373823248
C	0.28689718734480	-1.24454031597090	-0.62592552596215
C	0.87198768194699	0.10000736342980	-0.93271860648201
C	1.03919203441887	-2.32778698593777	-1.34094972183142

C	2.31614989416224	-0.24405859066506	-1.31166602696543
C	2.43630187481075	-1.73816910042179	-1.56714816533755
H	0.55786372819555	-2.57251036865311	-2.29599899105777
H	1.07348798381382	-3.25419020412191	-0.76601173453588
H	3.16430858585051	-2.12968002257624	-0.85289258964399
H	2.84788921900151	-1.90742892781167	-2.56258276827620
C	0.21509367143693	0.73227125610816	-2.17612178293006
H	0.73423584404996	1.65221745674744	-2.44758610313978
H	0.24802823972414	0.05097637503220	-3.02879365982205
H	-0.82953577992453	0.96501642642241	-1.96544139794283
O	3.22451782622677	0.53722944291280	-1.40113287615139

S_0^{min} (**15a**)

C	-0.68127897629354	0.98726916930094	0.79087557503424
C	0.70841900373627	1.05364955154243	0.17402823645072
C	1.17478131407350	2.44383876926325	-0.15459490949562
C	0.55855312044764	3.52615089945142	0.31486332439298
C	-0.64492295581107	3.40553350787793	1.12063277825441
C	-1.22572115551996	2.21437947870038	1.33250512150816
C	-1.30289371182493	-0.22271401253378	0.89391677471399
C	-0.65622442689923	-1.42910561533130	0.33967679064205
C	-1.19908064969917	-2.72622859312464	0.71302488108457
C	-2.33386017271400	-2.82968693034814	1.41822684486521
C	-3.05143609486458	-1.64444096285332	1.83518746398860
C	-2.56467728184690	-0.41862618935187	1.58415781586414

H	1.39176004584432	0.67568753417317	0.96242729036451
H	2.07267851967813	2.52431413969168	-0.75379334357916
H	0.94519952973078	4.51430849496719	0.09597482881410
H	-1.08923941880502	4.30291742618456	1.53411306232194
H	-2.13919883726482	2.17372746890918	1.90993983674217
H	-0.68429530139828	-3.61757756508395	0.37665781522880
H	-2.73398169914677	-3.80425820446910	1.67019618037757
H	-3.99005556036957	-1.75672646619494	2.36397080608148
H	-3.12214493368762	0.44607595879420	1.91665621501112
C	0.36388094674411	-1.29479135759857	-0.52683295077007
C	0.83407154439179	0.06369604104400	-0.98519950140675
C	1.17427733967436	-2.36616652994634	-1.20420293311841
C	2.28026173297079	-0.20830977716721	-1.42275209452340
C	2.53857781317808	-1.70673446505102	-1.42425518225052
H	0.72108804935996	-2.63180625315140	-2.16596845567643
H	1.23906998544957	-3.28281043990617	-0.61980679367771
H	3.21339563403934	-1.90789308523199	-0.58668961010669
H	3.05793175459150	-2.00366946580123	-2.33492046684946
C	0.04980966568797	0.50994402091936	-2.23648527192619
H	0.44817683895748	1.44750434760940	-2.62490596259241
H	0.11595769926209	-0.24123906660006	-3.02574007765089
H	-1.00226754361928	0.64997558646861	-1.98460406337080
O	3.08918905566258	0.62301761264008	-1.73658798963116

S_0^{min} (**11a**)

C	-0.61046584031846	1.05762285125099	0.79831046761557
C	0.41882279373163	1.22753545153008	-0.14612605703968
C	1.02045302318295	2.46889469807756	-0.29729054764531
C	0.64036094968553	3.53684286218008	0.50129903967217
C	-0.34927633055173	3.36955272765117	1.45577035563385
C	-0.96763913511250	2.13938903511965	1.59894297340421
C	-1.29531926698190	-0.25618962596600	0.92421163771251
C	-0.62843522802332	-1.41646650664090	0.50129937341785
C	-1.24768719104997	-2.65049326468855	0.60418916841625
C	-2.53196749105029	-2.75474927563561	1.11977171461279
C	-3.20352963488611	-1.61290928089082	1.52260863523550
C	-2.58957795707681	-0.37414644205621	1.42013045351881
H	1.39804078040439	-0.96119299900276	0.80490259130380
H	1.79873863235523	2.59115811939029	-1.03860376245916
H	1.12671993942063	4.49763064943572	0.38300515251998
H	-0.63767446730935	4.19514789337224	2.09516055681715
H	-1.72775208298743	2.01367579873579	2.36046650821310
H	-0.72596712254472	-3.54186007285085	0.27492148217629
H	-3.00944169025534	-3.72422762316204	1.19461795863765
H	-4.21307584687354	-1.68202545588793	1.90915374412592
H	-3.13822655676122	0.51333627755551	1.71111963184151
C	0.74966158788333	-1.21583085877443	-0.04713026673241
C	0.78800539569393	0.01551431609289	-0.96827433465812
C	1.46021060541404	-2.31595411827519	-0.82944012743908

C	2.19092097877494	-0.09909802797491	-1.56376156271341
C	2.63228358747765	-1.55840912171946	-1.46433259731774
H	0.80276730550938	-2.73672021289440	-1.59354349386389
H	1.78780769414859	-3.13753113537052	-0.19210209859600
H	3.51869527052845	-1.57937801207852	-0.82427782368822
H	2.94335709383830	-1.92227887573427	-2.44363785659101
C	-0.16259913541210	-0.10374687523323	-2.17948075709091
H	-0.06832318818713	0.79175562735547	-2.79384089196948
H	0.07025602476898	-0.96833014522996	-2.80427190054194
H	-1.19715104243838	-0.19362695507531	-1.84764074572018
O	2.83531799152099	0.77596150196494	-2.07569387029292

C1 minimum

C	0.84186071036683	-0.81940448027344	6.41078167929420
C	1.91107240778945	-0.19287089748906	5.77370750218255
C	3.05165161828459	-0.90192632265473	5.43721358106672
C	3.14556180128149	-2.25236949128726	5.74229242110330
C	2.09714605023298	-2.88254629525852	6.39471869805794
C	0.95470206585030	-2.17050015306953	6.72754061661996
C	-0.39563198722928	-0.06716134635455	6.74857592642410
C	-1.08872536973503	0.69141718644874	5.79231787296084
C	-2.20729654067017	1.42960485864215	6.17485054853868
C	-2.66975964194919	1.39565545024354	7.47806557432165
C	-2.00038304947515	0.63218639558740	8.42162133270965
C	-0.87308054305650	-0.08343977070264	8.05707112898262

H	1.85429418021639	0.86588563510966	5.54752354747049
H	3.87044414086194	-0.39738446543839	4.93876302298538
H	4.03565357726488	-2.80919279027596	5.47633209859745
H	2.16367358228304	-3.93577335218300	6.63896558711020
H	0.12840868779561	-2.67381159837212	7.21699155955349
H	-2.73609299238324	2.01621626047049	5.43228677683845
H	-3.54916090862802	1.96342287625501	7.75495650107327
H	-2.34859636090094	0.60377734122638	9.44676918799096
H	-0.33132097694404	-0.65369719330965	8.80245994581134
C	-0.66579463868944	0.72417934591567	4.37925797712796
C	-0.57572984123834	-0.33407350838062	3.54752275078572
C	-0.27832258146180	2.01316269447245	3.71204046710908
C	-0.04429022317586	0.12742753899539	2.28542591282136
C	0.28981864230435	1.58389747120269	2.36287932662045
H	0.43301285853541	2.57343233791784	4.32179400557080
H	-1.15941995988486	2.65215667088485	3.59873897423009
H	-0.07778264997755	2.12688551986526	1.49336427485874
H	1.37986131542880	1.66893797739485	2.33318309923507
C	-0.92353488393226	-1.76230067793282	3.77911659886751
H	-1.51632196382910	-2.14120058676511	2.94481421841431
H	-0.01904061509226	-2.37126700767854	3.84488302826523
H	-1.48969304108248	-1.88757699775411	4.70060878551863
O	0.11249803557251	-0.63359307754839	1.31876772319163
Br	3.75796154239689	-2.10195681830525	-1.99395127133183
Al	1.67982029530984	-1.41694777632700	-2.70470729821788

N	0.45467590955076	-1.37728028013099	-1.10917744791968
C	0.69277851391667	-2.70509103804651	-0.42709152906303
H	1.41270339244650	-3.29972432606521	-0.98592306849311
H	1.12079590203895	-2.52704845371824	0.55571449780015
C	-0.66044860935172	-3.41037813813748	-0.35345200221358
H	-0.77206831688026	-3.96923812352454	0.57606606798356
H	-0.74717676016909	-4.11514402752162	-1.18226685025326
C	-1.01156202804068	-1.33150102619864	-1.46903873809182
H	-1.13347909777254	-1.71738201950699	-2.47692623449119
C	-1.68733306301154	-2.29892156203681	-0.50102353798152
H	-1.88691785366145	-1.81331941930165	0.45409727236693
H	-2.63969086234695	-2.65143734917436	-0.89442857474668
C	-1.42445505457686	0.16717159518238	-1.39336561898182
O	-0.40428794620998	0.78453857416514	-0.62521941470836
B	0.61005395199653	-0.09969615412126	-0.20356233257631
C	-2.78271417457560	0.32992601056321	-0.70653335645924
C	-3.93838390469373	-0.14545575194172	-1.32410644164766
H	-3.87568113265617	-0.58146800735366	-2.31517082159955
C	-5.17202650254510	-0.06386049737278	-0.70214899546205
C	-5.24644024265908	0.51220136196054	0.56704225859586
H	-6.20909697185048	0.57698417767189	1.06551680028606
C	-4.11862261278488	1.01096718000266	1.19375100792228
C	-2.89160923539700	0.91907707143076	0.53799913927547
H	-2.00609734027289	1.32719077529006	1.00368383421631
C	-4.18816925855845	1.62443971874328	2.56597192644466

C	4.25048957621178	0.44899787259344	1.09172970415284
C	2.99518588489556	-0.09568158304939	0.87862186863698
C	-6.41541016299064	-0.57317220099838	-1.37800213530481
Br	1.83180518488099	0.53468321247693	-3.87729091242070
Br	0.69887701181377	-3.06447730388383	-4.01561206225370
C	-1.48718268735466	0.86574068443865	-2.75322369412060
C	-1.25217239493151	2.23721128659941	-2.78814450351102
H	-1.01372822300339	2.75350505820592	-1.86622211850761
C	-1.27914972197904	2.93786848783139	-3.98029491861292
C	-1.55889383660949	2.24534389542606	-5.15735526778997
H	-1.56467282415691	2.78207918378135	-6.10133857589827
C	-1.81379412065066	0.88563331460113	-5.14944631244146
C	-1.78849553690127	0.20780980537287	-3.93178669790147
H	-1.97334445981311	-0.86012654490777	-3.94710064714550
C	-0.96337243791356	4.40773424181687	-4.01781868892544
H	-6.94386595677356	-1.28794326410318	-0.74383089107083
H	-6.17967139445760	-1.06651859552706	-2.32076444460188
H	-7.10529546907730	0.24609837836218	-1.59226941727445
C	-2.05801516808566	0.12405024571918	-6.42202900677454
H	-3.74034202278119	2.62130557398294	2.57315619368369
H	-3.64530515103443	1.01005186766147	3.29027736146396
H	-5.21739836745648	1.71621307057380	2.91297928949209
C	3.66699759896523	2.28592278272923	-0.38277084753638
C	2.41108562933749	1.71086706894686	-0.56873384357933
C	2.05670285955675	0.51730990389230	0.04405753000591

C	4.56937074776766	1.64651398816368	0.45307307814693
H	5.55377246739463	2.08114436839888	0.60338230077976
H	-1.06306650065919	4.85939939129767	-3.03030493632844
H	0.06320944174724	4.57013291219713	-4.35620462408748
H	-1.62360553771267	4.93985249316601	-4.70482450532414
H	-2.16643656460257	0.79568482743418	-7.27380272369408
H	-1.22125421863175	-0.54867452711338	-6.62494104101841
H	-2.96023066456522	-0.48716557882154	-6.35309414424463
H	2.75277968062020	-1.03425472373231	1.36965900294097
H	1.69640408747761	2.19764162597955	-1.22387109410953
C	5.26713730581781	-0.25744996547082	1.94573683018686
H	4.80412834732159	-1.05195475803055	2.53296448110936
H	6.03751623160921	-0.71745298318025	1.32230546049827
H	5.76726794509167	0.43148955653015	2.63005428719796
C	4.03375063493375	3.54910652374927	-1.11085384789405
H	4.13323162875652	3.35184532240613	-2.18083527491742
H	3.26316531528715	4.31341478702027	-0.99247826146741
H	4.97909639828092	3.95844905333423	-0.75323347250266

C2 minimum

C	-5.91740229215738	0.40318558792528	-1.00765419524062
C	-6.24646247390613	0.52846258072972	-2.35601207798692
C	-5.60272335186738	1.45498654727120	-3.16115445819881
C	-4.61897572378651	2.27341207069908	-2.62791297561539
C	-4.29711364139374	2.17420902167480	-1.28194239330449

C	-4.94755689076448	1.25144490730039	-0.47884822102649
C	-6.59521632264375	-0.63131208620136	-0.18097307116370
C	-5.89817072886987	-1.54567253392168	0.62768065005710
C	-6.61357974732742	-2.47204666504931	1.38809125326001
C	-7.99364116563611	-2.52819132548969	1.34017302605494
C	-8.68416414693738	-1.63052369048087	0.54131111553921
C	-7.98794061912035	-0.69469101165563	-0.20195936212240
H	-6.99225743445617	-0.13094176893185	-2.78544830344771
H	-5.86293695488432	1.52745806598565	-4.21021641077857
H	-4.10481972550455	2.99031246432368	-3.25639335236887
H	-3.53767206647894	2.81568569854103	-0.85182147105613
H	-4.69084907925699	1.19269661252659	0.57268659442100
H	-6.07333881431019	-3.17922325515730	2.00640661889734
H	-8.52651604251894	-3.26602846190660	1.92661695616663
H	-9.76637091749279	-1.65212517935877	0.50397403265145
H	-8.53014734705671	0.02384425879710	-0.80499790067904
C	-4.42559692337378	-1.56725132357310	0.72119753140372
C	-3.54100459866446	-1.63237233940182	-0.29635736083478
C	-3.74608569580306	-1.47038275856217	2.06173802773371
C	-2.21878727163444	-1.45067595032088	0.25591069968617
C	-2.25525702528442	-1.52562358069879	1.74665769175234
H	-4.07592795516643	-2.26602998052767	2.73243713778974
H	-4.02457835933075	-0.52855624945081	2.54334234471438
H	-1.65480135195133	-0.73855018247674	2.19710794928187
H	-1.78602986361029	-2.47094186428240	2.03615186168428

C	-3.76142008570217	-1.76258252470066	-1.76240593716714
H	-4.76299311019531	-2.12817325673125	-1.98143130450264
H	-3.63441021495725	-0.79758126635122	-2.25878544049184
H	-3.03076438472105	-2.45200870424797	-2.18825333257942
O	-1.23469553124599	-1.23206393408571	-0.46588458548560
Br	3.26954298581306	-2.73610829874956	-2.00934758911362
Al	2.98598885837936	-0.58394486070894	-1.23299898017100
N	0.99744720154826	-0.24190608109677	-1.16787842815278
C	0.52537755217768	-0.80220665657176	-2.48678031088753
H	1.37079103780613	-0.92639190771965	-3.16071813764980
H	0.10508547671756	-1.79017309841629	-2.32216727152737
C	-0.47287197542443	0.19538931056718	-3.08462373635348
H	-1.40283963573797	-0.29326595298383	-3.37462047155655
H	-0.04221668593702	0.64452857402637	-3.98026194065139
C	0.56931545877445	1.20531263040072	-1.15828117660108
H	1.34208532623014	1.80510847670091	-1.63183340761532
C	-0.69794657481930	1.25210576087360	-2.01121699824686
H	-1.57516930317668	1.02058968239085	-1.40762483524433
H	-0.85316066013036	2.24569519685395	-2.42908912765808
C	0.38013619042273	1.56691219204621	0.33308676614184
O	0.06500346196232	0.33111287465074	0.94745461169393
B	0.26892044572769	-0.80126758710790	0.11995303683878
C	-0.74761015266242	2.57236018681305	0.56974739794934
C	-0.73354069505342	3.80524314692867	-0.06886021978002
H	0.06055475536320	4.03963504435789	-0.77038160356200

C	-1.70499069000577	4.76448336666667	0.18994398290942
C	-2.69872920764532	4.47339647822893	1.11738146525288
H	-3.46217093227131	5.21593112430830	1.32958031173335
C	-2.73367683609070	3.25056720076483	1.77680246450237
C	-1.75493426424319	2.30705138768584	1.48740897040157
H	-1.75675018163547	1.35602015648117	2.00219785987974
C	-3.81359635916913	2.96042961978071	2.78389979315519
C	1.06670208697383	-4.52707175160703	0.99643416380772
C	0.61497091278187	-3.39524880409103	0.33297957724922
C	-1.65525796699950	6.10285827558002	-0.49472014867580
Br	4.17444937500305	-0.30389134819519	0.69259658319163
Br	3.67787584908531	0.93966411883307	-2.84618983596116
C	1.62831158104252	2.15257695927565	1.00176157305722
C	1.80763400136476	1.92787635498958	2.36235014684721
H	1.08364351189132	1.32504309966325	2.89699889487060
C	2.90741735116955	2.43393080264620	3.03288613056277
C	3.83086375084405	3.19616787318410	2.32141404871017
H	4.70578682409484	3.58535693957829	2.83323284732149
C	3.66575078232875	3.45318351934894	0.97095909671781
C	2.54726353373038	2.93369151488162	0.32351417901515
H	2.44123312019809	3.13077208945351	-0.73669973169204
C	3.13116775773578	2.11675960630920	4.48556712800023
H	-0.97319053281324	6.77709605729494	0.02888670650330
H	-2.63752845817005	6.57573722699725	-0.51567392103662
H	-1.29853859505072	6.01027813113700	-1.52139286209804

C	4.69070590028832	4.22388675355899	0.18765904072726
H	-3.71010176507088	1.95555873208967	3.19614378901528
H	-4.80654488971816	3.04491596789794	2.33575301008484
H	-3.77310551554261	3.66471315001957	3.61723301005545
C	1.88712202477133	-3.10838808823911	2.78586248663400
C	1.42368721353496	-1.99721065803903	2.08957664339322
C	0.79153318728402	-2.11423142604163	0.85693947475889
C	1.69675711047861	-4.36453034444367	2.22696730471675
H	2.05745132484481	-5.24174409723014	2.75671670072053
H	3.62027405745344	2.94207144643215	5.00502123814913
H	2.19047920484464	1.90179901923100	4.99414133761071
H	3.77165262610230	1.23652836062864	4.58731040907470
H	5.21970423784945	3.55609905322663	-0.49667586567106
H	4.22505331786616	5.00697680692667	-0.41397386668995
H	5.42656273895153	4.68953330543540	0.84346729979850
H	0.13038249400080	-3.52355126093670	-0.63107980194084
H	1.57781095546830	-1.00912093931446	2.50900855246805
C	0.93243778387223	-5.88874571162583	0.37273446450998
H	0.90628999714353	-6.67550535114015	1.12803646577084
H	0.02640614474867	-5.96373683449410	-0.23080461006617
H	1.78137994024517	-6.08801958952826	-0.28625427560498
C	2.61465083854955	-2.93310876823038	4.09015908997834
H	3.60557023205043	-2.50680069288219	3.91520341391434
H	2.08005049744029	-2.25077336630455	4.75401554303293
H	2.74402501372365	-3.88335076815805	4.60942407483766

T_1^{min} (**C1** minimum structure)

C	0.62994577452464	-1.55406347742988	4.92881836289397
C	1.66888581138573	-1.32748130390610	4.02035399115323
C	2.22575116589911	-2.37620017822855	3.30070604218600
C	1.75435571980791	-3.66568214138809	3.47554024195341
C	0.74256499944591	-3.90825207776726	4.39866701142374
C	0.19423251775038	-2.86558220743496	5.12354680510605
C	0.02108928076915	-0.43059214599695	5.67343961159967
C	-0.35447263353284	0.78207736689578	5.01319195563782
C	-0.69806864464241	1.89593129672761	5.82653342543456
C	-0.77974894353563	1.78740192508514	7.19146897980498
C	-0.49826347988333	0.57091734930093	7.81661624616239
C	-0.09176087809241	-0.51288380996072	7.05541903888640
H	2.06756947370721	-0.32607144173863	3.90394385424831
H	3.03412318819148	-2.18227023726970	2.60618405661814
H	2.18012765812221	-4.48257083177487	2.90608702363522
H	0.37460143719097	-4.91645201491053	4.54540663612718
H	-0.61216380800579	-3.05911599197083	5.82161614570461
H	-0.95495063452638	2.83212398959943	5.34635056067448
H	-1.07271601854410	2.64594429648555	7.78311822910892
H	-0.56127825375906	0.48282089334031	8.89373411234705
H	0.20453658031679	-1.43149305600733	7.54857257901020
C	-0.43079253879911	0.90700851470654	3.61508745158074
C	-0.45929289103691	-0.18855730203099	2.61920489466510

C	-0.18944964215901	2.16491999829713	2.81647636012101
C	0.28213130576038	0.22886059441900	1.48597760529584
C	0.68335296775298	1.67210088740758	1.64250650032489
H	0.30061589846921	2.94240236918543	3.40037583833799
H	-1.12682316885100	2.57768014561433	2.42792081388200
H	0.54616157730358	2.22539357442092	0.71428220611177
H	1.75264615465430	1.71792728969526	1.87280833278905
C	-1.36322753698211	-1.35679584884211	2.59055818669186
H	-2.12426562134644	-1.19259448389546	1.81784466862594
H	-0.81244806825555	-2.25667637258011	2.30205494841334
H	-1.85581594533941	-1.51803437783952	3.54562134703615
O	0.48773047729631	-0.53848628125915	0.51269535305398
Br	4.54531863476844	-1.75466762015934	-2.43068960717047
Al	2.42603956059239	-1.45653582227217	-3.27426641240260
N	1.12209327903266	-1.42260970185177	-1.75485048829788
C	1.44252971014156	-2.65938299021192	-0.95083790248615
H	2.30440292008978	-3.17738271630790	-1.36849156080010
H	1.70374625642958	-2.36643693942576	0.06343020141608
C	0.19488918946452	-3.53703291393955	-0.98443721777586
H	0.06143193897259	-4.08158647190297	-0.04859520606824
H	0.28333432138416	-4.26641023793738	-1.79098659397024
C	-0.31397395665290	-1.54672829905912	-2.21088649407212
H	-0.31872683700736	-1.95732975219754	-3.21714033364862
C	-0.94446185932261	-2.57068727033344	-1.26885635819199
H	-1.28753852474869	-2.08964177243422	-0.35376220086283

H	-1.80360035001927	-3.05840617864607	-1.72728400926286
C	-0.89399781381951	-0.09325540347115	-2.21977211420261
O	0.06630560130195	0.69348966323158	-1.54067112544958
B	1.09293219009542	-0.06977182448782	-0.94075839177875
C	-2.24947067768301	-0.00612841114681	-1.50646240857943
C	-3.34217659936462	-0.72964042268840	-1.98138224237271
H	-3.23830180994792	-1.33263121918349	-2.87662703209364
C	-4.56808970737803	-0.68994460935314	-1.34134326424030
C	-4.70332399674268	0.10742074334922	-0.20429173539807
H	-5.66160111323436	0.14858249492834	0.30510299554780
C	-3.64643353929490	0.86431313800200	0.27037895506778
C	-2.42228491215235	0.80132447746528	-0.39724979356535
H	-1.59221098959671	1.40965873419893	-0.06160798748429
C	-3.80042334945768	1.73018406667586	1.49173724143848
C	4.60224095702906	0.83915128350584	0.49762070960192
C	3.38880811845807	0.20793331287389	0.27665531534989
C	-5.73955550250302	-1.47896022404453	-1.85847169690876
Br	2.31883056936078	0.36653391088045	-4.64202571076581
Br	1.77520651818431	-3.36459551741775	-4.42815968151362
C	-1.07852355525415	0.48099976015734	-3.62653807057683
C	-0.95225685482970	1.85622468770631	-3.79379355834302
H	-0.69029685901469	2.46764098696705	-2.93925345172242
C	-1.11613747200575	2.44388254748898	-5.03568912902306
C	-1.42757423746339	1.63207627989513	-6.12468038506341
H	-1.54857101235122	2.08162806694537	-7.10575562429301

C	-1.57496505781592	0.26306284238781	-5.98380914676648
C	-1.40475562065128	-0.30031605095709	-4.72084023684063
H	-1.50951508296295	-1.37582330693137	-4.62849016336879
C	-0.91688749911609	3.92311291862079	-5.21995509243731
H	-6.11423038396188	-2.16940368179165	-1.09969396303116
H	-5.46753072487229	-2.06054550173962	-2.73912153621902
H	-6.56425256653875	-0.81829156319624	-2.13411099117069
C	-1.87355747572215	-0.61908180484919	-7.16423173364680
H	-3.39818219358620	2.73042147119852	1.31829517149779
H	-3.26884113265872	1.30428493270394	2.34882898482684
H	-4.84738839654344	1.83408254593691	1.77649441686922
C	4.00896019515013	2.48134000292606	-1.18743443001581
C	2.79658034250741	1.82244165160347	-1.37653249421404
C	2.46997706026494	0.67745000022460	-0.66226383593308
C	4.89539953910925	1.98032613110316	-0.24480525787866
H	5.84693589480778	2.48145244407689	-0.09196256641077
H	0.07304878288352	4.12721158286446	-5.63580821925198
H	-1.65459991790704	4.34220002531008	-5.90641185257370
H	-0.99365825708078	4.45414863873827	-4.27066680528682
H	-2.08380671960586	-0.03016434526945	-8.05717066607306
H	-1.02234161774283	-1.26858094197813	-7.38076254271947
H	-2.73552770795390	-1.26131615890868	-6.97122241801593
H	3.16539653460069	-0.69342712413748	0.84179390110568
H	2.09790975600192	2.19712212752779	-2.11697304394949
C	5.60359704028513	0.26690128939754	1.46244537605619

H	5.11225011769764	-0.24129350950237	2.29475641595184
H	6.23812050380334	-0.46700771215394	0.95881972794606
H	6.25371496831461	1.04014597461032	1.87393371809787
C	4.35135363639774	3.68403662729204	-2.02189622947901
H	4.46450154116270	3.39579667868046	-3.06951432595249
H	3.56135168288358	4.43637613270021	-1.97541983792306
H	5.28297808182950	4.14719938335415	-1.69552338730976

T_1^{min} (**C2** minimum structure)

C	-1.96717043881811	-1.69185706084397	3.83292782852743
C	-2.23527630811403	-3.04921396516853	4.01538258076864
C	-2.71458303183734	-3.82086845392113	2.97191742131367
C	-2.95786462742863	-3.24558749128343	1.72948535267863
C	-2.73473602470138	-1.89173134800539	1.54369089489505
C	-2.23892487738020	-1.12094433739075	2.58600878549772
C	-1.43276062503789	-0.87066818025403	4.93909056260069
C	-0.34677538670002	0.04075680198899	4.73863030164599
C	-0.06003849332273	0.96009037270912	5.78716686043407
C	-0.71660883819633	0.90565404817394	6.98936107562445
C	-1.71291492423258	-0.04982274588592	7.20197258293429
C	-2.06376176846549	-0.91170174708914	6.17585326061939
H	-2.02561564787256	-3.50847077764346	4.97461977376987
H	-2.89302142507089	-4.87855327992152	3.12390057039974
H	-3.33067676641680	-3.85079299994784	0.91219021772512
H	-2.94883347485394	-1.42521375958837	0.59020464733716

H	-2.09472484066356	-0.05652291003043	2.43904561401810
H	0.73682295316626	1.67947570109097	5.64625113106032
H	-0.45353328257722	1.59996338698147	7.77785339736722
H	-2.23406905218736	-0.09498613556939	8.14973613616361
H	-2.89071988627695	-1.59841064579196	6.31441661288490
C	0.46216691219816	0.04956479826718	3.59197865496233
C	0.61848109119252	-1.00568432557050	2.56157943058242
C	1.21341391287667	1.24146647486871	3.04735269327927
C	0.91306858072149	-0.37223181829670	1.32761755776955
C	1.00686569504906	1.11443457094890	1.52618443770274
H	2.28480839301566	1.17546877444019	3.27201268034040
H	0.84517959674044	2.18690766354618	3.44215675823220
H	0.06179333841253	1.56712673659100	1.21263076755503
H	1.79734184505254	1.55539186339672	0.92197929203999
C	0.80007255594318	-2.45940382962035	2.75998515009225
H	0.06731753522622	-3.01549067294786	2.16611051254988
H	1.78608949695094	-2.75509762347726	2.38975997742836
H	0.70122437235130	-2.74629281202273	3.80337759230700
O	1.12674203104758	-1.03686897516194	0.28155616121113
Br	4.63942490549107	-2.26188901156628	-3.09727353387969
Al	2.56155963187710	-1.54502427941043	-3.78572165118807
N	1.35721543196685	-1.59414272722269	-2.17271081010965
C	1.65107006996986	-2.95045507988264	-1.57948737632703
H	2.23371139232052	-3.55026087838800	-2.27557379151966
H	2.25996778113985	-2.82126645159535	-0.68897300770836

C	0.30662480539469	-3.62719925451136	-1.30086375046193
H	0.28052043083529	-4.07982821585658	-0.30895440417212
H	0.14358737011390	-4.42022719690805	-2.03211652888603
C	-0.12126324597342	-1.57138016413965	-2.47095116851434
H	-0.28371946595246	-1.95766468542491	-3.47360055119771
C	-0.74077157036664	-2.53542410808417	-1.46088087688293
H	-0.92606877431279	-2.02959823992696	-0.51501113192245
H	-1.69483436976843	-2.92316865313077	-1.81462166125931
C	-0.54217090251264	-0.08310408625695	-2.35490641077026
O	0.43031468229415	0.49617762609668	-1.50497082537163
B	1.49787053734877	-0.37424127520997	-1.17004017356588
C	-1.93482777874364	0.10029373886105	-1.75010309348973
C	-3.03594567144881	-0.56287312659889	-2.27690790930822
H	-2.90972836128444	-1.23338706940087	-3.12050718345089
C	-4.30947292650646	-0.37286877085074	-1.75554938629295
C	-4.47329831651424	0.51926268917979	-0.70093104806429
H	-5.46532098766964	0.67870250195369	-0.28868949597684
C	-3.39415454288931	1.21665097713587	-0.17270208089762
C	-2.13033060262843	0.99395947203616	-0.70720121062645
H	-1.27793109890278	1.54299585968923	-0.33206559484455
C	-3.59369149483251	2.19623914311621	0.95222942156733
C	5.17397898970721	0.34560492547289	-0.04312798226335
C	3.93302518848614	-0.25526748344901	-0.18830796427217
C	-5.48246966325701	-1.13771856766246	-2.30539766498191
Br	2.77053954652682	0.41992290839597	-4.92688054814623

Br	1.55063552943561	-3.13781259983983	-5.14699524868820
C	-0.54173195580773	0.66650331286812	-3.69143075828825
C	-0.23687439036376	2.02302526230859	-3.68202343016509
H	0.02127534368211	2.49831073803729	-2.74367513440999
C	-0.22053389948070	2.76101261110819	-4.85285616230706
C	-0.53353625051342	2.12202385281493	-6.04996398199573
H	-0.50660084766880	2.68671601102502	-6.97700925012835
C	-0.85933560448145	0.77653123831493	-6.08533043498627
C	-0.87408256352422	0.06092429240672	-4.89071046743492
H	-1.11831735032271	-0.99413831180194	-4.93661575715243
C	0.18976954861225	4.20753370432294	-4.83985223472737
H	-5.32327598436849	-1.40903292697638	-3.34940346450809
H	-6.40183219361717	-0.55476786297108	-2.24113330616177
H	-5.64012752529794	-2.06245487008518	-1.74395779875004
C	-1.13343021597467	0.07033154639121	-7.38334313481739
H	-4.19801792845569	3.04662343604021	0.62944358145648
H	-2.64023394775226	2.58612435683549	1.31173098949926
H	-4.10995756081040	1.73107200706880	1.79509150818954
C	4.43070741598358	2.15044007713712	-1.48416794187226
C	3.19519021657135	1.51978395562923	-1.60243570571769
C	2.92676540177708	0.30988679076707	-0.97410540517823
C	5.40506715753696	1.55248885873468	-0.69867450624757
H	6.37557549972248	2.03066911536345	-0.60070587070003
H	0.00805292492777	4.66257416342483	-3.86541461815044
H	1.25774856741431	4.30047187519859	-5.05483890071903

H	-0.34957968845313	4.78378698004610	-5.59307408482388
H	-1.24644530261999	0.77707058179954	-8.20565797278981
H	-0.30743512046363	-0.60266463689534	-7.62608911165341
H	-2.04142420942695	-0.53305875822561	-7.32449577827170
H	3.76081200141110	-1.20566612197360	0.31029293130152
H	2.42668942033609	1.97412112507854	-2.21818942078938
C	6.26538180470448	-0.31919270416878	0.74962424914048
H	5.85659146343471	-0.94007024407129	1.54849056909153
H	6.86224712200999	-0.96797659576205	0.10345517057913
H	6.93954770308348	0.41273405380189	1.19710150396883
C	4.69662756173925	3.43030672657840	-2.22661570673579
H	4.68084489788639	3.25048181509012	-3.30419382631985
H	3.93221000208102	4.17973023496473	-2.01068637820416
H	5.66873361811840	3.85076742519635	-1.96725770092255

References

[1] D. Lenhart, A. Bauer, A. Pöthig, T. Bach, *Chem. Eur. J.* **2016**, *22*, 6519.

[2] X. Zhao, C. Song, J. D. Rainier, *J. Org. Chem.* **2020**, *85*, 5449.

[3] S. Poplata, T. Bach, *J. Am. Chem. Soc.* **2018**, *140*, 3228.

[4] L. F. Tietze, C. A. Vock, I. K. Krimmelbein, J. M. Wiegand, L. Nacke, T. Ramachandar, K. M. D. Islam, C. Gatz, *Chemistry* **2008**, *14*, 3670.

[5] T. R. Hoye, C. S. Jeffrey, F. Shao, *Nat. Protoc.* **2007**, *2*, 2451.

SC-XRD structure report for compound 14.

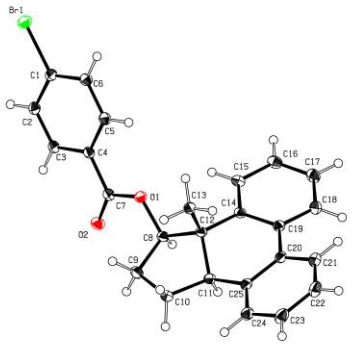

Figure 1: ORTEP representation of the solid-state structure of compound **14** (C = black, O = red, Br = light green) shown with 50 % probability displacement ellipsoids.

A colourless, plate-shaped crystal of $C_{25}H_{21}BrO_2$ coated with perfluorinated ether and fixed on top of a Kapton micro sampler was used for X-ray crystallographic analysis. The X-ray intensity data were collected at 100(2) K on a Bruker D8 VENTURE three-angle diffractometer with a TXS rotating anode with MoK_α radiation (λ=0.71073 Å) using APEX4.[1] The diffractometer was equipped with a Helios optic monochromator, a Bruker PHOTON III detector, and an Oxford Cryostreamlow temperature device.

A matrix scan was used to determine the initial lattice parameters. All data were integrated with the Bruker SAINT V8.40B software package using a narrow-frame algorithm and the reflections were corrected for Lorentz and polarisation effects, scan speed, and background.[2] The integration of the data using a orthorhombic unit cell yielded a total of 162022 reflections within a 2θ range [°] of 4.22 to 66.37 (0.65 Å), of which 7536 were independent. Data were corrected for absorption effects including odd and even ordered spherical harmonics by the multi-scan method (SADABS 2016/2).[3] Space group assignment was based upon systematic absences, E statistics, and successful refinement of the structure.

The structure was solved by direct methods using SHELXT and refined by full-matrix least-squares methods against F^2 by minimizing $\Sigma w(F_o^2 - F_c^2)^2$ using SHELXL in conjunction with SHELXLE.[4-6] All non-hydrogen atoms were refined with anisotropic displacement parameters. Hydrogen atoms were refined isotropically on calculated positions using a riding model with their U_{iso} values constrained to 1.5 times the U_{eq} of their pivot atoms for terminal sp^3 carbon atoms and a C–H distance of 0.98 Å. Non-methyl hydrogen atoms were refined using a riding model with methylene, aromatic, and other C–H distances of 0.99 Å, 0.95 Å, and 1.00 Å, respectively, and U_{iso} values constrained to 1.2 times the U_{eq} of their pivot atoms.

Neutral atom scattering factors for all atoms and anomalous dispersion corrections for the non-hydrogen atoms were taken from International Tables for Crystallography.[7] Supplementary crystallographic data reported in this paper have been deposited with the Cambridge Crystallographic Data Centre (CCDC 2492240) and can be obtained free of charge from The Cambridge Crystallographic Data Centre via www.ccdc.cam.ac.uk/structures.[8] This report and the CIF file were generated using FinalCif.[9]

Table 1. Crystal data and structure refinement for compound 14.

CCDC number	2492240
Empirical formula	$C_{25}H_{21}BrO_2$
Formula weight	433.33
Temperature [K]	100(2)
Crystal system	orthorhombic
Space group (number)	$P2_12_12_1$ (19)
a [Å]	10.3558(5)
b [Å]	12.4450(5)
c [Å]	15.3218(8)
α [°]	90
β [°]	90
γ [°]	90
Volume [Å^3]	1974.64(16)
Z	4
ρ_{calc} [gcm^{-3}]	1.458
μ [mm^{-1}]	2.100
F(000)	888
Crystal size [mm^3]	0.072×0.131×0.171
Crystal colour	colourless
Crystal shape	plate
Radiation	MoK_α (λ=0.71073 Å)
2θ range [°]	4.22 to 66.37 (0.65 Å)
Index ranges	−15 ≤ h ≤ 15<br>−19 ≤ k ≤ 19<br>−23 ≤ l ≤ 23
Reflections collected	162022
Independent reflections	7536<br>R_{int} = 0.0530<br>R_{sigma} = 0.0246
Completeness to θ = 25.242°	100.0
Data / Restraints / Parameters	7536 / 0 / 254
Goodness-of-fit on F^2	1.083
Final *R* indexes [I≥2σ(I)]	R_1 = 0.0264<br>wR_2 = 0.0643
Final *R* indexes [all data]	R_1 = 0.0313<br>wR_2 = 0.0666
Largest peak/hole [eÅ^{-3}]	0.76/−0.25

Flack X parameter	0.024(2)

**Atom**	***x***	***y***	***z***	U_{eq}
Br1	0.90891(2)	0.71618(2)	0.93799(2)	0.02624(5)
O1	0.34428(12)	0.78177(11)	0.71583(8)	0.0194(2)
O2	0.42610(14)	0.94746(10)	0.69538(10)	0.0239(3)
C1	0.76052(17)	0.75789(14)	0.87357(12)	0.0192(3)
C2	0.74979(18)	0.86438(14)	0.84703(13)	0.0212(3)
H2	0.815013	0.915180	0.861113	0.025
C3	0.64198(18)	0.89473(14)	0.79964(13)	0.0202(3)
H3	0.633503	0.967025	0.780574	0.024
C4	0.54565(17)	0.82038(14)	0.77956(12)	0.0177(3)
C5	0.55786(16)	0.71387(14)	0.80751(12)	0.0196(3)
H5	0.491962	0.663196	0.794598	0.023
C6	0.66655(18)	0.68219(14)	0.85423(12)	0.0203(3)
H6	0.676251	0.609742	0.872619	0.024
C7	0.43413(16)	0.85822(14)	0.72626(12)	0.0187(3)
C8	0.23457(17)	0.81024(13)	0.66139(12)	0.0181(3)
H8	0.265763	0.837843	0.603841	0.022
C9	0.14435(19)	0.89432(15)	0.70434(14)	0.0244(4)
H9A	0.157172	0.965971	0.677643	0.029
H9B	0.161702	0.899591	0.767735	0.029
C10	0.00493(17)	0.85346(15)	0.68768(13)	0.0210(3)
H10A	−0.033563	0.823589	0.741618	0.025
H10B	−0.051022	0.911890	0.665489	0.025
C11	0.02379(16)	0.76588(13)	0.61869(12)	0.0183(3)
H11	0.041794	0.802930	0.562063	0.022
C12	0.15003(15)	0.71024(14)	0.64718(11)	0.0161(3)
C13	0.13384(17)	0.64762(14)	0.73317(12)	0.0190(3)
H13A	0.065460	0.593832	0.726372	0.028
H13B	0.110578	0.697538	0.780076	0.028
H13C	0.215188	0.611635	0.747798	0.028
C14	0.19101(16)	0.63302(13)	0.57641(11)	0.0166(3)
C15	0.32032(17)	0.61335(13)	0.55713(13)	0.0203(3)
H15	0.385930	0.652722	0.586377	0.024
C16	0.3541(2)	0.53611(15)	0.49508(13)	0.0235(3)
H16	0.442510	0.521502	0.483435	0.028
C17	0.2586(2)	0.48093(16)	0.45060(13)	0.0249(4)
H17	0.281600	0.429350	0.407607	0.030
C18	0.1293(2)	0.50066(16)	0.46858(13)	0.0231(3)
H18	0.064390	0.463538	0.436756	0.028
C19	0.09338(19)	0.57470(13)	0.53312(11)	0.0186(3)
C20	−0.04384(16)	0.58947(14)	0.55913(12)	0.0188(3)
C21	−0.1367(2)	0.50972(16)	0.54514(13)	0.0246(4)
H21	−0.112498	0.444344	0.517620	0.030
C22	−0.26411(19)	0.52512(17)	0.57111(14)	0.0279(4)
H22	−0.326478	0.470499	0.561153	0.033
C23	−0.3000(2)	0.62037(19)	0.61158(14)	0.0288(4)
H23	−0.387169	0.631040	0.628923	0.035
C24	−0.20882(17)	0.70015(16)	0.62677(13)	0.0242(4)
H24	−0.233960	0.765287	0.654277	0.029
C25	−0.08077(18)	0.68506(14)	0.60186(11)	0.0192(3)

U_{eq} is defined as 1/3 of the trace of the orthogonalized U_{ij} tensor.

Table 1. Anisotropic displacement parameters (Å^2) for compound 14. The anisotropic displacement factor exponent takes the form: $-2\pi^2[\,h^2(a^*)^2U_{11} + k^2(b^*)^2U_{22} + \ldots + 2hka^*b^*U_{12}\,]$

Atom	U_{11}	U_{22}	U_{33}	U_{23}	U_{13}	U_{12}
Br1	0.02296(8)	0.02580(8)	0.02996(9)	0.00374(7)	−0.00653(7)	−0.00048(7)
O1	0.0180(5)	0.0164(5)	0.0238(6)	0.0019(5)	−0.0037(4)	−0.0011(5)
O2	0.0241(7)	0.0165(5)	0.0311(7)	0.0029(5)	−0.0023(5)	−0.0012(5)
C1	0.0190(7)	0.0197(7)	0.0190(8)	0.0002(6)	−0.0018(6)	0.0000(6)
C2	0.0222(8)	0.0177(7)	0.0236(8)	−0.0012(6)	−0.0034(6)	−0.0033(6)
C3	0.0226(8)	0.0146(6)	0.0234(8)	−0.0008(6)	−0.0024(6)	−0.0022(6)
C4	0.0183(7)	0.0159(6)	0.0188(7)	−0.0013(5)	0.0000(6)	−0.0007(5)
C5	0.0197(7)	0.0160(6)	0.0230(7)	0.0008(6)	−0.0003(6)	−0.0027(6)
C6	0.0219(8)	0.0165(7)	0.0226(8)	0.0020(6)	−0.0009(6)	−0.0011(6)
C7	0.0194(8)	0.0166(7)	0.0200(7)	−0.0011(6)	0.0009(6)	−0.0003(5)
C8	0.0180(7)	0.0148(6)	0.0216(8)	0.0014(5)	−0.0022(6)	0.0013(5)
C9	0.0218(8)	0.0179(7)	0.0336(10)	−0.0045(7)	−0.0017(7)	0.0026(6)
C10	0.0197(7)	0.0198(7)	0.0235(8)	−0.0017(6)	−0.0011(6)	0.0043(6)
C11	0.0177(7)	0.0181(7)	0.0191(7)	0.0003(6)	−0.0012(6)	0.0037(5)
C12	0.0159(6)	0.0144(6)	0.0180(7)	0.0015(6)	−0.0009(5)	0.0015(6)
C13	0.0204(7)	0.0189(7)	0.0177(7)	0.0011(6)	−0.0007(6)	0.0006(6)
C14	0.0187(7)	0.0149(6)	0.0162(7)	0.0024(5)	0.0004(5)	0.0020(5)
C15	0.0195(7)	0.0185(6)	0.0227(8)	0.0020(6)	0.0030(7)	0.0023(5)
C16	0.0242(8)	0.0230(8)	0.0232(8)	0.0024(7)	0.0065(7)	0.0046(7)
C17	0.0302(9)	0.0255(8)	0.0192(9)	−0.0027(6)	0.0050(7)	0.0041(7)
C18	0.0260(8)	0.0238(8)	0.0194(8)	−0.0027(6)	−0.0011(6)	0.0014(6)
C19	0.0202(7)	0.0191(6)	0.0166(6)	0.0002(5)	−0.0013(6)	0.0027(6)
C20	0.0179(6)	0.0220(7)	0.0165(7)	−0.0005(6)	−0.0023(6)	0.0017(6)
C21	0.0251(8)	0.0266(8)	0.0223(9)	−0.0031(7)	−0.0040(6)	−0.0009(7)
C22	0.0230(8)	0.0335(9)	0.0270(10)	−0.0047(8)	−0.0034(7)	−0.0057(7)
C23	0.0193(8)	0.0403(11)	0.0268(10)	−0.0048(8)	−0.0023(7)	−0.0005(7)
C24	0.0190(7)	0.0290(9)	0.0247(8)	−0.0045(7)	−0.0021(6)	0.0032(7)
C25	0.0190(7)	0.0218(7)	0.0167(7)	−0.0004(5)	−0.0032(6)	0.0022(6)

Table 2. Bond lengths and angles for compound 14.

Atom–Atom	Length [Å]
Br1–C1	1.8987(18)
O1–C7	1.340(2)
O1–C8	1.453(2)
O2–C7	1.210(2)
C1–C6	1.386(2)
C1–C2	1.391(2)
C2–C3	1.384(3)
C2–H2	0.9500
C3–C4	1.395(2)
C3–H3	0.9500
C4–C5	1.399(3)
C4–C7	1.491(2)
C5–C6	1.391(2)
C5–H5	0.9500
C6–H6	0.9500
C8–C12	1.537(2)
C8–C9	1.549(3)
C8–H8	1.0000

C9–C10	1.552(3)
C9–H9A	0.9900
C9–H9B	0.9900
C10–C11	1.531(2)
C10–H10A	0.9900
C10–H10B	0.9900
C11–C25	1.500(2)
C11–C12	1.542(2)
C11–H11	1.0000
C12–C14	1.510(2)
C12–C13	1.540(2)
C13–H13A	0.9800
C13–H13B	0.9800
C13–H13C	0.9800
C14–C15	1.393(2)
C14–C19	1.410(2)
C15–C16	1.397(3)
C15–H15	0.9500
C16–C17	1.383(3)
C16–H16	0.9500
C17–C18	1.390(3)
C17–H17	0.9500
C18–C19	1.402(2)
C18–H18	0.9500
C19–C20	1.487(3)
C20–C21	1.398(3)
C20–C25	1.411(2)
C21–C22	1.392(3)
C21–H21	0.9500
C22–C23	1.389(3)
C22–H22	0.9500
C23–C24	1.390(3)
C23–H23	0.9500
C24–C25	1.393(3)
C24–H24	0.9500

**Atom–Atom–Atom**	**Angle [°]**
C7–O1–C8	115.98(14)
C6–C1–C2	121.93(17)
C6–C1–Br1	119.57(13)
C2–C1–Br1	118.50(14)
C3–C2–C1	118.54(16)
C3–C2–H2	120.7
C1–C2–H2	120.7
C2–C3–C4	120.76(16)
C2–C3–H3	119.6
C4–C3–H3	119.6
C3–C4–C5	119.77(17)
C3–C4–C7	117.72(16)
C5–C4–C7	122.49(16)
C6–C5–C4	119.95(16)
C6–C5–H5	120.0
C4–C5–H5	120.0
C1–C6–C5	119.04(16)

C1–C6–H6	120.5
C5–C6–H6	120.5
O2–C7–O1	123.86(16)
O2–C7–C4	123.88(16)
O1–C7–C4	112.26(15)
O1–C8–C12	109.22(13)
O1–C8–C9	113.10(15)
C12–C8–C9	105.28(14)
O1–C8–H8	109.7
C12–C8–H8	109.7
C9–C8–H8	109.7
C8–C9–C10	105.66(14)
C8–C9–H9A	110.6
C10–C9–H9A	110.6
C8–C9–H9B	110.6
C10–C9–H9B	110.6
H9A–C9–H9B	108.7
C11–C10–C9	103.19(14)
C11–C10–H10A	111.1
C9–C10–H10A	111.1
C11–C10–H10B	111.1
C9–C10–H10B	111.1
H10A–C10–H10B	109.1
C25–C11–C10	120.26(15)
C25–C11–C12	111.06(14)
C10–C11–C12	103.43(14)
C25–C11–H11	107.1
C10–C11–H11	107.1
C12–C11–H11	107.1
C14–C12–C8	117.19(14)
C14–C12–C13	108.84(14)
C8–C12–C13	110.52(14)
C14–C12–C11	108.70(13)
C8–C12–C11	99.17(13)
C13–C12–C11	112.16(14)
C12–C13–H13A	109.5
C12–C13–H13B	109.5
H13A–C13–H13B	109.5
C12–C13–H13C	109.5
H13A–C13–H13C	109.5
H13B–C13–H13C	109.5
C15–C14–C19	119.93(16)
C15–C14–C12	122.30(15)
C19–C14–C12	117.64(15)
C14–C15–C16	120.42(18)
C14–C15–H15	119.8
C16–C15–H15	119.8
C17–C16–C15	119.85(18)
C17–C16–H16	120.1
C15–C16–H16	120.1
C16–C17–C18	120.25(17)
C16–C17–H17	119.9
C18–C17–H17	119.9
C17–C18–C19	120.77(18)

C17–C18–H18	119.6
C19–C18–H18	119.6
C18–C19–C14	118.69(17)
C18–C19–C20	121.57(17)
C14–C19–C20	119.69(15)
C21–C20–C25	118.90(17)
C21–C20–C19	121.88(16)
C25–C20–C19	119.18(15)
C22–C21–C20	120.69(18)
C22–C21–H21	119.7
C20–C21–H21	119.7
C23–C22–C21	119.95(18)
C23–C22–H22	120.0
C21–C22–H22	120.0
C22–C23–C24	120.16(19)
C22–C23–H23	119.9
C24–C23–H23	119.9
C23–C24–C25	120.32(18)
C23–C24–H24	119.8
C25–C24–H24	119.8
C24–C25–C20	119.94(17)
C24–C25–C11	123.35(16)
C20–C25–C11	116.71(16)

Table 3. Torsion angles for compound 14.

**Atom–Atom–Atom–Atom**	**Torsion Angle [°]**
C6–C1–C2–C3	−0.3(3)
Br1–C1–C2–C3	179.65(14)
C1–C2–C3–C4	0.5(3)
C2–C3–C4–C5	0.0(3)
C2–C3–C4–C7	−178.38(17)
C3–C4–C5–C6	−0.8(3)
C7–C4–C5–C6	177.50(17)
C2–C1–C6–C5	−0.5(3)
Br1–C1–C6–C5	179.57(14)
C4–C5–C6–C1	1.0(3)
C8–O1–C7–O2	2.1(3)
C8–O1–C7–C4	−177.40(14)
C3–C4–C7–O2	4.4(3)
C5–C4–C7–O2	−173.88(18)
C3–C4–C7–O1	−176.11(16)
C5–C4–C7–O1	5.6(2)
C7–O1–C8–C12	173.16(14)
C7–O1–C8–C9	−69.96(19)
O1–C8–C9–C10	−135.90(16)
C12–C8–C9–C10	−16.72(19)
C8–C9–C10–C11	−13.25(19)
C9–C10–C11–C25	163.06(16)
C9–C10–C11–C12	38.51(18)
O1–C8–C12–C14	−82.25(18)
C9–C8–C12–C14	156.01(15)
O1–C8–C12–C13	43.19(18)
C9–C8–C12–C13	−78.54(17)
O1–C8–C12–C11	161.12(13)
C9–C8–C12–C11	39.39(17)
C25–C11–C12–C14	58.45(18)
C10–C11–C12–C14	−171.23(14)
C25–C11–C12–C8	−178.64(14)
C10–C11–C12–C8	−48.31(16)
C25–C11–C12–C13	−61.95(19)
C10–C11–C12–C13	68.37(17)
C8–C12–C14–C15	34.5(2)
C13–C12–C14–C15	−91.76(19)
C11–C12–C14–C15	145.81(16)
C8–C12–C14–C19	−149.60(15)
C13–C12–C14–C19	84.12(17)
C11–C12–C14–C19	−38.31(19)
C19–C14–C15–C16	0.0(3)
C12–C14–C15–C16	175.80(16)
C14–C15–C16–C17	1.9(3)
C15–C16–C17–C18	−1.2(3)
C16–C17–C18–C19	−1.4(3)
C17–C18–C19–C14	3.3(3)
C17–C18–C19–C20	−174.17(18)
C15–C14–C19–C18	−2.5(2)
C12–C14–C19–C18	−178.52(15)
C15–C14–C19–C20	174.94(16)
C12–C14–C19–C20	−1.0(2)
C18–C19–C20–C21	22.4(3)
C14–C19–C20–C21	−155.02(17)
C18–C19–C20–C25	−159.84(17)

C14–C19–C20–C25	22.7(2)	C19–C20–C25–C24	−179.92(17)
C25–C20–C21–C22	1.4(3)	C21–C20–C25–C11	177.41(16)
C19–C20–C21–C22	179.20(18)	C19–C20–C25–C11	−0.4(2)
C20–C21–C22–C23	−0.2(3)	C10–C11–C25–C24	18.6(3)
C21–C22–C23–C24	−0.4(3)	C12–C11–C25–C24	139.46(17)
C22–C23–C24–C25	−0.2(3)	C10–C11–C25–C20	−160.86(16)
C23–C24–C25–C20	1.5(3)	C12–C11–C25–C20	−40.0(2)
C23–C24–C25–C11	−177.94(18)		
C21–C20–C25–C24	−2.1(3)		

References

[1] *APEX4 Suite of Crystallographic Software, Version 2021-10.0,* Bruker AXS Inc., Madison, Wisconsin, USA, **2021**.

[2] Bruker, *SAINT*, *V8.40B*, Bruker AXS Inc., Madison, Wisconsin, USA.

[3] L. Krause, R. Herbst-Irmer, G. M. Sheldrick, D. Stalke, *J. Appl. Cryst.* **2015**, *48*, 3–10, doi:10.1107/S1600576714022985.

[4] G. M. Sheldrick, *Acta Cryst.* **2015**, *A71*, 3–8, doi:10.1107/S2053273314026370.

[5] G. M. Sheldrick, *Acta Cryst.* **2015**, *C71*, 3–8, doi:10.1107/S2053229614024218.

[6] C. B. Huebschle, G. M. Sheldrick, B. Dittrich, *J. Appl. Cryst.* **2011**, *44*, 1281–1284, doi:10.1107/S0021889811043202.

[7] Ed. E. Prince, *International Tables for Crystallography Volume C, Mathematical, Physical and Chemical Tables*, International Union of Crystallography, Chester, England, **2006**, 500–502; 219–222; 193–199.

[8] C. R. Groom, I. J. Bruno, M. P. Lightfoot, S. C. Ward, *Acta Cryst.* **2016**, *B72*, 171–179, doi:10.1107/S2052520616003954.

[9] D. Kratzert, *FinalCif*, *V153*, https://dkratzert.de/finalcif.html.
